# Supplementary material for: Light-induced primary amines and o-nitrobenzyl alcohols cyclization as a versatile photoclick reaction for modular conjugation
Source: Nat Commun. 2020 Oct 29;11:5472. doi: 10.1038/s41467-020-19274-y (PMC7596520; doi:10.1038/s41467-020-19274-y)
Supplement: Supplementary file 1 — Supplementary Information [file 41467_2020_19274_MOESM1_ESM.pdf]

Supplementary Information

Light-induced primary amines and o-nitrobenzyl alcohols cyclization as a versatile photoclick reaction for modular conjugation

An-Di Guo[1, 2, 4], Dan Wei[1, 2, 4], Hui-Jun Nie[1, 4], Hao Hu[3], Chengyuan Peng[3], Shao-Tong, Li[1, 2], Ke-Nian Yan[1, 2], Bin-Shan Zhou[1], Lei Feng[1, 2], Chao Fang[1], Minjia Tan[3], Ruimin Huang[3], Xiao-Hua Chen[1]*

[1]Chinese Academy of Sciences Key Laboratory of Receptor Research, Shanghai Institute of Materia Medica, Chinese Academy of Sciences, Shanghai, 201203, China

[2]University of Chinese Academy of Sciences, No. 19A Yuquan Road, Beijing 100049, China

[3]State Key Laboratory of Drug Research, Shanghai Institute of Materia Medica, Chinese Academy of Sciences, Shanghai 201203, China

[4]These authors contributed equally: An-Di Guo, Dan Wei, Hui-Jun Nie.

*Correspondence: xhchen@simm.ac.cn

Table of Contents

1. General Information……………..……………...………………………………….…………………3

2. Supplementary Tables……………..……………………………………………..……………………4

3. Supplementary Figures………………………………………………………………………………10

4. Experimental Procedures and UPLC-MS Analysis of Small Molecules and Peptides ……….……24

5. Experimental Procedures for biomacromolecules…………………………………..……………43

6. Synthesis Procedures and Compounds Date…………………………………………..……..……49

7. UPLC-MS Analysis of Reactions and Products of Small Molecules…………..……………...…...62

8. ^{1}H and ^{13}C NMR of Indazolone Product…………………………………………………………….88

9. Full Gel Scan……………………………………………………………………………………….111

10. Supplementary References………………………………………...……………………….112

1. General Information

Unless otherwise noted, all the materials were obtained from commercial suppliers and were used without further purification. Analytical TLC was performed using pre-coated plates (HSGF254) and visualized with UV light or an I_2 chamber. Silica chromatography was performed using the indicated solvent system on Sinopharm Chemical Reagent silica gel (200−300 mesh). ^{1}H NMR spectra and ^{13}C NMR spectra were obtained on a Bruker AVANCE III 400 (400 MHz), Bruker AVANCE III 500 (500 MHz) and Bruker AVANCE III 600 (600 MHz) NMR spectrometer. Chemical shifts were reported in parts per million (ppm) on the δ scale from an internal standard (NMR descriptions: s, singlet; d, doublet; t, triplet; q, quartet; m, multiplet; br, broad). Coupling constants, *J*, are reported in Hertz. For HRMS analysis, samples were analyzed by flow-injection analysis into a Agilent 1290-6545 UHPLC-QTOF. LCMS analysis was performed on Waters UPLC-MS（ESI）with ACQUITY UPLC BEH C18 1.7 μm column (UPLC: Waters HPLC H-CLASS, MS: Waters SQ Detector 2), using 5-95% acetonitrile gradient (0.01% formic acid or 0.05% trifluoroacetic acid added) for 10 min or 5-50-95% acetonitrile (0.01% formic acid or 0.05% trifluoroacetic acid added) gradient for 11 min. Protein molecular weight was determined by Waters XEVO G2-XS. Protein deconvolution was performed using Waters UNIFI. Peptide LC-MS/MS analysis was performed by Orbitrap fusion mass spectrometer.

Light-induced reactions were conducted with Shanghai Heqi glassware B-002601 40×25 mm flat bottom flask and Titan 7.5×12.5×45 mm Quartz cuvette. Using ZF-7A 16 W 365 nm UV as light source.

Peptide was purchased from Genscript. Lysozyme was purchased from Sinopharm. Myoglobin and chymotrypsinogen A were purchased from Sigma. Sequence information for Lysozyme, chymotrypsinogen A, myoglobin were obtained from Protein Data Bank. TAMRA-N_3 (Cat. 760757), CuSO4 (Cat. 451657), THPTA (Cat. 762342), Sodium ascorbate (Cat. A4034), TCEP•HCl (Cat. C4706) and TBTA (Cat. 678937) were purchased from Sigma. Hoechst 33342 (Cat. H1399), Rhodamine 123 (Cat. R302), Fluorobrite DMEM (Cat. A1896701), BCA protein assay kit (Cat. 23225) and Chambered Coverglass (Cat. 155411) were purchased from Thermo. Ni-NTA agarose beads (Cat. 1018244) were purchased from Qiagen. PD-10 desalting column (Cat.17085101) was purchased from GE.

Human cell line SK-Br-3 was grown in DMEM containing 20% FBS and 1% PS at 37 ^{o}C with 5% CO_2. Human cell line MDA-MB-468 was grown in L-15 medium containing 10% FBS and 1% PS at 37 ^{o}C without CO_2. Human cell line Jurkat was grown in RPMI-1640 containing 10% FBS and 1% PS at 37 ^{o}C with 5% CO_2. Human cell line K562 was grown in IMDM containing 10% FBS and 1% PS at 37 ^{o}C with 5% CO_2. Human cell line HEK-293T was grown in DMEM containing 10% FBS and 1% PS at 37 ^{o}C with 5% CO2. All cell lines were obtained from the Cell Bank of the Chinese Academy of Sciences (Shanghai, China).

The cell Fluorescence confocal imaging was performed with Leica TCS-SP8 STED (Software: Leica LAS X 2.0.1). The fluorescent gel was visualized by GE Typhoon FLA 9500 and CLiNX ChemiScope 6300. Pearson’s r was calculated by the Coloc2 plugin in ImageJ (Fiji version). Docking of STK24 (PDB code: 4QO9) with Probe-1 and Nitroso-Probe-1 was performed with Schrodinger_Suites_2018-1. Cell viability CCK8 kit reading was performed by Tecan microplate reader (Software: Tecan Sparkcontrol 2.3). UV-Vis spectrum was measured by SHIMADZU UV-2600 (Software: UV Probe 2.70). LC-MS spectrum within the supplementary information was collected by Masslynx v4.1 software. Protein molecular weight deconvolution is performed by Waters UNIFI Workstation 1.9 software.

2. Supplementary Tables

Supplementary Table 1 | Reactivity of different o-NBA substrates (2.0 mM) and Cbz-Lys-OMe (0.5 mM). Yields were determined by ratio of peak area value of experiment to that of internal standard product on reverse-phase HPLC, the products of (**1, S1-S5**) are reported as an average of three independent trials.

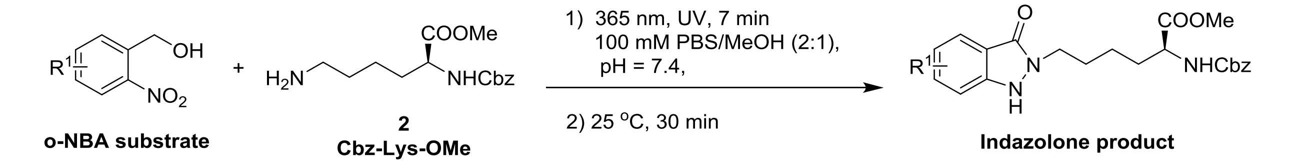

o-NBA substrates and yields of indazolone products

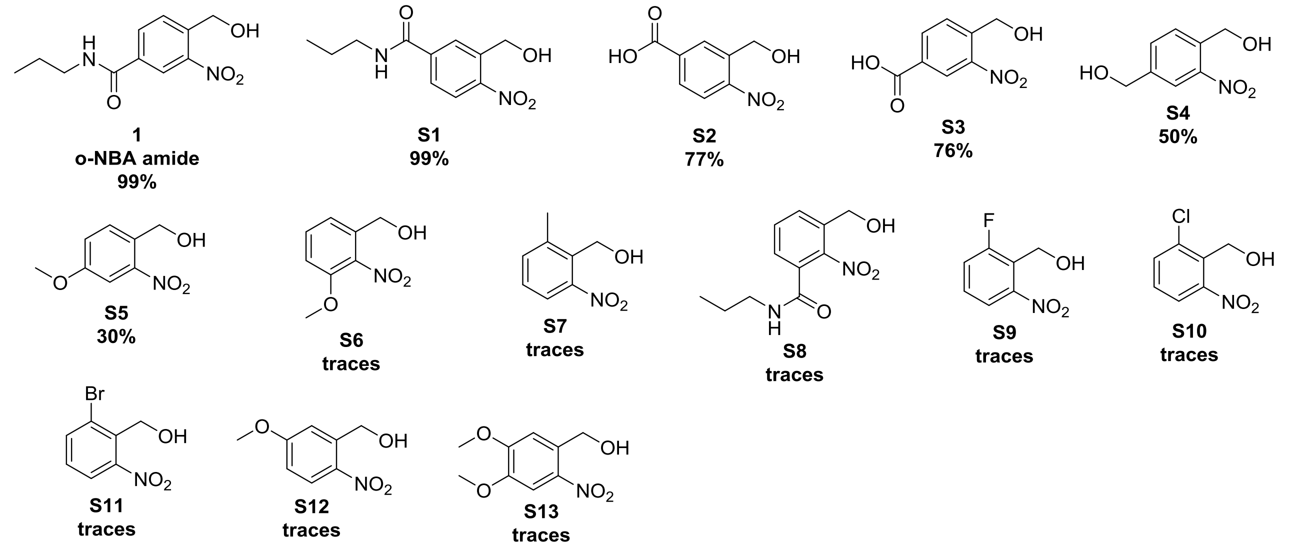

Supplementary Table 2 | Reactivity of o-NBA amide 1 and Cbz-Lys-OMe under different conditions. Yields were determined by ratio of peak area value of experiment to that of internal standard product on reverse-phase HPLC, are reported as an average of three independent trials.

**Entry**	**o-NBA amide 1 concentration**	**Cbz-Lys-OMe concentration**	**Buffer**	**Reaction conditions**	**Yields**
1	2.0 mM	0.5 mM	100 mM PBS/MeOH (2:1) pH = 7.4	Mix after o-NBA irradiated One portion	95%
2	2.0 mM	0.5 mM	100 mM PBS/MeOH (2:1) pH = 7.4	Irradiated in a mixture	> 98%
3	2.0 mM	0.5 mM	100 mM PBS/MeOH (2:1) pH = 7.4	Mix after o-NBA irradiated 3 aliquots	95%
4	2.0 mM	0.5 mM	100 mM PBS/MeOH (2:1) pH = 7.4	Mix after o-NBA irradiated 5 aliquots	95%
5	2.0 mM	0.5 mM	100 mM PBS/DMSO (2:1) pH = 7.4	Irradiated in a mixture	93%
6	2.0 mM	0.5 mM	100 mM PBS/DMSO (2:1) pH = 8.0	Irradiated in a mixture	> 98%
7	2.0 mM	0.5 mM	100 mM PBS/DMSO (2:1) pH = 8.0	Mix after o-NBA irradiated 3 aliquots	> 98%
8	2.0 mM	0.5 mM	100 mM PBS/MeOH (2:1) pH = 6.0	Irradiated in a mixture	31%
9	2.0 mM	0.5 mM	100 mM PBS/MeOH (2:1) pH = 8.0	Irradiated in a mixture	> 98%
10	2.0 mM	0.5 mM	100 mM PBS/ACN (2:1) pH = 7.4	Irradiated in a mixture	> 98%
11	0.4 mM	0.1 mM	20 mM PBS/MeOH (1:1) pH = 7.4	Irradiated in a mixture	> 98%
12	0.8 mM	0.1 mM	20 mM PBS/MeOH (1:1) pH = 7.4	Irradiated in a mixture	> 98%
13	0.2 mM	0.05 mM	20 mM PBS/MeOH (1:1) pH = 7.4	Irradiated in a mixture	> 98%
14	0.4 mM	0.05 mM	20 mM PBS/MeOH (1:1) pH = 7.4	Irradiated in a mixture	> 98%
15	0.2 mM	0.02 mM	20 mM PBS/MeOH (1:1) pH = 7.4	Irradiated in a mixture	98%
16	0.2 mM	0.01 mM	20 mM PBS/MeOH (1:1) pH = 7.4	Irradiated in a mixture	98%
17	0.1 mM	0.005 mM	20 mM PBS/MeOH (1:1) pH = 7.4	Irradiated in a mixture	83%
18	0.04 mM	0.002 mM	20 mM PBS/MeOH (1:1) pH = 7.4	Irradiated in a mixture	78%

Supplementary Table 3 | Selectivity of o-NBA amide 1 towards other amino acids

Entry	The equivalent of o-NBA amide 1 : Cbz-Lys-OMe : other amino acids	Other amino acids	Yield of Cbz-Lys-OMe	Yield of the reaction with other amino acids
1	4:1:1	NH_2-Lys (Cbz)-OMe	96%	<1%
2	8:1:1	Cbz-Cys-OMe	>99%	Cbz-Cys-OMe reduced with DTT before reaction, After reaction Cys-dimer $(\text{Cbz-Cys-OMe})_2$, and thiohydroxylamine byproduct were found
3	4:1:1	Cbz-Ser-OMe	>99%	N.D.
4	4:1:1	Cbz-Tyr-OMe	>99%	N.D.
5	4:1:1	Cbz-Glu-OMe	>99%	N.D.
6	4:1:1	Cbz-Asn-OH	>99%	N.D.
7	4:1:1	Cbz-Arg-OH	>99%	N.D.
8	4:1:1	Cbz-His-OH	>99%	N.D.
9	4:1:1	Cbz-Trp-OH	>99%	N.D.

N.D.: no detected on UPLC-MS. Each of the reaction mixture was collected, diluted with acetonitrile/H_2O and analysed by UPLC-MS.

Supplementary Table 4 | Kinases identified by Probe-1 in Jurkat and K562 cells.

Accession number	Name	Gene	Jurkat	K562
P06493	Cyclin-dependent kinase 1	CDK1	+	+
E9PC69	Non-specific serine/threonine protein kinase	MARK2	-	+
B4E0Y9	Serine/threonine-protein kinase 26	STK26	+	+
P78527	DNA-dependent protein kinase catalytic subunit	PRKDC	+	-
B4DR80	cDNA FLJ61159, highly similar to Serine/threonine-protein kinase 24	STK24	+	+
Q7KZI7-15	Isoform 15 of Serine/threonine-protein kinase MARK2	MARK2	+	-
Q00534	Cyclin-dependent kinase 6	CDK6	+	+
P27448	MAP/microtubule affinity-regulating kinase 3	MARK3	+	+
P06239	Tyrosine-protein kinase Lck	LCK	+	-
G3V5T9	Cyclin-dependent kinase 2	CDK2	+	+
Q13177	Serine/threonine-protein kinase PAK 2	PAK2	+	+
Q00536	Cyclin-dependent kinase 16	CDK16	-	+
Q13043	Serine/threonine-protein kinase 4	STK4	+	+
P19784	Casein kinase II subunit alpha	CSNK2A2	+	+
O00506	Serine/threonine-protein kinase 25	STK25	+	+
Q8TD19	Serine/threonine-protein kinase Nek9	NEK9	+	+
A0A2R8YD58	Casein kinase II subunit alpha	CSNK2A1	+	+
Q14012	Calcium/calmodulin-dependent protein kinase type 1	CAMK1	-	+
P17252	Protein kinase C alpha type	PRKCA	+	-
Q13188	Serine/threonine-protein kinase 3	STK3	+	+
P50750	Cyclin-dependent kinase 9	CDK9	+	+
P33981	Dual specificity protein kinase TTK	TTK	+	+
Q00535	Cyclin-dependent-like kinase 5	CDK5	+	+
Q9NSY1	BMP-2-inducible protein kinase	BMP2K	-	+
Q8IU85	Calcium/calmodulin-dependent protein kinase type 1D	CAMK1D	+	-
Q86TW2	Uncharacterized aarF domain-containing protein kinase 1	ADCK1	+	+
Q96GD4	Aurora kinase B	AURKB	+	+
Q5SWX3	Calcium/calmodulin-dependent protein kinase (CaM kinase) II gamma, isoform CRA_n	CAMK2G	-	+
F5H6Z0	Cyclin-dependent kinase 17	CDK17	+	+
Q13546	Receptor-interacting serine/threonine-protein kinase 1	RIPK1	+	+
O43293	Death-associated protein kinase 3	DAPK3	+	+
A0A2R8Y7H4	Ribose-phosphate pyrophosphokinase	PRPS1	+	-
Q13131	5-AMP-activated protein kinase catalytic subunit alpha-1	PRKAA1	+	+
A0A2Q3DQE3	Calcium/calmodulin-dependent protein kinase (CaM kinase) II gamma, isoform CRA_d	CAMK2G	+	-
Q9UEW8	STE20/SPS1-related proline-alanine-rich protein kinase	STK39	+	-
P07948-2	Isoform 2 of Tyrosine-protein kinase Lyn	LYN	-	+
O14965	Aurora kinase A	AURKA	+	+
P31751	RAC-beta serine/threonine-protein kinase	AKT2	+	+
A0A087WZU2	Cyclin-dependent kinase 16	CDK16	+	-
A0A0D9SEY1	Mitogen-activated protein kinase kinase kinase kinase 4, isoform CRA_a	MAP4K4	+	+
P19525	Interferon-induced, double-stranded RNA-	EIF2AK2	+	+

	activated protein kinase			
O95747	Serine/threonine-protein kinase OSR1	OXSR1	+	-
Q16512	Serine/threonine-protein kinase N1	PKN1	+	-
P04183	Thymidine kinase, cytosolic	TK1	-	+
P25098	Beta-adrenergic receptor kinase 1	GRK2	+	-
Q9HBH9	MAP kinase-interacting serine/threonine-protein kinase 2	MKNK2	-	+
P05771-2	Isoform Beta-II of Protein kinase C beta type	PRKCB	+	+
P17858	ATP-dependent 6-phosphofructokinase, liver type	PFKL	+	+
P49841	Glycogen synthase kinase-3 beta	GSK3B	+	-
Q96L34	MAP/microtubule affinity-regulating kinase 4	MARK4	+	+
P49840	Glycogen synthase kinase-3 alpha	GSK3A	-	+
A0A1W2PS05	Phosphatidylinositol 3-kinase catalytic subunit type 3	PIK3C3	+	+
P35626	Beta-adrenergic receptor kinase 2	GRK3	+	-
P31749	RAC-alpha serine/threonine-protein kinase	AKT1	+	+
Q16566	Calcium/calmodulin-dependent protein kinase type IV	CAMK4	+	-
Q99986	Serine/threonine-protein kinase VRK1	VRK1	+	-
J3QRU1	Tyrosine-protein kinase	YES1	-	+
Q92616	eIF-2-alpha kinase activator GCN1	GCN1	+	-
Q5JPT1	SH3 domain-containing kinase-binding protein 1 (Fragment)	SH3KBP1	+	-
Q9UKE5	TRAF2 and NCK-interacting protein kinase	TNIK	-	+
O14976	Cyclin-G-associated kinase	GAK	+	-
Q86UE8	Serine/threonine-protein kinase tousled-like 2	TLK2	+	+
Q9UKI8	Serine/threonine-protein kinase tousled-like 1	TLK1	+	+
O96013	Serine/threonine-protein kinase PAK 4	PAK4	+	+
J3QSD7	Cyclin-dependent kinase 12 (Fragment)	CDK12	+	+
P30085	UMP-CMP kinase	CMPK1	+	-
H3BPN6	Serine/threonine-protein kinase ULK3	ULK3	+	-
A8MT37	Glycogen synthase kinase-3 alpha	GSK3A	+	-
E9PER6	3-phosphoinositide-dependent protein kinase 1	PDPK1	+	-
D6R9G1	Cyclin-dependent kinase 7	CDK7	-	+
Q9ULX6	A-kinase anchor protein 8-like	AKAP8L	-	+
Q8N4C8	Misshapen-like kinase 1	MINK1	-	+
Q9BVS4	Serine/threonine-protein kinase RIO2	RIOK2	-	+
P06241	Tyrosine-protein kinase Fyn	FYN	+	-
Q9UHD2	Serine/threonine-protein kinase TBK1	TBK1	-	+
D6R938	Calcium/calmodulin-dependent protein kinase (CaM kinase) II delta, isoform CRA_e	CAMK2D	+	-
A0A2R8Y891	ATP-dependent 6-phosphofructokinase	PFKM	-	+
H3BV73	Protein kinase C beta type (Fragment)	PRKCB	+	-
F5GY51	Serine/threonine-protein kinase 38-like (Fragment)	STK38L	+	-
P28482	Mitogen-activated protein kinase 1	MAPK1	+	-
O60566	Mitotic checkpoint serine/threonine-protein kinase BUB1 beta	BUB1B	-	+
D6RAD4	Cyclin-dependent kinase 7	CDK7	+	-
O75063	Glycosaminoglycan xylosylkinase	FAM20B	-	+
K7EP59	Serine/threonine-protein kinase STK11	STK11	+	+
E5RJR5	S-phase kinase-associated protein 1	SKP1	+	+
Q9NSY1-2	Isoform 2 of BMP-2-inducible protein kinase	BMP2K	+	-
Q15136	Protein kinase A-alpha (Fragment)	PRKACA	-	+
F8VZ51	Cyclin-dependent kinase 4 (Fragment)	CDK4	-	+

H0YCY6	Triokinase/FMN cyclase (Fragment)	TKFC	+	-
Q9NWT1	p21-activated protein kinase-interacting protein 1	PAK1IP1	-	+
Q2M2I8	AP2-associated protein kinase 1	AAK1	+	+
Q15126	Phosphomevalonate kinase	PMVK	+	-
Q02750	Dual specificity mitogen-activated protein kinase kinase 1	MAP2K1	+	-
Q9H0C8	Integrin-linked kinase-associated serine/threonine phosphatase 2C	ILKAP	+	-
H3BLV9	SRSF protein kinase 1 (Fragment)	SRPK1	-	+
F8WBF5	Dual-specificity protein kinase CLK1	CLK1	+	-
Q16584	Mitogen-activated protein kinase kinase kinase 11	MAP3K11	+	-
O75116	Rho-associated protein kinase 2	ROCK2	+	-
Q9BRR6	ADP-dependent glucokinase	ADPGK	-	+
P41743	Protein kinase C iota type	PRKCI	-	+
G3V4E1	Diacylglycerol kinase (Fragment)	DGKA	+	-
H0Y3Y3	ATP-dependent 6-phosphofructokinase, platelet type (Fragment)	PFKP	-	+
F2Z2Y4	Pyridoxal kinase	PDXK	-	+
O95835	Serine/threonine-protein kinase LATS1	LATS1	-	+
P48729	Casein kinase I isoform alpha	CSNK1A1	+	-
B1AXG1	Non-specific serine/threonine protein kinase	RPS6KA3	+	-
P11802	Cyclin-dependent kinase 4	CDK4	+	-
Q8N433	G protein-coupled receptor kinase	GRK3	-	+
B1AVT0	Dual-specificity protein kinase CLK2	CLK2	-	+
P53350	Serine/threonine-protein kinase PLK1	PLK1	+	+
Q07002	Cyclin-dependent kinase 18	CDK18	+	-
P10644	cAMP-dependent protein kinase type I-alpha regulatory subunit	PRKAR1A	+	-
E9PGT3	Ribosomal protein S6 kinase	RPS6KA1	+	-
H3BP85	Serine/threonine-protein kinase ULK3	ULK3	-	+
A0A0U1RQV4	Rho-associated protein kinase 1	ROCK1	-	+
K7ERP6	cAMP-dependent protein kinase catalytic subunit alpha (Fragment)	PRKACA	+	-
Q9H0K1	Serine/threonine-protein kinase SIK2	SIK2	-	+
A0A087WVK0	Dual-specificity protein kinase CLK1	CLK1	-	+
Q6P5Z2	Serine/threonine-protein kinase N3	PKN3	-	+
H0YDJ3	Serine/threonine-protein kinase PRP4 homolog (Fragment)	PRPF4B	+	-
B4E0K5	Mitogen-activated protein kinase	MAPK14	+	-
D4Q8H0	Mitogen-activated protein kinase kinase kinase MLT	pk	+	-
B4DPS1	cDNA FLJ60771, highly similar to Serine/threonine-protein kinase 16 (EC 2.7.11.1)	STK16	-	+
F8W9W2	G protein-coupled receptor kinase	GRK6	-	+
D6RCP9	Deoxycytidine kinase	DCK	+	-
P48426	Phosphatidylinositol 5-phosphate 4-kinase type-2 alpha	PIP4K2A	+	-
A0A087WVC4	cAMP-dependent protein kinase catalytic subunit beta	PRKACB	+	-
Q9UIG0	Tyrosine-protein kinase BAZ1B	BAZ1B	+	-

3. Supplementary Figures

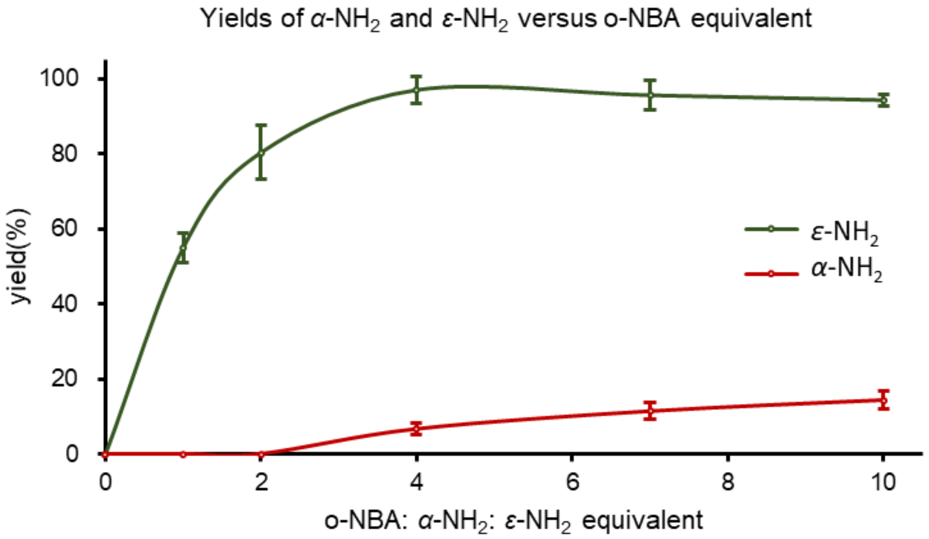

Supplementary Fig. 1 | o-NBA amide **1** reaction with **NH_2-Lys (Cbz)-OMe** (*α*-NH_2) or **Cbz-Lys-OMe (*ε*-NH_2).** Data are shown as mean ± SEM (n=3 independent experiments).

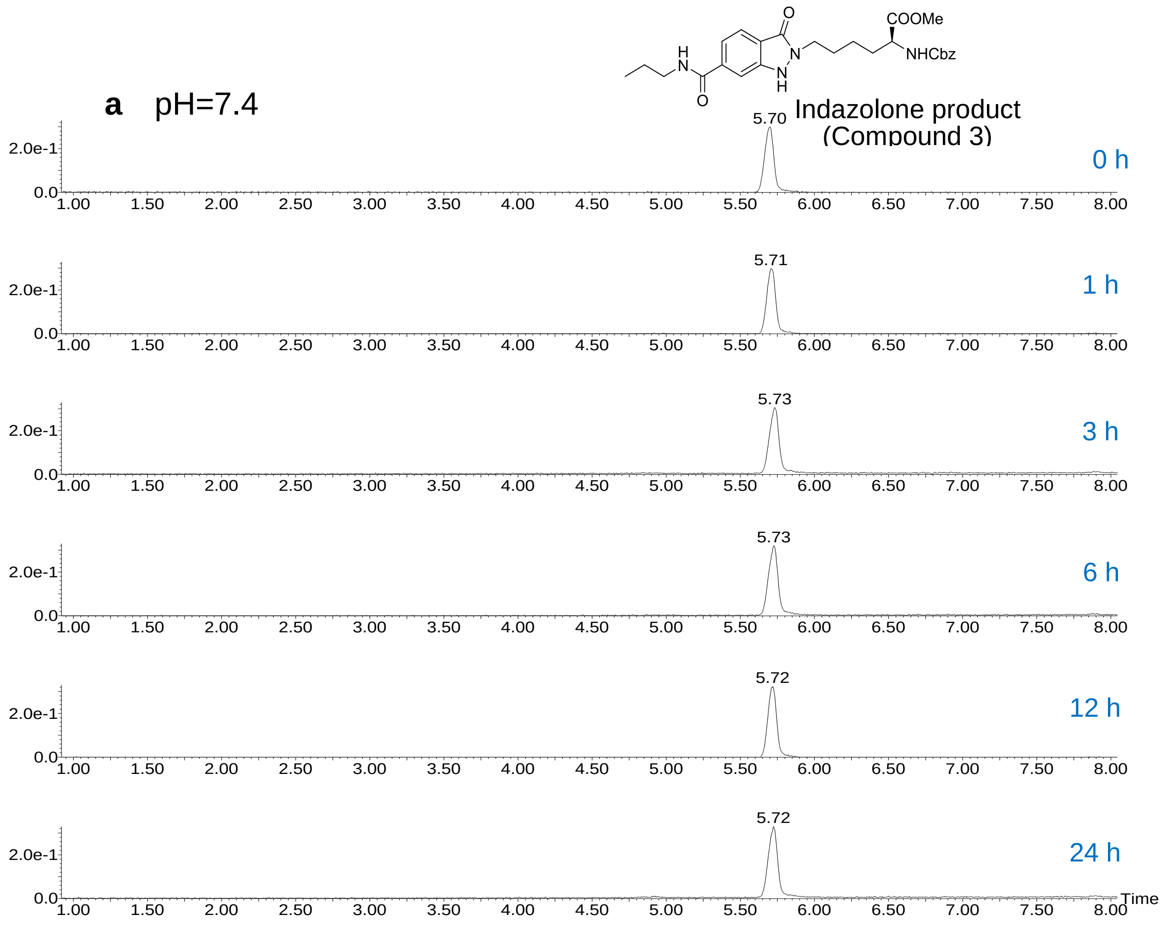

b pH= 6.0

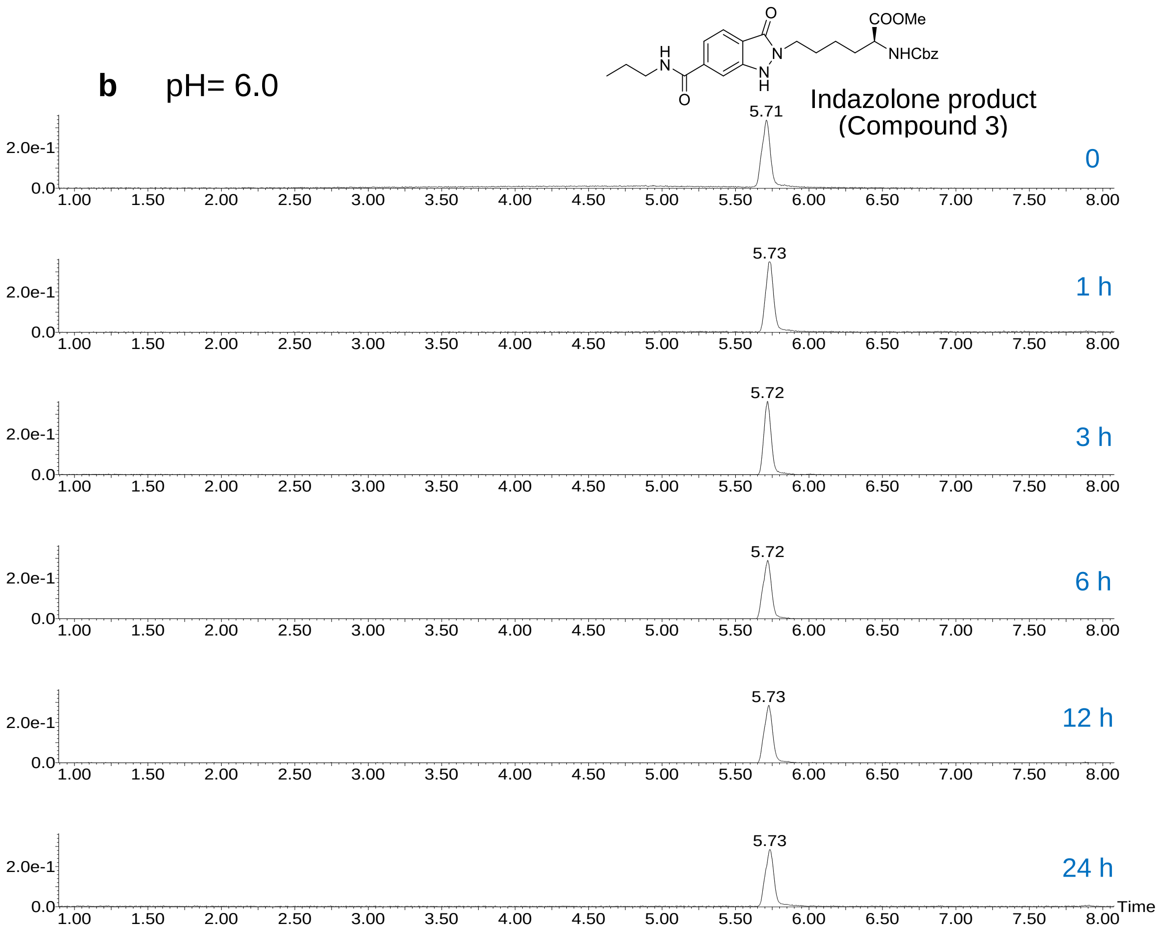

Indazolone product
(Compound 3)
COOMe
NHCbz
5.71
0
5.73
1 h
5.72
3 h
5.72
6 h
5.73
12 h
5.73
24 h
2.0e-1
0.0
1.00
1.50
2.00
2.50
3.00
3.50
4.00
4.50
5.00
5.50
6.00
6.50
7.00
7.50
8.00
Time

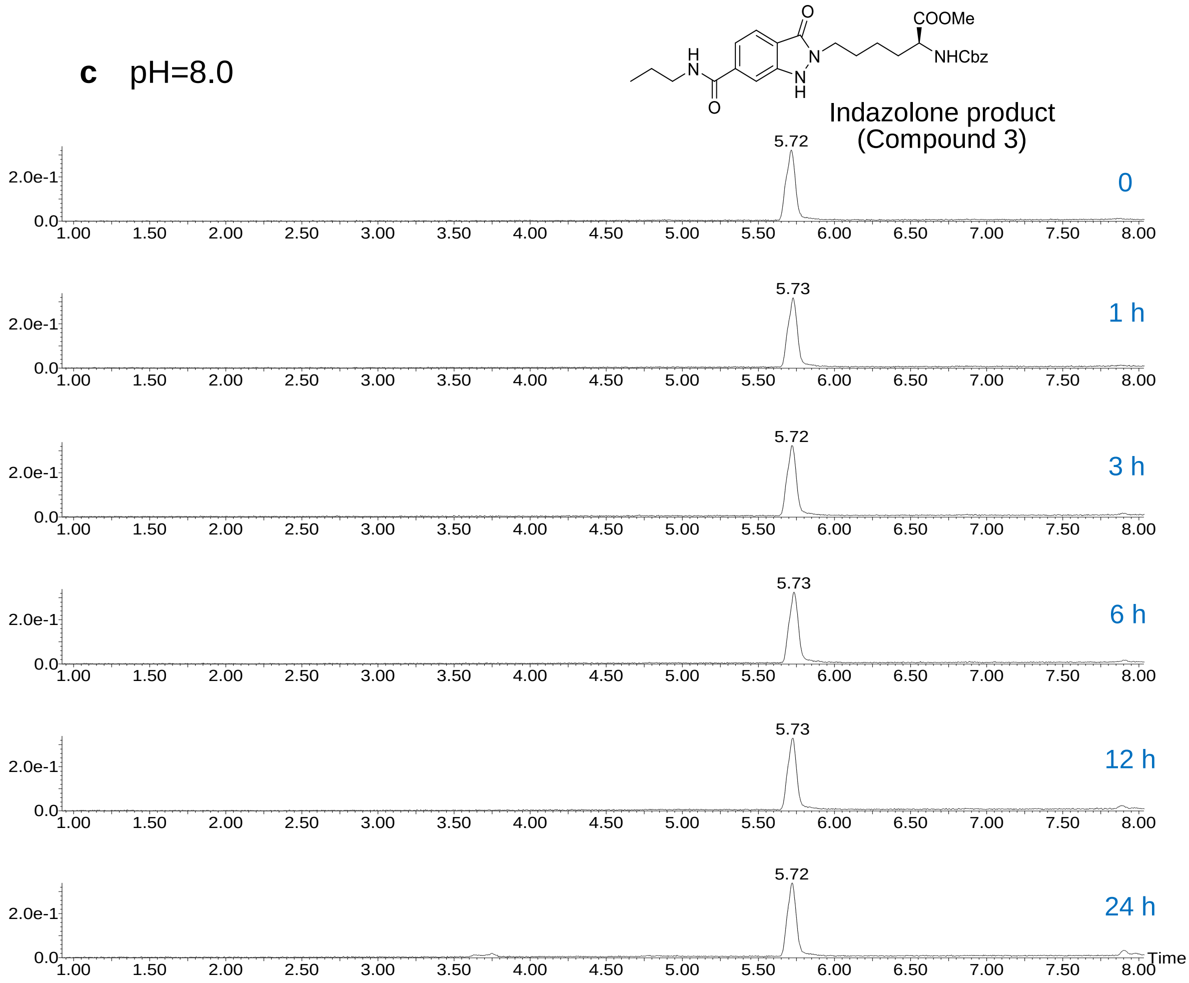

Supplementary Fig. 2 | Stability of indazolone product (compound 3) in pH = 7.4 **(a)** /6.0 **(b)** /8.0 **(c)** 100 mM PBS/MeOH (2:1).

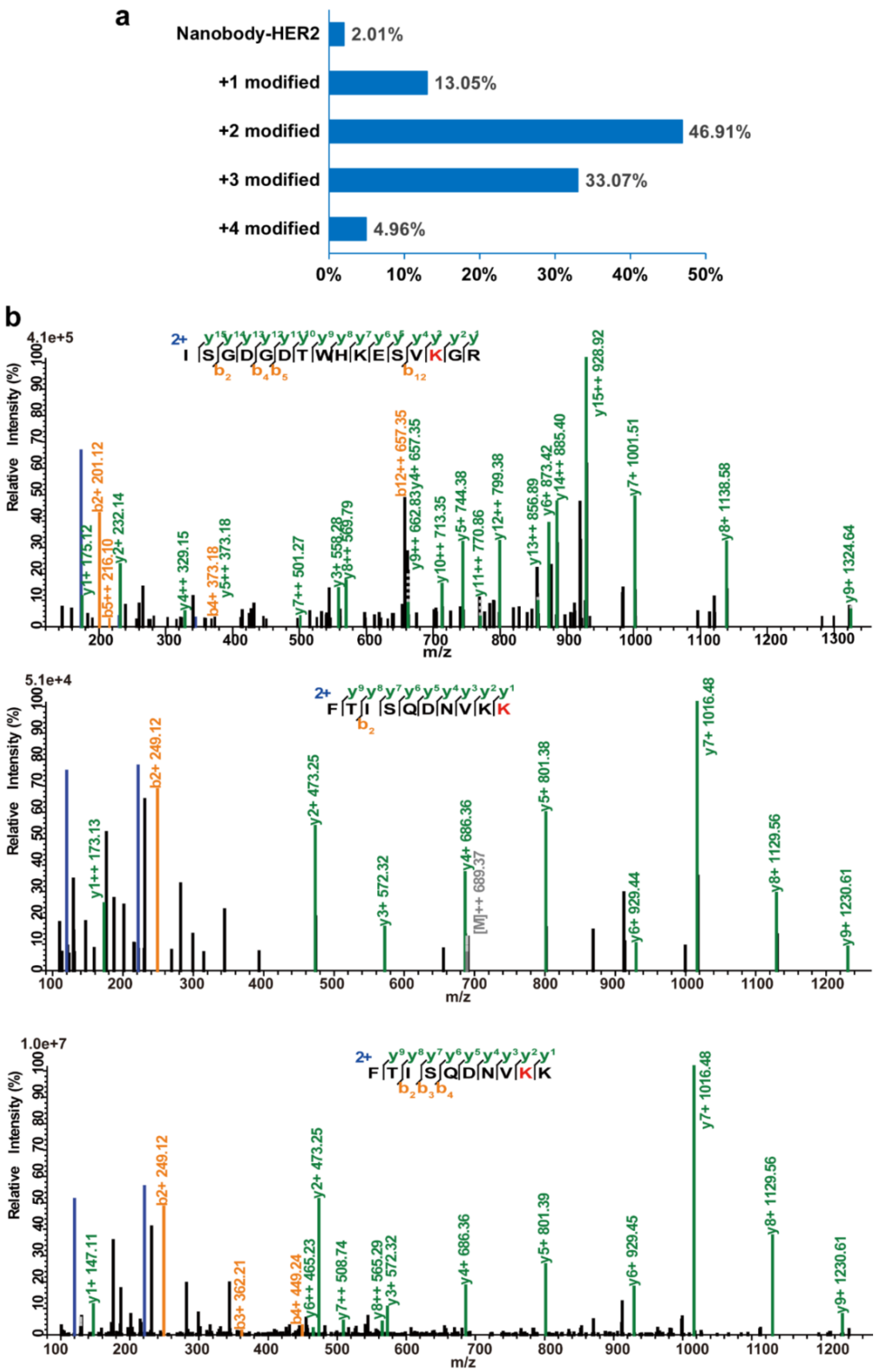

Supplementary Fig. 3 | a) Quantitative analysis of Nanobody-HER2 (50 µM) after o-NBA-alkyne (125 µM) labeling by peak height of mass spectrometry; b) Trypsin digest and subsequent ESI-MS/MS analysis of the fragments of Nanobody-HER2 conjugate confirmed the lysine-specific labeling.

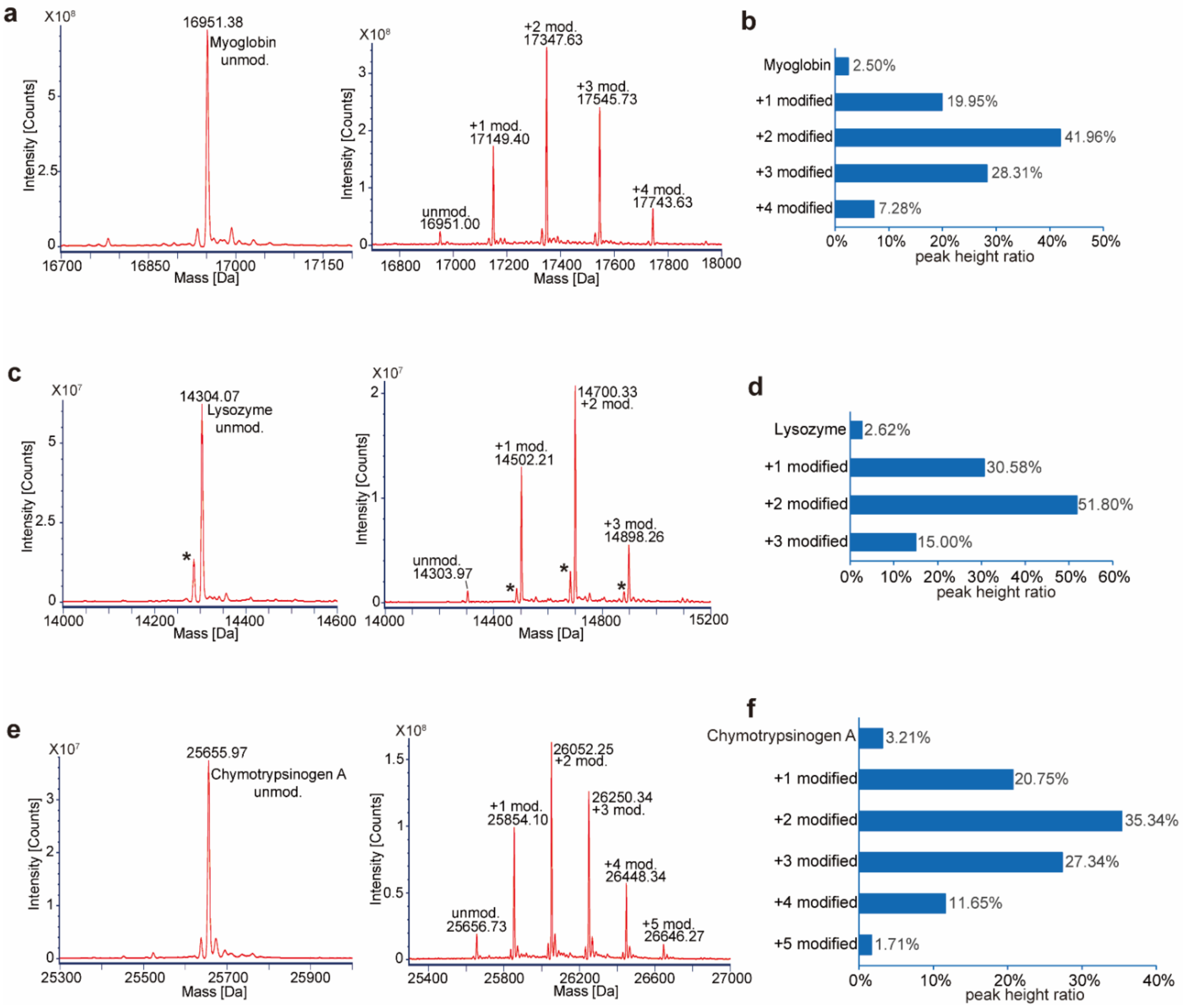

Supplementary Fig. 4 | a) ESI-TOF Spectrum of unmodified Myoglobin and o-NBA-alkyne (125 μM) modified Myoglobin (50 μM); b) Quantitative analysis of Myoglobin (50 μM) after o-NBA-alkyne (125 μM) labeling by peak height of mass spectrometry; c) ESI-TOF Spectrum of Lysozyme and o-NBA-alkyne (125 μM) modified Lysozyme (50 μM) (Asterisks represent impure proteins); d) Quantitative analysis of Lysozyme (50 μM) after o-NBA-alkyne (125 μM) labeling by peak height of mass spectrometry; e) ESI-TOF Spectrum of Chymotrypsinogen A and o-NBA-alkyne (125 μM) modified Chymotrypsinogen A (25 μM); f) Quantitative analysis of Chymotrypsinogen A (25 μM) after o-NBA-alkyne (125 μM) labeling by peak height of mass spectrometry.

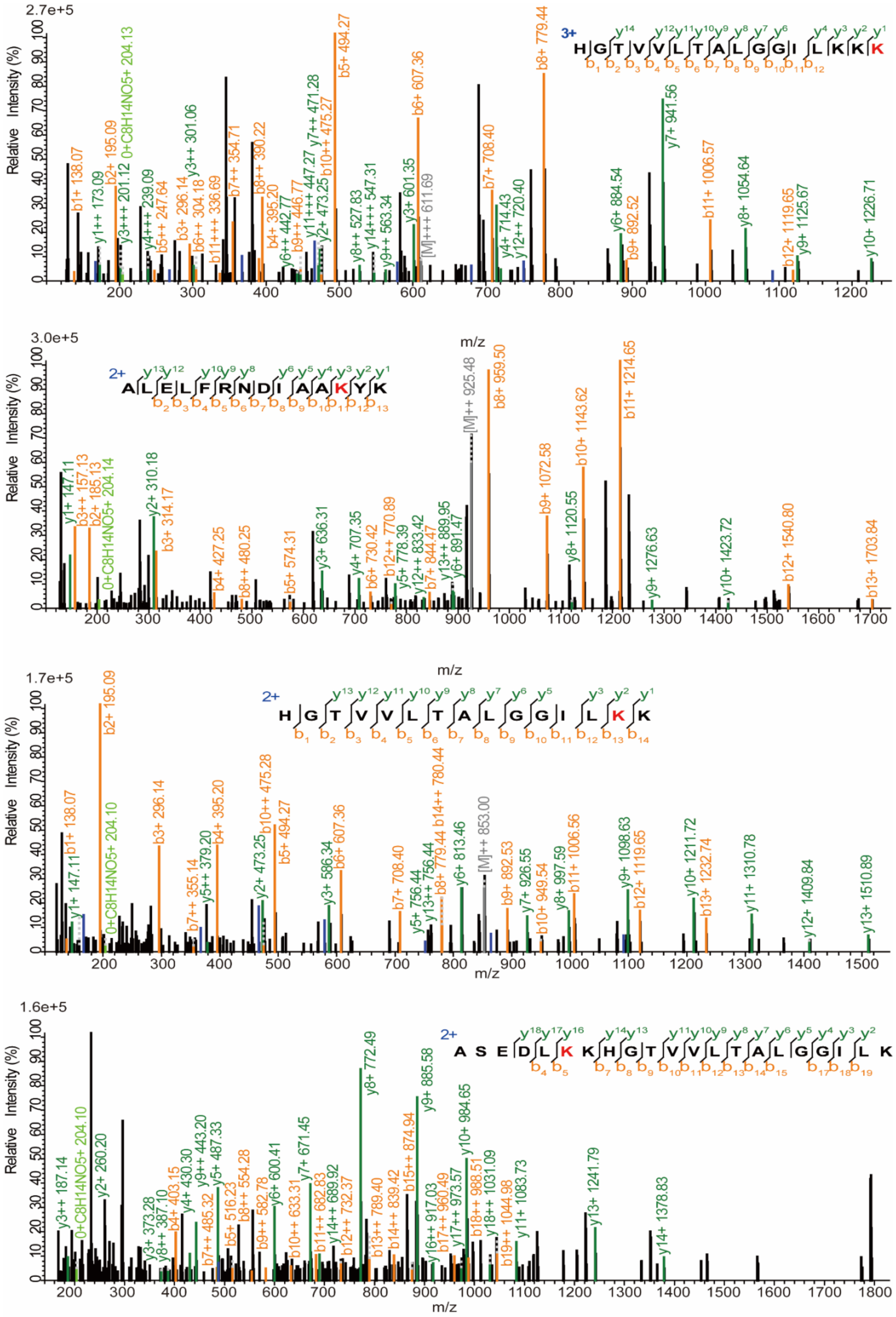

Supplementary Fig. 5 | Trypsin digest and subsequent ESI-MS/MS analysis of the fragments of Myoglobin and o-NBA-alkyne conjugate confirmed the lysine-specific labeling.

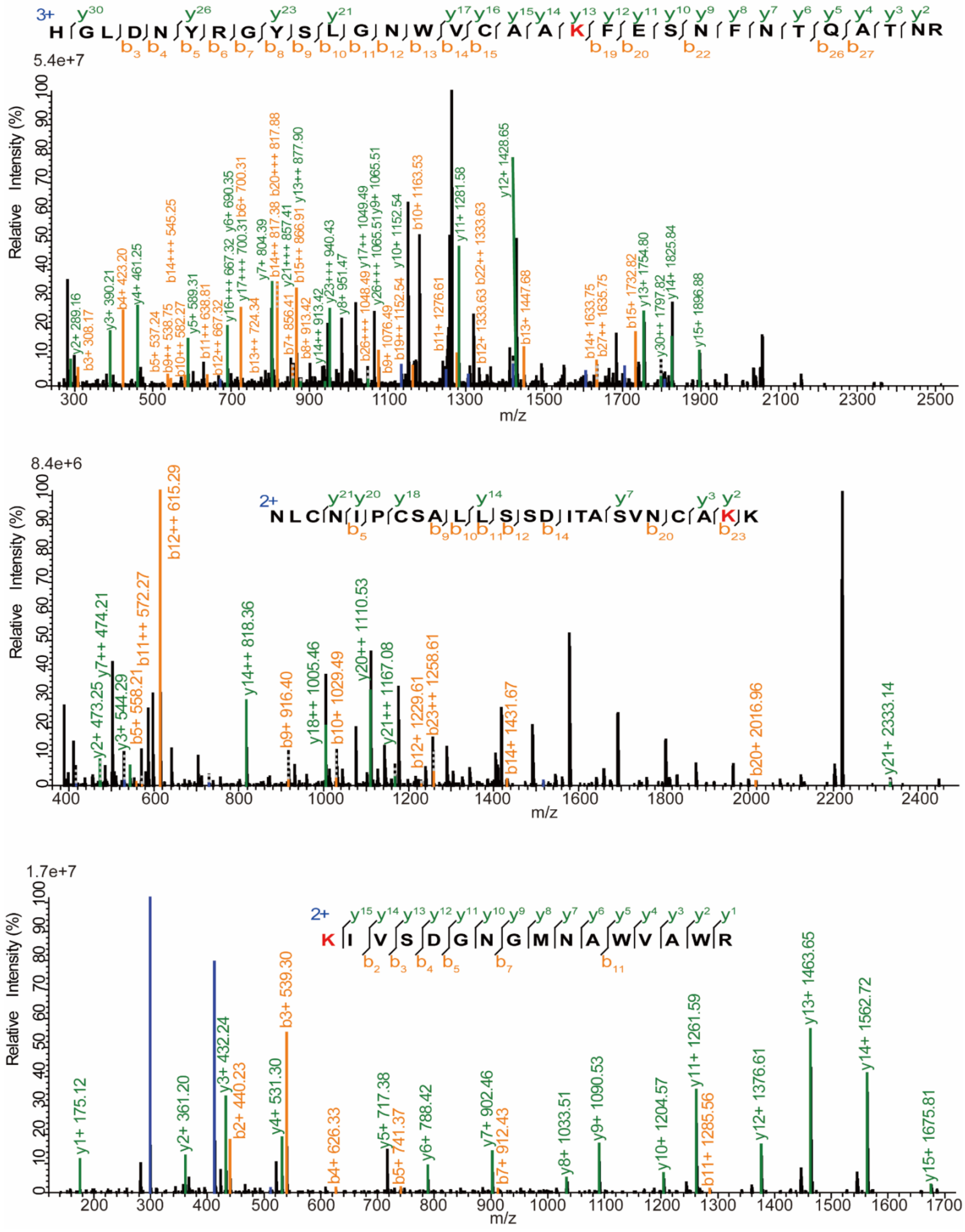

Supplementary Fig. 6 | Trypsin digest and subsequent ESI-MS/MS analysis of the fragments of Lysozyme and o-NBA-alkyne conjugate confirmed the lysine-specific labeling.

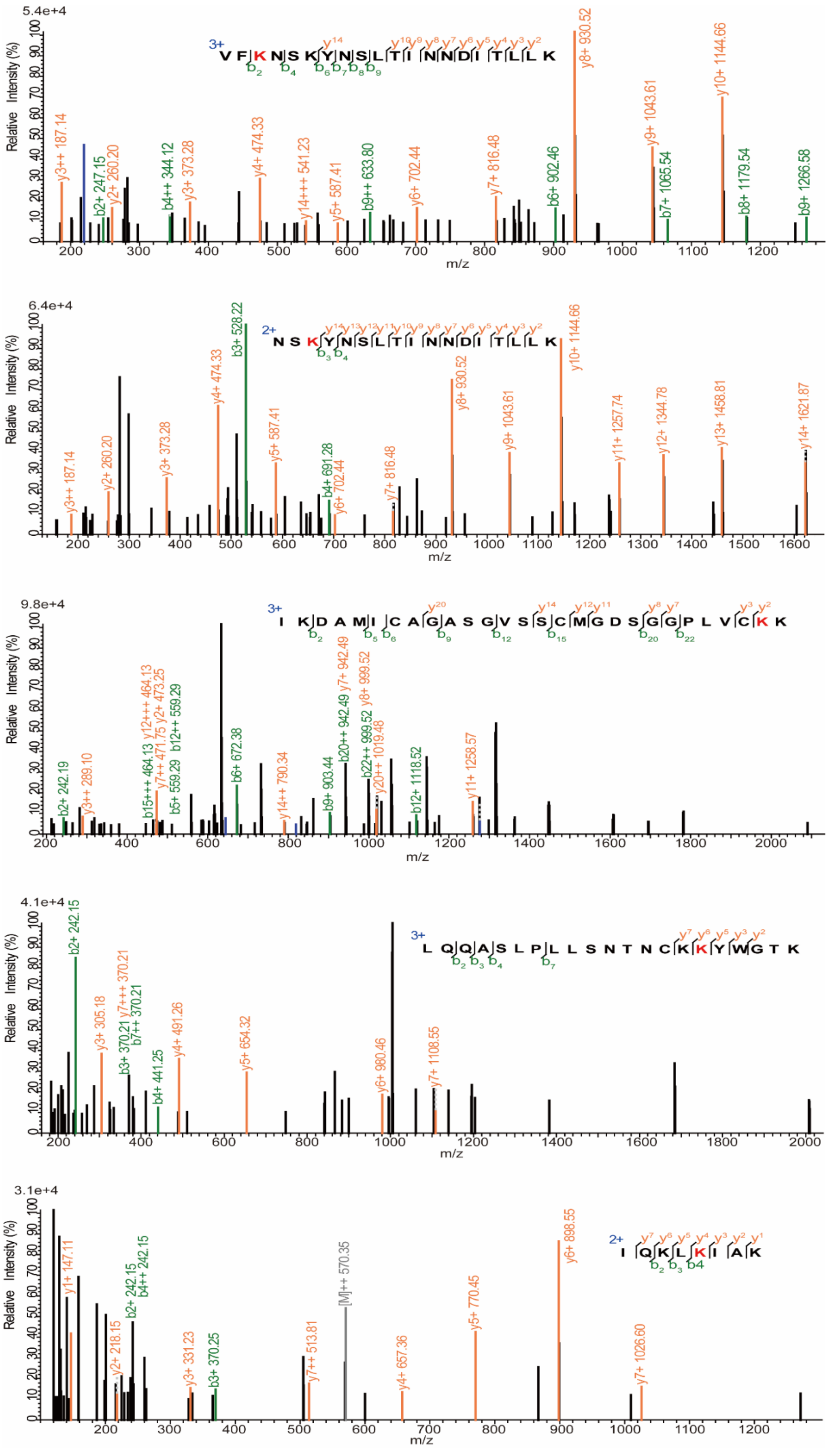

Supplementary Fig. 7 | Trypsin digest and subsequent ESI-MS/MS analysis of the fragments of Chymotrypsinogen A and o-NBA-alkyne conjugate confirmed the lysine-specific labeling.

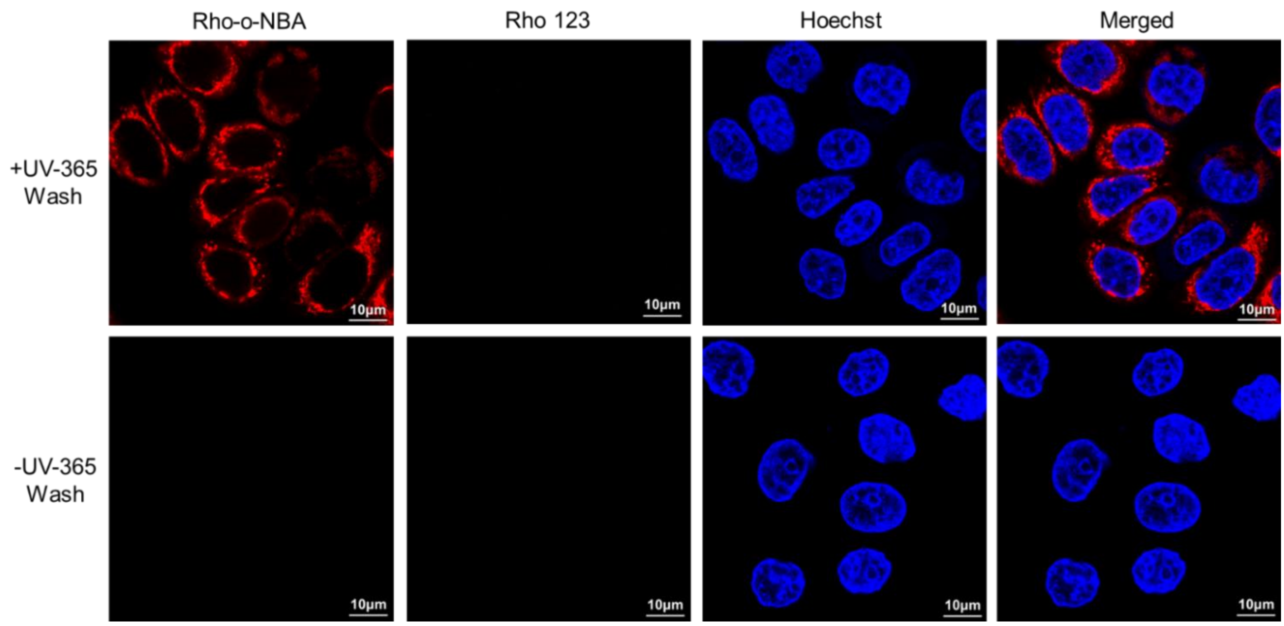

Supplementary Fig. 8 | Fixed cell imaging to validate the covalent bond was formed with Rho-o-NBA probe and mitochondria organelle, upon light activation (+UV-365 nm). Confocal images of MDA-MB-486 cells stained with Rho-o-NBA (Red channel), Rhodamine 123 (Green channel) and Hoechst 33342 (Blue channel) with (top panel) or without (bottom panel) 365nm UV light irradiation and then fixed by 4% PFA and washed to remove unreacted Rho 123 for intended comparison. Signal expansion was observed compared to live cell imaging (Figure. 6e, top panel, red color), which probably resulted from the fixation and washing procedures in the experiment, see Supplementary Fig. 19-20 for details.

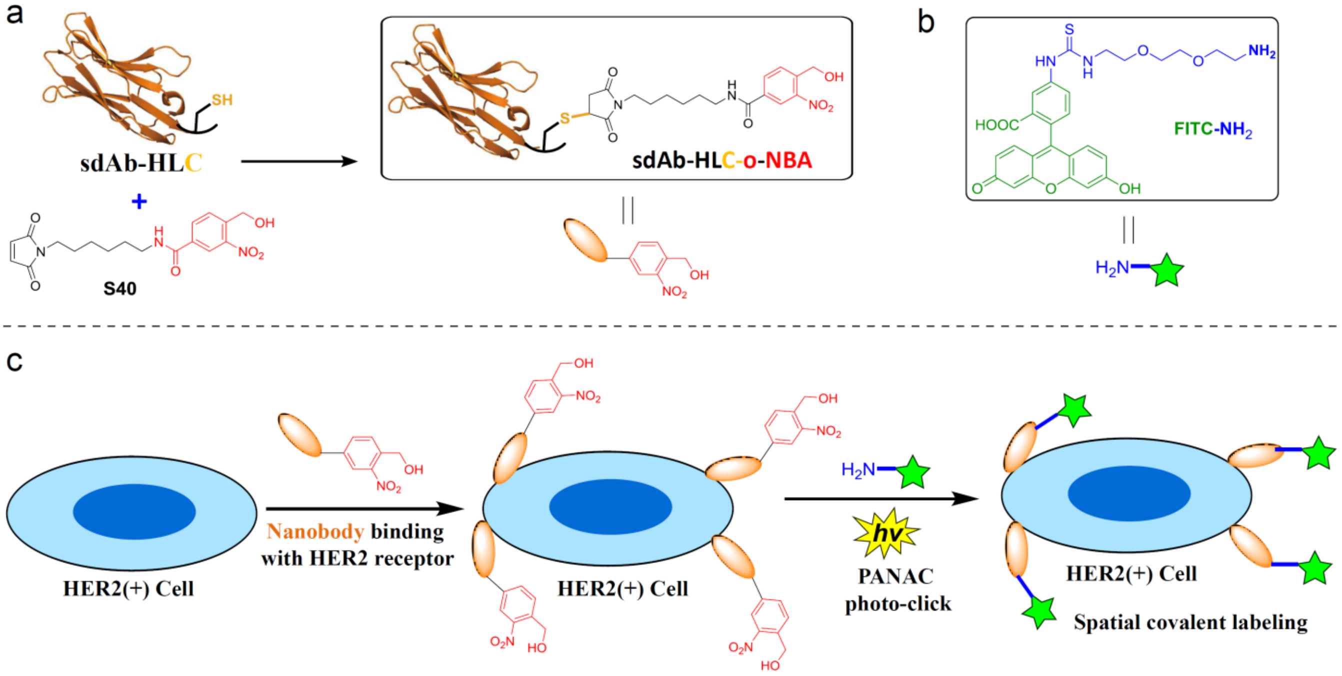

Supplementary Fig. 9 | Schematic illustration of spatial labeling of live cells by PANAC photo-click reaction. **a**, Introducing of the o-NBA moiety (compound **S40**) to the HER2 specific nanobody[1] (**sdAb-HLC**) at C-terminal to produce (**sdAb-HLC-o-NBA**). **b**, The structure of the FITC-NH_2 compound. **c**, The HER2 specific nanobody (**sdAb-HLC-o-NBA**) labeled with **FITC-NH_2** via PANAC photo-click reaction on the surface of HER2 positive cell SK-Br-3.

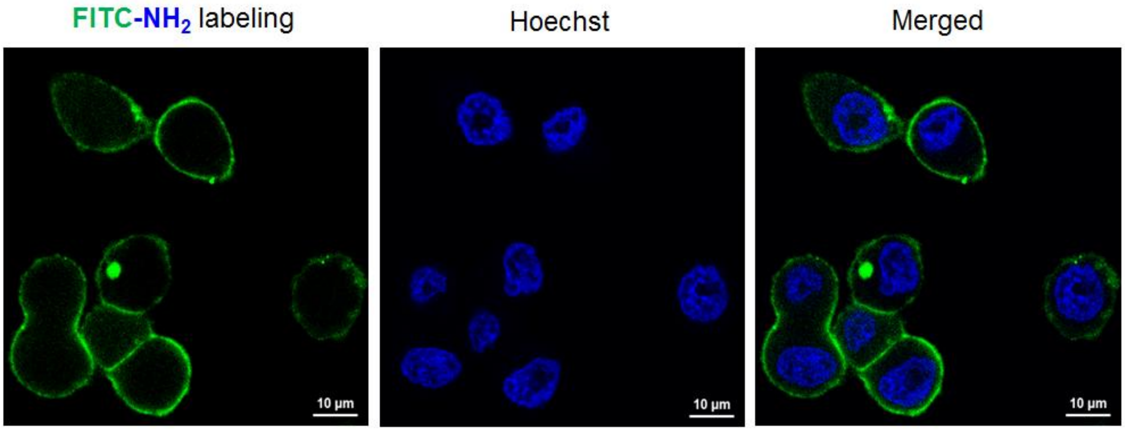

Supplementary Fig. 10 | SK-Br-3 Cells were incubated with HER2 specific nanobody (**sdAb-HLC-o-NBA**) and Hoechst 33324 (blue) at 37 ℃ for 1 h and 15 min respectively, washed with PBS 3 times, then incubated with **FITC-NH$_2$** (green) and exposed to 365 nm UV light irradiation for 20 min followed by PBS wash 3 times. Cell imaging using confocal fluorescence. Scale bar: 10 μm.

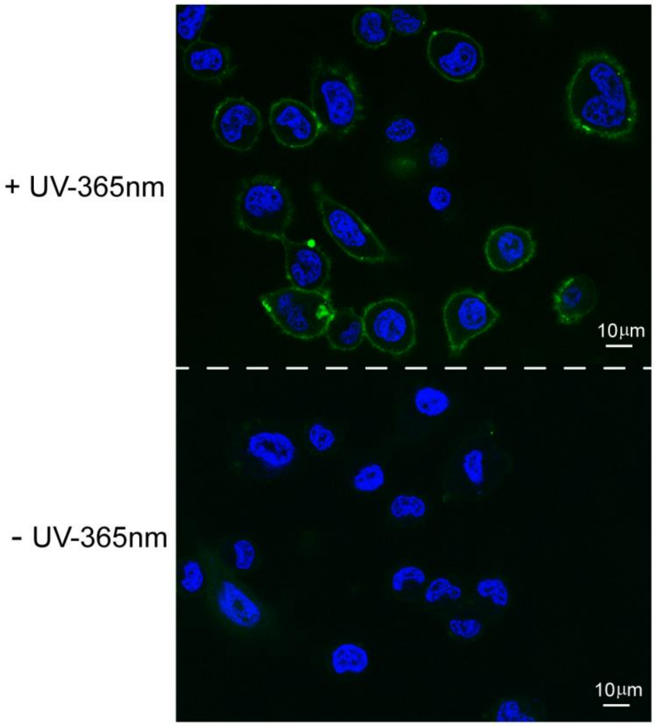

Supplementary Fig. 11 | Labeling of HER2 specific nanobody (**sdAb-HLC-o-NBA**) with FITC-NH$_2$ on the surface of SK-Br-3 cells via light-induced PANAC reaction. SK-Br-3 Cells were incubated with HER2 specific nanobody (**sdAb-HLC-o-NBA**) and Hoechst 33324 (blue) at 37 ℃ for 1 h and 15 min respectively, washed with PBS 3 times. Labeling with spatial control was demonstrated simply by shielding part of the cells from UV-365 nm light irradiation (bottom panel), or with light-activation for 10 min (top panel). After labeling, cells were washed by PBS wash for 3 times. And cell imaging using confocal fluorescence. Scale bar: 10 μm.

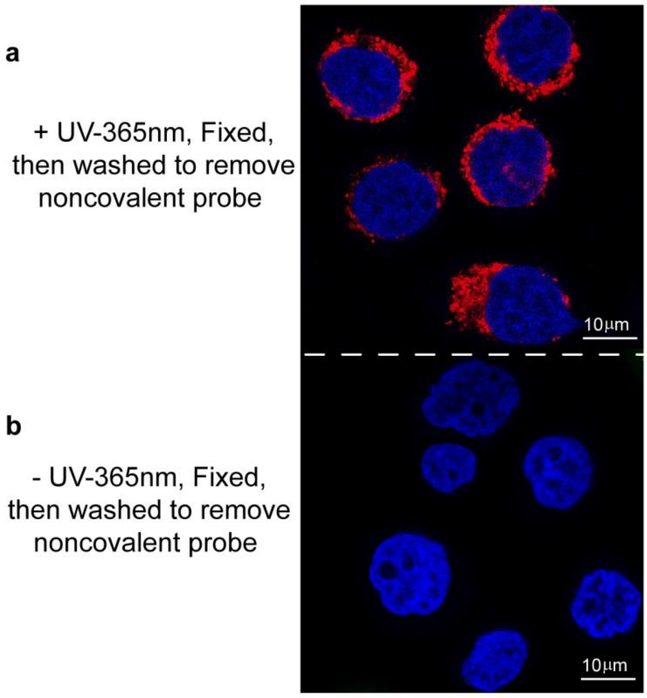

Supplementary Fig. 12 | Confocal images of MDA-MB-486 cells stained with Rho-o-NBA probe (Red channel) and Hoechst 33342 (Blue channel) in different conditions: Rho-o-NBA probe with light irradiation (a), or shielding part of the cells from UV-365 nm light irradiation (b), then fixed with 4% PFA and washed to remove unreacted probe for intended comparison.

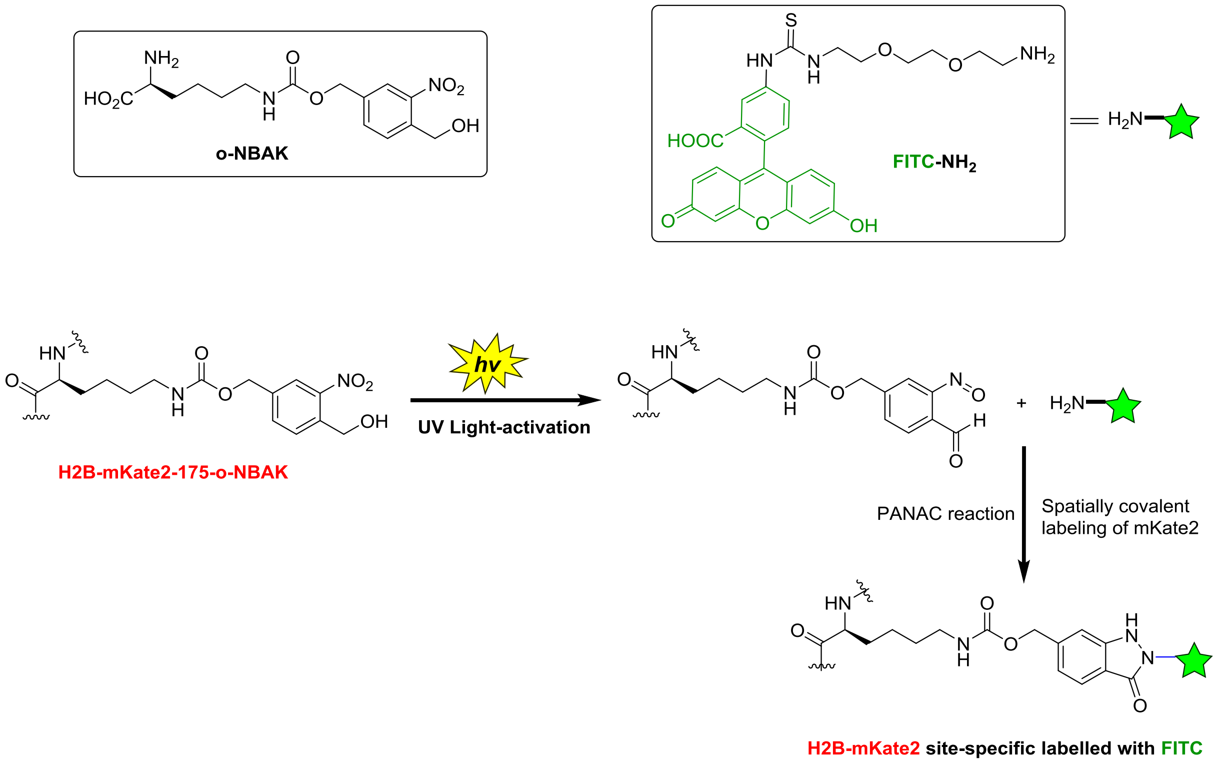

Supplementary Fig. 13 | Schematic illustration of site-specific labeling of H2B-mKate2-175-o-NBAK protein which localizes at the nucleolus, via PANAC photo-click reaction with temporal and spatial control.

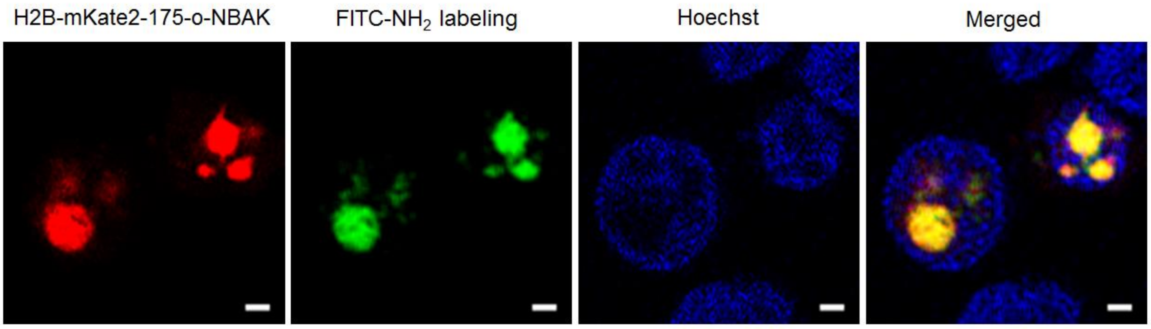

Supplementary Fig. 14 | Unnatural amino acid o-NBAK was added to the growth medium 1h prior transfection at a final concentration of 1 mM. HEK293T cells were transfected with pcDNA3.1-H2B-mKate2 K175TAG HEK293T (red channel) and pNEU-hMbPylRS-4xU6M15 plasmids[2]. After cultured for 24 h, HEK293T cells were fixed, stained with Hoechest 33342 (2 μg/mL, blue channel) for 15 min, incubated with FITC-NH_2 (20 μM, green channel) for 15 min and exposed to 365 nm for 20 min, then washed to remove unreacted probe. Scale bar: 2 μm.

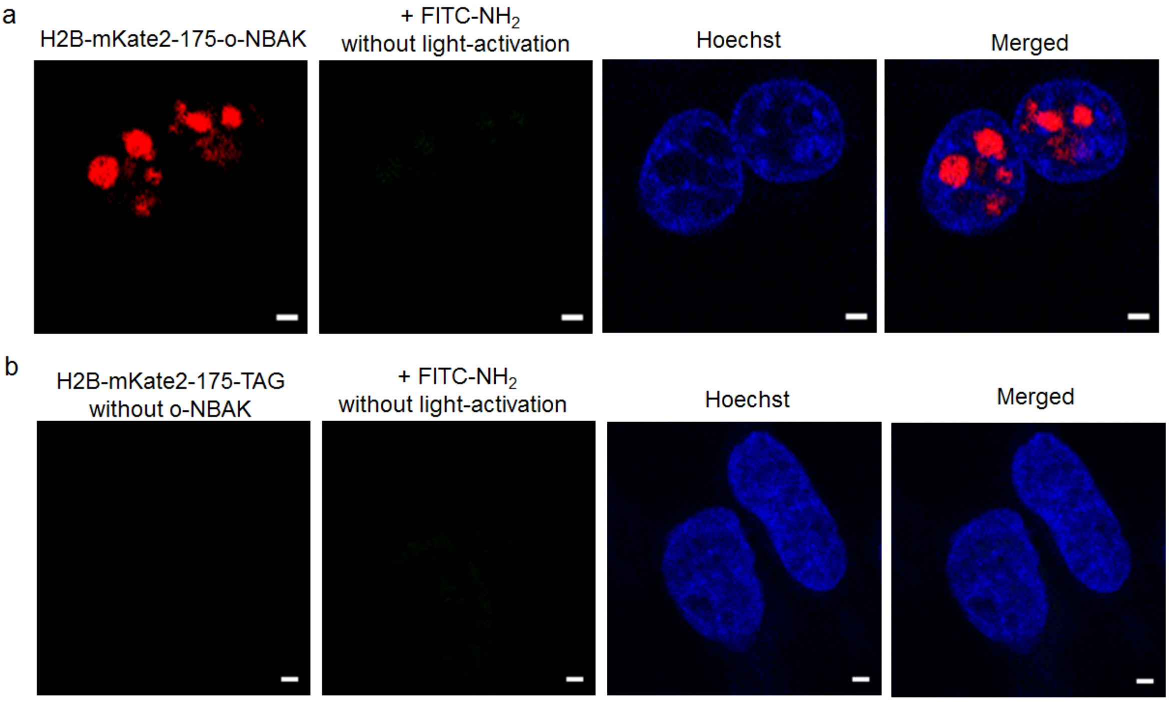

Supplementary Fig. 15 | a, with unnatural amino acid o-NBAK was added to the growth medium 1h prior transfection at a final concentration of 1 mM. **b**, without unnatural amino acid o-NBAK. HEK293T cells were transfected with pcDNA3.1-H2B-mKate2 K175TAG HEK293T (red channel) and pNEU-hMbPylRS-4xU6M15 plasmids. After cultured for 24 h, HEK293T cells were fixed, stained with Hoechest 33342 (2 μg/mL, blue channel) for 15 min, incubated with FITC-NH_2 (20 μM, green channel) for 15 min without light-activation, then washed to remove unreacted probe. Scale bar: 2 μm.

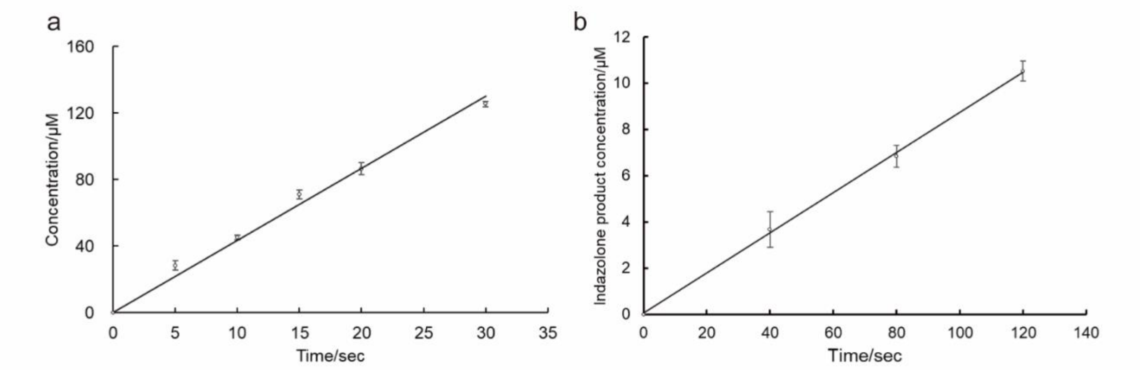

c

Compound	o-NBA amide 1	Ferrioxalate-polyoxometalate
ε_{365} (M^{-1} cm^{-1})	850	A_r = 2.46
k_0 (μM/s)	0.087	4.34
Φ	0.52	0.18

Supplementary Fig. 16 | Determining o-NBA amide 1 under 365 nm excitation to generate o-nitroso benzaldehyde quantum yields using a ferrioxalate-polyoxometalate-based chemical actinometer[3,4]. (a) Photoinduced formation of POM^- over a period of 30 sec. Data are shown as mean ± SEM (n=3 independent experiments). (b) Reaction of o-NBA amide 1 (20 μM) and methylamine (200 μM) over 120 s by UPLC-MS. The reaction is a zero-order reaction with light as a decisive step. Data are shown as mean ± SEM (n=3 independent experiments). (c) Photo-physical properties of o-NBA amide 1 with Ferrioxalate-polyoxometalate-based chemical actinometer as a reference. The absorbance (A_r) of ferrioxalate-polyoxometalate at 365 nm was 2.46 determined by UV-Vis spectrophotometer. The quantum yield (**Φ_n**) of o-NBA amide 1 under 365 nm excitation to generate o-nitroso benzaldehyde was calculated using the following equation: $\mathbf{\Phi_n} = (A_r/A_n)\ (\mathbf{k_n}/\mathbf{k_r})\ \mathbf{\Phi_r} = (\varepsilon_r c_r/\varepsilon_n c_n)\ (\mathbf{k_n}/\mathbf{k_r})\ \mathbf{\Phi_r}$.

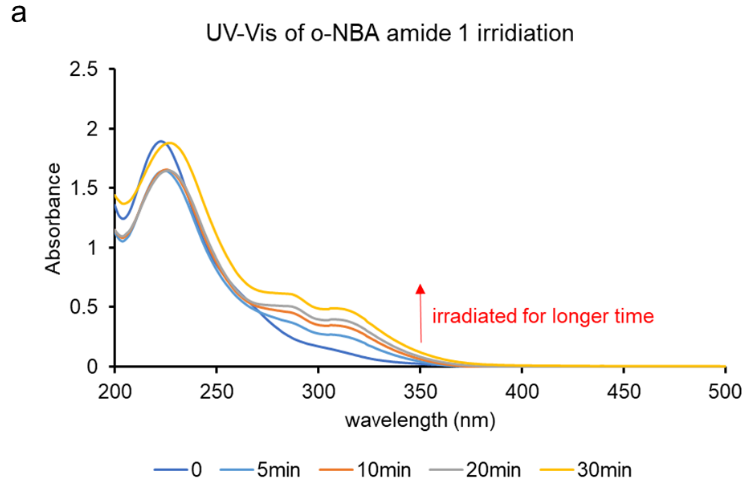

b

	λ_{max} (nm)	ε_{max} (M^{-1} cm^{-1})	ε_{365} (M^{-1} cm^{-1})	k_0 (μM/s)	Φ
**o-NBA amide 1**	224	18700	850	0.087	0.52

Supplementary Fig. 17 | (a) UV-Vis spectrum of o-NBA amide 1 (20 μM) in 10 mM PBS/MeOH (1:1, pH = 7.4) irradiated for 0/5 min/10 min/20 min/30 min. (b) photo-physical properties of o-NBA amide 1.

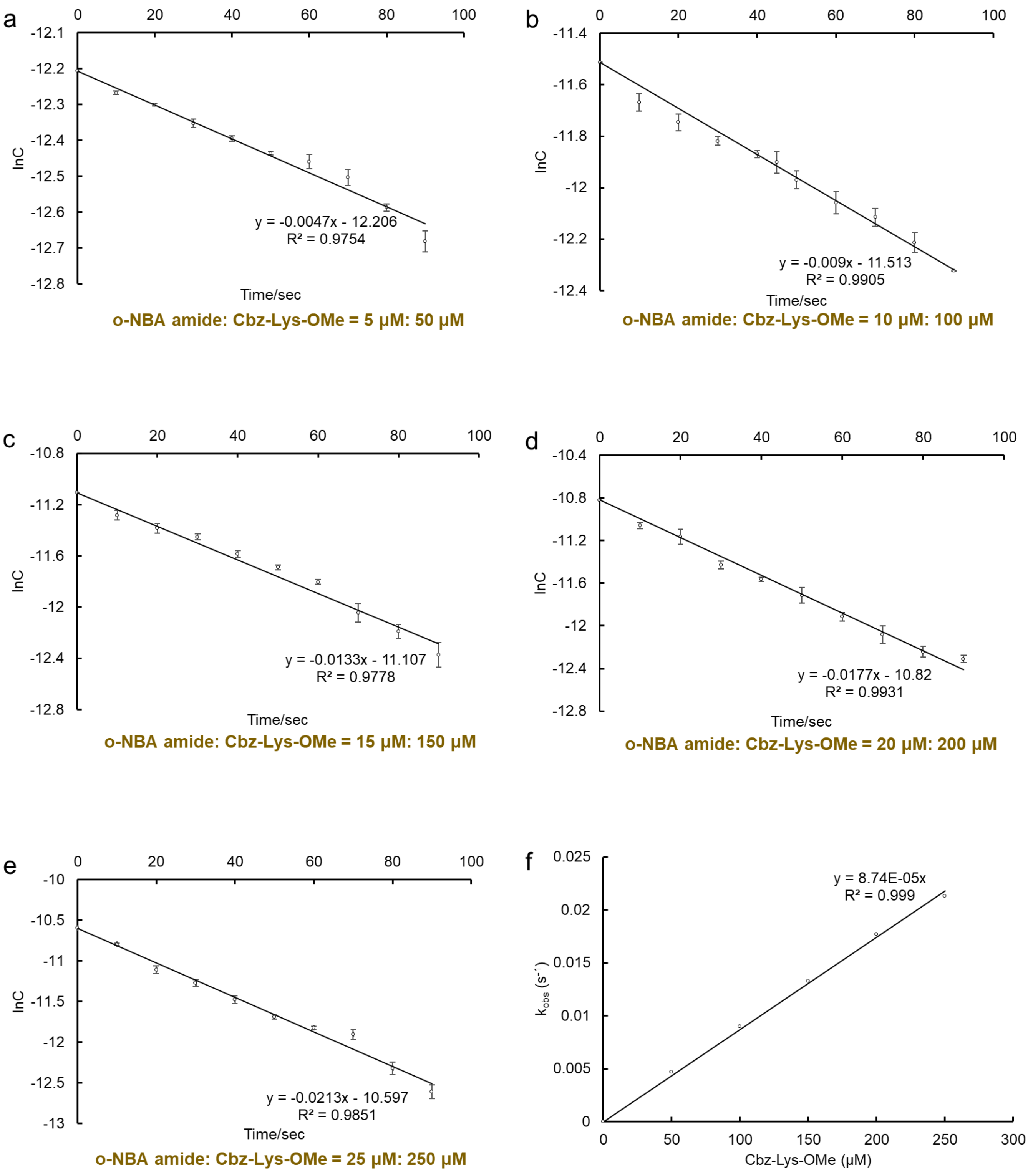

Supplementary Fig. 18 | Reaction curve of ln([o-NBA amide 1]) over 90 s with 50 μM (a)/ 100 μM (b)/ 150 μM (c)/ 200 μM (d)/ 250 μM (e) Cbz-Lys-OMe (10 equivalent) and curve of k_{obs} versus Cbz-Lys-OMe (f). o-NBA amide 1 (5/10/15/20/25 μM) and Cbz-Lys-OMe (50/100/150/200/250 μM) was irradiated under 365 nm in PBS/MeOH. The pseudo-first order reaction rate constant of o-NBA amide 1 (k_{obs}) was analyzed by ln([o-NBA amide 1])-t line. The second-order reaction rate constant $\mathbf{k_2}$ = k_{obs}/[Cbz-Lys-OMe] = 87.4 M^{-1} s^{-1}. Data are shown as mean ± SEM (n=3 independent experiments).

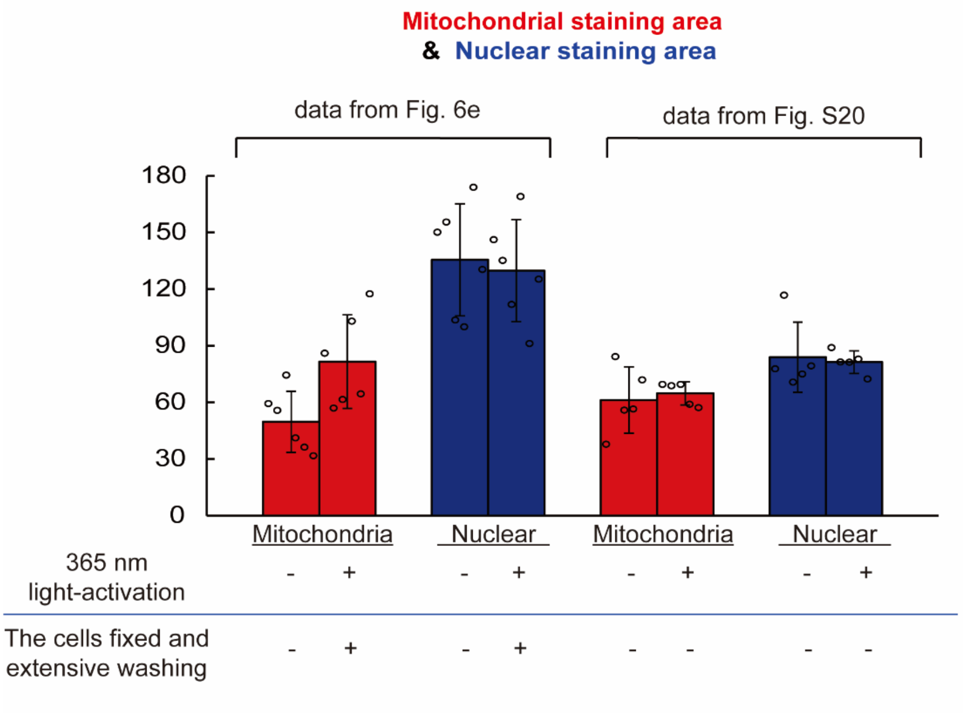

Supplementary Fig. 19 | Mitochondrial staining area and Nuclear staining Area of irradiated and unirradiated MDA-MB-468 cells (live cells, or fixed cells from Fig. 6e). The area of Mitochondrial and Nuclear was calculated by ImageJ software, respectively, each calculation ≥ 5 randomly picked cells. Data are shown as mean ± SEM (n ≥5. For data from Fig. 6e, n=6 cells in the same field of view; for data from Fig. S20, n=5 cells in the same field of view).

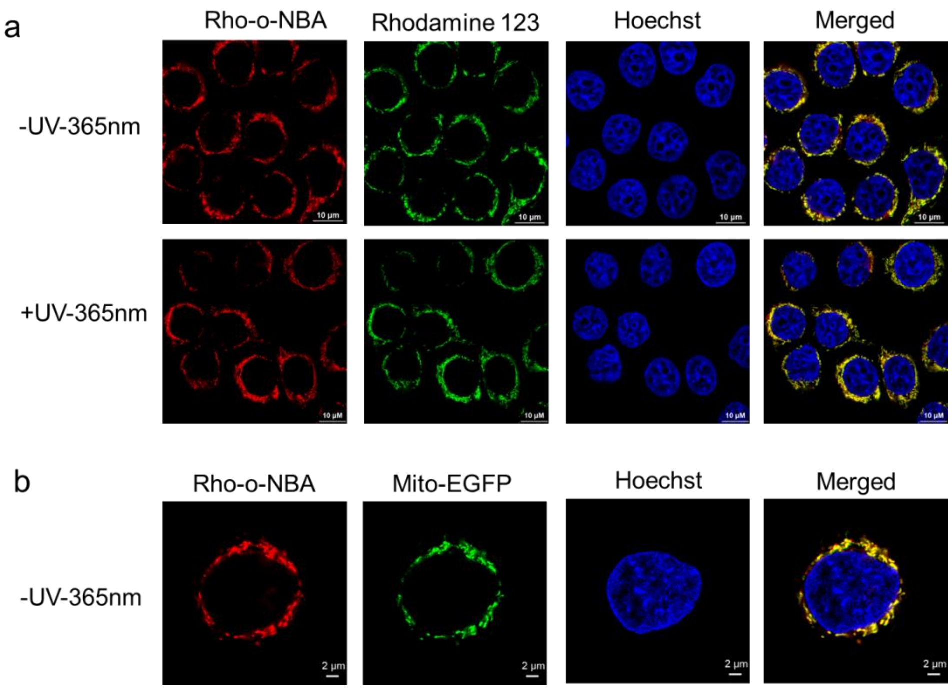

Supplementary Fig. 20 | Live cell imaging, in all of these experiments, no fixation and no washing procedure was employed. **a**, Confocal images of MDA-MB-486 cells stained with Rho-o-NBA (Red channel), Rhodamine 123 (Green channel) and Hoechst 33342 (Blue channel) without (a, top panel) or with (a, bottom panel) 365nm UV light irradiation. **b**, Co-localization of Rho-o-NBA probe (Red channel) and Mito-EGFP reporter (Green channel), and with Hoechst 33342 staining (Blue channel), without light activation.

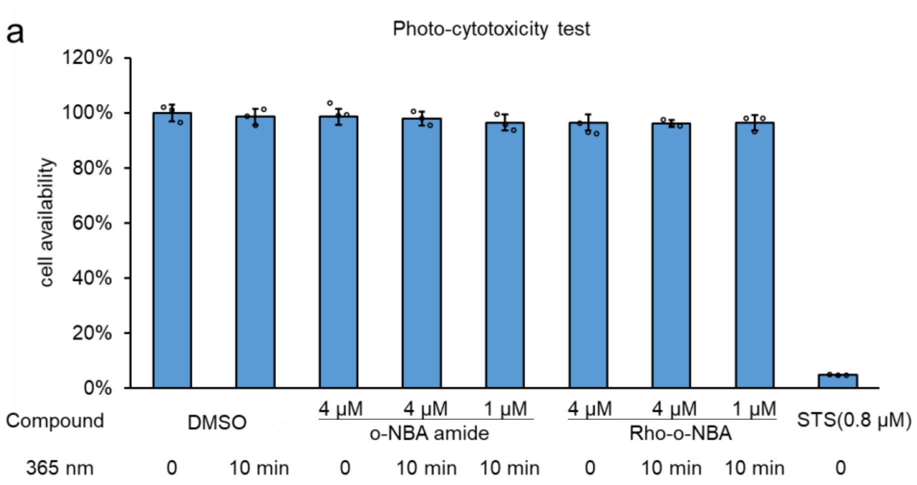

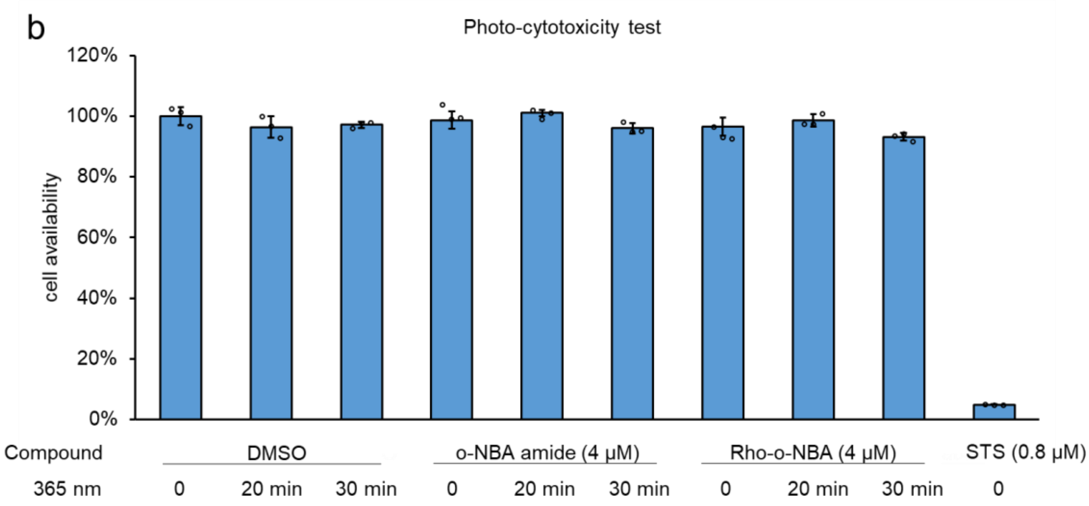

Supplementary Fig. 21 | Photo-toxicity on cell viability MDA-MB-468 cells with short time 10min (**a**) and longer time (**b**). MDA-MB-468 cells were incubated with o-NBA amide 1 (1 or 4 μM)/ Rho-o-NBA (1 or 4 μM)/ DMSO/ STS (0.8 μM) for 30 min and were irradiated with 365 nm UV light for (10, 20 or 30) min or without UV. After incubation for further 2 days, cell availability was analyzed by CCK8 kit. Staurosporine (STS)-induced apoptosis and cell viability as control. Data are shown as mean ± SEM (n=3 biologically independent samples).

4. Experimental Procedures and UPLC-MS Analysis of Peptides

4.1 The conditions for light-induced o-NBA amide 1 reacts with Cbz-Lys-OMe

Stock solution: o-NBA amide 1 (10 mM) in MeOH/DMSO/ACN and Cbz-Lys-OMe (10 mM) in MeOH/DMSO/ACN.

Method: Irradiated in a mixture: o-NBA amide 1 (2 mM) and Cbz-Lys-OMe (0.5 mM) in corresponding buffer were treated with 365 nm UV light for 7 min and shaken at 25 ^{o}C for 30 min. The samples were collected, diluted with MeOH/H_2O and analyzed by UPLC-MS.

Mix after o-NBA irradiated, One portion: o-NBA amide 1 (2 mM) was treated with 365 nm UV light for 7 min and then was added to Cbz-Lys-OMe (0.5 mM) in corresponding buffer immediately. The mixture was shaken at 25 ^{o}C for 30 min and then were collected, diluted with MeOH/H_2O and analyzed by UPLC-MS.

Mix after o-NBA irradiated, 3 or 5 aliquots: o-NBA amide 1 (2 mM) was treated with 365 nm UV light for 7 min and then was added to Cbz-Lys-OMe (0.5 mM) in corresponding buffer 3 or 5 aliquots with a 10 min- or 6 min-interval. The mixture was shaken at 25 ^{o}C for 30 min and then were collected, diluted with MeOH/H_2O and analyzed by UPLC-MS.

Low concentration: Stock solution: o-NBA amide 1 (1 mM/2 mM/4 mM/) in MeOH and Cbz-Lys-OMe (0.2 mM/0.4 mM/1 mM/2 mM) in MeOH. o-NBA amide 1 (0.2 mM/0.4 mM/0.8 mM) and Cbz-Lys-OMe (0.01 mM/0.02 mM/0.05 mM/0.1 mM) in 20 mM PBS/MeOH (1:1, pH = 7.4) were treated with 365 nm UV light for 7 min and shaken at 25 ^{o}C for 30 min. The samples were collected, diluted with MeOH/H_2O and analyzed by UPLC-MS.

The method of Yields detection: yields were determined by ratio of peak area value of experiment to that of internal standard product on reverse-phase HPLC, are reported as an average of three independent trials. See UPLC-MS Analysis of Peptides Part and UPLC-MS Analysis of Reactions and Products of Small Molecules Part for HPLC trace.

4.2 Reaction yields of o-NBA amide 1 and Cbz-Lys-OMe versus irradiation time

Stock solution: o-NBA amide 1 (10 mM) in MeOH, and Cbz-Lys-OMe (10 mM) in MeOH. o-NBA amide 1 (2 mM) and Cbz-Lys-OMe (0.5 mM) in 100 mM PBS/MeOH (2:1, pH = 7.4) were treated with 365 nm UV light for 30 s/1 min/2 min/4 min/6 min/8 min/10 min and shaken at 25 ^{o}C for 0 or 30 min. The samples were collected, diluted with MeOH/H_2O and analyzed by UPLC-MS. The yield was quantified by the standard curve of the product.

4.3 Half-life of photogenerated nitroso compound 3

o-NBA amide 1 (4 mM) in MeOH was treated with 365 nm UV light for 7 min and then was added to 100 mM PBS/MeOH (2:1, pH = 7.4) at a final concentration of 2 mM. The mixture was shaken at 25 ^{o}C for 0 /10 min/30 min/1 h/3 h/6 h/12 h/24 h/48 h. The samples were collected, diluted with MeOH/H_2O and analyzed by UPLC-MS. The nitroso compound 4 was quantified by a 0 min control group.

4.4 Reactivity of different o-NBA and amine substrates

Stock solution: o-NBA substrates (10 mM) in MeOH, and amine substrates (10 mM) in MeOH/DMSO/H_2O. o-NBA substrates (2 mM) and amine (0.5 mM) in 100 mM PBS/MeOH (2:1, pH = 7.4) were treated with 365 nm UV light for 7 min and shaken at 25 ^{o}C for 30 min. (Except compound 10/11/13, o-NBA substrate and amine were treated with 365 nm UV light for 10 min. Compound 22/24/25, o-NBA substrate and amine were treated with 365 nm UV light for 15 min. Compound 7, the reaction concentration of o-NBA substrate was 4 mM.) The samples were collected, diluted with MeOH/H_2O and analyzed by UPLC-MS. Yields were determined by ratio of peak area value of experiment to that of internal standard product on reverse-phase HPLC, are reported as an average of three independent trials. All of synthetic

procedures for starting materials, the NMR and HRMS date of products, see in Synthesis Procedures and Compounds Date Part. Yields and products analysis see LC-MS Analysis of Reactions and Products of Small Molecules Part for HPLC trace.

4.5 The selectivity of o-NBA amide 1 reaction towards Cbz-Ser-OMe/Cbz-Tyr-OMe/Cbz-Glu-OMe/Cbz-Asn-OH/ Cbz-Arg-OH/Cbz-His-OH/Cbz-Trp-OH

Stock solution: o-NBA amide 1 (10 mM) in MeOH, Cbz-Lys-OMe (10 mM) in MeOH, other Cbz-protected amino acid (10 mM) in MeOH. o-NBA amide 1 (2 mM), Cbz-Lys-OMe (0.5 mM) and other Cbz-protected amino acid (0.5 mM) in 100 mM PBS/MeOH (2:1, pH = 7.4) were treated with 365 nm UV light for 7 min and shaken at 25 ^{o}C for 30 min. The samples were collected, diluted with MeOH/H_2O and analyzed by UPLC-MS. The yield of indazolone product was quantified by the standard curve and the reactivity with other amino acid was analyzed by comparing the peak areas of the reaction starting materials.

4.6 The selectivity of o-NBA amide 1 reaction towards Cbz-Cys-OMe

Stock solution: o-NBA amide 1 (20 mM) in MeOH, Cbz-Lys-OMe (10 mM) in MeOH and Cbz-Cys-OMe (10 mM) in MeOH and DTT (320 mM) in H_2O. o-NBA amide 1 (4 mM), Cbz-Lys-OMe (0.5 mM) and Cbz-Cys-OMe (0.5 mM) in 100 mM PBS/MeOH (2:1, pH = 7.4) were treated with 365 nm UV light for 7 min and DTT (16 mM) was added and shaken for 1 min. The reaction mixture was shaken at 25 ^{o}C for 30 min. The samples were collected, diluted with MeOH/H_2O and analyzed by UPLC-MS. The yield of indazolone product was quantified by the standard curve and the reactivity with Cbz-Cys-OMe was analyzed by comparing the peak areas of the reaction starting materials.

4.7 o-NBA amide 1 reaction with NH_2-Lys (Cbz)-OMe (α-NH_2) and Cbz-Lys-OMe (ε-NH_2)

Stock solution: o-NBA amide 1 (2.5 mM/5 mM/10 mM/17.5 mM/25 mM) in MeOH, Cbz-Lys-OMe (10 mM) in MeOH and NH_2-Lys (Cbz)-OMe (10 mM) in MeOH. o-NBA amide 1 (0.5 mM/1 mM/2 mM/3.5 mM/5 mM), Cbz-Lys-OMe (0.5 mM) and NH_2-Lys (Cbz)-OMe (0.5 mM) in 100 mM PBS/MeOH (2:1, pH = 7.4) were treated with 365 nm UV light for 7 min and shaken at 25 ^{o}C for 0 or 30 min. The samples were collected, diluted with MeOH/H_2O and analyzed by UPLC-MS. The yield of α-NH_2 and ε-NH_2 indazolone product was quantified by the standard curve.

4.8 The stability of indazolone product

Stock solution: Indazolone product (compound 3) (10 mM) in MeOH. Indazolone product (0.5 mM) in 100mM PBS/MeOH (2:1, pH = 6.0/7.4/8.0) was shaken at 25 ^{o}C for 0/1 h/3 h/6 h/12 h/24 h. The samples were collected, diluted with MeOH/H_2O and analyzed by UPLC-MS. The stability of indazolone product was analyzed by its peak area.

4.9 The reaction of o-NBA-tag with peptide Ac-RYKSHWN-OH/Ac-SRKYDH-OH

Stock solution: o-NBA-tag (5 mM) in MeOH, and peptide (10 mM) in H_2O. o-NBA-tag (1.25 mM) and peptide (0.5 mM) in 100 mM PBS/MeOH (9:1, pH = 7.4) were treated with 365 nm UV light for 7 min and shaken at 25 ^{o}C for 30 min. The samples were collected, diluted with MeOH/H_2O and analyzed by UPLC-MS.

***o*-NBA-N$_3$ + Ac-SRKYDH-OH**

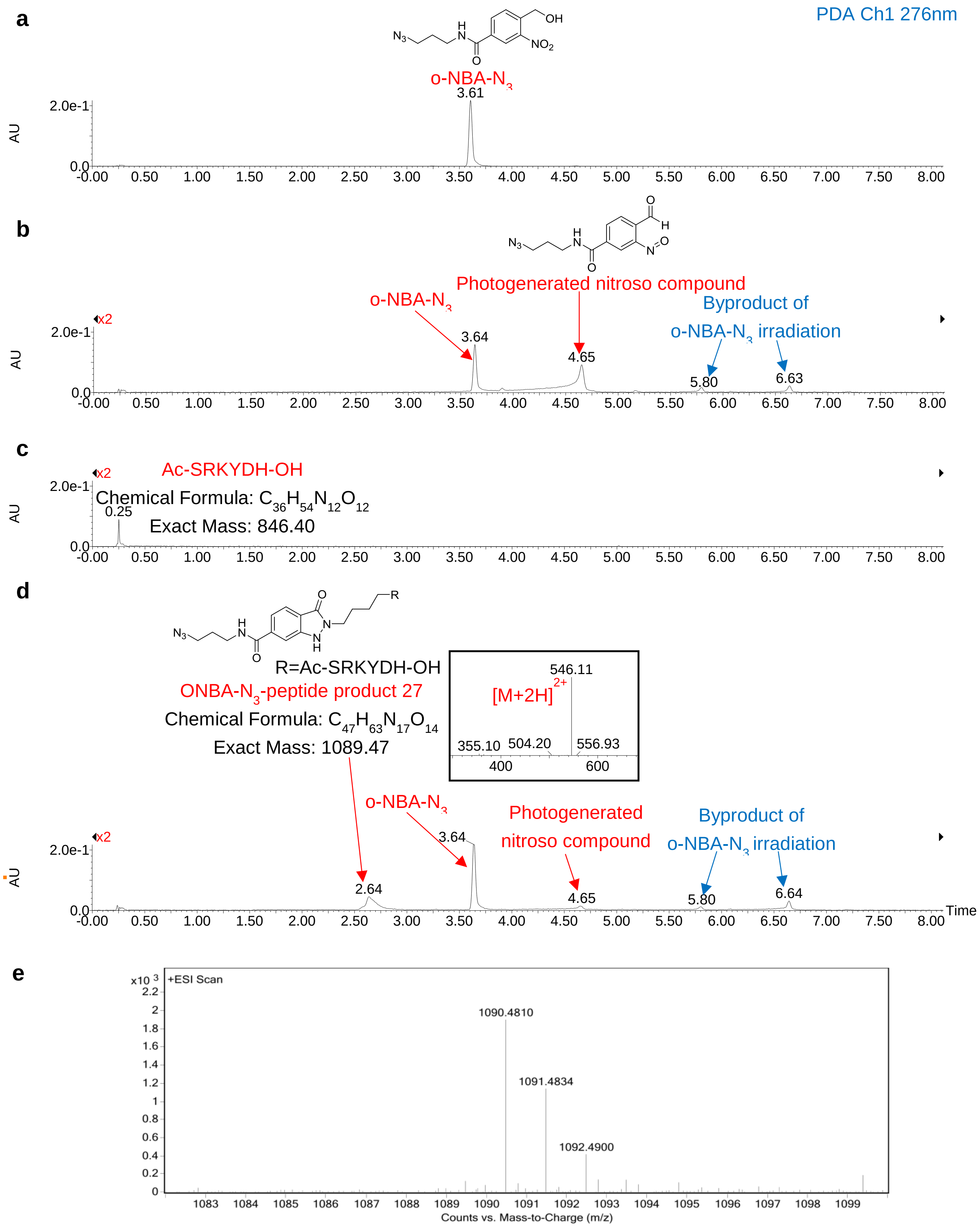

Formula Calculator Results

m/z	Calc m/z	Diff (mDa)	Diff (ppm)	Ion Formula	Ion
1090.481	1090.4813	0.3	0.28	C47 H64 N17 O14	(M+H)+

Supplementary Fig. 22 | a) o-NBA-N$_3$ (1.25 mM); b) o-NBA-N$_3$ (1.25 mM) was irradiated with 365 nm UV light; c) Ac-SRKYDH-OH (0.5 mM); d) o-NBA-N$_3$ (1.25 mM) and Ac-SRKYDH-OH (0.5 mM) were treated with 365 nm UV light; e) HRMS (ESI-Q-TOF) of o-NBA-N$_3$-peptide product.

o-NBA-biotin + Ac-RYKSHWN-OH

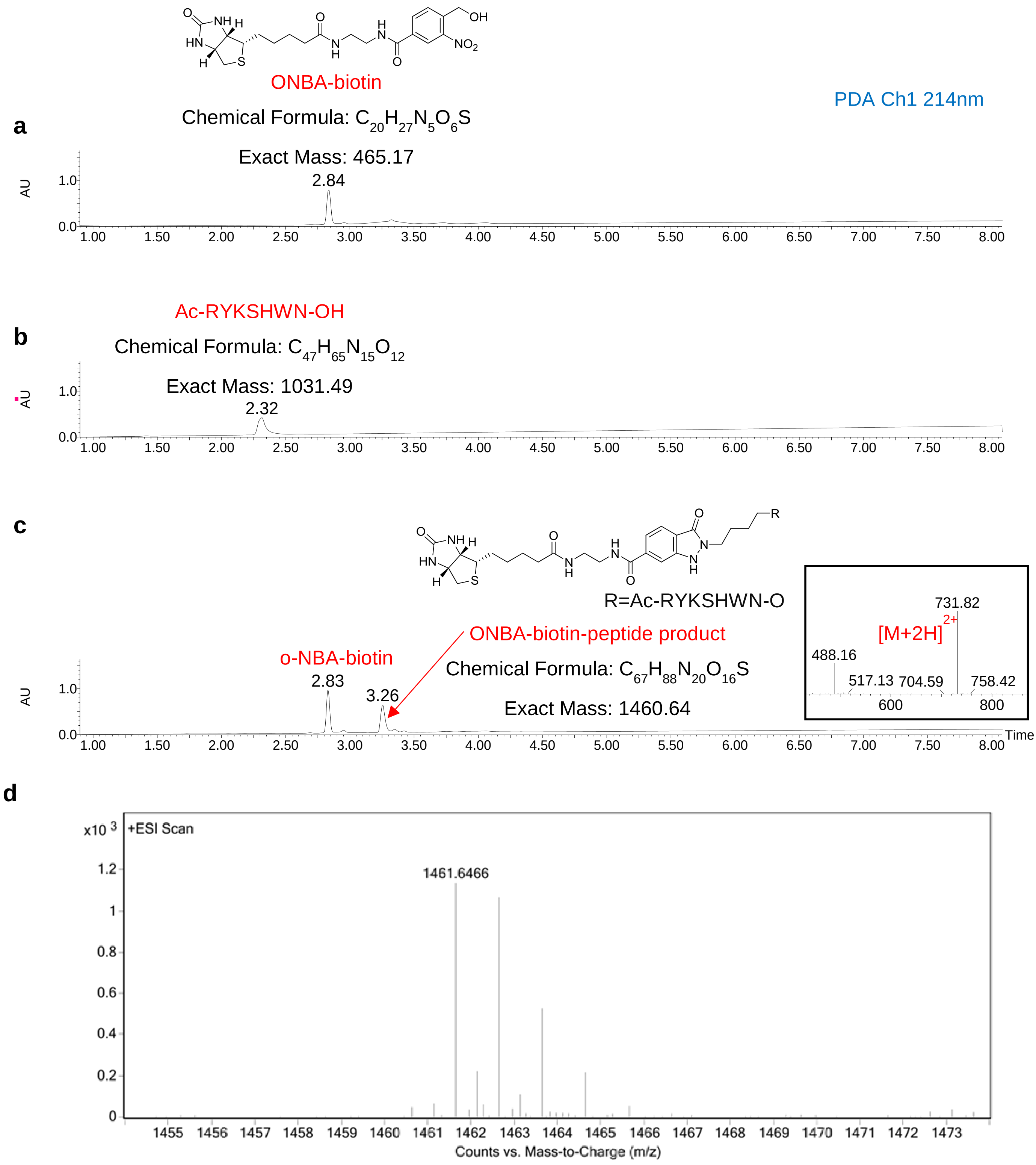

Formula Calculator Results

m/z	Calc m/z	Diff (mDa)	Diff (ppm)	Ion Formula	Ion
1461.6466	1461.6481	1.5	1.02	C67 H89 N20 O16 S	(M+H)+

Supplementary Fig. 23 | a) o-NBA-biotin (1.25 mM); b) Ac-RYKSHWN-OH (0.5 mM); c) o-NBA-biotin (1.25 mM) and Ac-RYKSHWN-OH (0.5 mM) were treated with 365 nm UV light; d) HRMS (ESI-Q-TOF) of o-NBA-biotin-peptide product.

4.10 The reaction of o-NBA-tag with peptide Ac-RCYMNK-OH

Stock solution: o-NBA-tag (10 mM) in MeOH, peptide (10 mM) in H_2O and DTT (160 mM) in H_2O. o-NBA-tag (2 mM) was treated with 365 nm UV light for 7 min and then was added to peptide (0.5 mM) in 100mM PBS/MeOH (9:1, pH = 7.4) 5 aliquots with a 6 min-interval. Then, DTT (8 mM) was added and shaken for 1 min. The samples were collected, diluted with MeOH/H_2O and analyzed by UPLC-MS.

o-NBA-alkyne + Ac-RCYMNK-OH

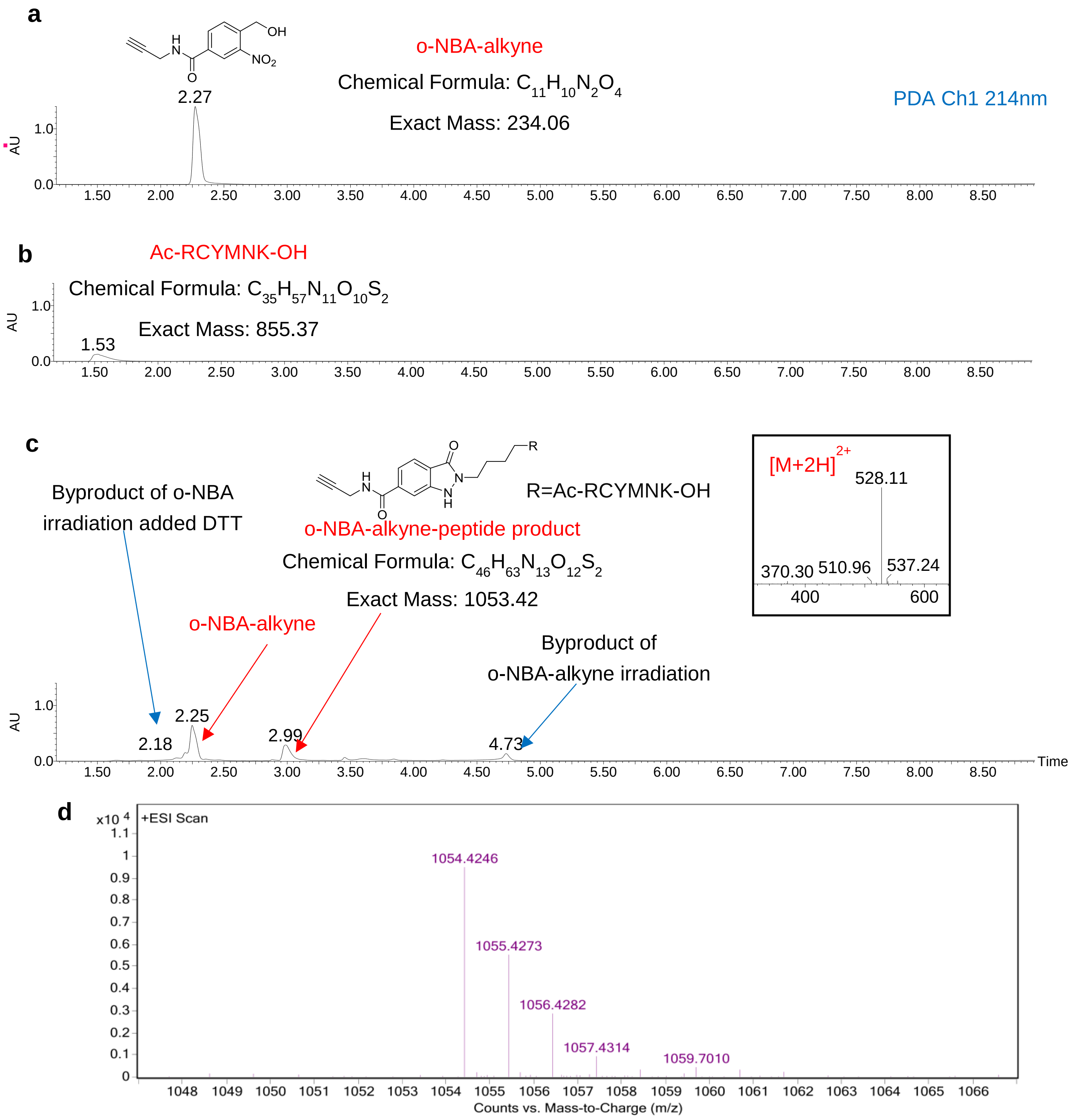

Formula Calculator Results

m/z	Calc m/z	Diff (mDa)	Diff (ppm)	Ion Formula	Ion
1054.4246	1054.4233	-1.3	-1.24	C46 H64 N13 O12 S2	(M+H)+

Supplementary Fig. 24 | a) o-NBA-alkyne (2 mM); b) Ac-RCYMNK-OH (0.5 mM); c) o-NBA-alkyne (2 mM) was irradiated with 365 nm UV light and then was added to Ac-RCYMNK-OH (0.5 mM) 5 aliquots and added DTT (8 mM); d) HRMS (ESI-Q-TOF) of o-NBA-alkyne-peptide product.

4.11 The intermolecular link of peptide Ac-RCYMNK-OH by compound 29

Stock solution: Compound 29 (2.6 mM) in MeOH and peptide (10 mM) in H_2O. Compound 29 (0.52 mM) and peptide (0.5 mM) in 100 mM PBS/MeOH (9:1, pH = 7.4) were shaken at 25 ^{o}C for 1 h. The mixture was treated with 365 nm UV light for 15 min and then was shaken at 25 ^{o}C for 30 min. The samples were collected, diluted with MeOH/H_2O and analyzed by UPLC-MS.

Compound 29 + Ac-RCYMNK-OH

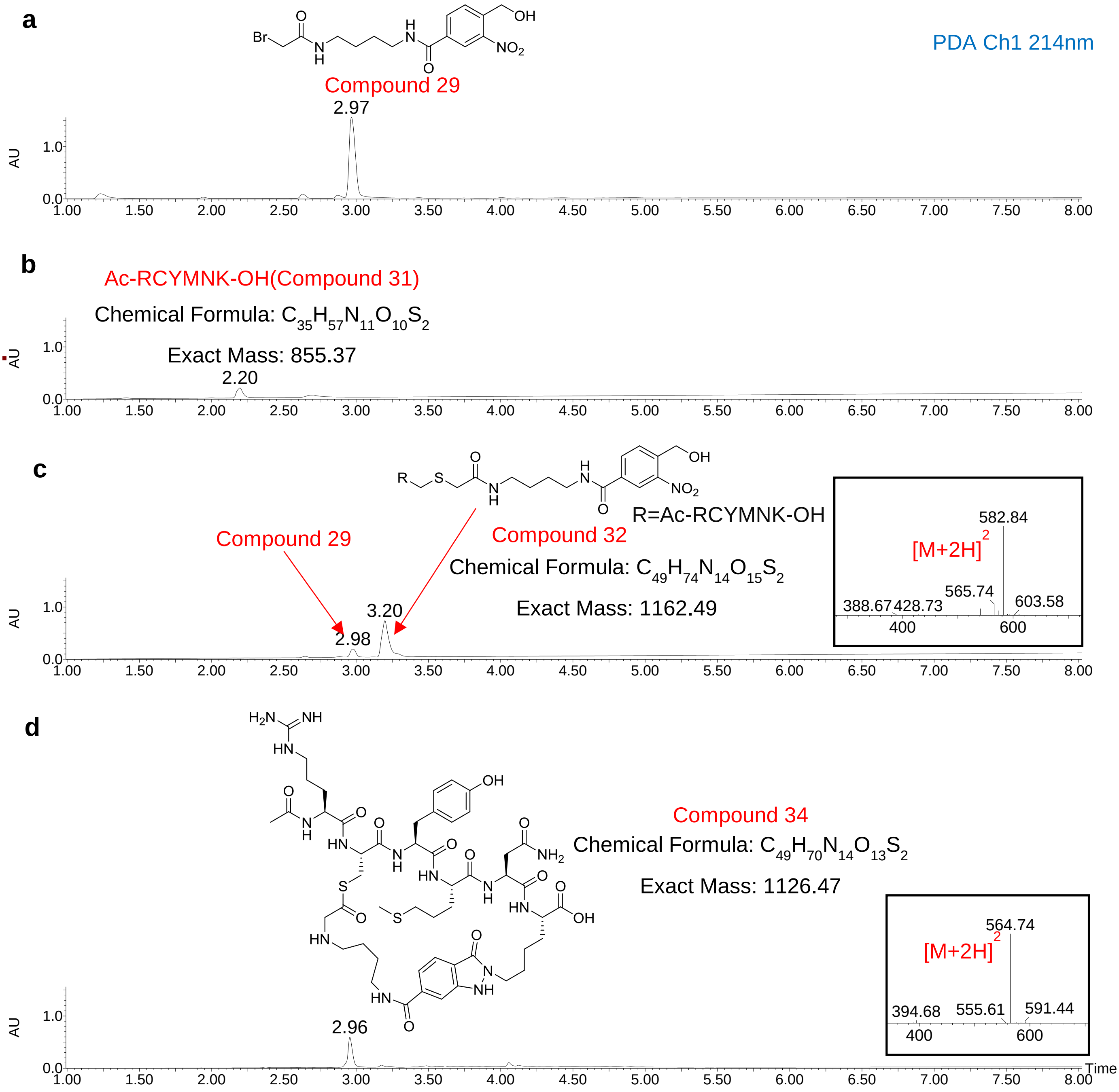

e

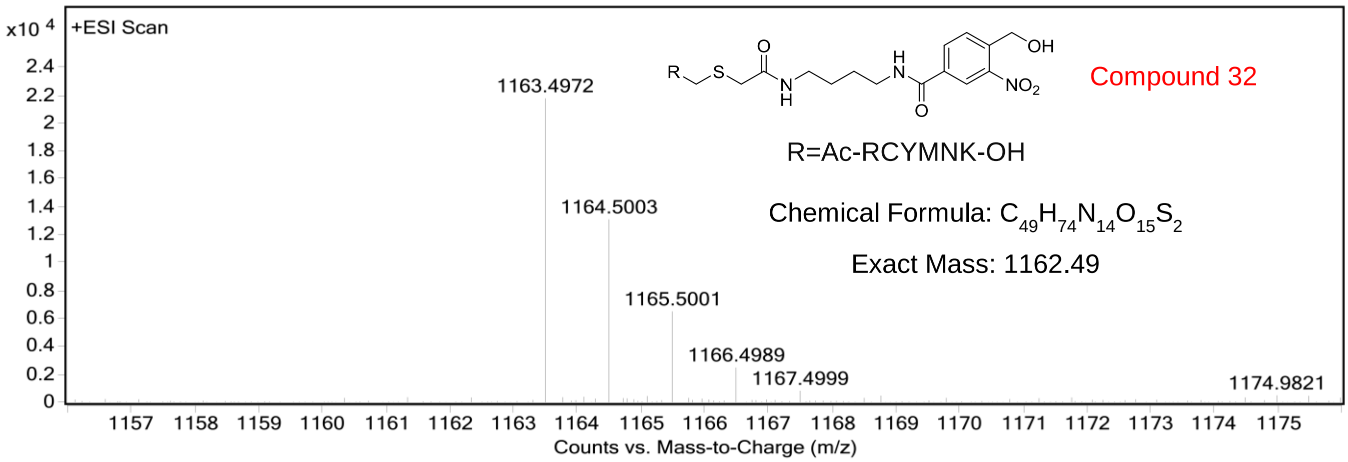

Formula Calculator Results

m/z	Calc m/z	Diff (mDa)	Diff (ppm)	Ion Formula	Ion
1163.4972	1163.4972	0.04	0.04	C49 H75 N14 O15 S2	(M+H)+

f

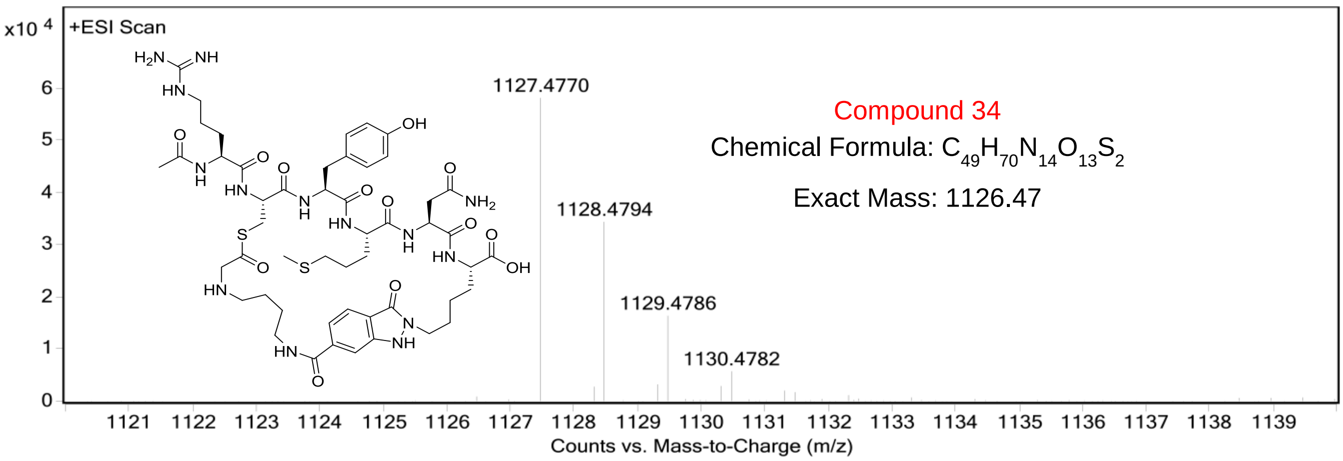

Formula Calculator Results

m/z	Calc m/z	Diff (mDa)	Diff (ppm)	Ion Formula	Ion
1127.477	1127.4761	-0.94	-0.84	C49 H71 N14 O13 S2	(M+H)+

Supplementary Fig. 25 | a) Compound 29 (0.52 mM); b) Ac-RCYMNK-OH (0.5 mM); c) Compound 29 (0.52 mM) reacted with Cys in Ac-RCYMNK-OH (0.5 mM) to afford compound 32; d) Compound 32 was treated with 365 nm UV light to afford compound 34; e) HRMS (ESI-Q-TOF) of compound 32; f) HRMS (ESI-Q-TOF) of compound 34.

4.12 The intermolecular link of peptide Ac-RCYMNK-OH by compound 30

Stock solution: Compound 30 (2.6 mM) in MeOH and peptide (10 mM) in H_2O. Compound 30 (0.52 mM) and peptide (0.5 mM) in 100 mM PBS/MeOH (9:1, pH = 7.4) were shaken at 25 ^oC for 12 h. The mixture was treated with 365 nm UV light for 15 min and then was shaken at 25 ^oC for 30 min. The samples were collected, diluted with MeOH/H_2O and analyzed by UPLC-MS.

Compound 30 + Ac-RCYMNK-OH

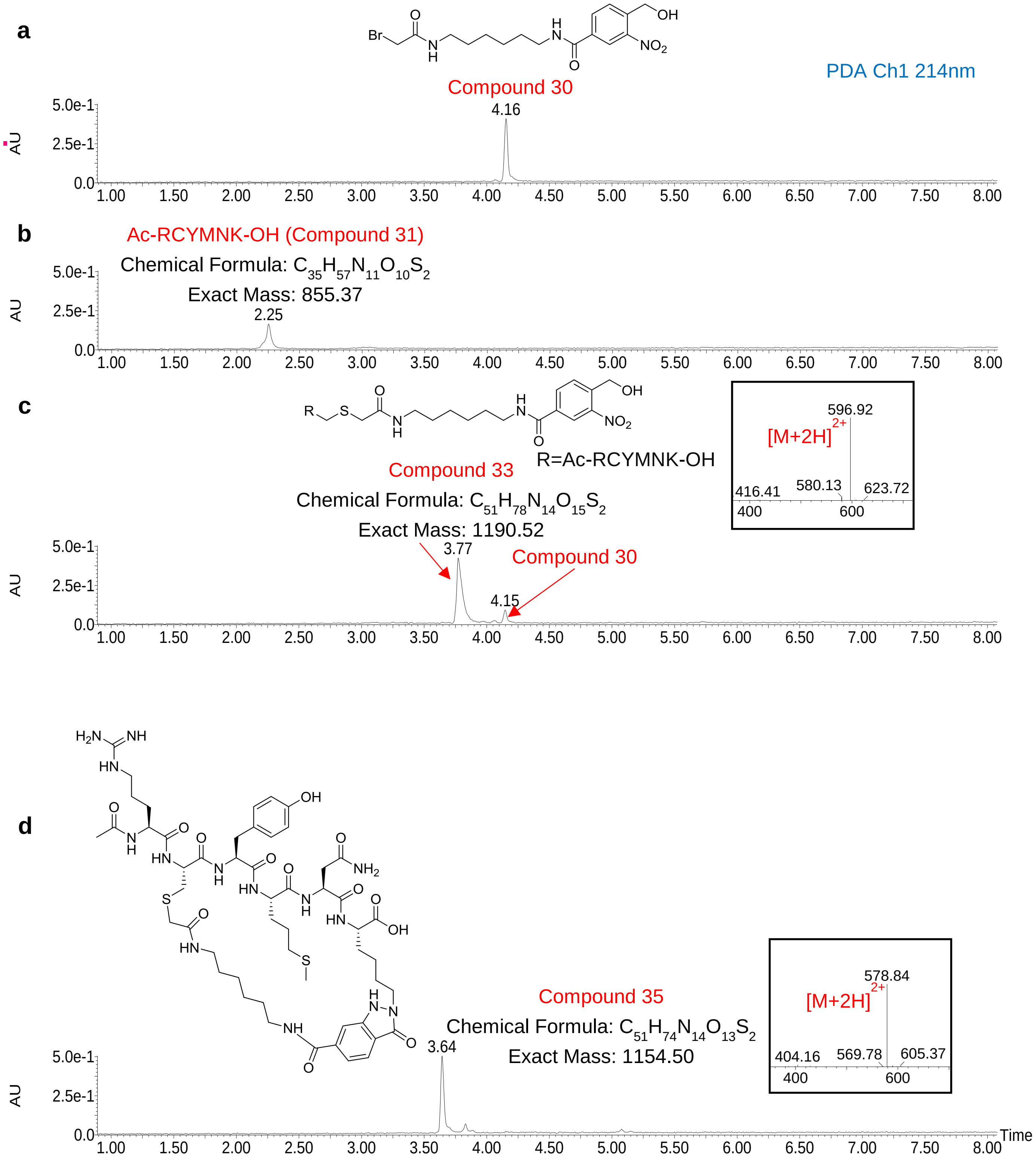

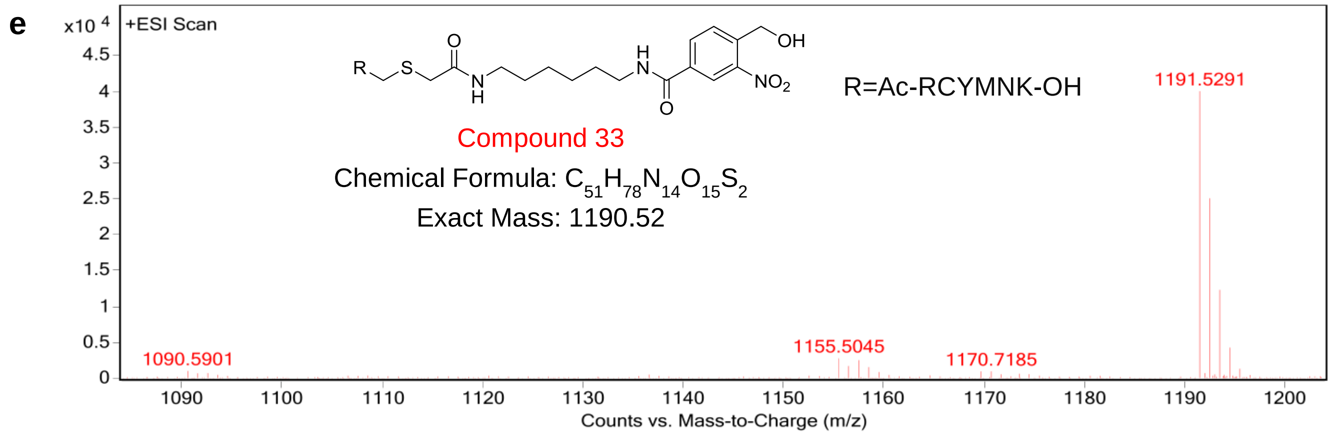

Formula Calculator Results

m/z	Calc m/z	Diff (mDa)	Diff (ppm)	Ion Formula	Ion
1191.5291	1191.5285	-0.56	-0.47	C51 H79 N14 O15 S2	(M+H)+

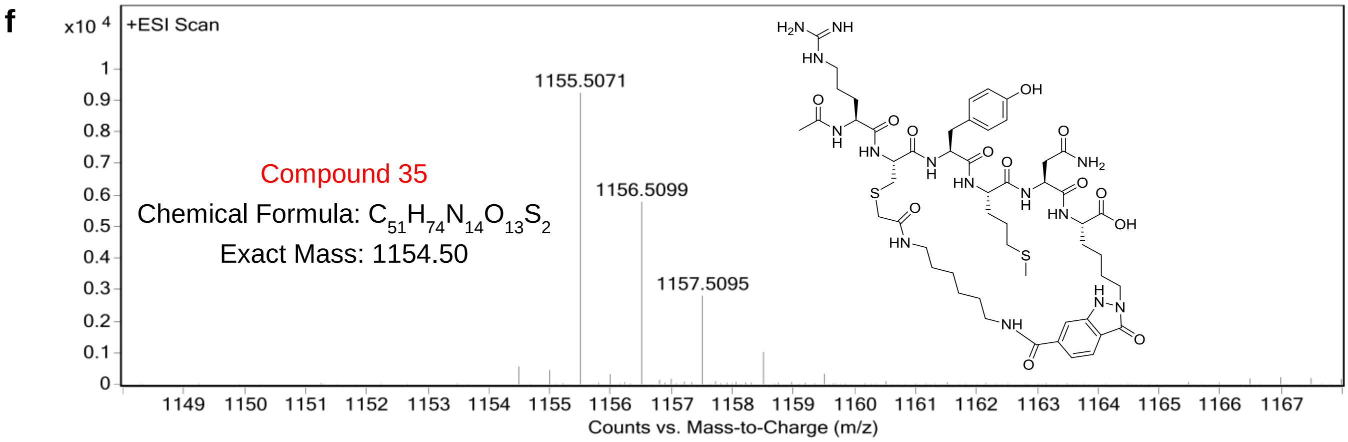

Formula Calculator Results

m/z	Calc m/z	Diff (mDa)	Diff (ppm)	Ion Formula	Ion
1155.5071	1155.5074	0.27	0.23	C51 H75 N14 O13 S2	(M+H)+

Supplementary Fig. 26 | a) Compound 30 (0.52 mM); b) Ac-RCYMNK-OH (0.5 mM); c) Compound 30 (0.52 mM) reacted with Cys in Ac-RCYMNK-OH (0.5 mM) to afford compound 33; d) Compound 33 was treated with 365 nm UV light to afford compound 35; e) HRMS (ESI-Q-TOF) of compound 33; f) HRMS (ESI-Q-TOF) of compound 35.

4.13 The intermolecular link of peptide Ac-RYSHWKN-OH and Ac-RYKSHWN-OH by compound 36

Stock solution: Compound 36 (30 mM) in DMF, pyridine (300 mM) in ACN, NBS (300 mM) in DMF and peptide (10 mM) in H_2O. Compound 20 (30 mM) in DMF was added pyridine (29.4 mM) in ACN and mixed. The mixture was added NBS (29.4 mM) in DMF and shaken at 25 ^{o}C for 5 min. Then the mixture was added to peptide (0.5 mM) in 50 mM PBS/ACN (1:1, pH = 7.4) 5 aliquots with a 1 min-interval.[5] The compound 20-peptide-Tyr product (compound 37) was purified by HPLC in ACN/H_2O, diluted with 100 mM PBS/MeOH (9:1, pH = 7.4), treated with 365 nm UV light for 15 min and then was shaken at 25 ^{o}C for 30 min. The samples were collected, diluted with MeOH/H_2O and analyzed by UPLC-MS.

Compound 36 + Ac-RYSHWKN-OH

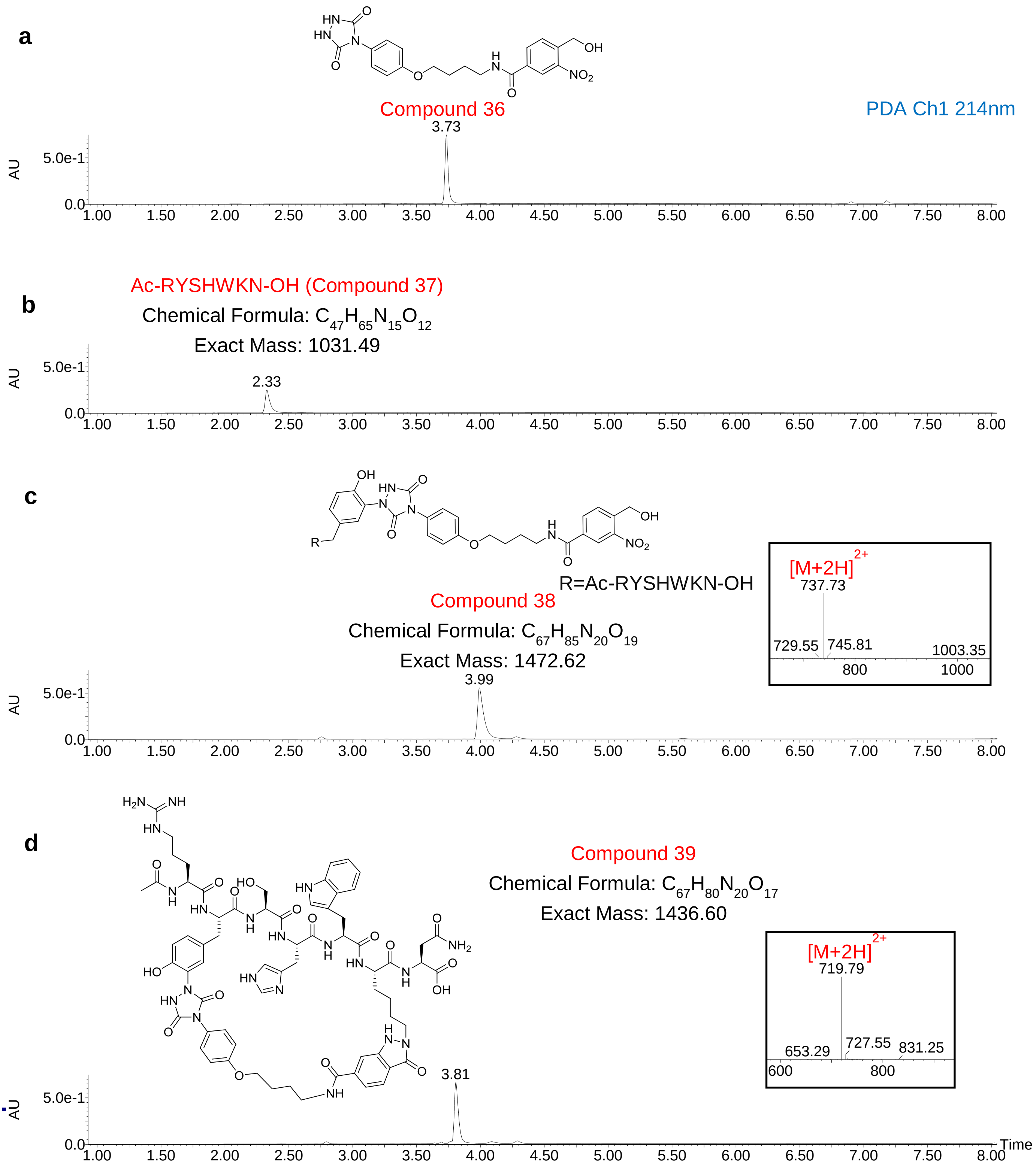

e

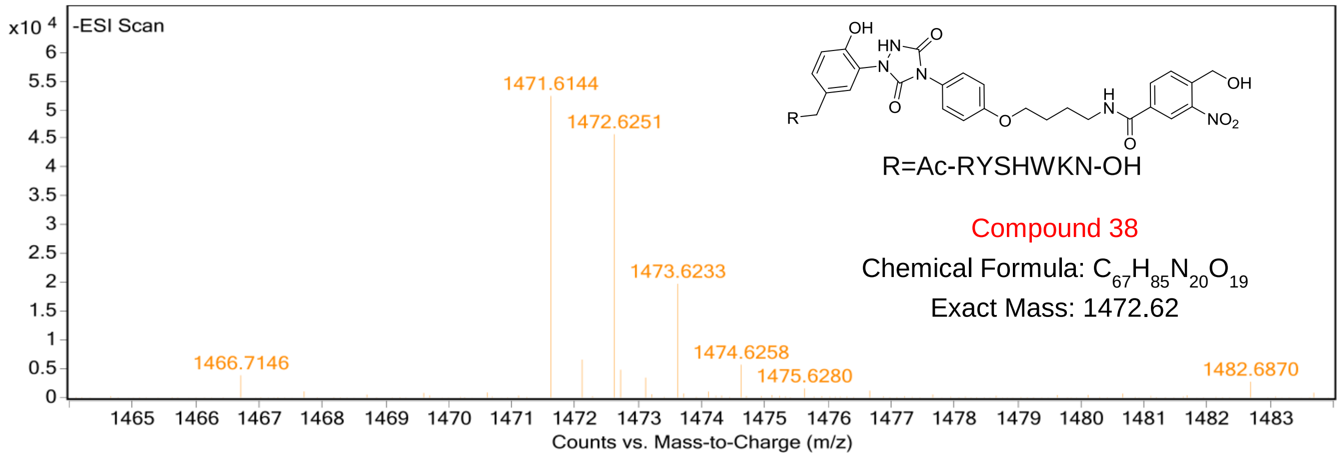

Formula Calculator Results

m/z	Calc m/z	Diff (mDa)	Diff (ppm)	Ion Formula	Ion
1471.6144	1471.6149	0.48	0.33	C67 H83 N20 O19	(M-H)-

f

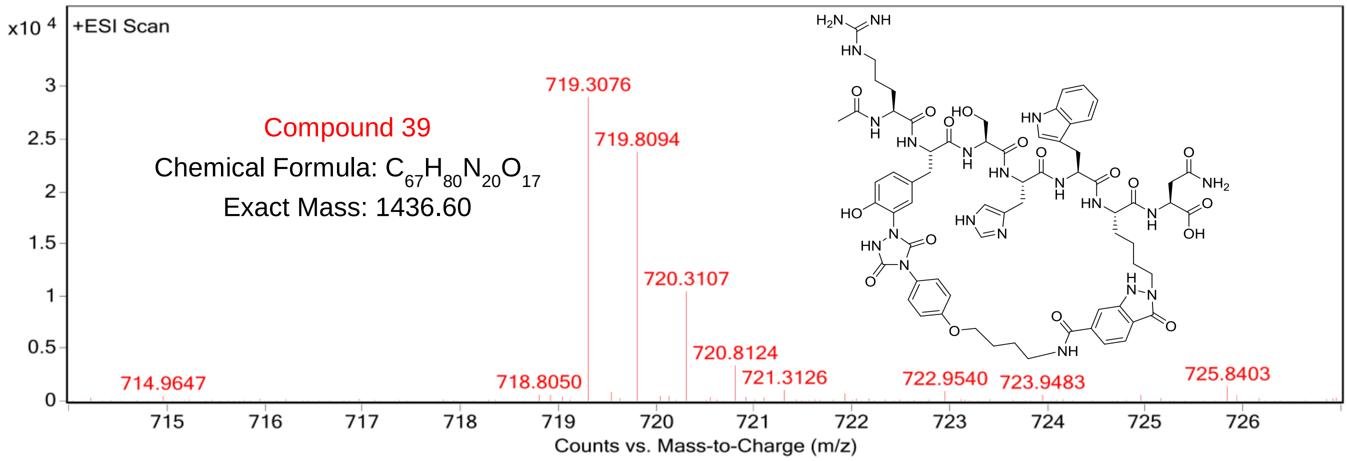

Formula Calculator Results

m/z	Calc m/z	Diff (mDa)	Diff (ppm)	Ion Formula	Ion
719.3076	719.3078	0.24	0.33	C67 H82 N20 O17	(M+2H)+2

Supplementary Fig. 27 | a) Compound 36 (0.5 mM); b) Ac-RYSHWKN-OH (0.5 mM); c) Compound 38; d) Compound 38 was treated with 365 nm UV light to afford compound 39; e) HRMS (ESI-Q-TOF) of compound 38; f) HRMS (ESI-Q-TOF) of compound 39.

4.14 Analysis of o-NBA amide 1 generated byproduct after 365 nm irradiation

a o-NBA amide 1 was irradiated with 365 nm UV light for 7 min and analyzed by UPLC-MS.

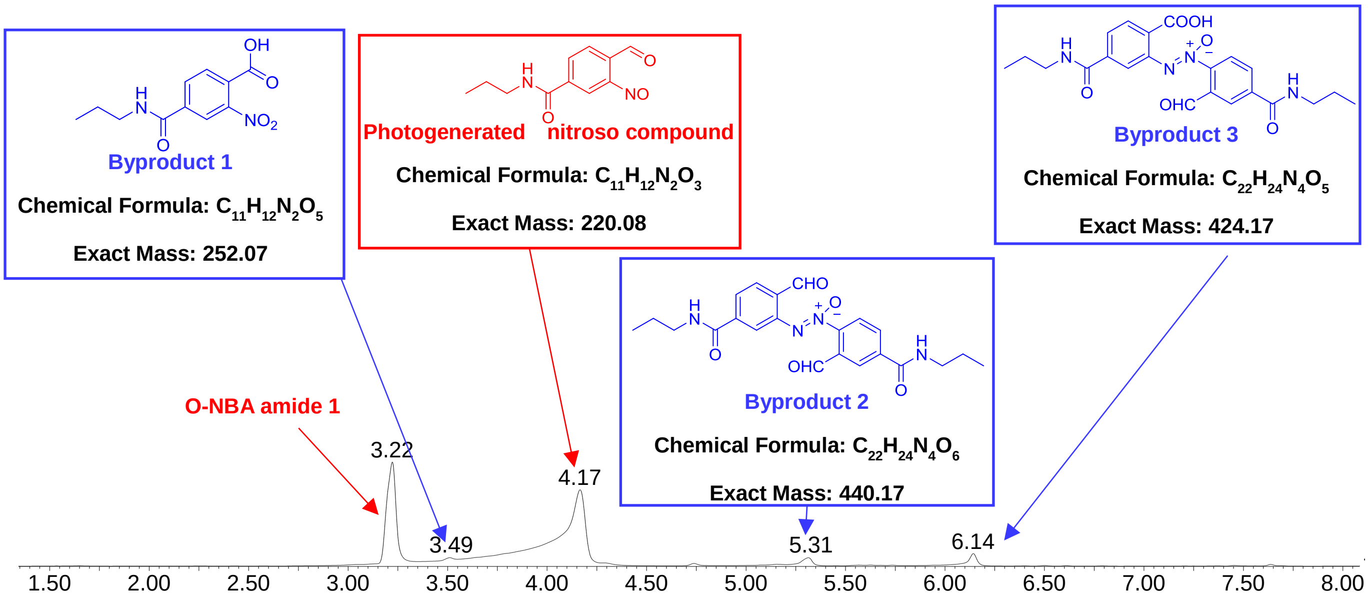

b MS spectrum of Byproduct 1/2/3 and Photogenerated nitroso compound.

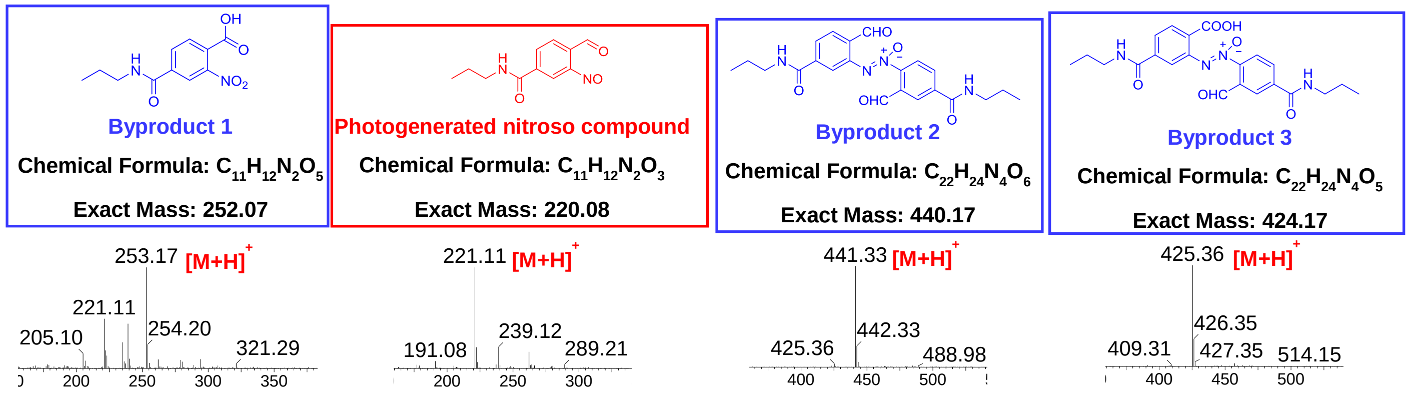

Supplementary Fig. 28 | a) o-NBA amide 1 (2 mM) in 100 mM PBS/MeOH (2:1, pH = 7.4) was irradiated with 365 nm UV light for 7 min and analyzed by UPLC-MS; b) LRMS of Byproduct 1/2/3 and Photogenerated nitroso compound. Byproducts were traces and structures were inferred by references[6-8] and LRMS.

4.15 Analysis of o-NBA amide 1 generated byproduct after 365 nm irradiation in the presence of Cbz-Glu-OMe

a   Cbz-Glu-OMe was injected into UPLC-MS as a control.

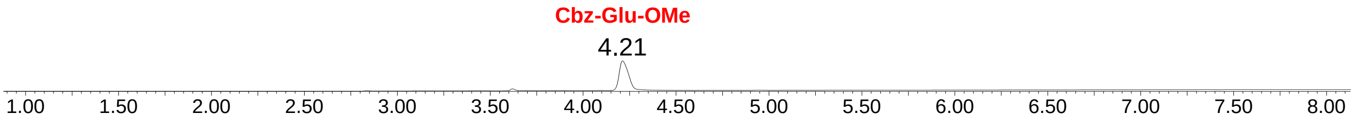

b o-NBA amide 1 and Cbz-Glu-OMe was treated with 365 nm UV light for 7 min and analyzed by UPLC-MS.

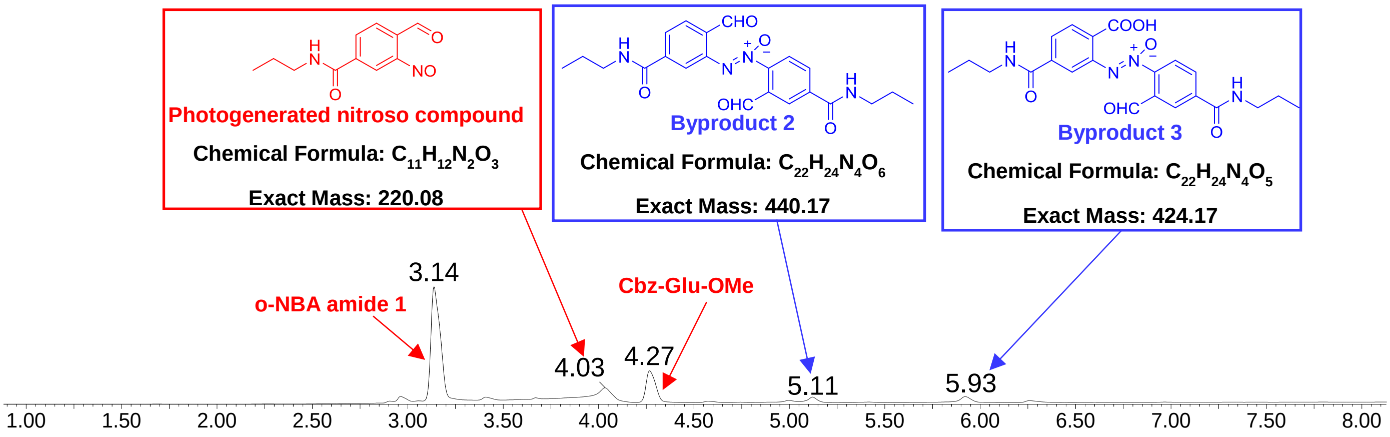

Supplementary Fig. 29 | a) Cbz-Glu-OMe (0.5 mM) in 100 mM PBS/MeOH (2:1, pH = 7.4) was injected into UPLC-MS as a control; b) o-NBA amide 1 (2 mM) and Cbz-Glu-OMe (0.5 mM) 100 mM PBS/MeOH (2:1, pH = 7.4) was treated with 365 nm UV light for 7 min and analyzed by UPLC-MS.

4.16 Analysis of o-NBA amide 1 generated byproduct after 365 nm irradiation in the presence of Cbz-Tyr-OMe

a Cbz-Tyr-OMe was injected into UPLC-MS as a control.

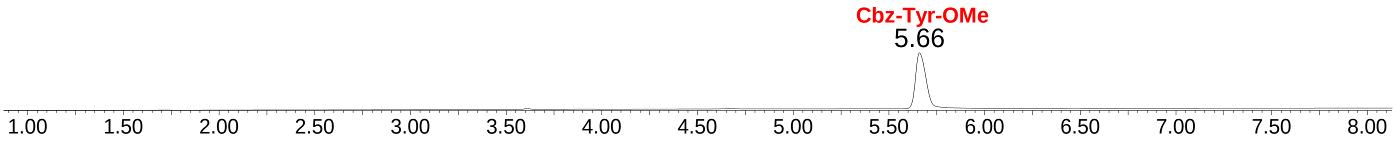

b o-NBA amide 1 and Cbz-Tyr-OMe was treated with 365 nm UV light for 7 min and analyzed by UPLC-MS.

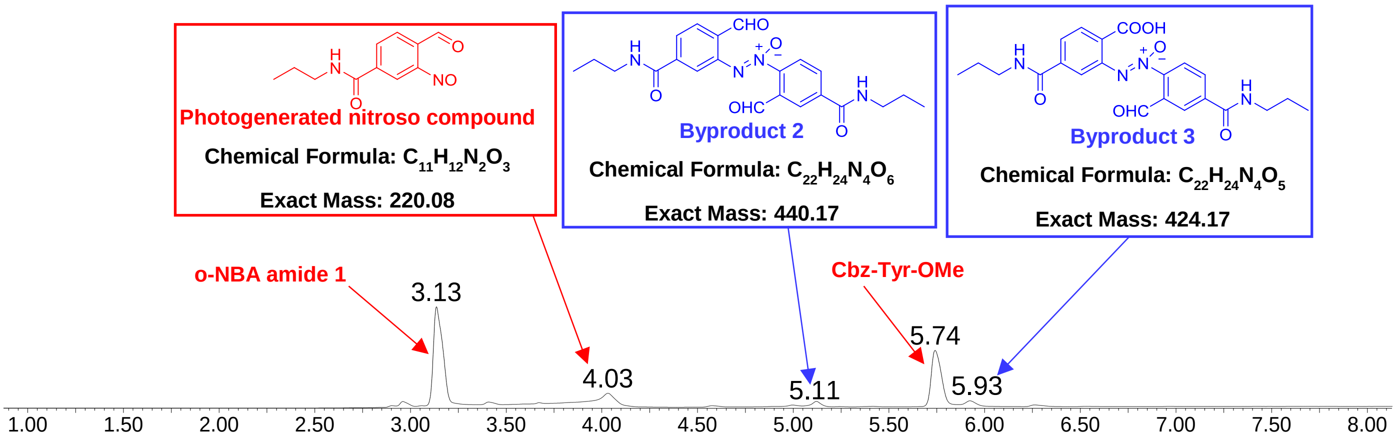

Supplementary Fig. 30 | a) Cbz-Tyr-OMe (0.5 mM) in 100 mM PBS/MeOH (2:1, pH = 7.4) was injected into UPLC-MS as a control; b) o-NBA amide 1 (2 mM) and Cbz-Tyr-OMe (0.5 mM) 100 mM PBS/MeOH (2:1, pH = 7.4) was treated with 365 nm UV light for 7 min and analyzed by UPLC-MS.

4.17 Analysis of o-NBA amide 1 generated byproduct after 365 nm irradiation in the presence of Cbz-Ser-OMe

a **Cbz-Ser-OMe was injected into UPLC-MS as a control.**

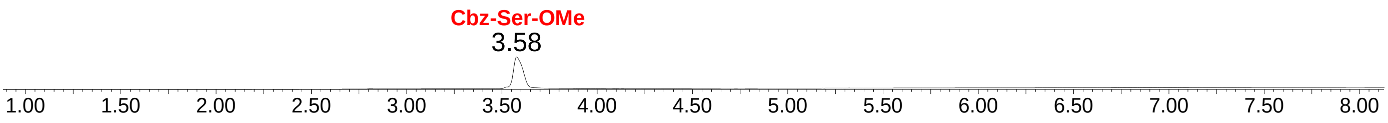

b **o-NBA amide 1 and Cbz-Ser-OMe was treated with 365 nm UV light for 7 min and analyzed by UPLC-MS.**

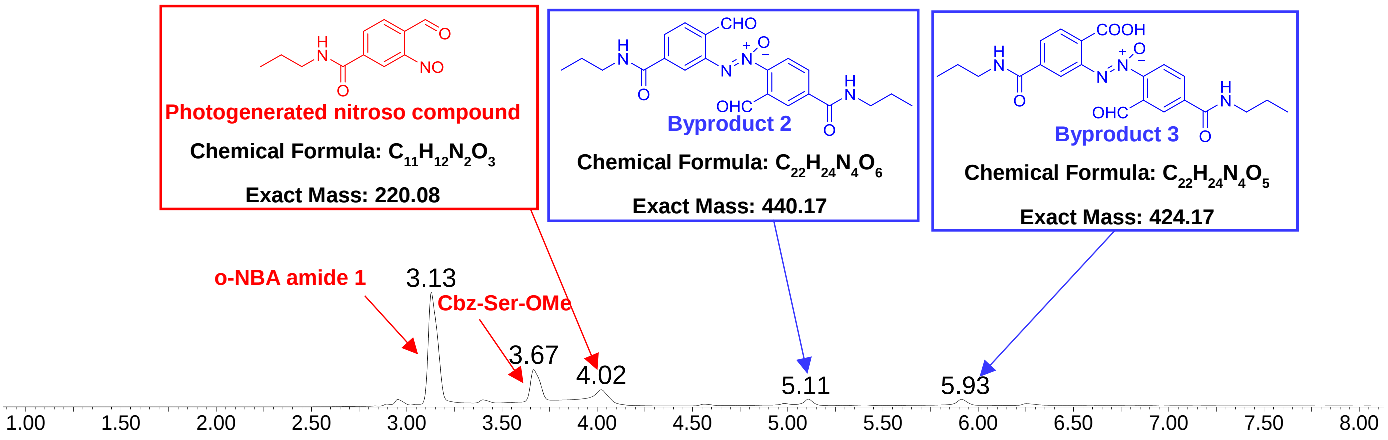

Supplementary Fig. 31 | a) Cbz-Ser-OMe (0.5 mM) in 100 mM PBS/MeOH (2:1, pH = 7.4) was injected into UPLC-MS as a control; b) o-NBA amide 1 (2 mM) and Cbz-Ser-OMe (0.5 mM) 100 mM PBS/MeOH (2:1, pH = 7.4) was treated with 365 nm UV light for 7 min and analyzed by UPLC-MS.

4.18 Analysis of o-NBA amide 1 generated byproduct after 365 nm irradiation in the presence of Cbz-His-OH

a Cbz-His-OH was injected into UPLC-MS as a control.

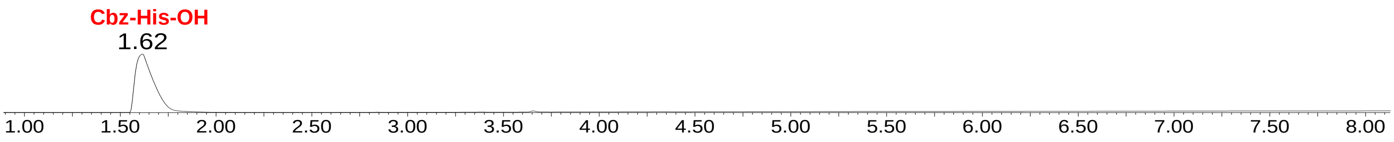

b o-NBA amide 1 and Cbz-His-OH was treated with 365 nm UV light for 7 min and analyzed by UPLC-MS.

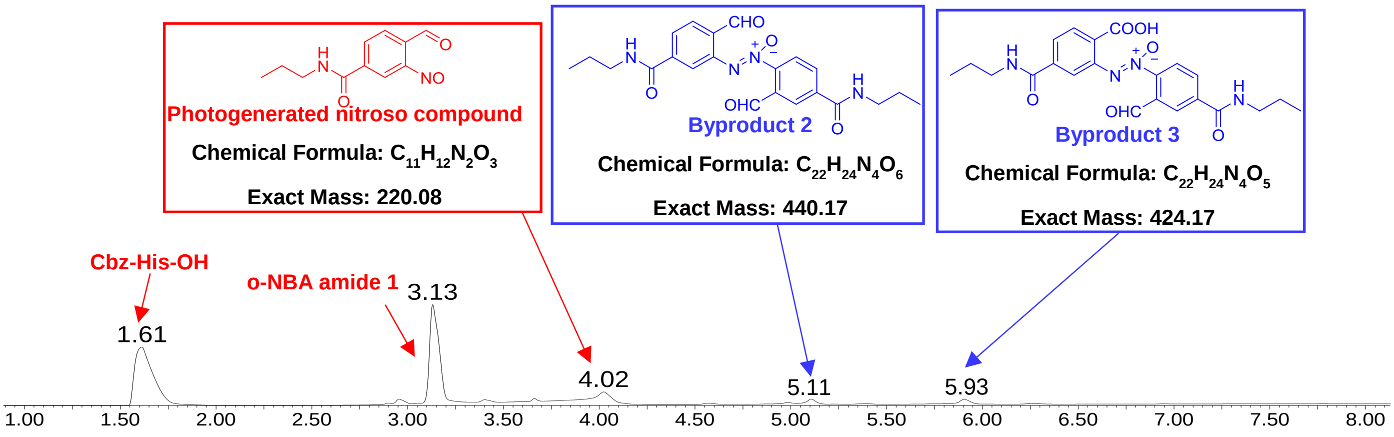

Supplementary Fig. 32 | a) Cbz-His-OH (0.5 mM) in 100 mM PBS/MeOH (2:1, pH = 7.4) was injected into UPLC-MS as a control; b) o-NBA amide 1 (2 mM) and Cbz-His-OH (0.5 mM) 100 mM PBS/MeOH (2:1, pH = 7.4) was treated with 365 nm UV light for 7 min and analyzed by UPLC-MS.

4.19 Analysis of o-NBA amide 1 generated byproduct after 365 nm irradiation in the presence of Cbz-Asn-OH

a Cbz-Asn-OH was injected into UPLC-MS as a control.

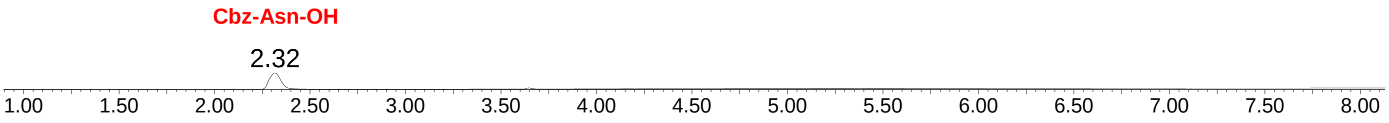

b o-NBA amide 1 and Cbz-Asn-OH was treated with 365 nm UV light for 7 min and analyzed by UPLC-MS.

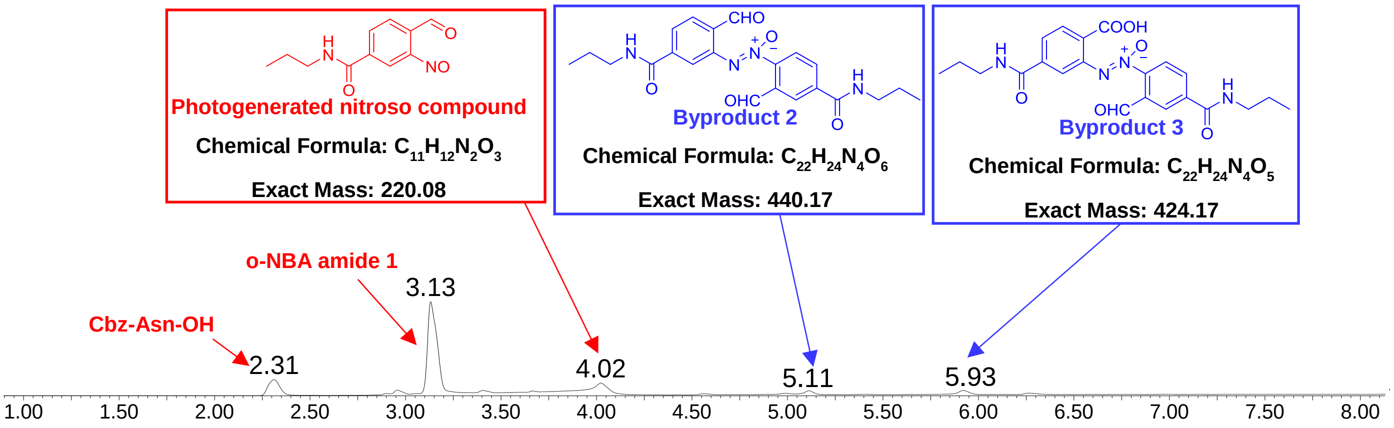

Supplementary Fig. 33 | a) Cbz-Asn-OH (0.5 mM) in 100 mM PBS/MeOH (2:1, pH = 7.4) was injected into UPLC-MS as a control; b) o-NBA amide 1 (2 mM) and Cbz-Asn-OH (0.5 mM) 100 mM PBS/MeOH (2:1, pH = 7.4) was treated with 365 nm UV light for 7 min and analyzed by UPLC-MS.

4.20 Analysis of o-NBA amide 1 generated byproduct after 365 nm irradiation in the presence of Cbz-Cys-OMe

a **Cbz-Cys-OMe was injected into UPLC-MS as a control.**

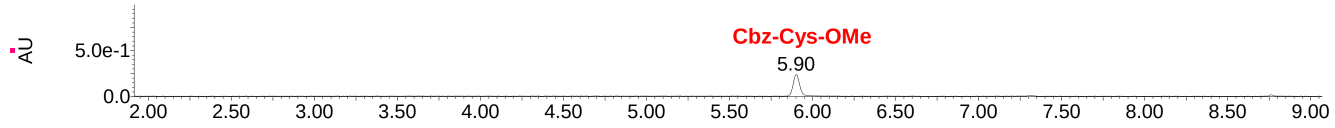

b **o-NBA amide 1 and Cbz-Cys-OMe were treated with 365 nm UV light for 7 min and analyzed by UPLC-MS.**

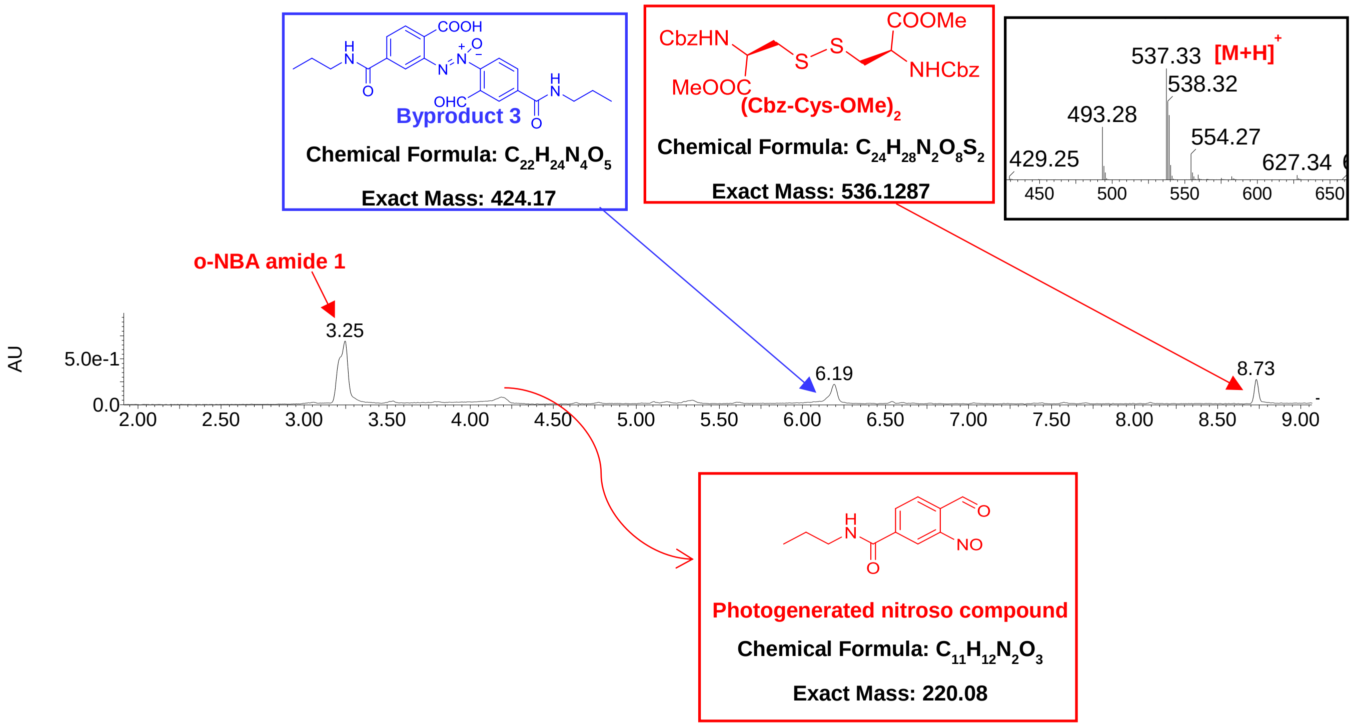

Supplementary Fig. 34 | a) Cbz-Cys-OMe (0.5 mM) in 100 mM PBS/MeOH (2:1, pH = 7.4) was injected into UPLC-MS as a control; b) o-NBA amide 1 (2 mM) and Cbz-Cys-OMe (0.5 mM) in 100 mM PBS/MeOH (2:1, pH = 7.4) was treated with 365 nm UV light for 7 min and analyzed by UPLC-MS.[7,8]

4.21 UV-Vis spectrum of o-NBA amide 1 before and after irradiation

Stock solution: o-NBA amide 1 (10 mM) in MeOH. o-NBA amide 1 (20 μM) in 10 mM PBS/MeOH (1:1, pH = 7.4) were treated with 365 nm UV light for 0/5/10/20/30 min and analyzed by UV-Vis spectrophotometer (UV2045, Shimadzu).

4.22 Determining quantum yields of o-NBA amide 1 irradiation

Photoinduced formation of POM^{-} over a period of 30 sec was determined by UV-Vis spectrophotometer ($\boldsymbol{\varepsilon}_{\mathbf{730}}$ = 2100 M^{-1} cm^{-1}). ($[Fe^{3+}]_0$ = 5 mM, $[C_2O_4^{2-}]_0$ = 60 mM, $[SiW_{12}^{4-}]_0$ = 1 mM.) The zero-order reaction constant of o-NBA amide 1 (20 μM) and methylamine (200 μM) over 120s was determined by UPLC-MS. The absorbance (A_s) of ferrioxalate-polyoxometalate at 365 nm was 2.46 determined by UV-Vis spectrophotometer. The quantum yield ($\boldsymbol{\Phi}_{\mathbf{n}}$) of o-NBA amide 1 under 365 nm excitation to generate o-nitroso benzaldehyde was calculated using the following equation: $\boldsymbol{\Phi}_{\mathbf{n}} = (A_r/A_n)\ (\mathbf{k_n}/\mathbf{k_r})\ \boldsymbol{\Phi}_{\mathbf{r}} = (\boldsymbol{\varepsilon}_{\mathbf{r}} c_r/\boldsymbol{\varepsilon}_{\mathbf{n}} c_n)\ (\mathbf{k_n}/\mathbf{k_r})\ \boldsymbol{\Phi}_{\mathbf{r}}$.

4.23 Reaction constant determination of o-NBA amide 1 and Cbz-Lys-OMe photo-reaction

Stock solution: o-NBA amide 1 (50/100/150/200/250 μM) in MeOH and Cbz-Lys-OMe (0.5/1.0/1.5/2.0/2.5 mM) in MeOH. o-NBA amide 1 (5/10/15/20/25 μM) and Cbz-Lys-OMe (50/100/150/200/250 μM) in PBS/MeOH were treated with 365 nm UV light for 10/20/30/40/50/60/70/80/90 s. The samples were collected and analyzed by UPLC-MS. The yield was quantified by the standard curve of the product.

5. Experimental Procedures for Biomacromolecules

5.1 Expression and purification of Nanobody-HER2

BL21 (DE3) cells were transformed with expression plasmid PET-26b-2RS15d using heat shock and recovered in 500 μL SOC media and incubated at 37 ℃ for 1 h before plating to 2YT agar plate containing 100 μg/mL Ampicillin. A single colony from the plate was picked and used to inoculate 5 mL 2YT containing corresponding probiotic. A 1 mL aliquot of overnight culture was then used to inoculate 100 mL 2YT containing the same concentrations of antibiotics. The cells were grown at 37 ℃ until OD600 reached ~0.5, and the protein expression was induced by adding 1 mM IPTG for 8 h. The cells were pelletized in 50 mL conical tubes by centrifugation at 4200 g for 30 min at 4 ℃ and stored at -80 ℃. Next day, the cell pellets were resuspended in 10 mL lysis buffer. After incubation on ice for 30 min, the lysates were further treated with ultrasonication. Following centrifuge, the supernatants were incubation with 100 μL Ni-NTA agarose beads at 4 ℃ for 2 h with gentle shaking. The resin was centrifuged briefly and washed three times with washing buffer. Finally, the protein was eluted with elution buffer. A 10 μL aliquot from the elution fraction was mixed with one fourth amount of 5 × SDS loading buffer and heated at 98 ℃ for 8 min, before loading onto 12% SDS-PAGE gel. After exchanging to a phosphate buffer (pH 7.5), the protein was analyzed by LC/ESI-MS and the protein mass was obtained by deconvoluting the charge ladder.

5.2 Protein sequences

Nanobody-HER2-wt-His6:
MQVQLQESGGGSVQAGGSLKLTCAASGYIFNSCGMGWYRQSPGRERELVSRISGDGDTWHKESVKGRFTISQDNV
KKTLYLQMNSLKPEDTAVYFCAVCYNLETYWGQGTQVTVSSGGHHHHHH
Lysozyme (purchased):
KVFGRCELAAAMKRHGLDNYRGYSLGNWVCAAKFESNFNTQATNRNTDGSTDYGILQINSRWWCNDGRTPGSR
NLCNIPCSALLSSDITASVNCAKKIVSDGNGMNAWVAWRNRCKGTDVQAWIRGCRL
Myoglobin (purchased):
GLSDGEWQQVLNVWGKVEADIAGHGQEVLIRLFTGHPETLEKFDKFKHLKTEAEMKASEDLKKHGTVVLTALGG
ILKKKGHHEAELKPLAQSHATKHKIPIKYLEFISDAIIHVLHSKHPGDFGADAQGAMTKALELFRNDIAAKYKELGF
QG
Chymotrypsinogen A (purchased):
CGVPAIQPVLSGLSRIVNGEEAVPGSWPWQVSLQDKTGFHFCGGSLINENWVVTAAHCGVTTSDVVVAGEFDQGS
SSEKIQKLKIAKVFKNSKYNSLTINNDITLLKLSTAASFSQTVSAVCLPSASDDFAAGTTCVTTGWGLTRYTNANTPD
RLQQASLPLLSNTNCKKYWGTKIKDAMICAGASGVSSCMGDSGGPLVCKKNGAWTLVGIVSWGSSTCSTSTPGVY
ARVTALVNWVQQTLAAN

5.3 The modification of Lysozyme/Myoglobin with o-NBA-alkyne

o-NBA-alkyne was prepared as a 2.5 mM stock in MeOH solution. Lysozyme/Myoglobin was prepared as a 55 μM stock in PBS solution. o-NBA-alkyne (2.5 mM) in MeOH was treated with 365 nm UV light for 7 min and then was added to Lysozyme/Myoglobin (55 μM) in PBS one portion to a final concentration of 125 μM and mixed. The mixture was shaken at 25 ^{o}C for 1 h. The samples were collected, diluted with H_2O and analyzed by ESI-TOF.

5.4 The modification of Chymotrypsinogen A with o-NBA-alkyne

o-NBA-alkyne was prepared as a 2.5 mM stock in MeOH solution. Chymotrypsinogen A was prepared as a 27.5 μM

stock in PBS solution. o-NBA-alkyne (2.5 mM) in MeOH was treated with 365 nm UV light for 7 min and then was added to Chymotrypsinogen A (27.5 μM) in PBS one portion to a final concentration of 125 μM and mixed. The mixture was shaken at 25 ^{o}C for 1 h. The samples were collected, diluted with H_2O and analyzed by ESI-TOF.

5.5 Trypsinization of protein conjugate and LC-MS/MS analysis

The protein conjugate (50 μM) was reduced with 5 mM DTT at 56 ℃ for 30 min and alkylated with 20mM iodoacetamide in the dark for 15 min. Then the mixture was diluted with 50 mM ammonium bicarbonate. Sequencing grade trypsin (Promega) was incubated with the mixture at 1:10 ratio at 37 ℃ overnight. Digestion was stopped by adding 1% formic acid. The resulting peptides were enriched with C18 OMIX tips (Agilent) and eluted in 50% acetonitrile and 0.1% formic acid. The samples were dried down by SpeedVac and resuspended in 0.1% formic acid for analysis by LC-MS/MS.

5.6 SDS-PAGE of labeled model protein after CuAAC

TAMRA-N_3 was prepared as a 1.25 mM stock in DMSO solution. $CuSO_4$ and THPTA were prepared as a 50 mM stock in H_2O solution and premixed with a volume ratio of 1:5. Sodium ascorbate was prepared as a 100 mM stock in H_2O solution. One hundred microliters of o-NBA-alkyne labeled model proteins (25 μM or 50 μM) were added TAMRA-N_3 (100 μM), premixed $CuSO_4$ (100 μM), THPTA (500 μM) and sodium ascorbate (5 mM). The mixture was rotated at 25 ^{o}C for 1 h and analyzed by SDS-PAGE.

5.7 Fluorescence imaging of live-cell membrane with o-NBA-alkyne modified Nanobody-HER2 followed by TAMRA-N_3 CuAAC.

o-NBA-alkyne was prepared as a 2.5 mM stock in MeOH solution. TAMRA-N_3 was prepared as a 1.25 mM stock in DMSO solution. $CuSO_4$ and THPTA were prepared as a 50 mM stock in H_2O solution and premixed with a volumn ratio of 1:5. Sodium ascorbate was prepared as a 100 mM stock in H_2O solution. Hoechst 33342 was prepared as 1 mg/mL stock solution in H_2O. o-NBA-alkyne (2.5 mM) in MeOH was treated with 365 nm UV light for 7 min, then was added to Nanobody-HER2 (55 μM, 0.77 mg/mL) in PBS one portion to a final concentration of 125 μM and mixed. The mixture was shaken at 25 ^{o}C for 1 h. The samples were collected, diluted with H_2O and analyzed by ESI-TOF. The obtained o-NBA-alkyne modified Nanobody-HER2 (50 μM) was added TAMRA-N_3 (100 μM), premixed $CuSO_4$ (100 μM) & THPTA (500 μM), sodium ascorbate (5 mM). The mixture was rotated at 25 ^{o}C for 1 h. Then excess small molecular impurities were removed and Nanobody-HER2-TAMRA conjugate (15 μM in PBS) was obtained by PD-10 desalting column. SK-Br-3 and MDA-MB-468 cells were seeded to Chambered Coverglass and allowed to grow to approximately 70% confluence. SK-Br-3 and MDA-MB-468 cells were incubated with Nanobody-HER2-TAMRA conjugate (300 nM) in Fluorobrite DMEM at 37 ^{o}C for 1 h and Hoechst 33342 (1 μg/mL) at 37 ^{o}C for 15 min, washed with PBS twice and observed under Leica confocal fluorescence microscope (552 nm and 405 nm).

5.8 Colocalization of Rho-o-NBA on mitochondria

MDA-MB-468 cells were seeded to Chambered Coverglass and allowed to grow to approximately 70% confluence. MDA-MB-468 was incubated with Rho-o-NBA (1 μM) and Rhodamine 123 (400 nM) for 30 min in Fluorobrite DMEM at 37 ^{o}C for 30 min and Hoechst 33342 (1 μg/mL) at 37 ^{o}C for 15 min, washed with PBS twice and observed under confocal fluorescence microscope (552 nm, 488 nm and 405 nm).

5.9 Rho-o-NBA for mitochondria-targeted labeling in live cells via PANAC reaction

MDA-MB-468 cells were seeded to Chambered Coverglass and allowed to grow to approximately 70% confluence. Rho-o-NBA and Rhodamine 123 were prepared as a 1 mM and 400 μM stock in DMSO solution respectively. Hoechst 33342 was prepared as a 1 mg/mL stock solution in H_2O. MDA-MB-468 was incubated with Rho-o-NBA (1 μM) and Rhodamine 123 (400 nM) for 30 min in Fluorobrite DMEM at 37 ^oC for 30 min and Hoechst 33342 (1 μg/mL) at 37 ^oC for 15 min, washed with PBS twice, exposed to 365 nm for 10 min or without light-activation. Then cells fixed with 4% PFA, followed by PBS wash 3 times. The samples were observed under Leica confocal fluorescence microscope (552 nm, 488 nm and 405 nm).

5.10 Treatment of Jurkat and K562 cells with probe-1 and preparation of cell lysates

Jurkat and K562 cells were cultured to a density of $1x10^6$/mL. Cells were incubated with probe-1 (20 μM for Jurkat and 10 μM for K562) in complete medium for 1 h at 37 ℃. Cells were collected, washed with cooled PBS, resuspended in cooled PBS and exposed to 365 nm UV light or not for 10 min on ice. Cells were collected by centrifugation, washed with cooled DPBS and lysed ultrasonically in cooled DPBS containing 1X complete EDTA-free protease inhibitor cocktail. The lysate was centrifuged at 15,000 g for 30 min at 4 ℃, the supernatant was obtained and normalized by BCA protein assay kit.

5.11 Click conjugation with biotin-azide and Streptavidin affinity enrichment of biotinylated proteins

The samples were denatured with 1% SDS and heating and diluted with DPBS to 1 mg/mL concentration for CuAAC. Samples were added TCEP•HCl (2 mM), $CuSO_4$ (1 mM), TBTA (100 μM), biotin-N_3 (25 μM) and rotated for 2 h. Then proteins were precipitated by acetone, washed with MeOH twice and re-dissolved in 1.2% SDS in PBS with heat. Labeled proteins were then immobilized with streptavidin beads (Thermo Fisher Scientific). After washing with PBS and water, beads were rinsed with 6M urea in PBS, added 10 mM DTT and incubated at 65 ^oC for 15 min, 20 mM 2-iodoacetamide and shaken at 37 ^oC for 30 min in the dark. The beads were diluted with PBS, centrifuged and resuspended in 2M urea in PBS.

5.12 On-bead trypsin digestion of pulldown products and LC-MS/MS analysis

Samples were treated once more with 5 mM DTT (5 min), followed by incubation with 400 ng sequencing grade trypsin overnight at 37 ℃. Digestion was stopped by adding 1% formic acid. The resulting peptides were enriched with C18 Omix Tips (Agilent Technologies) and eluted with 50% acetonitrile and 0.1% formic acid. The samples were dried down by SpeedVac and then resuspended in 0.1% formic acid for analysis by LC-MS/MS. Peptides resulting from trypsinization were analyzed on an Orbitrap Velos (Thermo Scientific) connected to a NanoAcquity™ Ultra Performance UPLC system (Waters)(data collection software: Xcalibur 3.0.63). An EasySpray C18 column was used to resolve peptides (90-min gradient with 0.1% formic acidin water as mobile phase A and 0.1% formic acid in acetonitrile as mobile phase B). The LTQ OrbitrapVelos was operated in the data-dependent mode to automatically switch between MS and MS/MS. The top six precursor ions with a charge state of 2^+ or higher were fragmented by CID. All generated peak lists were searched against SwissProt human database using Protein Prospector (data analysis software: Mascot v2.3). The database search was performed with the following parameters: a mass tolerance of 20 ppm for precursor masses; ±0.8 Da for MS/MS, cysteine carbamidomethylation as a fixed modification and methionine oxidation as a variable modification. The enzyme was specified as trypsin with 1 missed cleavage allowed.

5.13 Photo-cytotoxicity test of o-NBA compound toward MDA-MB-468 cell

MDA-MB-468 cells were seeded to 96-well plates at 5x10^4 and allowed to grow 2 days. Then cells were incubated with o-NBA amide 1 (4 or 1 μM)/Rho-o-NBA (4 or 1 μM)/DMSO/STS (0.8 μM) for 30 min and were irradiated with 365 nm UV light for 10/20/30 min or without UV. After incubation for another 2 days, cell availability was analyzed by CCK8 kit.

5.14 MDA-MB-486 cells stained with Rho-o-NBA with or without 365nm UV light irradiation

MDA-MB-468 cells were seeded to Chambered Coverglass and allowed to grow to approximately 70% confluence. Rho-o-NBA and Rhodamine 123 were prepared as a 1 mM and 400 μM stock in DMSO solution respectively. Hoechst 33342 was prepared as a 1 mg/mL stock solution in DMSO.

For space-resolved imaging, the aluminum foil as photo-mask was used. MDA-MB-468 was incubated with Rho-o-NBA (1 μM) and Rhodamine 123 (400 nM) for 30 min in Fluorobrite DMEM at 37 ^{o}C for 30 min and Hoechst 33342 (1 μg/mL) at 37 ^{o}C for 15 min, washed with PBS twice. Labeling with spatial control was demonstrated simply by shielding half part of the cells without light irradiation (the aluminum foil as photo-mask), for the other part of the same dish with UV-365 nm light-activation for 10 min. Then cells fixed with 4% PFA, followed by PBS wash 3 times, cells were imaged under Leica confocal fluorescence microscope (552 nm and 405 nm). (for the experiments of Figure 7a, and Supplementary Fig. 12).

5.15 Preparation of sdAb-HLC-o-NBA

sdAB-HLC was expressed and purified according to published literature[1]. sdAb-HLC (0.7 mg/mL) was added 180-fold molar excess of 2-MEA hydrochloride in PBS (pH 7.4) and incubated at 37 ℃ for 90 min. The reduced sdAb-HLC solution was buffer exchanged to 0.2 M NH_4OAc (pH 6.0−6.5) using PD-10 desalting columns and incubated with 10-fold molar excess of compound S40 at 20 ℃ for 1 h. After conjugation complete, the sdAb-HLC-o-NBA was buffer exchanged to PBS (pH 7.4).

sdAB-HLC sequence: MQVQLQESGGGSVQAGGSLKLTCAASGYIFNSCGMGWYRQSPGRERELVSRISGDGDTWH KESVKGRFTISQDNVKKTLYLQMNSLKPEDTAVYFCAVCYNLETYWGQGTQVTVSSGGHHHHHHSPSTPPTPSPST PPC

5.16 sdAb-HLC-o-NBA space-resolved labeling of cell membrane

SK-Br-3 cells were seeded to 35 mm dish and allowed to grow to approximately 70% confluence. SK-Br-3 were incubated with Nanobody-HER2-Cys-o-NBA conjugate (500 nM) in PBS at 37 ^{o}C for 45 min and Hoechst 33342 (1 μg/mL) at 37 ^{o}C for 15 min, washed with PBS twice. Then cells were incubated with FITC-NH_2 (200 μM) for 15 min and treated with 365 nm UV-light for 20 min for labeling of HER2 specific nanobody on the surface of SK-Br-3 cell (for the experiments of Supplementary Fig. 10).

For space-resolved imaging, the aluminum foil as photo-mask was used. SK-Br-3 Cells were incubated with HER2 specific Nanobody-HER2-Cys-o-NBA conjugate (500 nM) in PBS and Hoechst 33324 (1 μg/mL) at 37 ℃ for 1 h and 15 min respectively, washed with PBS 3 times. Labeling with spatial control was demonstrated simply by shielding half part of the cells without light irradiation (the aluminum foil as photo-mask), for the other part of the same dish with UV-365 nm light-activation for 10 min. After labeling, cells were washed by PBS wash for 3 times, cells were imaged under Leica confocal fluorescence microscope (488 nm and 405 nm). (for the experiments of Figure 7b and Supplementary Fig. 11).

5.17 Construction of pcDNA3.1-H2B-mKate2 K175TAG

The coding sequence for H2B-mKate2 was amplified from plasmid pmH2B-6-mKate2 gifted by Prof. Ren[9] and cloned into plasmid pcDNA3.1 using the *HindIII* and *NotI* restriction sites to make the plasmid pcDNA3.1-H2B-mKate2. To construct the mKate2 K175TAG mutant plasmid pcDNA3.1-H2B-mKate2 K175TAG, mutagenesis PCR was performed as the manufacturer's instructions with the following primers:

forward (5'-3'): CTTGTAGACCACATACAGATCCAAGAAACCCGCTAAGAACC;

reverse (5'-3'): TGTATGTGGTCTACAAGTTGCAGATCAGGTGGCCCCCG

using the Hieff Mut^{TM} Site-Directed Mutagenesis Kit (Yeasen, Shanghai, China, #11003ES10). All the constructs were confirmed by DNA sequencing.

5.18 Expression of H2B-mKate2 175oNBAK in mammalian cells

Cell culture coverslips were precoated with Poly-lysine coating reagent and then placed into each well of 12-well plates. Cultured HEK293T cells were planted into each well and cultured in the cell incubator with 5% CO_2 at 37 ^{o}C. After about 17 h at 70-80% cell confluency, the media were refreshed and 1 mM o-NBAK or equal volume of DMSO as negative control were added into the indicated wells. One hour later, plasmids pcDNA3.1-H2B-mKate2 K175TAG (0.5 μg) and pNEU-hMbPylRS-4xU6M15 (0.5 μg, Addgene, #105830) were transfected into one well of the cells using the Lipofectamine 3000 Transfection Kit (Invitrogen, #L3000-015) to express H2B-mKate2 175oNBAK. Cells were harvested at about 24 h after transfection and subjected to subsequent fixation and probing procedures.

5.19 Fixation and labeling of transfected HEK293T cells

Transfected pcDNA3.1-H2B-mKate2 K175TAG HEK293T and pNEU-hMbPylRS-4xU6M15 cells were fixed by 4% PFA and washed with PBS for 3 times with 5 min interval. Then cells were stained with Hoechst 33342 (2 μg/mL) and washed with PBS twice. Then cells were incubated with FITC-NH_2 (20 μM) for 15 min at room temperature and exposed to 365 nm or not for 20 min. The samples were washed 4 times with PBS and observed under Leica confocal fluorescence microscope (552 nm, 488 nm and 405 nm).

5.20 Expression of Mito-EGFP in mammalian cells and labeling

Cultured MDA-MB-468 cells were planted into 6-well plates and cultured in the cell incubator without CO_2 at 37 ^{o}C. After about 24 h at 70-80% cell confluency, 0.5 μg Mito-EGFP plasmid (*Neurosci. Bull.* 2017, **33**, 685-694) was transfected into one well of the cells using the Lipofectamine 3000 Transfection Kit (Invitrogen, #L3000-015) to express Mito-EGFP. Cells were harvested at about 48 h after transfection and subjected to subsequent probing procedures.

Transfected MDA-MB-468 cells was incubated with Rho-o-NBA (1 μM) for 30 min in Fluorobrite DMEM at 37 ^{o}C for 30 min and Hoechst 33342 (1 μg/mL) at 37 ^{o}C for 15 min, washed with PBS twice, exposed to 365 nm for 10 min or not and observed under Leica confocal fluorescence microscope (552 nm, 488 nm and 405 nm).

5.21 Statistics and Reproducibility

The experiments in figure 5c, 5e, 5f and supplementary figure 8, 10, 11 were independently repeated at least 3 times with similar results. The experiments in figure 6e and supplementary figure 12a-b, 14, 15a-b, 20a-b were independently repeated twice with similar results.

6. Synthesis Procedures and Compounds Date

6.1 General procedure for synthesis of o-NBA amide 1, o-NBA-alkyne and o-NBA-N_3

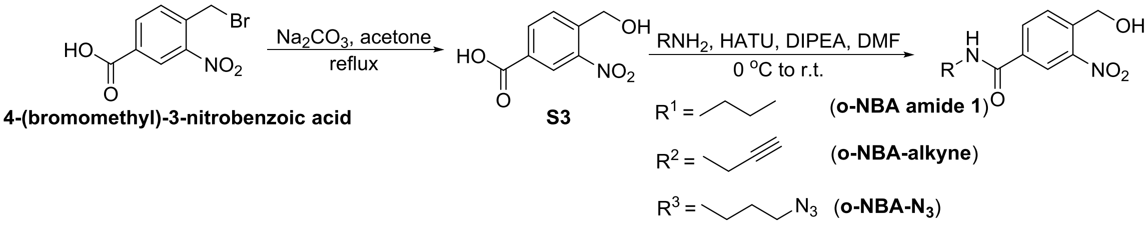

4-(bromomethyl)-3-nitrobenzoic acid (5.00 g, 19.2 mM) and Na_2CO_3 (7.13 g, 67.3 mM) in acotone/H_2O (1:1, 150 mL) were refluxed for 3 h. Acetone was removed in vacuo and the aqueous phase was extracted with Et_2O twice. The aqueous phase was added concentrated HCl until pH = 1 or below and extracted with ethyl acetate 3 times. The organic layer was washed with H_2O and brine, dried over $MgSO_4$ and concentrated in vacuo without further purification to afford **compound S3** as a brown oil (3.78 g, 98%). ^{1}H NMR (500 MHz, MeOD) δ 8.60 (s, 1H), 8.29 (d, *J* = 8.0 Hz, 1H), 7.97 (d, *J* = 8.1 Hz, 1H), 5.00 (s, 2H).

Compound S3 (1.0 equiv), HATU (1.2 equiv) and propylamine (3.0 equiv) or propargylamine (3.0 equiv) or 3-azidopropan-1-amine (1.0 equiv) in anhydrous DMF were added DIPEA (3.0 equiv) dropwise at 0 ^{o}C, and then the mixture was stirred at room temperature overnight. The mixture was added H_2O and extracted with ethyl acetate 3 times. The organic layer was washed with saturated $NaHCO_3$, 0.1 M HCl, H_2O, brine, dried over Na_2SO_4 and concentrated in vacuo. The residue was purified by silica chromatography to afford product.

o-NBA amide 1 Yield: 64%. ^{1}H NMR (400 MHz, MeOD) δ 8.50 (d, *J* = 1.7 Hz, 1H), 8.14 (dd, *J* = 8.1, 1.7 Hz, 1H), 7.96 (d, *J* = 8.2 Hz, 1H), 4.99 (s, 2H), 3.39 – 3.33 (m, 2H), 1.66 (dq, *J* = 14.7, 7.4 Hz, 2H), 1.02 – 0.94 (m, 3H). ^{13}C NMR (101 MHz, MeOD) δ 167.5, 148.4, 142.5, 135.6, 132.9, 129.7, 124.5, 61.8, 42.9, 23.6, 11.8. HRMS (ESI-Q-TOF): m/z $[M+H]^+$ Calcd for $C_{11}H_{15}N_2O_4^+$: 239.1026; found: 239.1040.

o-NBA-alkyne (compound S14) Yield: 58%. ^{1}H NMR (400 MHz, MeOD) δ 8.51 (d, *J* = 1.7 Hz, 1H), 8.15 (dd, *J* = 8.2, 1.8 Hz, 1H), 7.97 (d, *J* = 8.2 Hz, 1H), 4.99 (s, 2H), 4.18 (d, *J* = 2.5 Hz, 2H), 2.64 – 2.62 (m, 1H). ^{13}C NMR (101 MHz, MeOD) δ 167.1, 148.3, 142.8, 134.9, 133.0, 129.8, 124.6, 80.4, 72.3, 61.8, 30.1. HRMS (ESI-Q-TOF): m/z $[M-H]^-$ Calcd for $C_{11}H_9N_2O_4^-$: 233.0568; found: 233.0566.

o-NBA-N_3 Yield: 51%. ^{1}H NMR (500 MHz, MeOD) δ 8.52 (d, *J* = 1.8 Hz, 1H), 8.15 (dd, *J* = 8.1, 1.8 Hz, 1H), 7.98 (d, *J* = 8.1 Hz, 1H), 4.99 (s, 2H), 3.49 (t, *J* = 6.9 Hz, 2H), 3.43 (t, *J* = 6.7 Hz, 2H), 1.90 (p, *J* = 6.8 Hz, 2H). ^{13}C NMR (126 MHz, MeOD) δ 167.7, 148.5, 142.6, 135.5, 133.0, 129.8, 124.5, 61.8, 50.2, 38.6, 29.7. HRMS (ESI-Q-TOF): m/z $[M+Na]^+$ Calcd for $C_{11}H_{13}N_5NaO_4^+$: 302.0860; found: 302.0853.

6.2 Synthesis of Compound S1 and S2

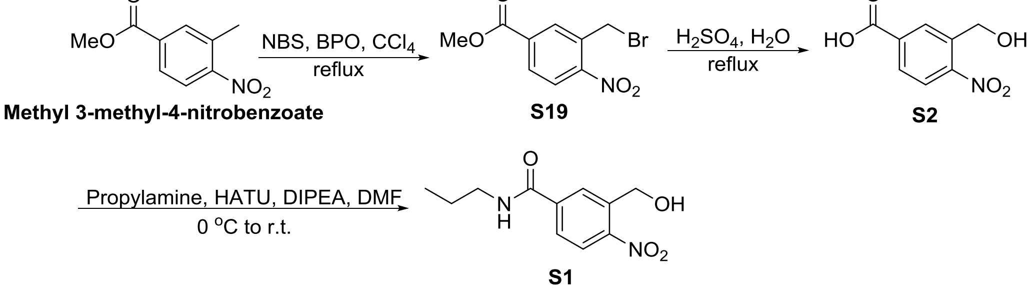

Methyl 3-methyl-4-nitrobenzoate (2.00 g, 10.2 mM) in CCl_4 (50 mL) was added BPO (247 mg, 1.02 mM) and NBS (2.00 g, 11.3 mM) at room temperature. The mixture was refluxed overnight. The cooled mixture was filtered and washed

with hexane. The filtrate was concentrated in vacuo and the residue was purified by silica chromatography to afford **compound S19** as a brown oil (1.44 g, 51%). ^{1}H NMR (400 MHz, $CDCl_3$) δ 8.24 (d, *J* = 1.7 Hz, 1H), 8.13 (dd, *J* = 8.5, 1.8 Hz, 1H), 8.06 (d, *J* = 8.5 Hz, 1H), 4.83 (s, 2H), 3.99 (s, 3H).

A mixture of **compound S19** (1.44 g, 5.25 mM), concentrated sulfuric acid (3.2 mL) and H_2O (50 mL) was refluxed overnight. The cooled mixture was extracted with ethyl acetate. The organic layer was washed with H_2O, brine, dried over Na_2SO_4 and concentrated in vacuo. The residue was purified by silica chromatography to afford **compound S2** as a brown oil (859 mg, 83%). ^{1}H NMR (500 MHz, MeOD) δ 8.25 (s, 1H), 7.88 – 7.83 (m, 2H), 4.74 (s, 2H). ^{13}C NMR (126 MHz, MeOD) δ 168.9, 150.7, 139.1, 138.0, 130.9, 130.0, 125.6, 61.7. HRMS (ESI-Q-TOF): m/z $[M-H]^-$ Calcd for $C_8H_6NO_5^-$: 196.0251; found: 196.0247.

Compound S2 (800 mg, 4.06 mmol), HATU (2.30 g, 6.09 mmol) and propylamine (1.3 mL, 16.2 mmol) in anhydrous DMF (10 mL) were added DIPEA (2.0 mL, 12.2 mmol) dropwise at 0 ^{o}C, and then the mixture was stirred at room temperature overnight. The mixture was added H_2O and extracted with ethyl acetate 3 times. The organic layer was washed with saturated $NaHCO_3$, 0.1 M HCl, H_2O, brine, dried over Na_2SO_4 and concentrated in vacuo. The residue was purified by silica chromatography to afford **compound S1** as a brown oil (513 mg, 53%). ^{1}H NMR (500 MHz, MeOD) δ 8.27 (d, *J* = 1.8 Hz, 1H), 8.10 (d, *J* = 8.4 Hz, 1H), 7.86 (dd, *J* = 8.4, 2.0 Hz, 1H), 4.96 (s, 2H), 3.38 – 3.34 (m, 2H), 1.65 (dt, *J* = 14.7, 7.3 Hz, 2H), 0.99 (t, *J* = 7.4 Hz, 3H). ^{13}C NMR (126 MHz, MeOD) δ 168.4, 150.1, 140.5, 139.5, 128.7, 127.7, 125.8, 61.7, 43.0, 23.6, 11.8. HRMS (ESI-Q-TOF): m/z $[M+H]^+$ Calcd for $C_{11}H_{15}N_2O_4^+$: 239.1026; found: 239.1028.

6.3 Synthesis of Compound S15, S16 and S18

Compound S18 was synthesized according to published literature.[10] ^{1}H NMR (500 MHz, MeOD) δ 8.46 (s, 1H), 7.54 (t, *J* = 7.8 Hz, 1H), 7.47 (d, *J* = 7.4 Hz, 1H), 7.27 (d, *J* = 8.0 Hz, 1H), 5.18 (dd, *J* = 13.3, 5.1 Hz, 1H), 4.58 – 4.47 (m, 2H), 4.44 – 4.37 (m, 2H), 3.49 – 3.42 (m, 2H), 2.94 (ddd, *J* = 18.7, 13.5, 5.4 Hz, 1H), 2.81 (ddd, *J* = 17.6, 4.4, 2.3 Hz, 1H), 2.48 (qd, *J* = 13.3, 4.6 Hz, 1H), 2.26 – 2.17 (m, 1H). ^{13}C NMR (126 MHz, MeOD) δ 173.3, 170.8, 169.9, 153.2, 133.1, 130.4, 129.9, 116.0, 114.4, 64.4, 52.3, 45.6, 38.7, 31.0, 22.8. LRMS (ESI-SQD2): m/z $[M+H]^+$ Calcd for $C_{15}H_{18}N_3O_4^+$: 304.13; found: 304.25.

Compound S15 was synthesized according to published literature.[11] ^{1}H NMR (600 MHz, DMSO) δ 11.00 (s, 1H), 8.40 (s, 3H), 7.79 (d, *J* = 7.8 Hz, 1H), 7.71 (s, 1H), 7.62 (d, *J* = 7.9 Hz, 1H), 5.13 (dd, *J* = 13.3, 5.1 Hz, 1H), 4.48 (d, *J* = 17.4 Hz, 1H), 4.35 (d, *J* = 17.4 Hz, 1H), 4.16 (q, *J* = 5.8 Hz, 2H), 2.92 (ddd, *J* = 17.4, 13.7, 5.4 Hz, 1H), 2.61 (dd, *J* = 10.2, 6.3 Hz, 1H), 2.46 – 2.37 (m, 1H), 2.07 – 1.94 (m, 1H).

Compound S16 was synthesized according to published literature.[12] ^{1}H NMR (600 MHz, DMSO) δ 8.18 (d, *J* = 3.1 Hz, 1H), 7.80 (dd, *J* = 8.3, 1.5 Hz, 2H), 7.60 (d, *J* = 7.8 Hz, 2H), 6.78 (d, *J* = 7.8 Hz, 1H), 6.72 (t, *J* = 8.0 Hz, 1H), 4.07 (d, *J* = 5.4 Hz, 2H), 2.55 (s, 3H).

6.4 Synthesis of o-NBA-biotin

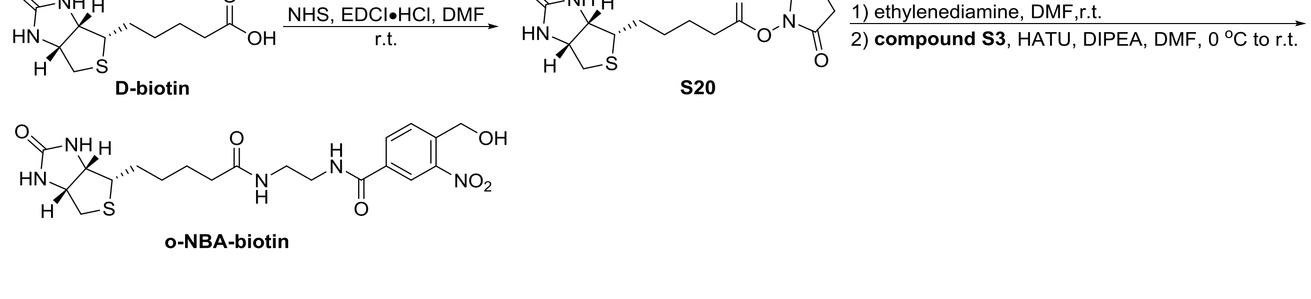

D-biotin (3.00 g, 12.3 mmol), EDCI•HCl (2.82 g, 14.7 mmol) and NHS (1.70 g, 14.7 mmol) in DMF (100 mL) was stirred at room temperature overnight. The reaction mixture was concentrated in vacuo and the residue was filtered, washed with EtOH/AcOH/H_2O (95:1:4) and dried in vacuo. The product **compound S20** was obtained without further purification as a yellow oil (3.74 g, 89%). ^{1}H NMR (500 MHz, DMSO) δ 6.45 (s, 1H), 6.38 (s, 1H), 4.30 (dd, *J* = 7.5, 5.2 Hz, 1H), 4.16 – 4.12 (m, 1H), 3.13 – 3.07 (m, 1H), 2.84 (d, *J* = 5.1 Hz, 1H), 2.81 (s, 4H), 2.67 (t, *J* = 7.4 Hz, 2H), 2.58 (d, *J* = 13.3 Hz, 1H), 1.74 – 1.57 (m, 6H).

To a stirred solution of ethylenediamine (3.91 mL, 58.6 mM) in anhydrous DMF (20 mL) was added **compound S20** (1.00 g, 2.93 mmol) in anhydrous DMF (10 mL) dropwise and stirred overnight. The mixture was added Et_2O. The precipitated product was filtered, washed with ethyl acetate and dried in vacuo without further purification (753 mg, 90%). The product from previous step (400 mg, 1.40 mmol), **compound S3** (276 mg, 1.40 mmol) and HATU (637 mg, 1.68 mmol) in anhydrous DMF (15 mL) was added DIPEA (0.69 mL, 4.19 mmol) dropwise at 0 ^{o}C, and then the mixture was stirred at room temperature overnight. The mixture was added H_2O and extracted with ethyl acetate 3 times. The organic layer was washed with saturated $NaHCO_3$, 0.1 M HCl, H_2O, brine, dried over Na_2SO_4 and concentrated in vacuo. The residue was purified by silica chromatography and **o-NBA-biotin** was obtained as a yellow oil (331 mg, 51%). ^{1}H NMR (500 MHz, MeOD) δ 8.52 (d, *J* = 1.7 Hz, 1H), 8.15 (dd, *J* = 8.1, 1.7 Hz, 1H), 7.99 (d, *J* = 8.2 Hz, 1H), 5.00 (s, 2H), 4.46 (dd, *J* = 7.9, 4.9 Hz, 1H), 4.24 (dd, *J* = 7.9, 4.5 Hz, 1H), 3.53 (t, *J* = 5.9 Hz, 2H), 3.44 (t, *J* = 5.8 Hz, 2H), 3.14 – 3.09 (m, 1H), 2.89 (dd, *J* = 12.8, 5.0 Hz, 1H), 2.68 (d, *J* = 12.7 Hz, 1H), 2.21 (t, *J* = 7.4 Hz, 2H), 1.66 (m, 4H), 1.59 – 1.49 (m, 2H). ^{13}C NMR (126 MHz, MeOD) δ 176.7, 167.8, 166.1, 148.5, 142.6, 135.5, 133.0, 129.9, 124.6, 63.3, 61.8, 61.6, 56.9, 41.1, 41.0, 39.9, 36.8, 29.7, 29.4, 26.8. HRMS (ESI-Q-TOF): m/z $[M+H]^+$ Calcd for $C_{20}H_{28}N_5O_6S^+$: 466.1755; found: 466.1744.

6.5 Synthesis of Rho-o-NBA

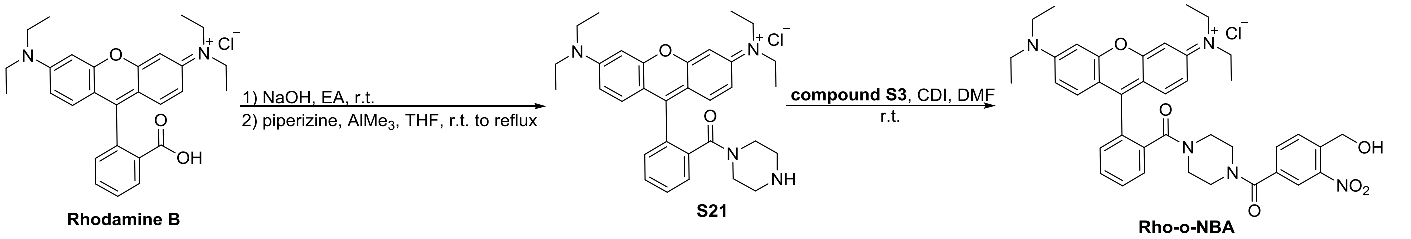

Rhodamine B (5.00 g, 10.4 mmol) was dispersed in 1 M NaOH (100 mL) and ethyl acetate (100 mL), and then the aqueous layer was extracted with ethyl acetate twice. The organic layer was washed with brine, dried over Na_2SO_4 and concentrated in vacuo. The product Rhodamine B base was obtained without further purification as a red oil (4.89 g, 86%). Piperazine (1.89 g, 21.92 mmol) in anhydrous DCM was added $AlMe_3$ in toluene (2.0 M, 5.48 mL, 10.96 mmol) at room temperature. After stirring for 1 h, white precipitate appeared and Rhodamine B base (3.00 g, 5.48 mmol) in anhydrous DCM was added dropwise at room temperature. The reaction mixture was refluxed overnight. After cooled down to room temperature, the mixture was added 0.1 M HCl until no more bubbles were observed. The mixture was filtered and washed with DCM and DCM/MeOH (4:1). The filtrate was concentrated and dissolved in DCM, filtered to remove undissolved salt and concentrated again. The residue was dispersed in $NaHCO_3$ (0.02%) and ethyl acetate, and the aqueous layer was washed with ethyl acetate 5 times to remove materials. The aqueous layer was saturated with NaCl, acidified with 1 M HCl and extracted with ^{i}PrOH/DCM (4:1) 3 times. The organic layer was dried over Na_2SO_4 and concentrated. The residue was purified by silica chromatography to afford **compound S21** as a pink oil (2.11 g, 53%). ^{1}H NMR (400 MHz, MeOD) δ 7.83 – 7.74 (m, 3H), 7.53 (dd, *J* = 5.8, 2.9 Hz, 1H), 7.27 (d, *J* = 9.5 Hz, 2H), 7.09 (dd, *J* = 9.5, 2.4 Hz, 2H), 6.98 (d, *J* = 2.4 Hz, 2H), 3.74 – 3.66 (m, 12H), 3.13 (d, *J* = 4.5 Hz, 4H), 1.36 – 1.28 (m, 12H).

Compound S21 (132 mg, 0.241 mmol) in anhydrous DMF (2.5 mL) was added CDI (41 mg, 0.253 mmol) and stirred for 1 h. **Compound S3** (47 mg, 0.241 mmol) was added and stirred overnight. The solvent was removed in vacuo and the

residue was purified by silica chromatography to afford **Rho-o-NBA** as a pink oil (59 mg, 34%).^{1}H NMR (400 MHz, MeOD) δ 8.07 (d, *J* = 1.5 Hz, 1H), 7.96 (d, *J* = 8.0 Hz, 1H), 7.77 (s, 2H), 7.73 (dd, *J* = 8.0, 1.6 Hz, 2H), 7.52 (d, *J* = 4.9 Hz, 1H), 7.28 (d, *J* = 8.7 Hz, 2H), 7.07 (d, *J* = 8.8 Hz, 2H), 6.98 (d, *J* = 2.4 Hz, 2H), 4.96 (s, 2H), 3.70 (q, *J* = 7.1 Hz, 8H), 3.49 (s, 8H), 1.31 (t, *J* = 7.1 Hz, 12H). ^{13}C NMR (126 MHz, MeOD) δ 170.0, 169.7, 159.3, 157.3, 156.9, 148.4, 141.4, 136.5, 136.0, 133.2, 133.1, 132.2, 131.8, 131.4, 131.3, 130.0 , 128.9, 124.5, 115.4, 114.9, 97.4, 61.7, 46.9, 12.8. HRMS (ESI-Q-TOF): m/z [M-Cl]$^+$ Calcd for $C_{40}H_{44}N_5O_6^+$: 690.3286; found: 690.3274.

6.6 Synthesis of compound S17

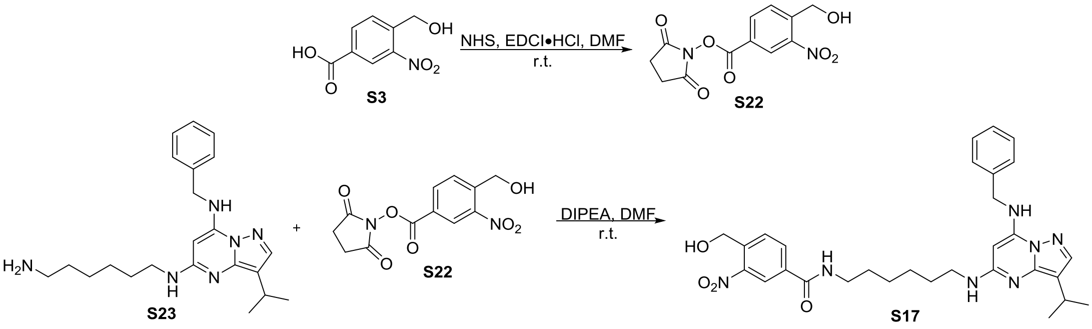

Compound S23 was synthesized according to published literature.[13]

Compound S3 (1.00 g, 5.07 mmol) and NHS (702 mg, 6.08 mmol) in DMF (15 mL) was added EDCI•HCl (1.16 g, 6.08 mmol). The reaction mixture was stirred at room temperature for 2 h. The mixture was added H_2O and extracted with ethyl acetate 3 times. The organic layer was washed with 2.8% $KHSO_4$, H_2O, brine, dried over Na_2SO_4 and concentrated in vacuo. The residue was purified by silica chromatography to afford product **compound S22** as a yellow oil (710 mg, 48%).

Compound S23 (80 mg, 0.210 mmol), **S26** (62 mg, 0.210 mmol) were dissolved in 4 ml DMF, added with DIPEA (8 μL, 0.105 mmol). The mixture was stirred at room temperature for 40 min. Then the solvent was evaporated in vacuo, the yellow product **compound S17** was obtained by column chromatography as a yellow oil (70 mg, 60 %). ^{1}H NMR (400 MHz, MeOD) δ 8.52 (d, *J* = 1.8 Hz, 1H), 8.13 (dd, *J* = 8.1, 1.8 Hz, 1H), 7.95 (d, *J* = 8.2 Hz, 1H), 7.64 (s, 1H), 7.38 (d, *J* = 7.1 Hz, 2H), 7.36 – 7.30 (m, 2H), 7.28 – 7.22 (m, 1H), 5.00 (s, 2H), 4.87 (s, 1H), 4.53 (s, 2H), 3.40 (t, *J* = 7.1 Hz, 2H), 3.30 (t, *J* = 7.0 Hz, 2H), 3.12 – 3.03 (m, 1H), 1.67 – 1.55 (m, 4H), 1.45 – 1.40 (m, 4H), 1.29 (d, *J* = 6.9 Hz, 6H). ^{13}C NMR (126 MHz, DMSO) δ 163.8, 156.4, 146.5, 146.1, 141.2, 139.6, 138.6, 134.1, 132.1, 128.4, 127.0, 126.8, 123.0, 110.5, 72.4, 59.9, 44.6, 29.0, 26.3, 23.4, 23.1. HRMS (ESI-SQD2): m/z [M+H]$^+$ Calcd for $C_{30}H_{38}N_7O_4^+$: 560.2980; found: 560.2971.

6.7 Synthesis of compound 29 and 30

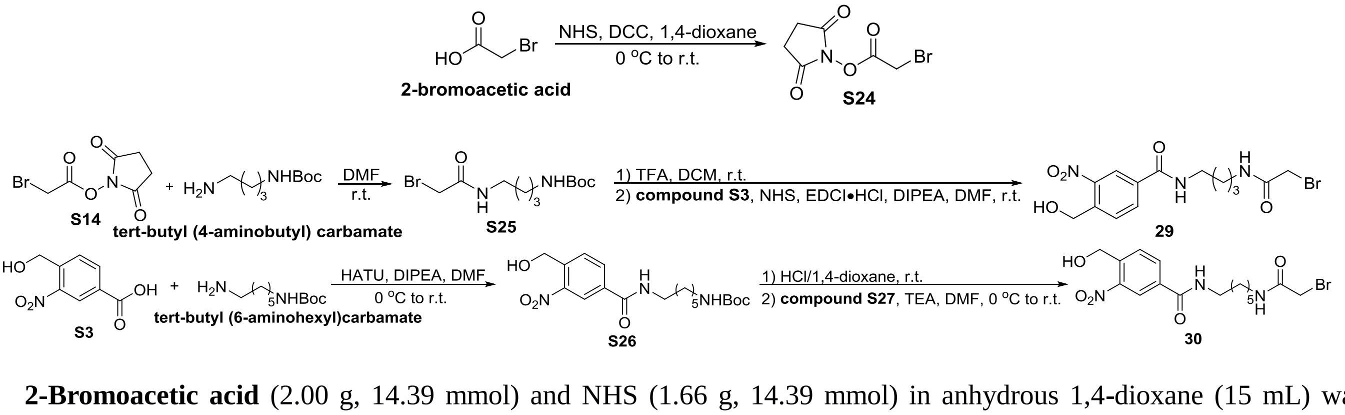

2-Bromoacetic acid (2.00 g, 14.39 mmol) and NHS (1.66 g, 14.39 mmol) in anhydrous 1,4-dioxane (15 mL) was added DCC (3.27 g, 15.83 mmol) in anhydrous 1,4-dioxane (5 mL) dropwise at 0 °C. The reaction mixture was stirred at room temperature for 2 h and filtered. The filtrate was concentrated in vacuo to afford **compound S24** as a yellow oil (2.43 g, 77%).^{1}H NMR (400 MHz, DMSO) δ 4.64 (s, 2H), 2.83 (s, 4H).

Compound S24 (1.18 g, 5.00 mmol) and **tert-butyl (4-aminobutyl) carbamate** (1.13 g, 5.00 mmol) in DMF (20 mL) was stirred at room temperature overnight. The reaction mixture was concentrated in vacuo and purified by silica chromatography to afford **compound S25** as a yellow oil (760 mg, 49%). ^{1}H NMR (500 MHz, $CDCl_3$) δ 6.64 (s, 1H), 4.60 (s, 1H), 3.87 (s, 2H), 3.31 (dd, *J* = 12.9, 6.7 Hz, 2H), 3.23 – 3.07 (m, 2H), 1.62 – 1.52 (m, 4H), 1.44 (s, 9H).

Compound S25 (300 mg, 0.97 mmol) in DCM (10 mL) was added TFA (2.5 mL) and stirred at room temperature for 1 h. The reaction mixture was concentrated in vacuo to afford product for next step without further purification. **Compound S3** (211 mg, 1.07 mmol) in DMF (10 mL) was added NHS (160 mg, 1.39 mmol) and EDCI•HCl (410 mg, 2.14 mmol) and stirred overnight. The reaction mixture was stirred at room temperature for 30 min and the product from previous step (compound 13-De-Boc product) and DIPEA (0.83 mL, 5.00 mmol) was added and stirred for 4 h. The reaction mixture was added H_2O and ethyl acetate, and the aqueous layer was extracted with ethyl acetate 3 times. The combined organic layer was washed with brine, dried over Na_2SO_4, filtered and concentrated in vacuo. The residue was purified by silica chromatography to afford **compound 25** as a yellow oil (162 mg, 43% for 2 steps). ^{1}H NMR (500 MHz, MeOD) δ 8.52 (d, *J* = 1.8 Hz, 1H), 8.15 (dd, *J* = 8.1, 1.8 Hz, 1H), 7.98 (d, *J* = 8.1 Hz, 1H), 4.99 (s, 2H), 3.81 (s, 2H), 3.43 (t, *J* = 6.8 Hz, 2H), 3.26 (t, *J* = 6.8 Hz, 2H), 1.64 (m, 4H). ^{13}C NMR (126 MHz, MeOD) δ 169.5, 167.6, 148.4, 142.5, 135.6, 133.0, 129.8, 124.5, 61.8, 40.7, 40.6, 28.8, 27.7, 27.6. HRMS (ESI-Q-TOF): m/z $[M+H]^+$ Calcd for $C_{14}H_{19}BrN_3O_5^+$: 388.0503; found: 388.0511.

Compound S3 (400 mg, 2.03 mmol), HATU (849 mg, 2.23 mmol) and tert-butyl (6-aminohexyl) carbamate (483 mg, 2.23 mmol) in anhydrous DMF (10 mL) were added DIPEA (1 mL, 6.09 mmol) dropwise at 0 ^{o}C, and then the mixture was stirred at room temperature overnight. The mixture was added H_2O and extracted with ethyl acetate 3 times. The organic layer was washed with H_2O, brine, dried over Na_2SO_4 and concentrated in vacuo. The residue was purified by silica chromatography to afford **compound S26** as a yellow oil (670 mg, 84%). ^{1}H NMR (400 MHz, DMSO) δ 8.75 (t, *J* = 5.5 Hz, 1H), 8.50 (d, *J* = 1.7 Hz, 1H), 8.21 (dd, *J* = 8.1, 1.6 Hz, 1H), 7.92 (d, *J* = 8.1 Hz, 1H), 6.77 (t, *J* = 5.4 Hz, 1H), 5.66 (t, *J* = 5.5 Hz, 1H), 4.87 (d, *J* = 5.4 Hz, 2H), 3.27 (dd, *J* = 12.9, 6.7 Hz, 2H), 2.89 (q, *J* = 6.6 Hz, 2H), 1.56 – 1.48 (m, 2H), 1.36 (s, 9H), 1.35 – 1.17 (m, 6H).

Compound S26 (102 mg ,0.30 mmol) was dissolved in 1,4-dioxane (5 mL) and was added 4 M HCl/1,4-dioxane (4 mL). The reaction mixture was stirred at room temperature for 6 h and concentrated to afford product for next step without further purification. The product was dissolved in anhydrous DMF (5 mL), added Et_3N (0.13 mL, 0.90 mmol) and then was added **compound S24** (78 mg, 0.332 mmol) in anhydrous DMF (3 mL) at 0 ^{o}C. The reaction mixture was stirred at room temperature for 15 min and added H_2O and ethyl acetate. The organic layer was washed with H_2O and brine, dried over Na_2SO_4 and then concentrated in vacuo. The residue was purified by silica chromatography to afford **compound 30** as a yellow oil (75 mg, 60% for 2 steps). ^{1}H NMR (600 MHz, DMSO) δ 8.75 (t, *J* = 5.5 Hz, 1H), 8.50 (d, *J* = 1.6 Hz, 1H), 8.24 (t, *J* = 5.1 Hz, 1H), 8.21 (dd, *J* = 8.1, 1.6 Hz, 1H), 7.92 (d, *J* = 8.1 Hz, 1H), 4.87 (s, 2H), 3.81 (s, 2H), 3.27 (dd, *J* = 13.0, 6.8 Hz, 2H), 3.06 (dd, *J* = 12.9, 6.8 Hz, 2H), 1.56 – 1.50 (m, 2H), 1.44 – 1.39 (m, 2H), 1.33 – 1.28 (m, 4H). ^{13}C NMR (151 MHz, DMSO) δ 165.8, 163.9, 146.6, 141.2, 134.1, 132.1, 128.4, 123.0, 59.9, 39.4, 38.9, 29.6, 28.9, 28.8, 26.2, 26.0. HRMS (ESI-Q-TOF): m/z $[M+H]^+$ Calcd for $C_{16}H_{23}BrN_3O_5^+$: 416.0816; found: 416.0819.

6.8 Synthesis of compound 36

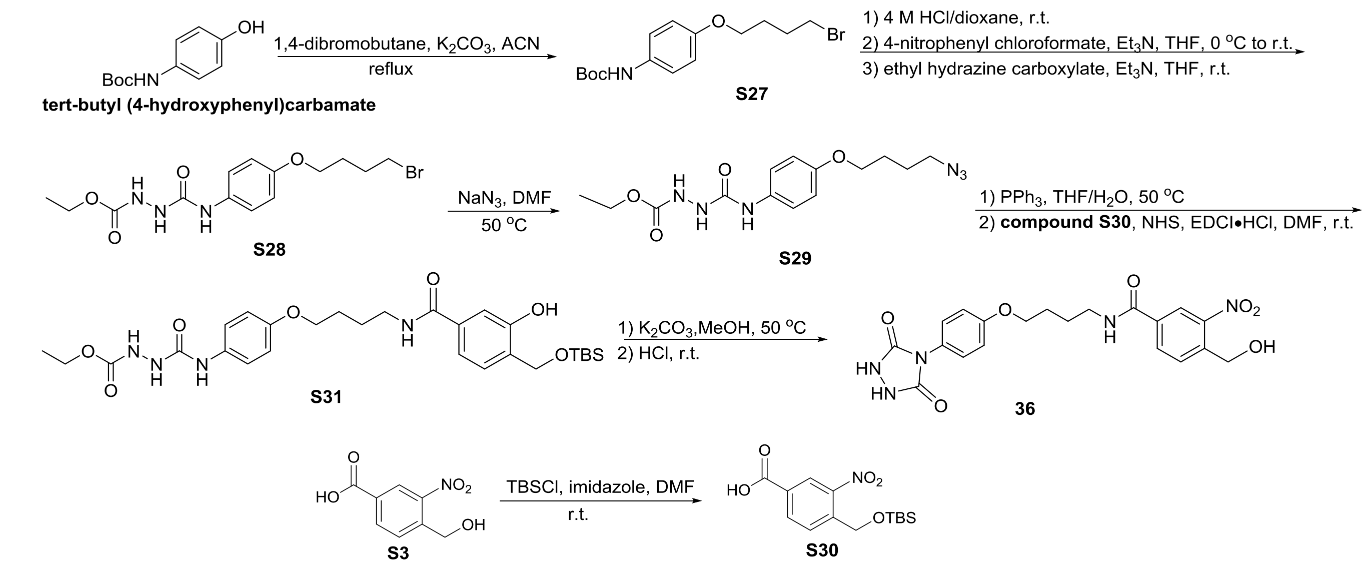

tert-butyl (4-hydroxyphenyl) carbamate (3.00 g,14.34 mmol) and 1,4-dibromobutane (9.29 g, 43.0 mmol) in ACN (80 mL) were added K_2CO_3 (2.97 g, 21.5 mmol) and refluxed for 12 h. The reaction mixture was filtered, concentrated and purified by silica chromatography to afford **compound S27** as a yellow oil (2.62 g, 53%).^{1}H NMR (400 MHz, DMSO) δ 9.11 (s, 1H), 7.32 (d, J = 9.0 Hz, 2H), 6.82 (d, J = 9.0 Hz, 2H) , 3.93 (t, J = 6.3 Hz, 2H), 3.60 (t, J = 6.7 Hz, 2H), 2.00 – 1.90 (m, 2H), 1.85 – 1.75 (m, 2H), 1.45 (s, 9H).

Compound S27 (1.60 g, 4.64 mmol) was dissolved in 4 M HCl/dioxane and stirred at room temperature for 2 h. The mixture was concentrated in vacuo. The residue was dissolved in anhydrous THF (70 mL), added Et_3N (1.93 ml, 13.9 mmol) and 4-nitrophenyl chloroformate (1.50 g, 7.42 mmol) at 0 ^{o}C and stirred at room temperature overnight. The mixture was added ethyl hydrazine carboxylate (772 mg, 7.42 mmol) and Et_3N (1.0 ml, 7.42 mmol) and stirred for 3 h. The mixture was added 1 M HCl, H_2O and ethyl acetate. The aqueous layer was extracted with ethyl acetate 3 times and the combined organic layer was washed with brine, dried over Na_2SO_4, filtered and concentrated in vacuo. The residue was purified by silica chromatography to afford **compound S28** as a brown oil (1.25 g, 72%).^{1}H NMR (400 MHz, DMSO) δ 8.88 (s, 1H), 8.53 (s, 1H), 7.90 (s, 1H), 7.34 (d, J = 8.9 Hz, 2H), 6.83 (d, J = 9.0 Hz, 2H), 4.05 (q, J = 7.1 Hz, 2H), 3.94 (t, J = 6.3 Hz, 2H), 3.60 (t, J = 6.7 Hz, 2H), 2.06 – 1.90 (m, 2H), 1.87 – 1.74 (m, 2H), 1.19 (t, J = 6.8 Hz, 3H).

Compound S28 (400 mg, 1.07 mmol) in anhydrous DMF (10 mL) was added NaN_3 (140 mg, 2.14 mmol). The reaction mixture was stirred at 50 ^{o}C for 3 h and then was added H_2O and ethyl acetate. The aqueous layer was extracted with ethyl acetate 3 times and the combined organic layer was washed with brine, dried over Na_2SO_4, filtered and concentrated in vacuo to afford product **compound S29** as a brown oil (341 mg, 95%).^{1}H NMR (400 MHz, DMSO) δ 8.88 (s, 1H), 8.53 (s, 1H), 7.90 (s, 1H), 7.33 (d, J = 8.8 Hz, 2H), 6.83 (d, J = 8.9 Hz, 2H), 4.05 (q, J = 7.1 Hz, 2H), 3.93 (t, J = 6.0 Hz, 2H), 3.40 (t, J = 6.7 Hz, 2H), 1.79 – 1.62 (m, 4H), 1.19 (t, J = 6.7 Hz, 3H).

Compound S3 (2.50 g, 12.7 mmol) in anhydrous DMF (15 mL) was added TBSCl (3.82 g, 25.4 mmol) and imidazole (1.73 g, 25.4 mmol) and stirred at room temperature overnight. The reaction mixture was added 10% Na_2CO_3 solution, and then pH was adjusted to 5-6 with 1 M HCl at 0 ^{o}C. The aqueous layer was extracted with ethyl acetate 3 times, and the organic layer was washed with 1 M HCl twice, dried over Na_2SO_4, filtered and concentrated in vacuo. The residue was purified by silica chromatography to afford **compound S30** as a brown oil (3.40 g, 86%).^{1}H NMR (400 MHz, DMSO) δ 13.60 (s, 1H), 8.49 (s, 1H), 8.29 (d, J = 8.1 Hz, 1H), 7.93 (d, J = 8.1 Hz, 1H), 5.10 (s, 2H), 0.92 (s, 9H), 0.11 (s, 6H).

Compound S29 (300 mg, 0.89 mmol) in anhydrous THF (10 mL) was added PPh_3 (303 mg, 1.16 mmol) and stirred for 3 h. The reaction mixture was added H_2O (2 mL) and stirred at 50 ^{o}C for 12 h. The reaction mixture was concentrated to afford crude product without further purification. **Compound S30** (277 mg, 0.89 mmol) in anhydrous DMF (10 mL) was

added NHS (134 mg, 1.16 mmol) and EDCI•HCl (341 mg, 1.78 mmol) and stirred for 3 h, and then compound 8-reduction product was added and stirred for 4 h. The reaction mixture was added H_2O and ethyl acetate, and the aqueous layer was extracted with ethyl acetate 3 times. The combined organic layer was washed with brine, dried over Na_2SO_4, filtered and concentrated in vacuo. The residue was purified by silica chromatography to afford **compound S31** as a yellow oil (322 mg, 60%). ^{1}H NMR (400 MHz, DMSO) δ 8.88 (s, 1H), 8.81 (t, *J* = 5.3 Hz, 1H), 8.52 (s, 2H), 8.23 (d, *J* = 8.1 Hz, 1H), 7.88 (d, *J* = 8.2 Hz, 2H), 7.33 (d, *J* = 8.8 Hz, 2H), 6.82 (d, *J* = 8.9 Hz, 2H), 5.08 (s, 2H), 4.05 (q, *J* = 7.1 Hz, 2H), 3.94 (t, *J* = 5.9 Hz, 2H), 3.39 – 3.31 (m, 2H), 1.85 – 1.62 (m, 4H), 1.19 (t, *J* = 6.7 Hz, 3H), 0.91 (s, 9H), 0.11 (s, 6H).

Compound S31 (200 mg, 0.35 mmol) in MeOH was added K_2CO_3 and stirred at 50 ^oC for 12 h. The pH of reaction was adjusted to 2, stirred at room temperature for 1 h and then concentrated in vacuo. The residue was purified by silica chromatography to afford **compound 36** as a yellow oil (65 mg, 42%).[4] ^{1}H NMR (500 MHz, DMSO) δ 10.32 (s, 2H), 8.82 (t, *J* = 5.5 Hz, 1H), 8.52 (d, *J* = 1.7 Hz, 1H), 8.23 (dd, *J* = 8.1, 1.6 Hz, 1H), 7.93 (d, *J* = 8.1 Hz, 1H), 7.31 (d, *J* = 8.9 Hz, 2H), 7.01 (d, *J* = 9.0 Hz, 2H), 5.65 (s, 1H), 4.87 (s, 2H), 4.04 (t, *J* = 6.3 Hz, 2H), 3.37 (d, *J* = 6.8 Hz, 2H), 1.83 – 1.75 (m, 2H), 1.71 (dt, *J* = 13.7, 6.7 Hz, 2H). ^{13}C NMR (126 MHz, DMSO) δ 164.0, 157.8, 153.8, 146.6, 141.2 , 134.0, 132.1, 128.5, 127.6, 124.5, 123.1, 114.6, 67.4, 59.9, 29.0, 26.2, 25.6. HRMS (ESI-Q-TOF): m/z [M+H]$^+$ Calcd for $C_{20}H_{22}N_5O_7^+$: 444.1514; found: 444.1516.

6.9 Synthesis of Probe-1

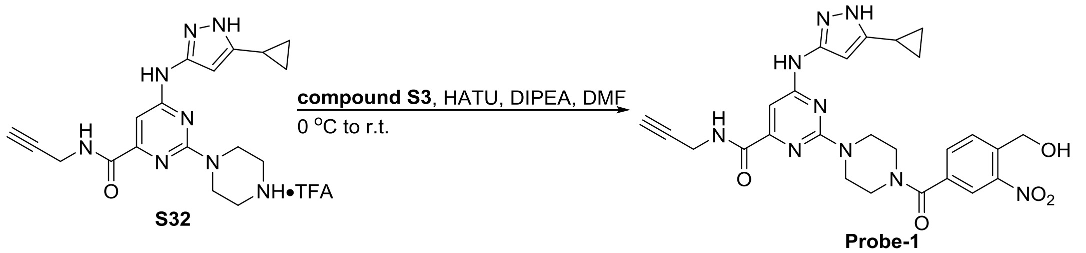

Compound S32 was synthesized according to published literature.[14]

Compound S32 (673 mg, 1.40 mmol), **compound S3** (276 mg, 1.40 mmol) and HATU (637 mg, 1.68 mmol) in anhydrous DMF (15 mL) was added DIPEA (0.69 mL, 4.19 mmol) dropwise at 0 ^oC, and then the mixture was stirred at room temperature overnight. The mixture was added H_2O and extracted with ethyl acetate 3 times. The organic layer was washed with saturated $NaHCO_3$, 0.1 M HCl, H_2O, brine, dried over Na_2SO_4 and concentrated in vacuo. The residue was purified by silica chromatography and **Probe-1** was obtained as a yellow oil (405 mg, 53%). ^{1}H NMR (500 MHz, DMSO) δ 12.03 (s, 1H), 9.81 (s, 1H), 8.93 (s, 1H), 8.12 (d, *J* = 1.5 Hz, 1H), 7.93 (d, *J* = 8.0 Hz, 1H), 7.87 (dd, *J* = 8.0, 1.4 Hz, 1H), 6.82 (s, 1H), 6.15 (s, 1H), 4.87 (s, 2H), 4.01 (dd, *J* = 5.8, 1.9 Hz, 2H), 3.89 (m, 4H), 3.39 (m, 4H), 3.08 (t, *J* = 2.4 Hz, 1H), 1.92 – 1.84 (m, 1H), 0.90 (d, *J* = 7.0 Hz, 2H), 0.67 (d, *J* = 4.7 Hz, 2H). ^{13}C NMR (126 MHz, DMSO) δ 167.0, 163.6, 160.7, 146.7, 139.6, 135.4, 132.0, 128.6, 123.2, 92.9, 81.2, 72.6, 59.8, 48.6, 28.1, 7.8, 6.9. HRMS (ESI-Q-TOF): m/z [M+H]$^+$ Calcd for $C_{26}H_{28}N_9O_5^+$: 546.2208; found: 546.2202.

6.10 Indazolone products

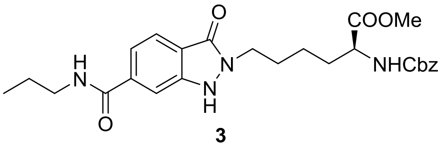

3

^{1}H NMR (600 MHz, MeOD) δ 7.81 (d, *J* = 8.3 Hz, 1H), 7.73 (s, 1H), 7.54 (dd, *J* = 8.2, 1.1 Hz, 1H), 7.34 – 7.26 (m, 5H), 5.07 – 5.00 (m, 2H), 4.16 (dd, *J* = 9.4, 4.8 Hz, 1H), 3.99 (tt, *J* = 14.2, 7.3 Hz, 2H), 3.66 (s, 3H), 3.36 (t, *J* = 7.2 Hz, 2H), 1.91 – 1.78 (m, 3H), 1.73 (dd, *J* = 14.3, 6.2 Hz, 1H), 1.66 (dd, *J* = 14.5, 7.3 Hz, 2H),

1.45 – 1.38 (m, 2H), 0.99 (t, *J* = 7.4 Hz, 3H). ^{13}C NMR (126 MHz, MeOD) δ 174.5, 169.5, 162.1, 158.7, 146.4, 139.5, 138.2, 129.4, 129.0, 128.7, 124.3, 120.9, 119.8, 112.4, 67.6, 55.2, 52.6, 44.7, 43.0, 31.9, 28.7, 23.8, 23.7, 11.8. HRMS (ESI-Q-TOF): m/z $[M+H]^+$ Calcd for $C_{26}H_{33}N_4O_6^+$: 497.2395; found: 497.2407.

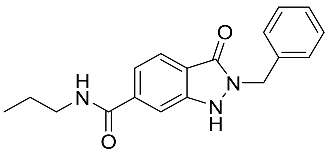

5

^{1}H NMR (400 MHz, DMSO) δ 10.46 (s, 1H), 8.56 (t, *J* = 5.5 Hz, 1H), 7.74 (d, *J* = 8.2 Hz, 1H), 7.64 (s, 1H), 7.53 (dd, *J* = 8.2, 0.9 Hz, 1H), 7.30 (ddd, *J* = 15.3, 13.4, 6.9 Hz, 5H), 5.03 (s, 2H), 3.22 (dd, *J* = 13.2, 6.6 Hz, 2H), 1.59 – 1.48 (m, 2H), 0.89 (t, *J* = 7.4 Hz, 3H). ^{13}C NMR (101 MHz, DMSO) δ 166.0, 160.3, 145.6, 138.0, 136.9, 128.7, 127.7, 123.2, 119.7, 118.7, 111.4, 47.0, 41.3, 22.5, 11.6. HRMS (ESI-Q-TOF): m/z $[M-H]^-$ Calcd for $C_{18}H_{18}N_3O_2^-$: 308.1405; found: 308.1411.

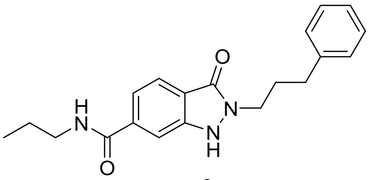

6

^{1}H NMR (500 MHz, DMSO) δ 10.45 (s, 1H), 8.60 (t, *J* = 5.5 Hz, 1H), 7.70 (d, *J* = 8.2 Hz, 2H), 7.54 (d, *J* = 9.1 Hz, 1H), 7.28 (t, *J* = 7.4 Hz, 2H), 7.23 (d, *J* = 7.0 Hz, 2H), 7.18 (t, *J* = 7.1 Hz, 1H), 3.84 (t, *J* = 7.0 Hz, 2H), 3.23 (dd, *J* = 13.1, 6.6 Hz, 2H), 2.59 (t, *J* = 7.7 Hz, 2H), 2.05 – 1.93 (m, 2H), 1.60 – 1.49 (m, 2H), 0.89 (t, *J* = 7.4 Hz, 3H). ^{13}C NMR (126 MHz, DMSO) δ 165.8, 160.0, 145.4, 141.2, 137.5, 128.3, 125.8, 122.8, 119.5, 111.1, 42.9, 41.1, 32.2, 29.6, 22.3, 11.4. HRMS (ESI-Q-TOF): m/z $[M-H]^-$ Calcd for $C_{20}H_{22}N_3O_2^-$: 336.1718; found: 336.1725.

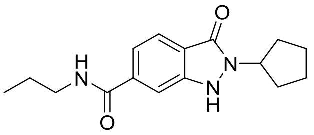

7

^{1}H NMR (500 MHz, MeOD) δ 7.80 (d, *J* = 8.6 Hz, 1H), 7.73 (s, 1H), 7.55 (dd, *J* = 8.3, 1.3 Hz, 1H), 5.00 – 4.93 (m, 1H), 3.36 (t, *J* = 7.2 Hz, 2H), 2.11 (dd, *J* = 15.4, 6.9 Hz, 2H), 1.91 (dd, *J* = 8.1, 4.0 Hz, 4H), 1.78 – 1.72 (m, 2H), 1.66 (dd, *J* = 14.5, 7.3 Hz, 2H), 0.99 (t, *J* = 7.4 Hz, 3H). ^{13}C NMR (126 MHz, MeOD) δ 169.6, 162.3, 146.9, 139.5, 124.2, 121.0, 120.2, 112.7, 56.6, 43.0, 31.3, 25.6, 23.7, 11.8. HRMS (ESI-Q-TOF): m/z $[M+H]^+$ Calcd for $C_{16}H_{22}N_3O_2^+$: 288.1707; found: 288.1709.

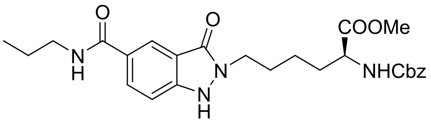

8

^{1}H NMR (600 MHz, MeOD) δ 8.24 (s, 1H), 8.03 (d, *J* = 8.7 Hz, 1H), 7.36 – 7.27 (m, 6H), 5.08 – 5.02 (m, 2H), 4.18 (dd, *J* = 9.1, 4.7 Hz, 1H), 4.05 – 3.95 (m, 2H), 3.68 (s, 3H), 3.37 (t, *J* = 7.1 Hz, 2H), 1.86 (ddd, *J* = 21.0, 13.4, 6.7 Hz, 3H), 1.78 – 1.71 (m, 1H), 1.67 (dd, *J* = 14.5, 7.3 Hz, 2H), 1.44 (dd, *J* = 14.9, 7.5 Hz, 2H), 1.32 (s, 3H), 1.00 (t, *J* = 7.4 Hz, 3H). ^{13}C NMR (151 MHz, MeOD) δ 174.6, 169.5, 162.2, 158.7, 147.7, 138.2, 132.1, 129.4, 129.0, 128.9, 128.7, 123.7, 116.9, 112.6, 67.6, 55.2, 52.6, 44.7, 42.9, 31.9, 28.7, 28.1, 23.8, 11.8. HRMS (ESI-Q-TOF): m/z $[M-H]^-$ Calcd for $C_{26}H_{31}N_4O_6^-$: 495.2249; found: 495.2243.

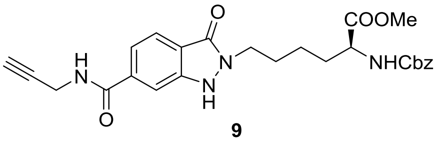

9

^{1}H NMR (500 MHz, DMSO) δ 10.51 (s, 1H), 9.07 (t, *J* = 5.4 Hz, 1H), 7.70 (d, *J* = 8.5 Hz, 3H), 7.54 (d, *J* = 8.1 Hz, 1H), 7.38 – 7.28 (m, 5H), 5.04 – 4.97 (m, 2H), 4.08 (dd, *J* = 5.3, 2.3 Hz, 2H), 4.00 (dd, *J* = 13.0, 8.7 Hz, 1H), 3.81 (s, 2H), 3.59 (s, 3H), 3.14 (t, *J* = 2.3 Hz, 1H), 1.76 – 1.59 (m, 4H), 1.30 (dd, *J* = 14.7, 7.6 Hz, 2H). ^{13}C NMR (126 MHz, DMSO) δ 172.9, 165.6, 159.8, 156.1, 145.1, 136.9, 136.5, 128.4, 127.8, 127.7, 123.0, 119.5, 119.1, 111.3, 81.2, 73.0, 65.5, 53.7, 51.8, 42.9, 30.1, 28.7, 27.3, 22.6. HRMS (ESI-Q-TOF): m/z $[M+H]^+$ Calcd for $C_{26}H_{29}N_4O_6^+$: 493.2082; found: 493.2090.

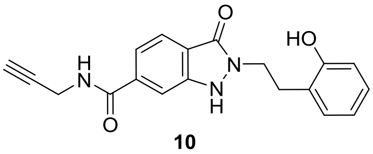

10

^{1}H NMR (500 MHz, DMSO) δ 10.55 (s, 1H), 9.47 (s, 1H), 9.06 (t, *J* =5.5 Hz, 1H), 7.71 (s, 1H), 7.67 (d, *J* = 8.0 Hz, 1H), 7.50 (d, *J* = 7.8 Hz, 1H), 7.02 (dd, *J* = 13.0, 7.2 Hz, 2H), 6.80 (d, *J* = 7.8 Hz, 1H), 6.67 (t, *J* = 7.3 Hz, 1H), 4.09 – 4.04 (m, 3H), 4.04 – 3.97 (m, 2H), 2.98 – 2.91 (m, 2H), 1.99 (dt, *J* = 12.3, 7.0 Hz, 1H). ^{13}C NMR (126 MHz, DMSO) δ 165.6, 159.7, 155.4, 145.2, 136.5, 130.1, 130.0, 127.5, 124.3, 123.0, 119.5, 119.3, 114.9, 111.3, 81.2, 72.9, 43.2, 28.8, 28.6. HRMS (ESI-Q-TOF): m/z $[M+H]^+$ Calcd for $C_{19}H_{18}N_3O_3^+$: 336.1343; found: 336.1337.

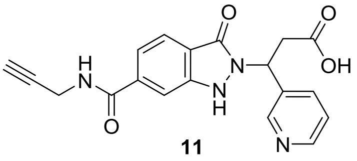

11

^{1}H NMR (500 MHz, DMSO) δ 9.08 (t, *J* = 5.5 Hz, 1H), 8.60 (d, *J* = 2.0 Hz, 1H), 8.49 (dd, *J* = 4.7, 1.3 Hz, 1H), 7.81 – 7.77 (m, 1H), 7.73 (d, *J* = 8.2 Hz, 1H), 7.68 (s, 1H), 7.55

(dd, J = 8.2, 1.1 Hz, 1H), 7.39 (dd, J = 7.9, 4.8 Hz, 1H), 5.93 (t, J = 7.8 Hz, 1H), 4.07 (dd, J = 5.5, 2.4 Hz, 2H), 3.37 – 3.27 (m, 2H), 3.13 (t, J = 2.4 Hz, 1H). ^{13}C NMR (126 MHz, DMSO) δ 171.2, 165.6, 163.1, 149.1, 148.4, 146.1, 137.1, 134.9, 134.7, 123.7, 123.3, 119.9, 118.9, 111.7, 81.2, 73.0, 52.4, 36.6, 28.7. HRMS (ESI-Q-TOF): m/z [M+H]$^+$ Calcd for $C_{19}H_{17}N_4O_4^+$: 365.1244; found: 365.1236.

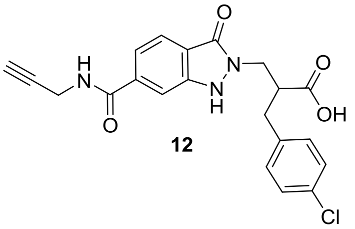
12

^{1}H NMR (500 MHz, DMSO) δ 9.05 (t, J =5.5 Hz, 1H), 7.69 (d, J = 8.4 Hz, 2H), 7.51 (d, J = 8.1 Hz, 1H), 7.30 (d, J = 8.2 Hz, 2H), 7.24 (d, J = 8.2 Hz, 2H), 4.11 – 4.02 (m, 3H), 3.98 (dd, J = 13.9, 6.4 Hz, 2H), 3.12 – 3.11 (m, 1H), 2.85 (dd, J = 13.6, 8.5 Hz, 1H), 2.74 (dd, J = 13.8, 5.3 Hz, 1H). ^{13}C NMR (126 MHz, DMSO) δ 173.8, 165.6, 160.2, 145.5, 137.6, 136.8, 131.0, 130.7, 128.2, 123.1, 119.7, 119.0, 111.4, 81.2, 73.0, 45.6, 44.9, 34.4, 28.7. HRMS (ESI-Q-TOF): m/z [M+H]$^+$ Calcd for $C_{21}H_{19}ClN_3O_4^+$: 412.1059; found: 412.1052.

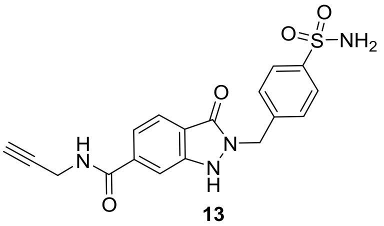
13

^{1}H NMR (500 MHz, DMSO) δ 10.63(s, 1H), 9.02 (t, J =5.5 Hz, 1H), 7.79 (d, J = 8.2 Hz, 2H), 7.73 (d, J = 8.3 Hz, 1H), 7.66 (s, 1H), 7.49 (d, J = 8.0 Hz, 1H), 7.42 (d, J = 8.1 Hz, 2H), 5.11 (s, 2H), 4.06 (dd, J = 5.2, 2.2 Hz, 2H), 3.12 (t, J = 2.4 Hz, 1H). ^{13}C NMR (126 MHz, DMSO) δ 165.6, 160.2, 145.6, 143.4, 140.6, 137.0, 128.0, 126.0, 123.3, 119.8, 118.8, 111.5, 81.1, 73.0, 46.5, 28.6. HRMS (ESI-Q-TOF): m/z [M+H]$^+$ Calcd for $C_{18}H_{17}N_4O_4^+$: 385.0965; found: 385.0959.

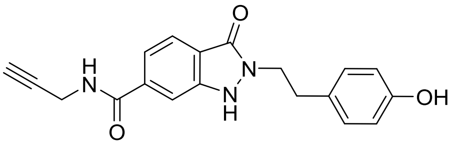
14

^{1}H NMR (600 MHz, DMSO) δ 10.55 (s, 1H), 9.19 (s,1H), 9.06 (t, J =5.5 Hz, 1H), 7.71 (s, 1H), 7.67 (d, J = 8.1 Hz, 1H), 7.52 (d, J = 8.1 Hz, 1H), 7.00 (d, J = 8.3 Hz, 2H), 6.64 (d, J = 8.3 Hz, 2H), 4.07 (s, 2H), 3.97 (t, J = 7.4 Hz, 2H), 3.14 (t, J = 2.4 Hz, 1H), 2.89 (t, J = 7.5 Hz, 2H). ^{13}C NMR (151 MHz, DMSO) δ 165.6, 159.6, 155.8, 145.1, 136.5, 129.5, 128.2, 123.0, 119.5, 119.2, 115.2, 111.2, 81.2, 72.9, 44.9, 32.9, 28.6. HRMS (ESI-Q-TOF): m/z [M+H]$^+$ Calcd for $C_{19}H_{18}N_3O_3^+$: 336.1343; found: 336.1338.

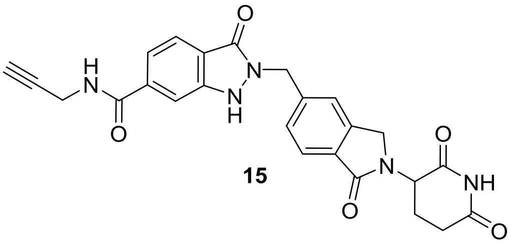
15

^{1}H NMR (500 MHz, DMSO) δ 10.97 (s, 1H), 9.05 (t, J = 5.4 Hz, 1H), 7.75 (dd, J = 21.5, 8.0 Hz, 2H), 7.68 (s, 1H), 7.53 (t, J = 9.1 Hz, 1H), 7.49 (s, 1H), 7.42 (d, J = 7.8 Hz, 1H), 5.16 (d, J = 10.5 Hz, 2H), 5.10 (dd, J = 13.3, 5.1 Hz, 1H), 4.44 (d, J = 17.3 Hz, 1H), 4.31 (d, J = 17.4 Hz, 1H), 4.11 – 4.03 (m, 2H), 3.13 (t, J = 2.4 Hz, 1H), 2.96 – 2.86 (m, 1H), 2.66 – 2.57 (m, 1H), 2.49-2.31 (m, 1H), 2.07 – 1.94 (m, 1H). ^{13}C NMR (126 MHz, DMSO) δ 172.8, 170.9, 167.7, 165.7, 160.0, 145.6, 142.6, 140.8, 132.2, 131.1, 127.5, 124.0, 123.2, 122.6, 119.4, 113.9, 111.6, 81.2, 72.9, 51.6, 47.2, 46.9, 31.2, 28.6, 22.5. HRMS (ESI-Q-TOF): m/z [M+H]$^+$ Calcd for $C_{25}H_{22}N_5O_5^+$: 472.1615; found: 472.1511.

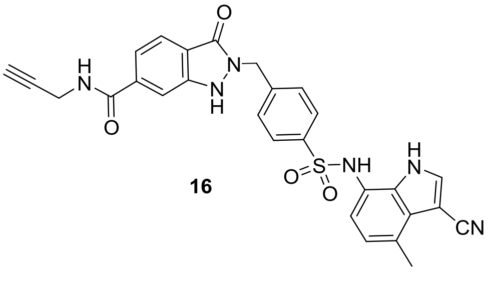
16

^{1}H NMR (500 MHz, DMSO) δ 11.97 (s, 1H), 9.06 (t, J = 5.5 Hz, 1H), 8.15 (s, 1H), 7.76 (d, J = 8.2 Hz, 1H), 7.72 – 7.66 (m, 3H), 7.56 (d, J = 8.2 Hz, 1H), 7.36 (d, J = 8.3 Hz, 2H), 6.74 (d, J = 7.9 Hz, 1H), 6.59 (d, J = 7.7 Hz, 1H), 5.09 (s, 2H), 4.07 (dd, J = 5.3, 2.3 Hz, 2H), 3.13 (t, J = 2.4 Hz, 1H), 2.55 (s, 3H). ^{13}C NMR (126 MHz, DMSO) δ 165.6, 160.1, 145.6, 141.7, 140.1, 138.7, 136.9, 135.2, 130.5, 128.0, 127.3, 127.1, 126.5, 123.3, 122.5, 120.8, 119.7, 118.6, 118.0, 117.4, 111.6, 84.3, 81.2, 73.0, 46.5, 28.7, 17.6. HRMS (ESI-Q-TOF): m/z [M+H]$^+$ Calcd for $C_{28}H_{23}N_6O_4S^+$: 539.1496; found: 539.1490.

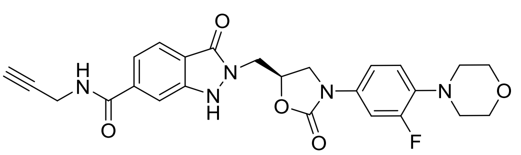
17

^{1}H NMR (500 MHz, DMSO) δ 10.60 (s, 1H), 9.08 (t, J = 5.5 Hz, 1H), 7.72 (d, J = 10.5 Hz, 2H), 7.56 (dd, J = 8.2, 1.1 Hz, 1H), 7.41 (dd, J = 14.9, 2.5 Hz, 1H), 7.14 (dd, J = 8.6, 2.2 Hz, 1H), 7.06 – 7.00 (m, 1H), 5.09 – 5.03 (m,

1H), 4.33 – 4.26 (m, 1H), 4.19 (t, J = 9.0 Hz, 1H), 4.14 – 4.03 (m, 3H), 3.88 (dd, J = 9.2, 5.9 Hz, 1H), 3.76 – 3.68 (m, 4H), 3.13 (t, J = 2.5 Hz, 1H), 2.98 – 2.90 (m, 4H). ^{13}C NMR (126 MHz, DMSO) δ 165.5, 160.8, 155.5, 153.8, 153.5, 145.8, 137.0, 135.6, 135.6, 133.2, 133.1, 123.2, 119.8, 119.2, 118.6, 114.3, 111.5, 106.9, 106.7, 81.1, 72.9, 70.5, 66.1, 50.7, 47.4, 46.5, 28.6. HRMS (ESI-Q-TOF): m/z [M+Na]$^+$ Calcd for $C_{25}H_{24}FN_5NaO_5^+$: 516.1654; found: 516.1645.

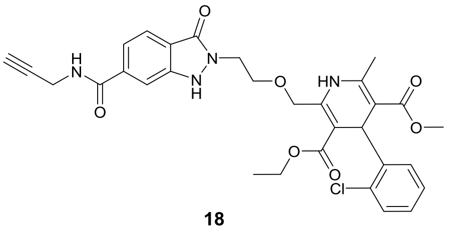

18

^{1}H NMR (500 MHz, DMSO) δ 10.54 (s, 1H), 9.07 (t, J = 5.5 Hz, 1H), 8.38 (s, 1H), 7.74 (d, J = 7.8 Hz, 2H), 7.60 – 7.56 (m, 1H), 7.25 – 7.21 (m, 2H), 7.17 (td, J = 7.6, 1.2 Hz, 1H), 7.12 – 7.07 (m, 1H), 5.25 (s, 1H), 4.59 (dd, J = 46.6, 14.2 Hz, 2H), 4.16 – 4.03 (m, 4H), 3.98 – 3.89 (m, 2H), 3.78 (t, J = 5.6 Hz, 2H), 3.51 (s, 3H), 3.14 (t, J = 2.5 Hz, 1H), 2.25 (s, 3H), 1.08 (t, J = 7.1 Hz, 3H). ^{13}C NMR (126 MHz, DMSO) δ 167.0, 166.2, 165.5, 160.6, 145.8, 145.3, 145.2, 144.6, 136.7, 131.0, 130.9, 128.9, 127.7, 127.4, 123.0, 119.7, 119.0, 111.5, 102.2, 101.7, 81.4, 72.9, 67.8, 66.3, 59.3, 50.5, 43.4, 36.6, 28.6, 18.1, 14.0. HRMS (ESI-Q-TOF): m/z [M+Na]$^+$ Calcd for $C_{31}H_{31}ClN_4NaO_7^+$: 629.1773; found: 629.1779.

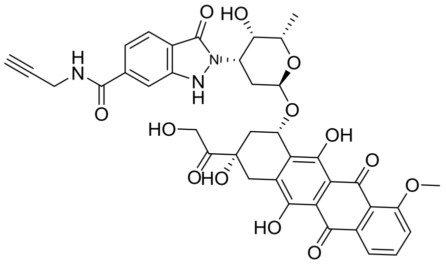

19

^{1}H NMR (500 MHz, DMSO) δ 13.98 (s, 1H), 13.15 (s, 1H), 9.09 (t, J = 5.2 Hz, 1H), 8.10 (s, 1H), 7.93 (s, 1H), 7.87 – 7.80 (m, 2H), 7.70 (d, J = 9.0 Hz, 1H), 7.58 (d, J = 7.2 Hz, 1H), 7.50 (d, J = 8.9 Hz, 1H), 5.57 (s, 1H), 5.45 (s, 1H), 5.34 (d, J = 13.5 Hz, 1H), 5.17 (d, J = 6.6 Hz, 1H), 5.00 (s, 1H), 4.88 (t, J = 5.5 Hz, 1H), 4.63 (d, J = 5.5 Hz, 2H), 4.47 (dd, J = 12.6, 6.1 Hz, 1H), 4.03 (d, J = 3.1 Hz, 2H), 3.92 (s, 3H), 3.83 (d, J = 5.5 Hz, 1H), 3.09 (s, 1H), 3.01 (d, J = 18.2 Hz, 1H), 2.93 – 2.86 (m, 1H), 2.61 (t, J = 11.5 Hz, 1H), 2.30 (d, J = 13.9 Hz, 1H), 2.15 (dd, J = 14.2, 5.2 Hz, 1H), 2.03 – 1.94 (m, 1H), 1.85 (d, J = 8.6 Hz, 1H), 1.19 (d, J = 6.3 Hz, 3H). ^{13}C NMR (126 MHz, DMSO) δ 214.0, 186.4, 186.3, 165.4, 160.7, 156.1, 154.4, 136.2, 135.2, 134.6, 134.1, 131.0, 127.2, 121.5, 121.3, 119.9, 119.7, 118.9, 115.4, 112.3, 110.7, 110.6, 110.0, 99.8, 81.2, 74.8, 72.8, 70.3, 66.6, 66.0, 63.8, 56.5, 52.5, 36.6, 32.0, 28.6, 16.9. HRMS (ESI-Q-TOF): m/z [M-H]$^-$ Calcd for $C_{38}H_{34}N_3O_{13}^-$: 740.2097; found: 740.2087.

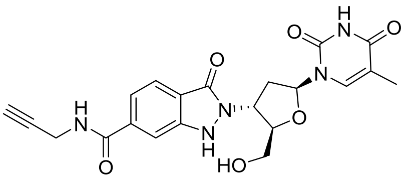

20

^{1}H NMR (500 MHz, MeOD) δ 7.92 (d, J = 1.2 Hz, 1H), 7.84 (dd, J = 8.2, 0.5 Hz, 1H), 7.79 (s, 1H), 7.61 (dd, J = 8.2, 1.3 Hz, 1H), 6.47 (t, J = 6.6 Hz, 1H), 5.35 (dt, J = 9.4, 5.9 Hz, 1H), 4.33 (dt, J = 6.0, 3.1 Hz, 1H), 4.18 (d, J = 2.5 Hz, 2H), 3.90 (dd, J = 12.3, 2.6 Hz, 1H), 3.76 (dd, J = 12.3, 3.5 Hz, 1H), 2.75 (ddd, J = 14.1, 6.8, 5.8 Hz, 1H), 2.63 (t, J = 2.5 Hz, 1H), 2.53 (ddd, J = 14.3, 9.4, 6.2 Hz, 1H), 1.92 (d, J = 1.1 Hz, 3H). ^{13}C NMR (126 MHz, MeOD) δ 168.9, 166.4, 163.7, 152.4, 148.3, 139.4, 138.2, 124.6, 121.8, 120.8, 113.3, 111.8, 86.3, 84.0, 80.5, 72.2, 62.4, 55.2, 36.6, 30.1, 12.5. HRMS (ESI-Q-TOF): m/z [M+Na]$^+$ Calcd for $C_{21}H_{21}N_5NaO_6^+$: 462.1384; found: 462.1395.

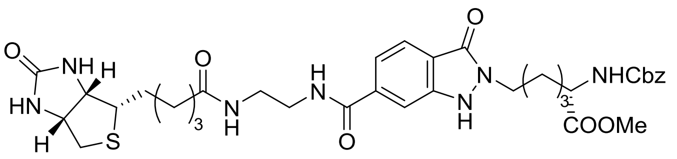

21

^{1}H NMR (500 MHz, MeOD) δ 7.82 (d, J = 8.3 Hz, 1H), 7.74 (s, 1H), 7.55 (d, J = 8.2 Hz, 1H), 7.35 – 7.26 (m, 5H), 5.08 – 5.00 (m, 2H), 4.42 (dd, J = 7.7, 4.9 Hz, 1H), 4.19 – 4.14 (m, 2H), 4.04 – 3.93 (m, 2H), 3.67 (s, 3H), 3.53 (t, J = 5.8 Hz, 2H), 3.44 (t, J = 5.9 Hz, 2H), 3.04 – 3.00 (m, 1H), 2.84 (dd, J = 12.8, 5.0 Hz, 1H), 2.65 (d, J = 12.7 Hz, 1H), 2.21 (td, J = 7.2, 1.4 Hz, 2H), 1.91 – 1.47 (m, 8H), 1.45 – 1.33 (m, 4H). ^{13}C NMR (126 MHz, MeOD) δ 176.7, 174.6, 169.7, 162.0, 158.7, 146.3, 139.1, 138.1, 129.5, 129.0, 128.7, 124.4, 121.0, 119.8, 112.5, 67.6, 63.2, 61.6, 56.8, 55.2, 52.6, 44.8, 41.2, 41.0, 39.9, 36.8, 31.9, 29.6, 29.4, 28.7, 26.8, 23.8. HRMS (ESI-Q-TOF): m/z [M+Na]$^+$ Calcd for $C_{35}H_{45}N_7NaO_8S^+$: 746.2943; found: 746.2962.

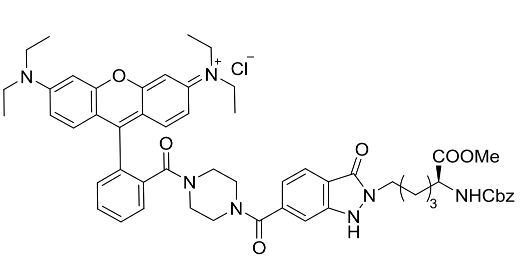

22

^{1}H NMR (500 MHz, DMSO) δ 7.78 – 7.67 (m, 3H), 7.64 (d, J = 7.9 Hz, 1H), 7.51 (d, J = 5.1 Hz, 1H), 7.33 (d, J = 9.2 Hz, 5H), 7.21 (s, 1H), 7.11 (d,

J = 19.7 Hz, 4H), 6.98 (d, *J* = 8.1 Hz, 1H), 6.93 (d, *J* = 2.0 Hz, 2H), 4.99 (s, 2H), 3.98 (d, *J* = 4.5 Hz, 1H), 3.78 (s, 2H), 3.64 (d, *J* = 7.0 Hz, 8H), 3.58 (s, 3H), 3.33 (dd, *J* = 120.8, 60.2 Hz, 8H), 1.65 (m, 4H), 1.29 (s, 2H), 1.19 (t, *J* = 6.9 Hz, 12H). ^{13}C NMR (126 MHz, DMSO) δ 172.9, 168.7, 166.6, 159.7, 157.1, 156.2, 155.6, 155.2, 145.0, 138.3, 136.9, 135.2, 131.8, 130.7, 129.8, 128.4, 127.8, 127.8, 127.7, 127.5, 123.3, 117.6, 116.8, 114.5, 114.3, 113.1, 110.6, 96.0, 65.5, 53.8, 51.8, 45.4, 42.9, 30.1, 27.3, 22.6, 12.4. HRMS (ESI-Q-TOF): m/z [M-Cl]$^+$ Calcd for $C_{55}H_{62}N_7O_8$+: 948.4654; found: 948.4645.

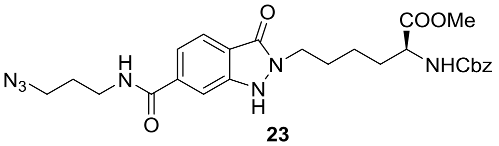

23

^{1}H NMR (500 MHz, DMSO) δ 10.44 (s, 1H), 8.63 (t, *J* = 5.6 Hz, 1H), 7.69 (dd, *J* = 7.5, 1.7 Hz, 3H), 7.53 (dd, *J* = 8.4, 0.8 Hz, 1H), 7.38 – 7.23 (m, 5H), 5.08 – 4.94 (m, 2H), 4.03 – 3.96 (m, 1H), 3.85 – 3.76 (m, 2H), 3.59 (s, 3H), 3.42 (t, *J* = 6.7 Hz, 2H), 3.35 (dd, *J* = 12.5, 6.7 Hz, 2H), 1.80 (p, *J* = 6.8 Hz, 2H), 1.76 – 1.59 (m, 4H), 1.36 – 1.27 (m, 2H). ^{13}C NMR (126 MHz, DMSO) δ 172.8, 166.0, 159.8, 156.1, 145.2, 137.3, 136.9, 128.3, 127.8, 127.7, 122.9, 119.5, 118.9, 111.1, 65.5, 53.7, 51.8, 48.6, 42.9, 36.8, 30.1, 28.3, 27.3, 22.5. HRMS (ESI-Q-TOF): m/z [M+Na]$^+$ Calcd for $C_{26}H_{31}N_7NaO_6{}^+$: 560.2228; found: 560.2226.

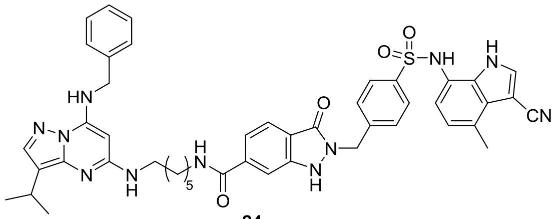

24

^{1}H NMR (500 MHz, DMSO) δ 11.99 (s, 1H), 8.56 (t, *J* = 5.5 Hz, 1H), 8.17 (d, *J* = 2.6 Hz, 2H), 7.80 – 7.69 (m, 4H), 7.66 (s, 1H), 7.61 (s, 1H), 7.55 (dd, *J* = 8.2, 1.2 Hz, 1H), 7.41 – 7.31 (m, 6H), 7.24 (t, *J* = 6.7 Hz, 1H), 6.78 – 6.72 (m, 1H), 6.62 – 6.52 (m, 2H), 5.10 (s, 2H), 4.45 (d, *J* = 6.4 Hz, 2H), 3.30 – 3.17 (m, 4H), 2.95 (dt, *J* = 13.7, 6.9 Hz, 1H), 2.56 (s, 3H), 1.57 – 1.43 (m, 4H), 1.38-1.28 (m, 4H), 1.24 (d, *J* = 6.9 Hz, 6H). ^{13}C NMR (126 MHz, DMSO) δ 165.7, 163.2, 156.5, 146.0, 145.6, 145.5, 141.8, 139.6, 138.6, 138.0, 135.2, 130.5, 128.4, 127.9, 127.3, 126.9, 126.8, 126.5, 123.1, 122.4, 118.0, 117.4, 117.4, 111.3, 110.5, 84.3, 46.4, 44.6, 42.7, 29.0, 29.0, 26.4, 26.3, 23.4, 23.1, 17.6. HRMS (ESI-Q-TOF): m/z [M+H]$^+$ Calcd for $C_{47}H_{50}N_{11}O_4S^+$: 864.3762; found: 864.3755.

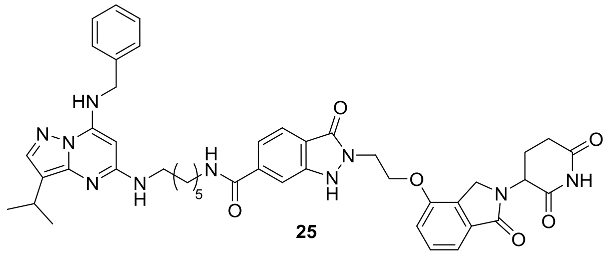

25

^{1}H NMR (500 MHz, DMSO) δ 10.95 (s, 1H), 10.42 (s, 1H), 8.54 (t, *J* = 5.4 Hz, 1H), 7.71 (d, *J* = 8.0 Hz, 2H), 7.68 (s, 1H), 7.59 (s, 1H), 7.52 (d, *J* = 9.1 Hz, 1H), 7.46 (t, *J* = 7.8 Hz, 1H), 7.31 (tt, *J* = 15.2, 7.4 Hz, 6H), 7.22 (t, *J* = 6.8 Hz, 1H), 6.53 (t, *J* = 5.3 Hz, 1H), 5.09 (s, 1H), 5.05 (dd, *J* = 13.3, 5.1 Hz, 1H), 4.44 (d, *J* = 6.4 Hz, 4H), 4.23 (s, 2H), 4.13 (dd, *J* = 38.6, 17.4 Hz, 2H), 3.27 – 3.16 (m, 4H), 2.97 – 2.83 (m, 2H), 2.58 (d, *J* = 16.5 Hz, 1H), 2.33 – 2.20 (m, 1H), 1.93 (dd, *J* = 11.6, 6.3 Hz, 1H), 1.55 – 1.44 (m, 4H), 1.31 (s, 4H), 1.22 (d, *J* = 6.9 Hz, 8H). ^{13}C NMR (126 MHz, DMSO) δ 172.9, 171.0, 168.0, 165.8, 161.0, 156.5, 153.4, 146.1, 145.9, 145.6, 139.7, 138.6, 137.9, 133.3, 130.0, 129.9, 128.5, 127.0, 126.8, 123.0, 119.8, 118.8, 115.6, 115.1, 111.4, 110.6, 72.5, 65.6, 51.6, 45.0, 44.6, 43.2, 31.2, 29.0, 26.4, 23.4, 23.1, 22.5. HRMS (ESI-SQD2): m/z [M+H]$^+$ Calcd for $C_{45}H_{51}N_{10}O_6{}^+$: 827.3988; found: 827.3981.

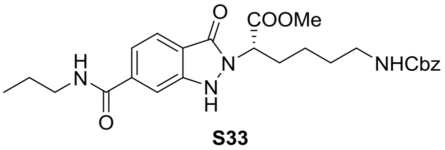

S33

^{1}H NMR (500 MHz, MeOD) δ 7.82 (d, *J* = 8.3 Hz, 1H), 7.74 (s, 1H), 7.52 (dd, *J* = 8.3, 1.1 Hz, 1H), 7.28 (dd, *J* = 9.2, 5.9 Hz, 5H), 5.24 (dd, *J* = 9.9, 5.8 Hz, 1H), 4.99 (s, 2H), 3.74 (s, 3H), 3.07 (t, *J* = 6.7 Hz, 2H), 2.17 (dd, *J* = 15.3, 9.3 Hz, 2H), 1.65 (dd, *J* = 14.5, 7.3 Hz, 2H), 1.60 – 1.46 (m, 3H), 1.22 (dd, *J* = 11.1, 4.9 Hz, 1H), 0.99 (t, *J* = 7.4 Hz, 3H). ^{13}C NMR (126 MHz, MeOD) δ 171.7, 169.6, 163.6, 158.9, 148.1, 139.7, 138.4, 129.4, 128.9, 128.6, 124.6, 120.8, 119.2, 113.2, 67.3, 57.6, 53.2, 43.0, 41.2, 30.5, 30.0, 24.1, 23.7, 11.8. HRMS (ESI-Q-TOF): m/z [M+H]$^+$ Calcd for $C_{26}H_{33}N_4O_6{}^+$: 497.2395; found: 497.2405.

6.11 Synthesis of compound S40

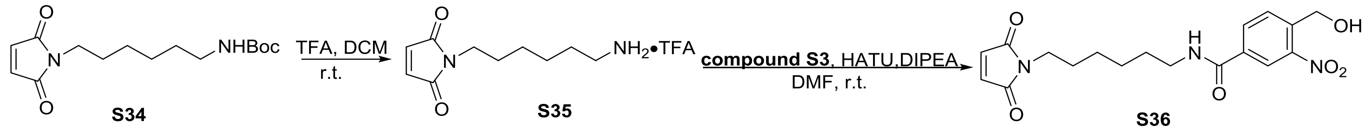

Compound S34 was synthesized according to published literature.[15]

Compound S34 (74mg，0.25 mmol) added to a solution of 2:1 CH_2Cl_2 and trifluoroacetic acid (1.5 mL) and left to stir at room temperature for 1 h. The solvent was removed *in vacuo*. **Compound S3** (54mg, 0.275 mmol), HATU (104mg, 0.275 mmol), and the resulting oil was then dissolved in dry DMF. DIPEA (227 μL, 1.38 mmol) was added to this solution. The reaction was allowed to stir at room temperature for 1 h. The mixture was diluted with ethyl acetate, washed with water and brine, dried over Na_2SO_4. After the solvent was removed in vacuo, the resulting residues were purified by flash chromatography to give **compound S36** as yellow oil (30 mg，32%). ^{1}H NMR (600 MHz, DMSO) δ 8.74 (t, *J* = 5.5 Hz, 1H), 8.49 (d, *J* = 1.7 Hz, 1H), 8.20 (dd, *J* = 8.1, 1.7 Hz, 1H), 7.92 (d, *J* = 8.1 Hz, 1H), 6.99 (s, 2H), 5.65 (t, *J* = 5.5 Hz, 1H), 4.87 (d, *J* = 5.5 Hz, 2H), 3.39 (t, *J* = 7.1 Hz, 2H), 3.25 (dd, *J* = 12.8, 6.9 Hz, 2H), 1.53 – 1.46 (m, 4H), 1.34 – 1.29 (m, 2H), 1.25 – 1.22 (m, 2H). ^{13}C NMR (126 MHz, DMSO) δ 171.1, 163.8, 146.6, 141.1, 134.4, 134.0, 132.0, 128.4, 123.0, 59.9, 37.0, 28.8, 27.9, 25.9, 25.8. HRMS (ESI-Q-TOF): m/z $[M+H]^+$ Calcd for $C_{18}H_{22}N_3O_6^+$: 376.1503; found: 376.1496.

6.12 Syntheis of FITC-NH_2

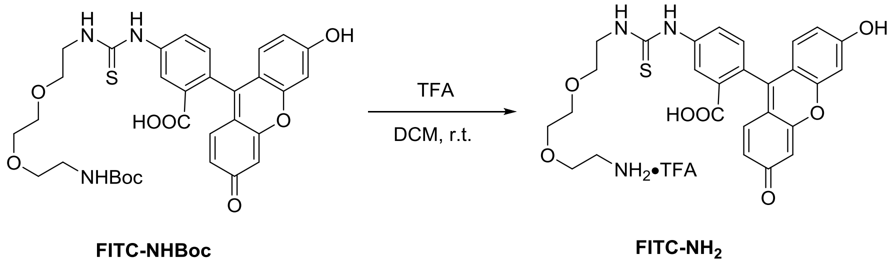

FITC-NHBoc was synthesized according to published literature.[16]

FITC-NHBoc (70 mg, 0.11 mmol) dissolved in DCM (2.5 mL), trifluoroacetic acid (1.0 mL) was added and the solution was left to stir at room temperature for 1 h. The solvent was removed *in vacuo* and lyophilized to give FITC-NH_2 as brilliant yellow oil (59 mg, 99%). ^{1}H NMR (500 MHz, DMSO) δ 8.59 – 8.19 (m, 1H), 8.07 – 7.64 (m, 3H), 7.19 (d, *J* = 7.5 Hz, 1H), 6.79 – 6.49 (m, 4H), 3.91 – 3.36 (m, 10H), 2.99 (s, 2H). ^{13}C NMR (126 MHz, DMSO) δ 180.8, 168.7, 159.7, 159.0, 158.8, 152.1, 147.1, 141.7, 129.6, 129.2, 126.6, 124.2, 117.3, 116.6, 115.0, 112.8, 109.9, 102.4, 83.7, 69.8, 69.6, 68.5, 66.8, 43.8. HRMS (ESI-Q-TOF): m/z $[M+H]^+$ Calcd for $C_{27}H_{28}N_3O_7S^+$: 538.1642; found: 538.1633.

6.13 Isolated yields of indazolone product 3/5/6/9 in large scale

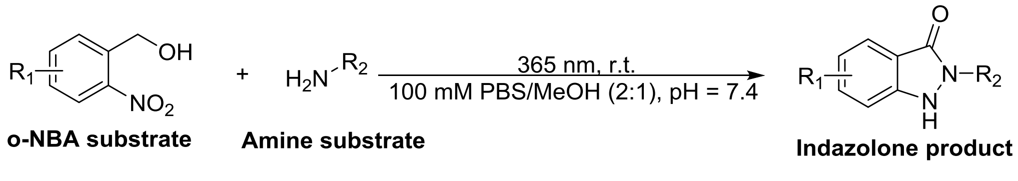

o-NBA substrates (40 mM in MeOH, 10.5 mL) and amine substrates (10 mM in MeOH, 10.5 mL) in 100 mM PBS/MeOH (1:1, pH = 7.4, 31.5 mL) were stirred and treated with 365 nm UV light for 40 min (20 min x 2, interval 10 min) in a 10 cm glass dish. The mixture was diluted with H_2O, saturated with NaCl and extracted with ethyl acetate. The organic layer was washed with H_2O and brine, dried over Na_2SO_4 and concentrated in vacuo. The residue was purified by silica chromatography to afford indazolone products.

7. UPLC-MS Analysis of Reactions and Products

The yields of the reaction of different o-NBAs and primary amine substrates are shown in Supplementary Table 5. After the reaction, the samples were collected, diluted with MeOH/H_2O and analyzed by UPLC-MS. Yields were determined by ratio of peak area value of experiment to that of internal standard product on reverse-phase HPLC, are reported as an average of three independent trials. Stock solution: o-NBA substrates (10 mM) in MeOH, and amine substrates (10 mM) in MeOH/DMSO/H_2O. o-NBA substrates (2 mM) and amine (0.5 mM) in 100 mM PBS/MeOH (2:1, pH = 7.4) were treated with 365 nm UV light for 7 min and shaken at 25 ^{o}C for 30 min. (Except compound 10/11/13, o-NBA substrate and amine were treated with 365 nm UV light for 10 min. Compound 22/24/25, o-NBA substrate and amine were treated with 365 nm UV light for 15 min. Compound 7, the reaction concentration of o-NBA substrate was 4 mM.)

Supplementary Table 5 | Reactivity of o-NBA-tag and amine substrates.

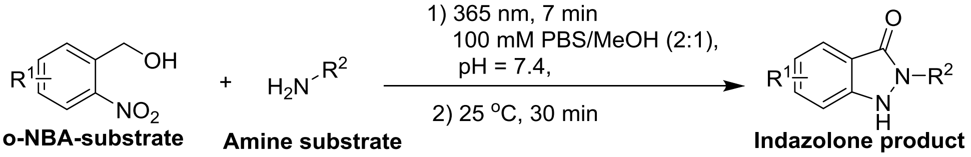

Entries	o-NBA-substrates	Amine substrates	Structures of Products and Yields
1	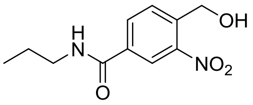 **o-NBA-amide 1**	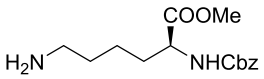 **Cbz-Lys-OMe**	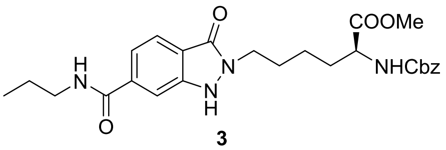 **3** **> 98%**
2	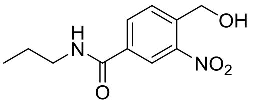 **o-NBA-amide 1**	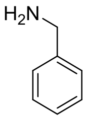 **Phenylmethanamine**	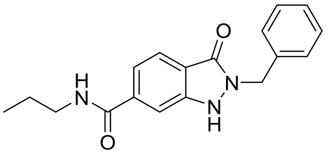 **5** **98%**
3	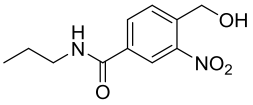 **o-NBA-amide 1**	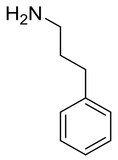 **3-Phenylpropan-1-amine**	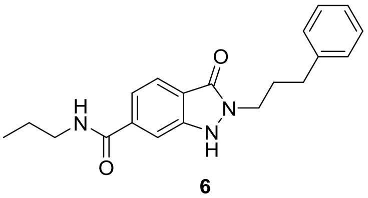 **6** **>98%**
4	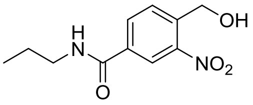 **o-NBA-amide 1** **(4.0 mM reaction concentration)**	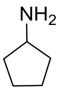 **Cyclopentanamine**	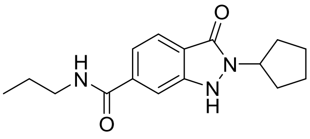 **7** **97%**
5	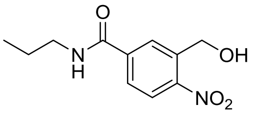 **S1**	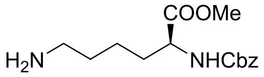 **Cbz-Lys-OMe**	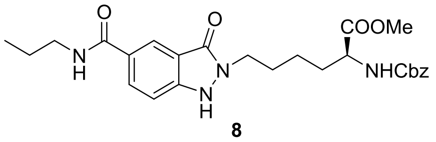 **8** **>98%**

6	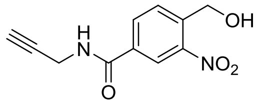 **o-NBA-alkyne** **S14**	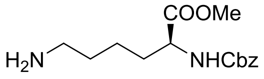 **Cbz-Lys-OMe**	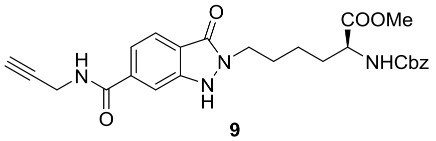 **9** **> 98%**
7	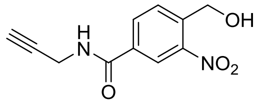 **o-NBA-alkyne** **S14**	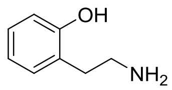 **2-(2-aminoethyl)phenol**	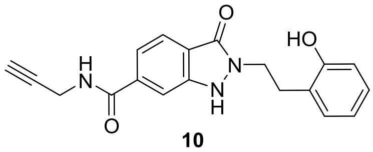 **10** **93%**
8	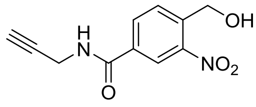 **o-NBA-alkyne** **S14**	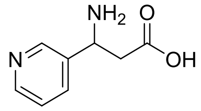 **3-amino-3-(pyridin-3-yl) propanoic acid**	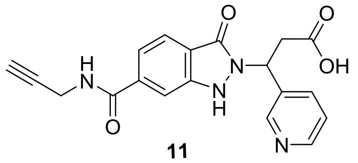 **11** **86%**
9	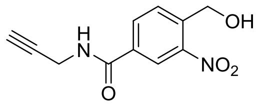 **o-NBA-alkyne** **S14**	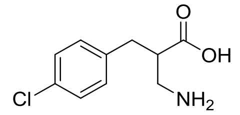 **3-amino-2-(4-chlorobenzyl) propanoic acid**	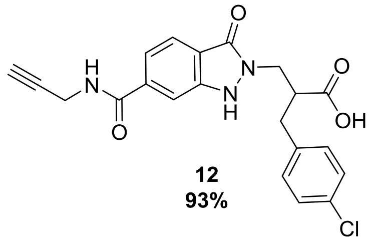 **12** **93%**
10	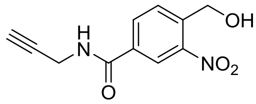 **o-NBA-alkyne** **S14**	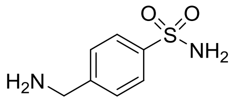 **4-(aminomethyl) benzenesulfonamide**	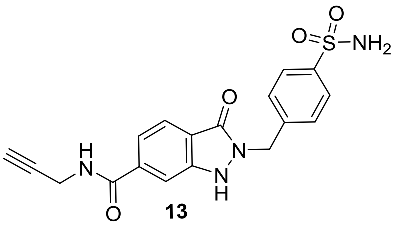 **13** **92%**
11	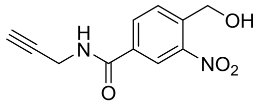 **o-NBA-alkyne** **S14**	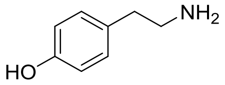 **4-(2-aminoethyl)phenol**	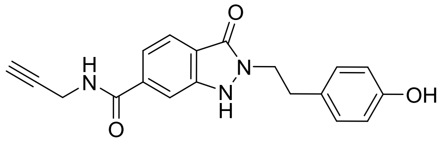 **14** **89%**
12	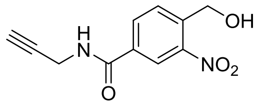 **o-NBA-alkyne** **S14**	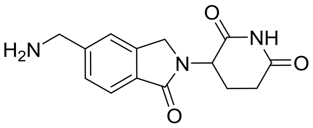 **S15** **3-(5-(aminomethyl)-1-oxoisoindolin-2-yl) piperidine-2,6-dione**	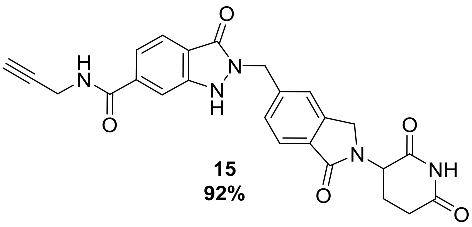 **15** **92%**
13	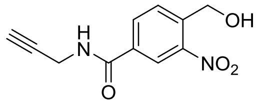 **o-NBA-alkyne** **S14**	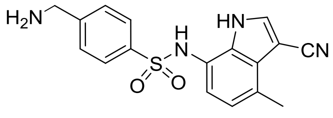 **S16** **4-(aminomethyl)-*N*-(3-cyano-4-methyl-1*H*-indol-7-yl) benzenesulfonamide**	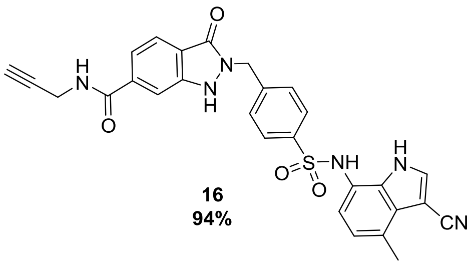 **16** **94%**

14	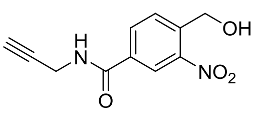<br>**o-NBA-alkyne**<br>**S14**	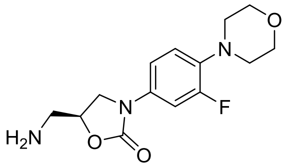<br>**Deacetyl Linezolid**	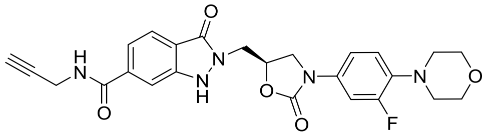<br>**17**<br>**98%**
15	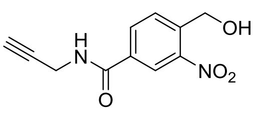<br>**o-NBA-alkyne**<br>**S14**	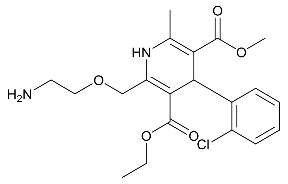<br>**Amlodipine**	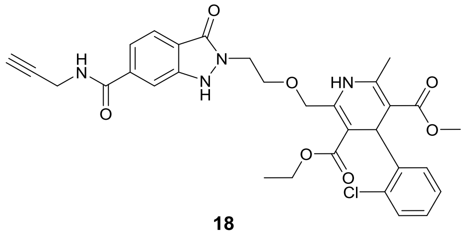<br>**18**<br>**> 98%**
16	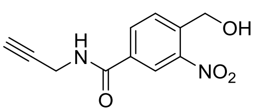<br>**o-NBA-alkyne**<br>**S14**	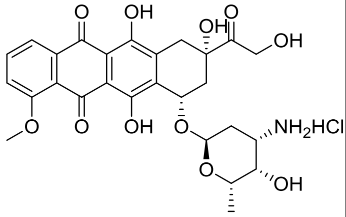<br>**Doxorubicin hydrochloride**	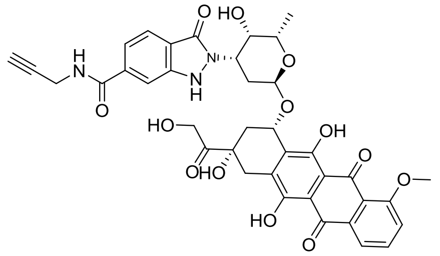<br>**19**<br>**85%**
17	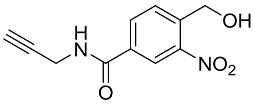<br>**o-NBA-alkyne**<br>**S14**	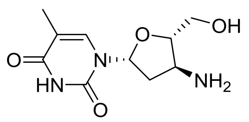<br>**3'-Amino-3'-Deoxythymidinee**	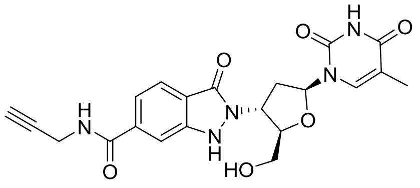<br>**20**<br>**> 98%**
18	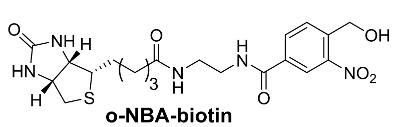<br>**o-NBA-biotin**	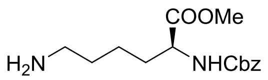<br>**Cbz-Lys-OMe**	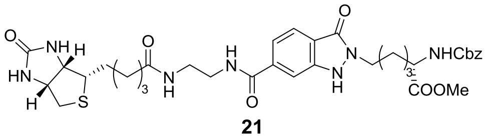<br>**21**<br>**>98%**
19	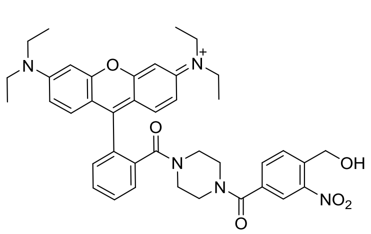<br>**Rho-o-NBA**	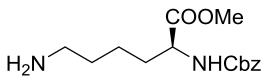<br>**Cbz-Lys-OMe**	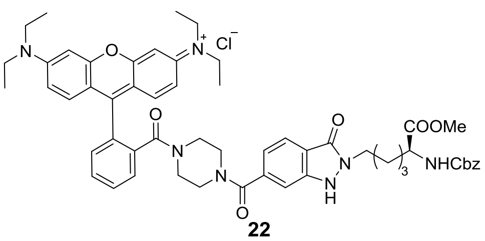<br>**22**<br>**98%**
20	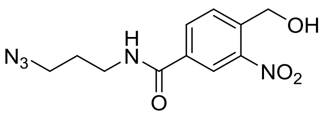<br>**o-NBA-N_3**	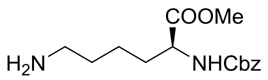<br>**Cbz-Lys-OMe**	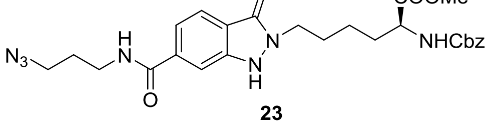<br>**23**<br>**> 98%**

21	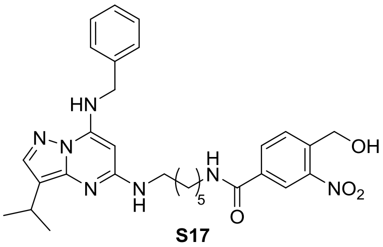<br>**S17**	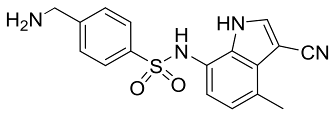<br>**S16**<br>**4-(aminomethyl)-*N*-**<br>**(3-cyano-4-methyl-1*H*-indol-7-yl)**<br>**benzenesulfonamide**	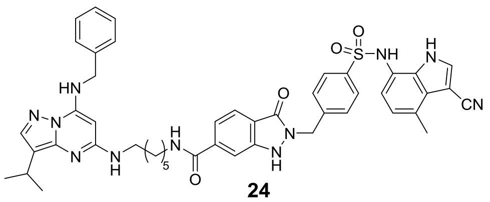<br>**24**<br>**72%**
22	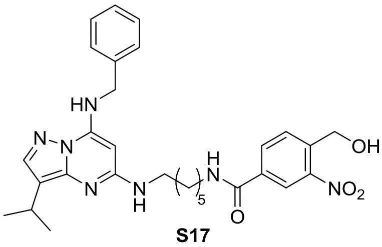<br>**S17**	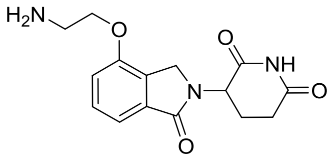<br>**S18**<br>**3-(5-(aminomethyl)-**<br>**1-oxoisoindolin-2-yl)**<br>**piperidine-2,6-dione**	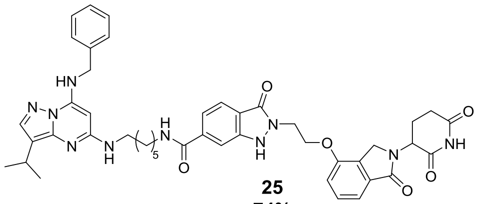<br>**25**<br>**74%**

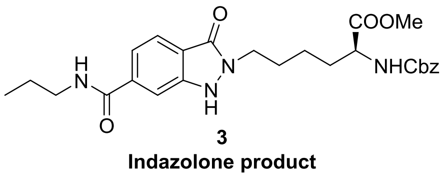

3
Indazolone product

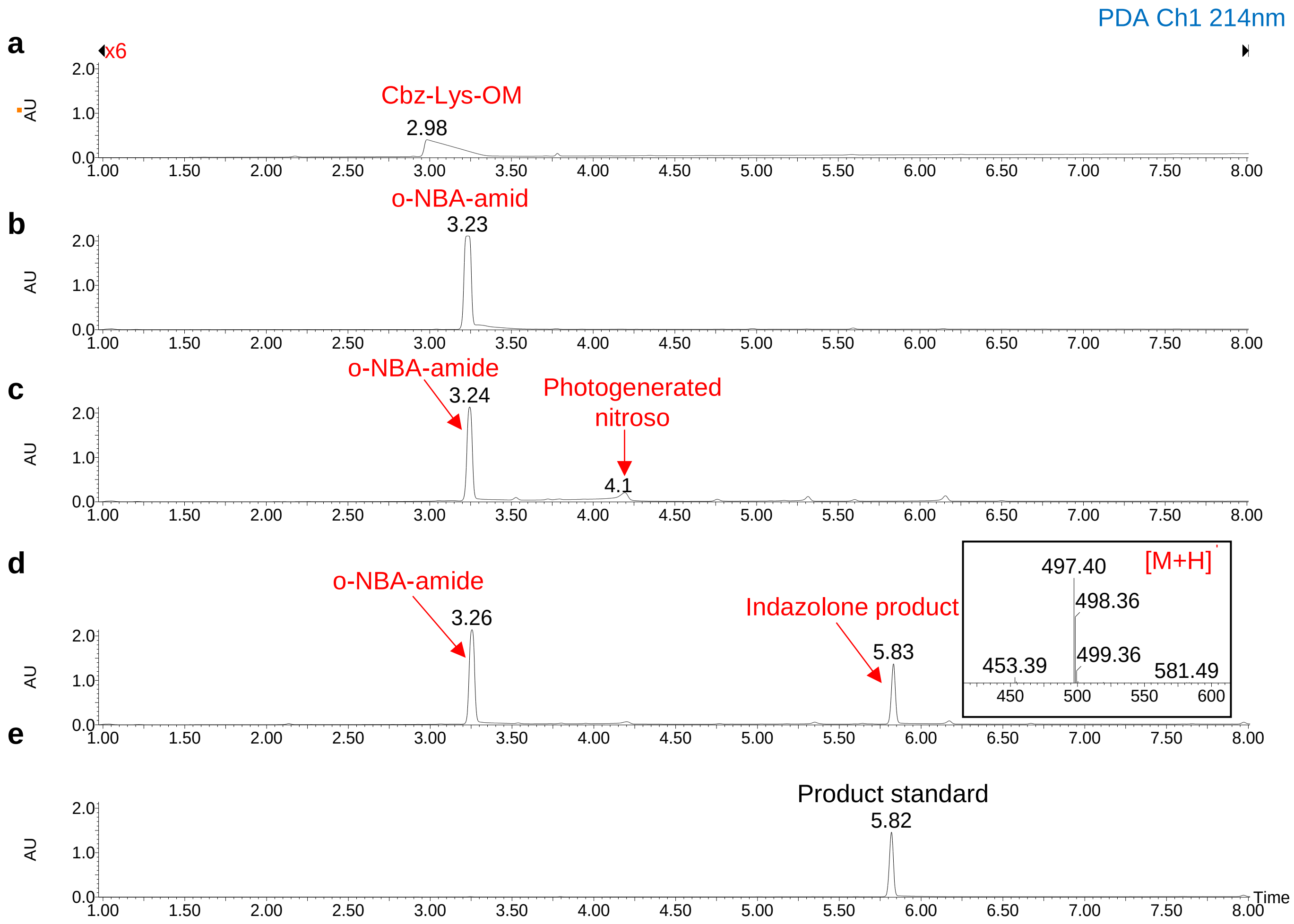

Supplementary Fig. 35 | a) Cbz-Lys-OMe (0.5 mM); b) o-NBA amide 1 (2 mM); c) o-NBA amide 1 (2 mM) was irradiated with 365 nm UV light; d) o-NBA amide 1 (2 mM) photo-reacted with Cbz-Lys-OMe (0.5 mM); e) Product standard (0.5 mM) (NMR and HRMS data in Synthesis Part).

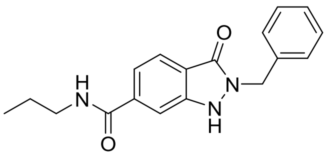

5

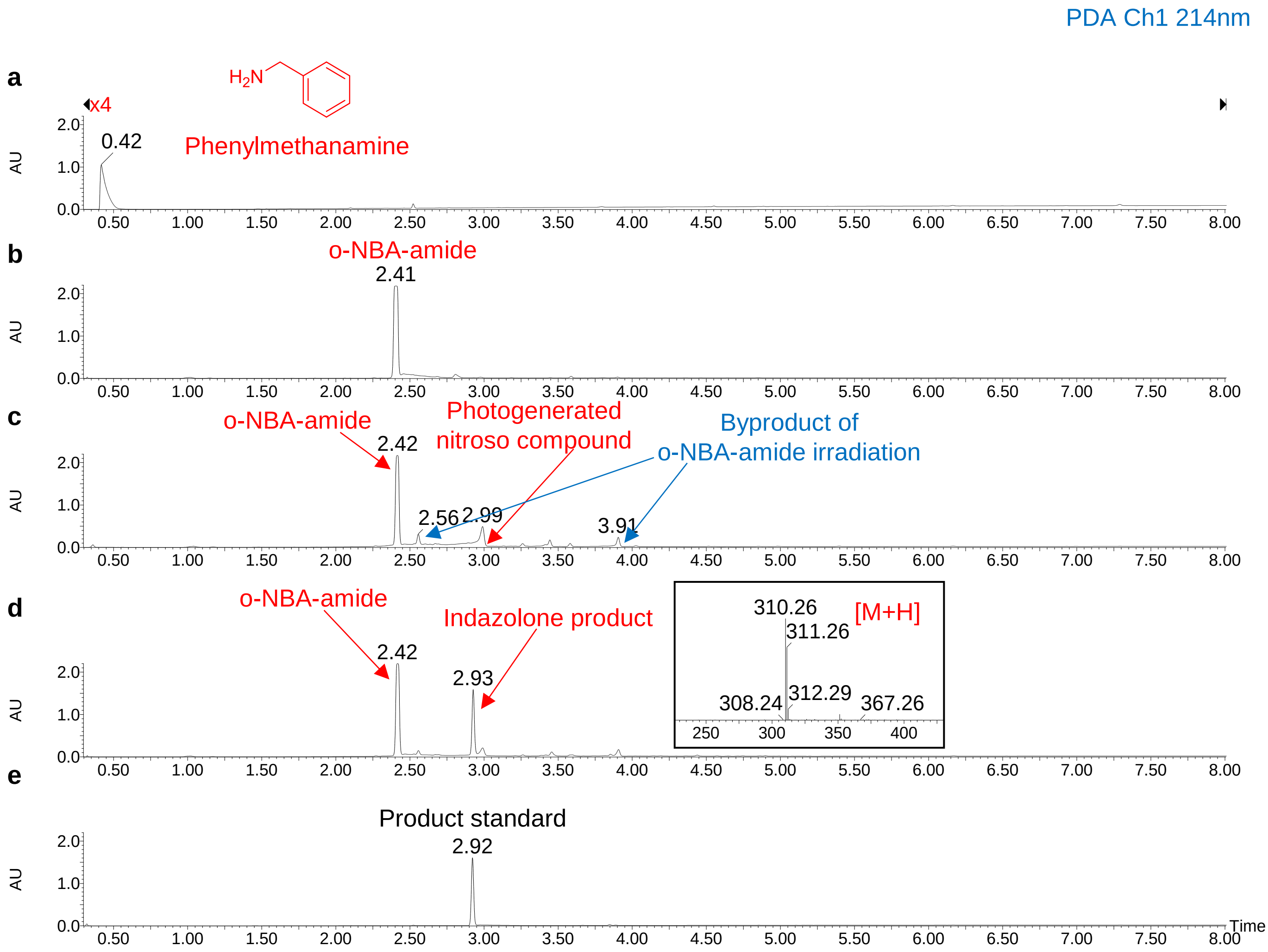

Supplementary Fig. 36 | a) Benzylamine (0.5 mM); b) o-NBA amide 1 (2 mM); c) o-NBA amide 1 (2 mM) was irradiated with 365 nm UV light; d) o-NBA amide 1 (2 mM) photo-reacted with benzylamine (0.5 mM); e) Product standard (0.5 mM) (NMR and HRMS data in Synthesis Part).

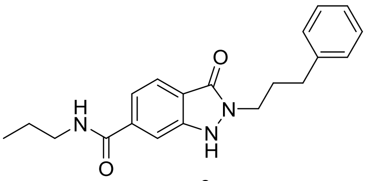

6

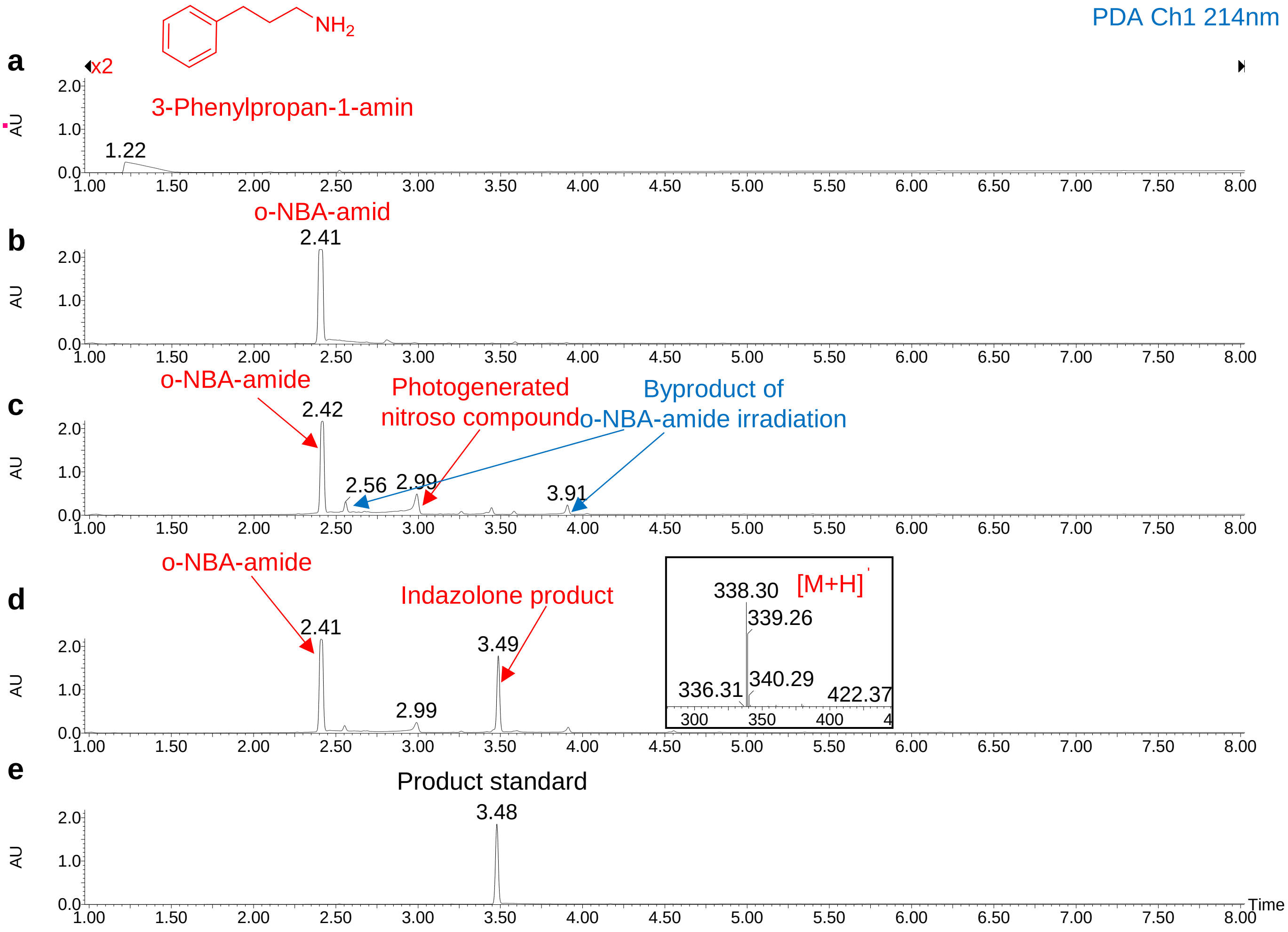

Supplementary Fig. 37 | a) 3-Phenylpropan-1-amine (0.5 mM); b) o-NBA amide 1 (2 mM); c) o-NBA amide 1 (2 mM) was irradiated with 365 nm UV light; d) o-NBA amide 1 (2 mM) photo-reacted with 3-Phenylpropan-1-amine (0.5 mM); e) Product standard (0.5 mM) (NMR and HRMS data in Synthesis Part).

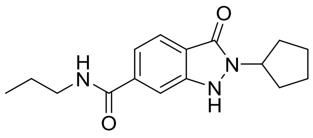

7

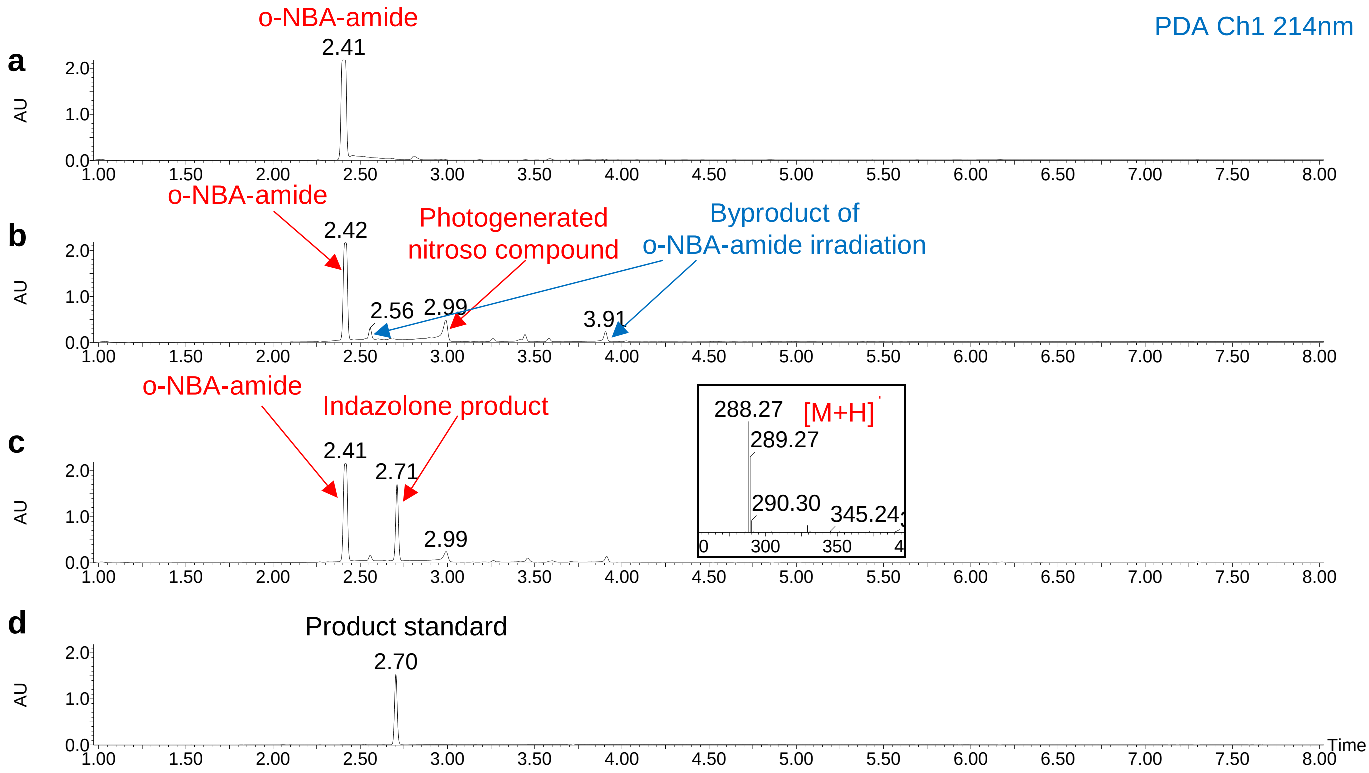

Supplementary Fig. 38 | a) o-NBA amide 1 (4 mM); b) o-NBA amide 1 (4 mM) was irradiated with 365 nm UV light; c) o-NBA amide 1 (4 mM) photo-reacted with cyclopentylamine (0.5 mM); d) Product standard (0.5 mM) (NMR and HRMS data in Synthesis Part).

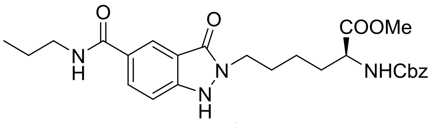

8

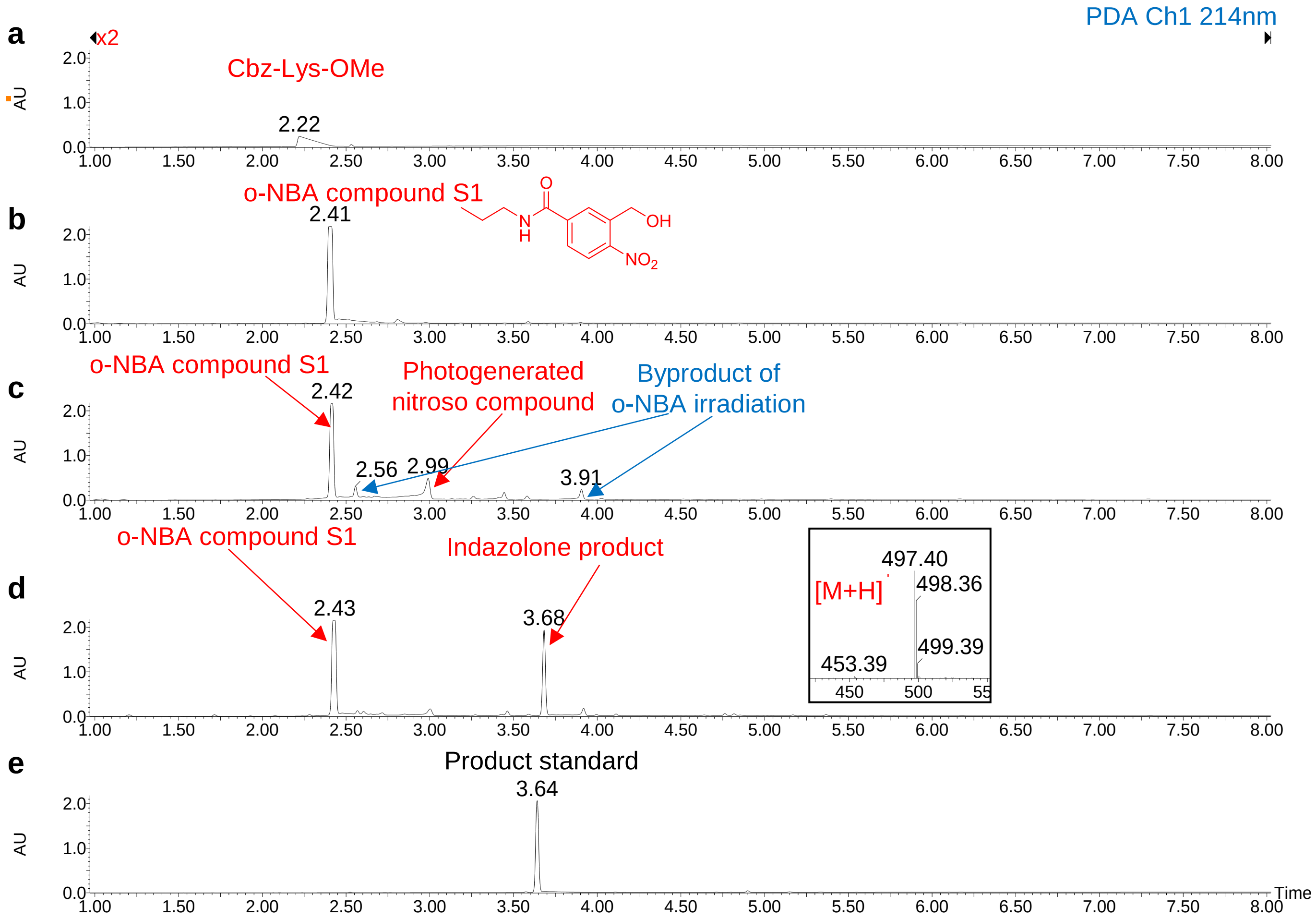

Supplementary Fig. 39 | a) Cbz-Lys-OMe (0.5 mM); b) Compound S1 (2 mM); c) Compound S1 (2 mM) was irradiated with 365 nm UV light; d) Compound S1 (2 mM) photo-reacted with Cbz-Lys-OMe (0.5 mM); e) Product standard (0.5 mM) (NMR and HRMS data in Synthesis Part).

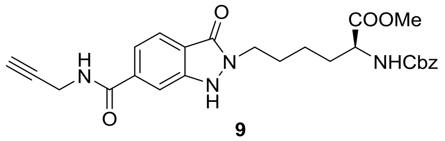

9

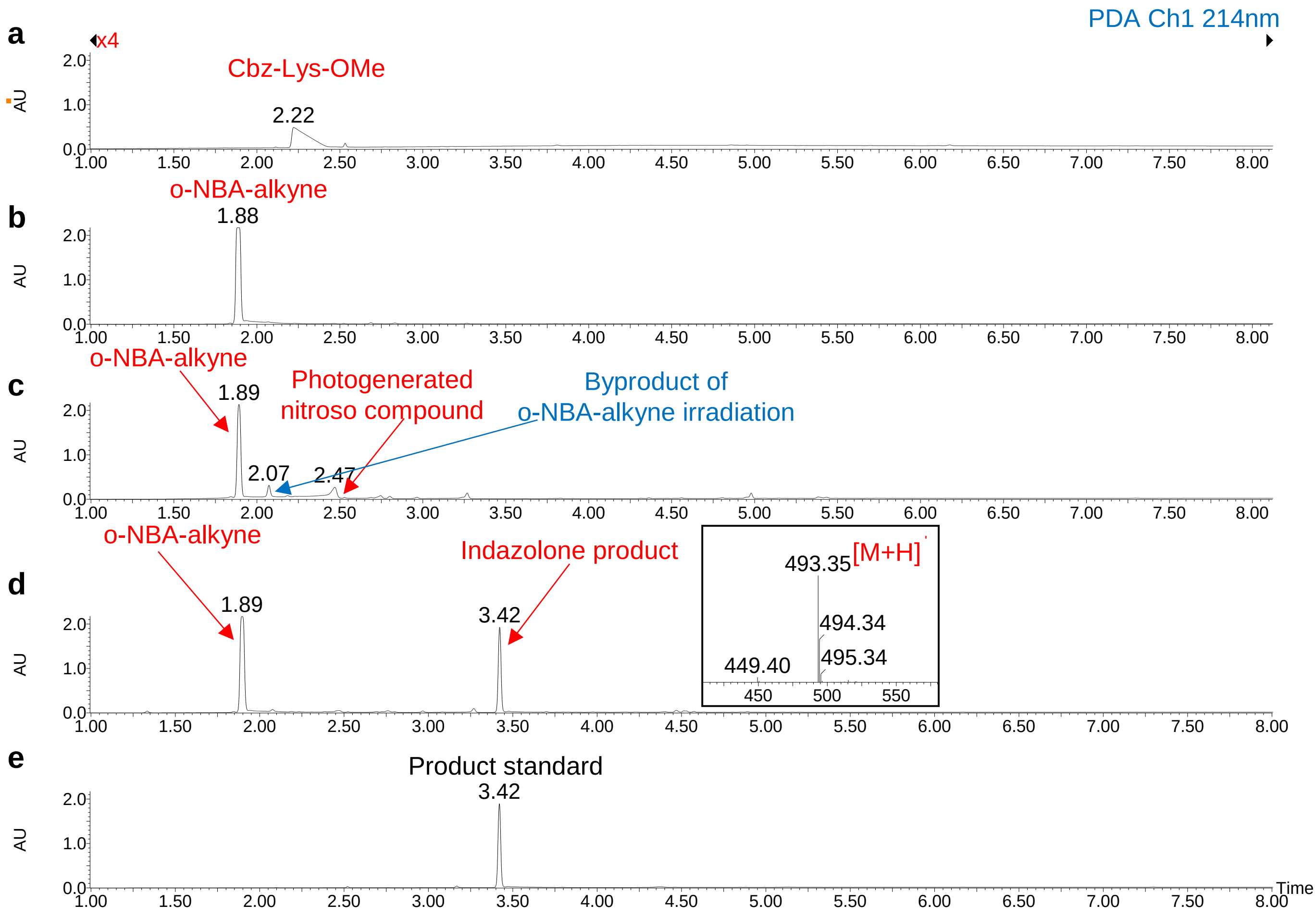

Supplementary Fig. 40 | a) Cbz-Lys-OMe (0.5 mM); b) o-NBA-alkyne (2 mM); c) o-NBA-alkyne (2 mM) was irradiated with 365 nm UV light; d) o-NBA-alkyne (2 mM) photo-reacted with Cbz-Lys-OMe (0.5 mM); e) Product standard (0.5 mM) (NMR and HRMS data in Synthesis Part).

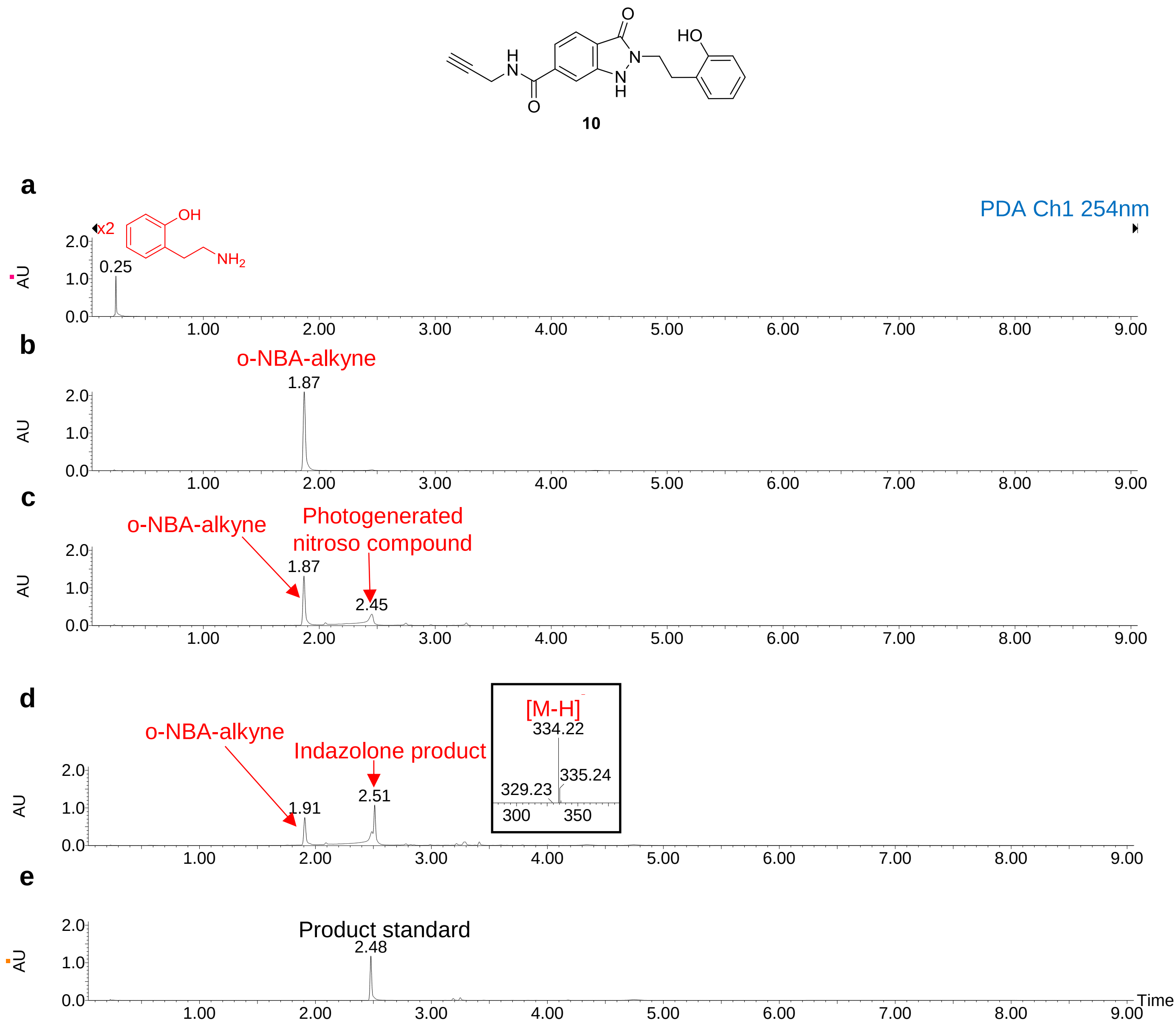

Supplementary Fig. 41 | a) Amine substrate (0.5 mM); b) o-NBA-alkyne (2 mM); c) o-NBA-alkyne (2 mM) was irradiated with 365 nm UV light; d) o-NBA-alkyne (2 mM) photo-reacted with amine substrate (0.5 mM); e) Product standard (0.5 mM) (NMR and MS data in Synthesis Part).

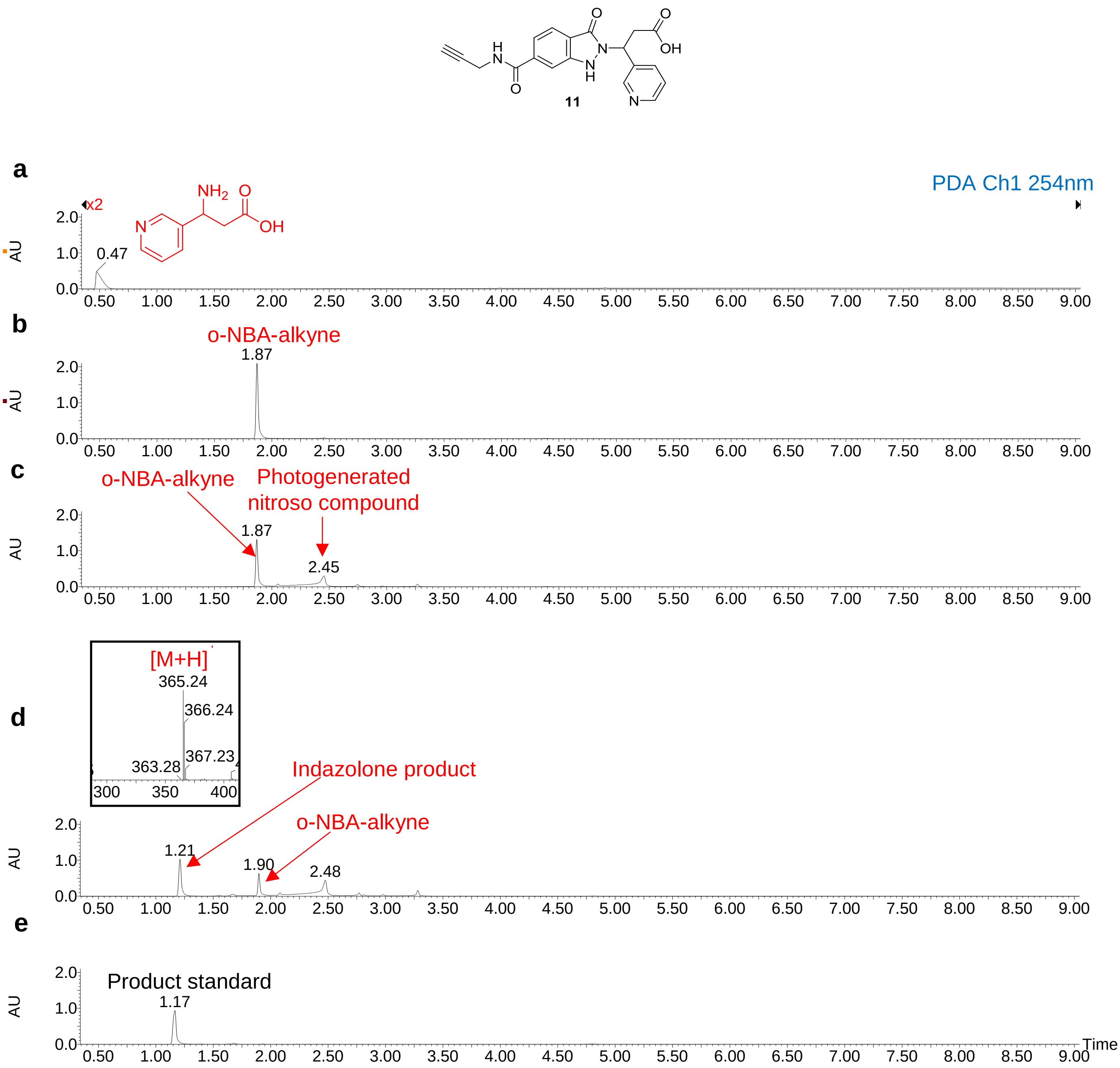

Supplementary Fig. 42 | a) Amine substrate (0.5 mM); b) o-NBA-alkyne (2 mM); c) o-NBA-alkyne (2 mM) was irradiated with 365 nm UV light; d) o-NBA-alkyne (2 mM) photo-reacted with amine substrate (0.5 mM); e) Product standard (0.5 mM) (NMR and MS data in Synthesis Part).

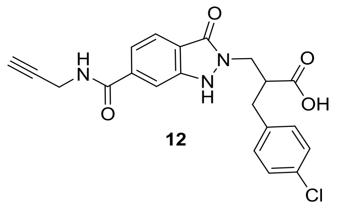

12

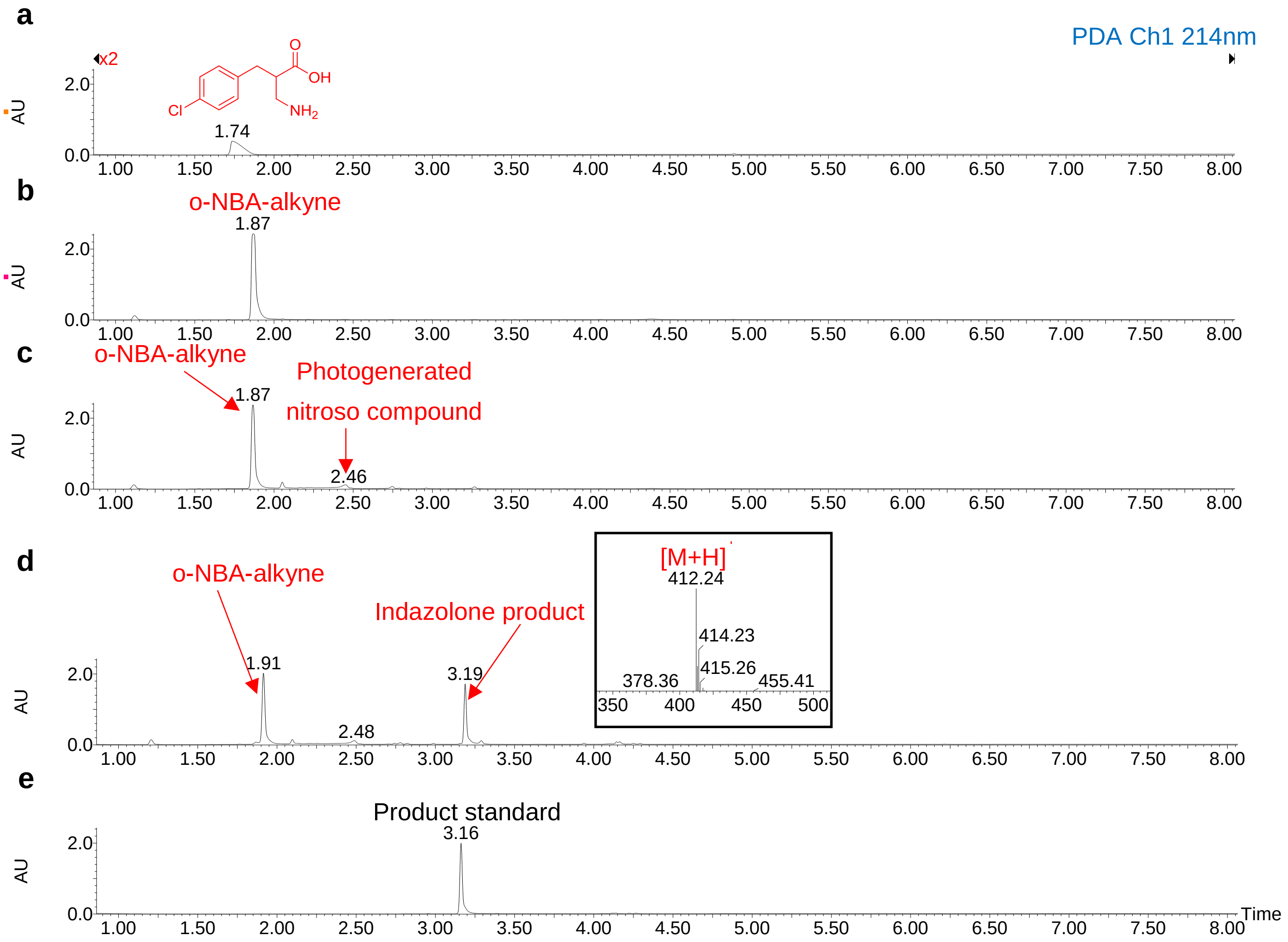

Supplementary Fig. 43 | a) Amine substrate (0.5 mM); b) o-NBA-alkyne (2 mM); c) o-NBA-alkyne (2 mM) was irradiated with 365 nm UV light; d) o-NBA-alkyne (2 mM) photo-reacted with amine substrate (0.5 mM); e) Product standard (0.5 mM) (NMR and MS data in Synthesis Part).

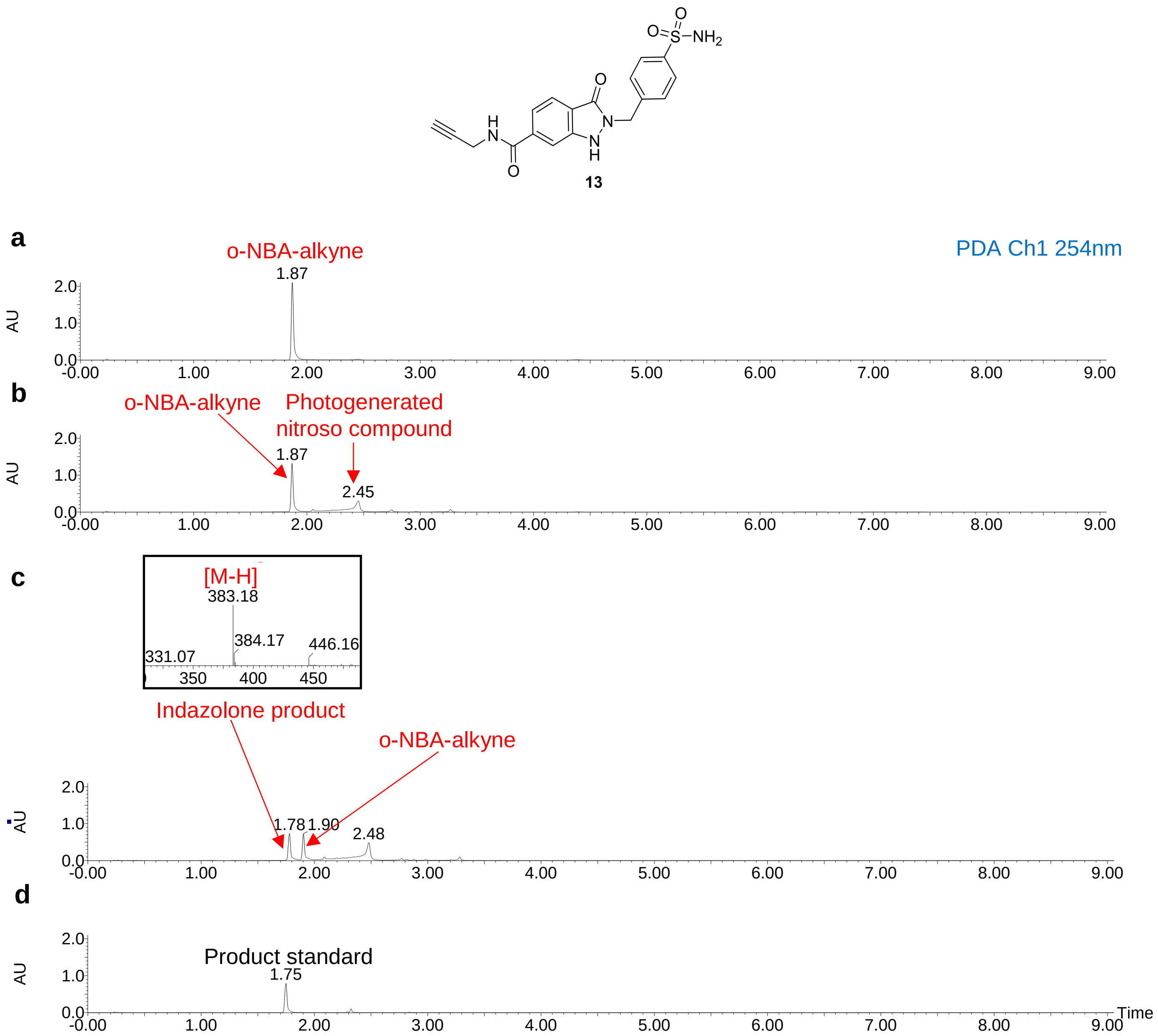

Supplementary Fig. 44 | a) o-NBA-alkyne (2 mM); b) o-NBA-alkyne (2 mM) was irradiated with 365 nm UV light; c) o-NBA-alkyne (2 mM) photo-reacted with amine substrate (0.5 mM); d) Product standard (0.5 mM) (NMR and MS data in Synthesis Part).

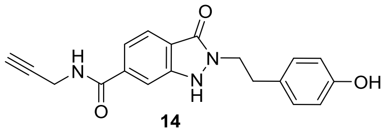

14

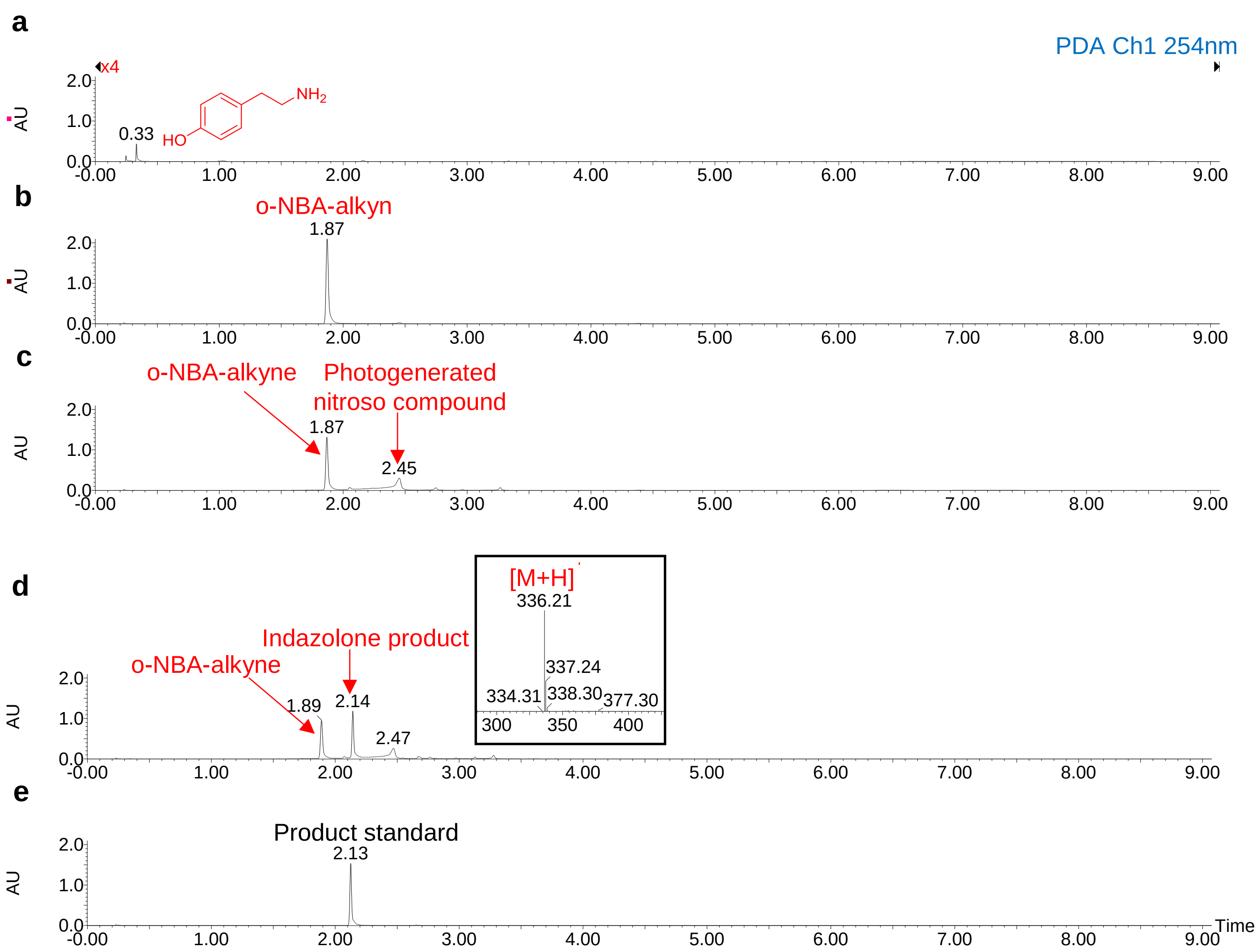

Supplementary Fig. 45 | a) Amine substrate (0.5 mM); b) o-NBA-alkyne (2 mM); c) o-NBA-alkyne (2 mM) was irradiated with 365 nm UV light; d) o-NBA-alkyne (2 mM) photo-reacted with amine substrate (0.5 mM); e) Product standard (0.5 mM) (NMR and MS data in Synthesis Part).

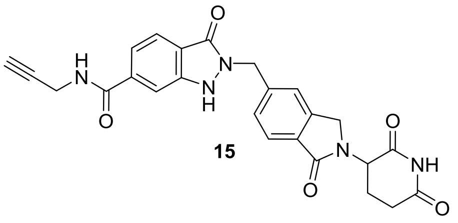

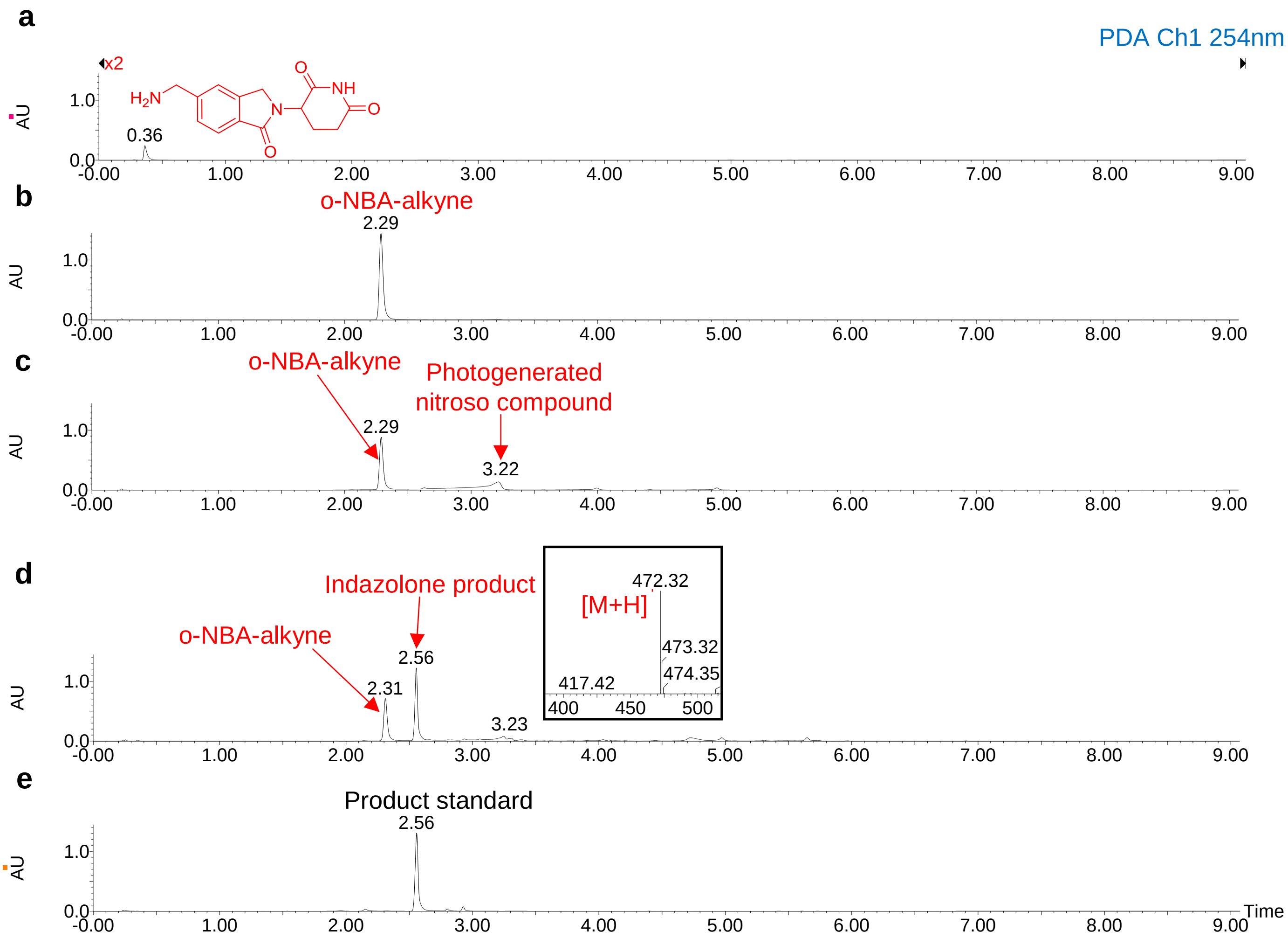

Supplementary Fig. 46 | a) Amine substrate (0.5 mM); b) o-NBA-alkyne (2 mM); c) o-NBA-alkyne (2 mM) was irradiated with 365 nm UV light; d) o-NBA-alkyne (2 mM) photo-reacted with amine substrate (0.5 mM); e) Product standard (0.5 mM) (NMR and MS data in Synthesis Part).

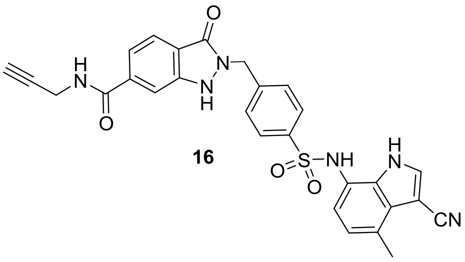

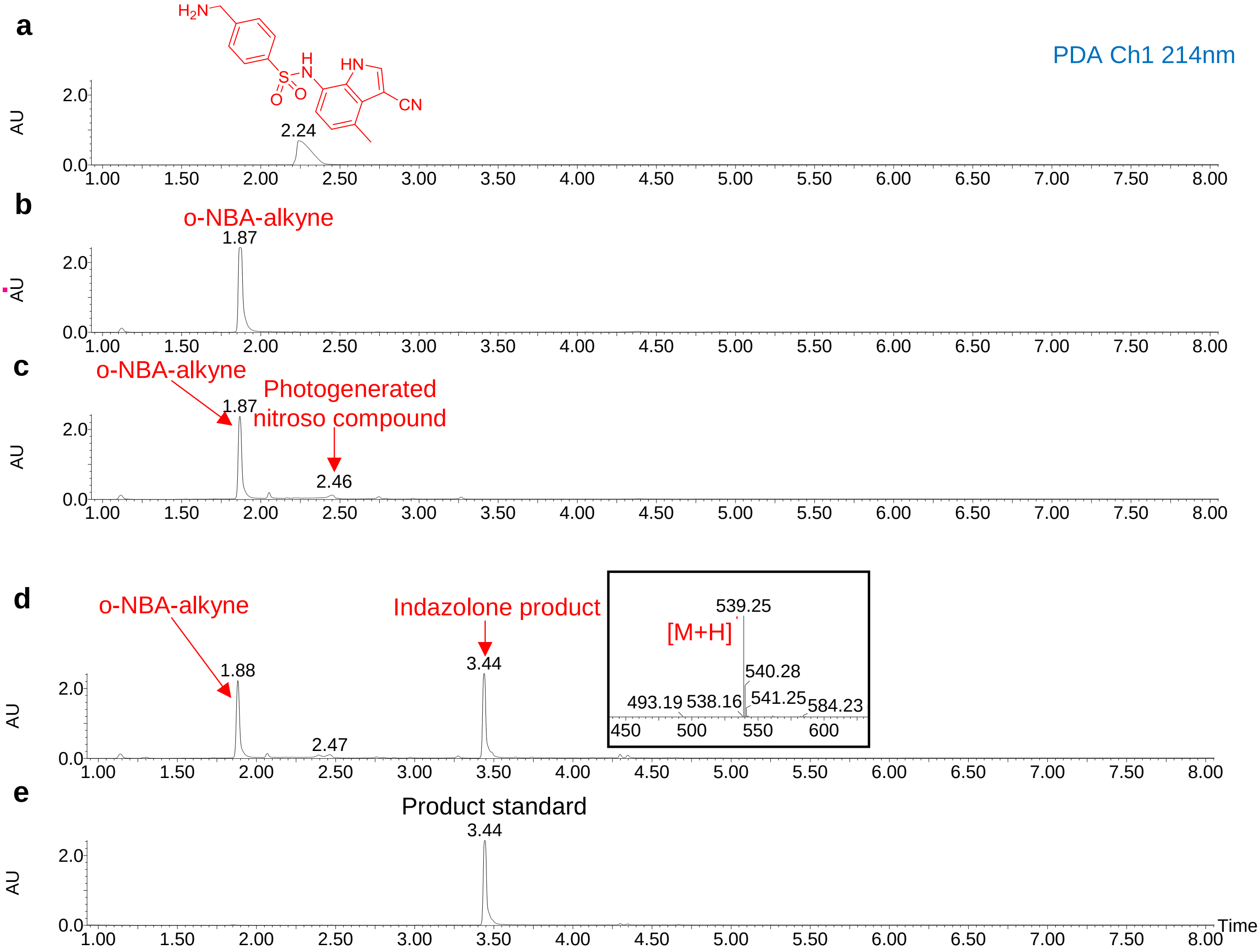

Supplementary Fig. 47 | a) Amine substrate (0.5 mM); b) o-NBA-alkyne (2 mM); c) o-NBA-alkyne (2 mM) was irradiated with 365 nm UV light; d) o-NBA-alkyne (2 mM) photo-reacted with amine substrate (0.5 mM); e) Product standard (0.5 mM) (NMR and MS data in Synthesis Part).

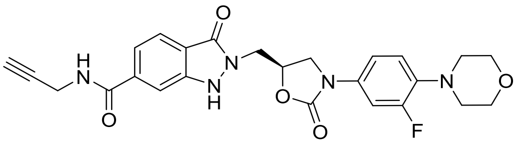

17

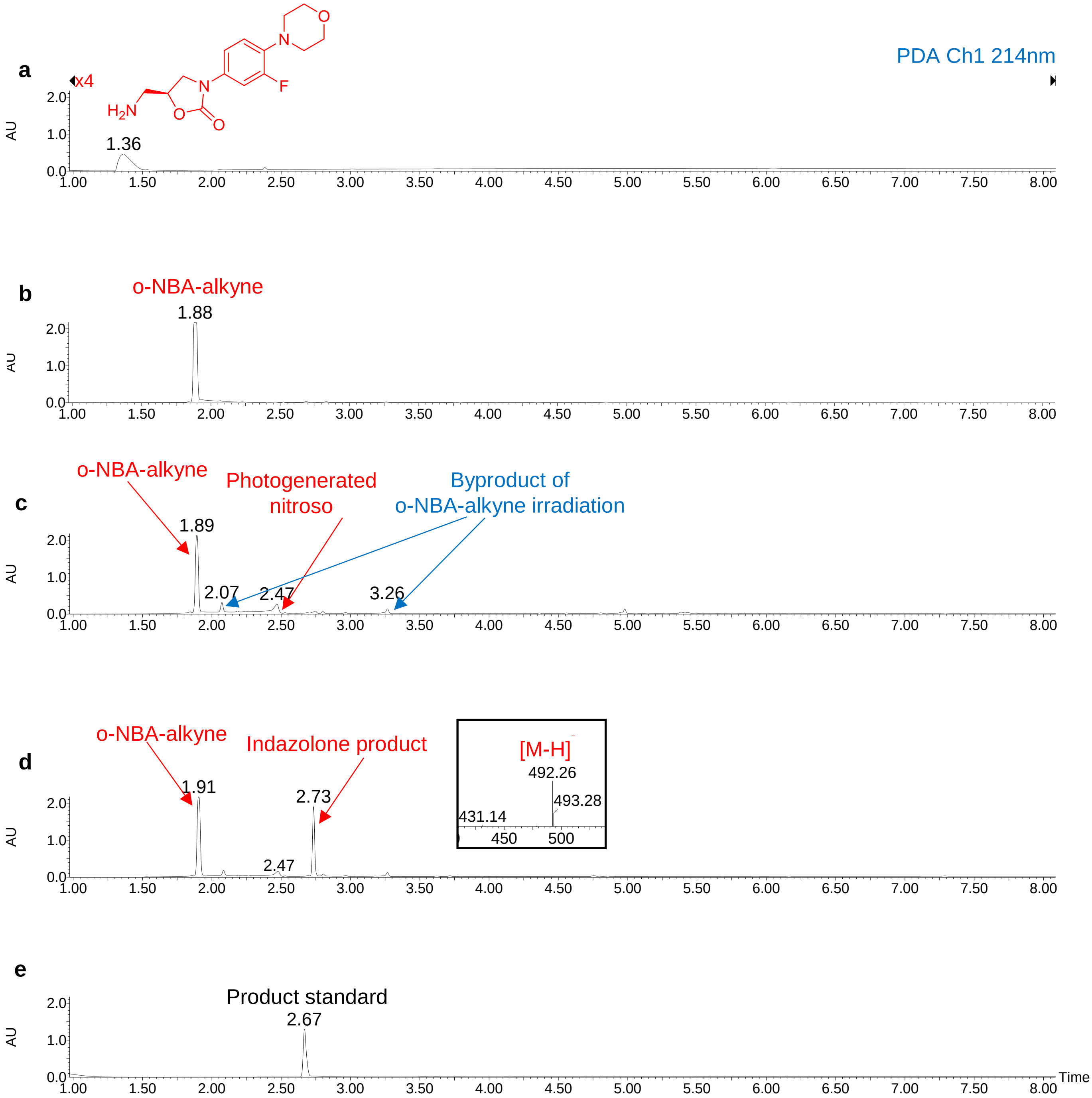

Supplementary Fig. 48 | a) Amine substrate (0.5 mM); b) o-NBA-alkyne (2 mM); c) o-NBA-alkyne (2 mM) was irradiated with 365 nm UV light; d) o-NBA-alkyne (2 mM) photo-reacted with amine substrate (0.5 mM); e) Product standard (0.5 mM) (NMR and HRMS data in Synthesis Part).

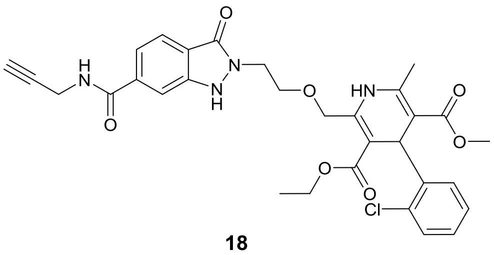

18

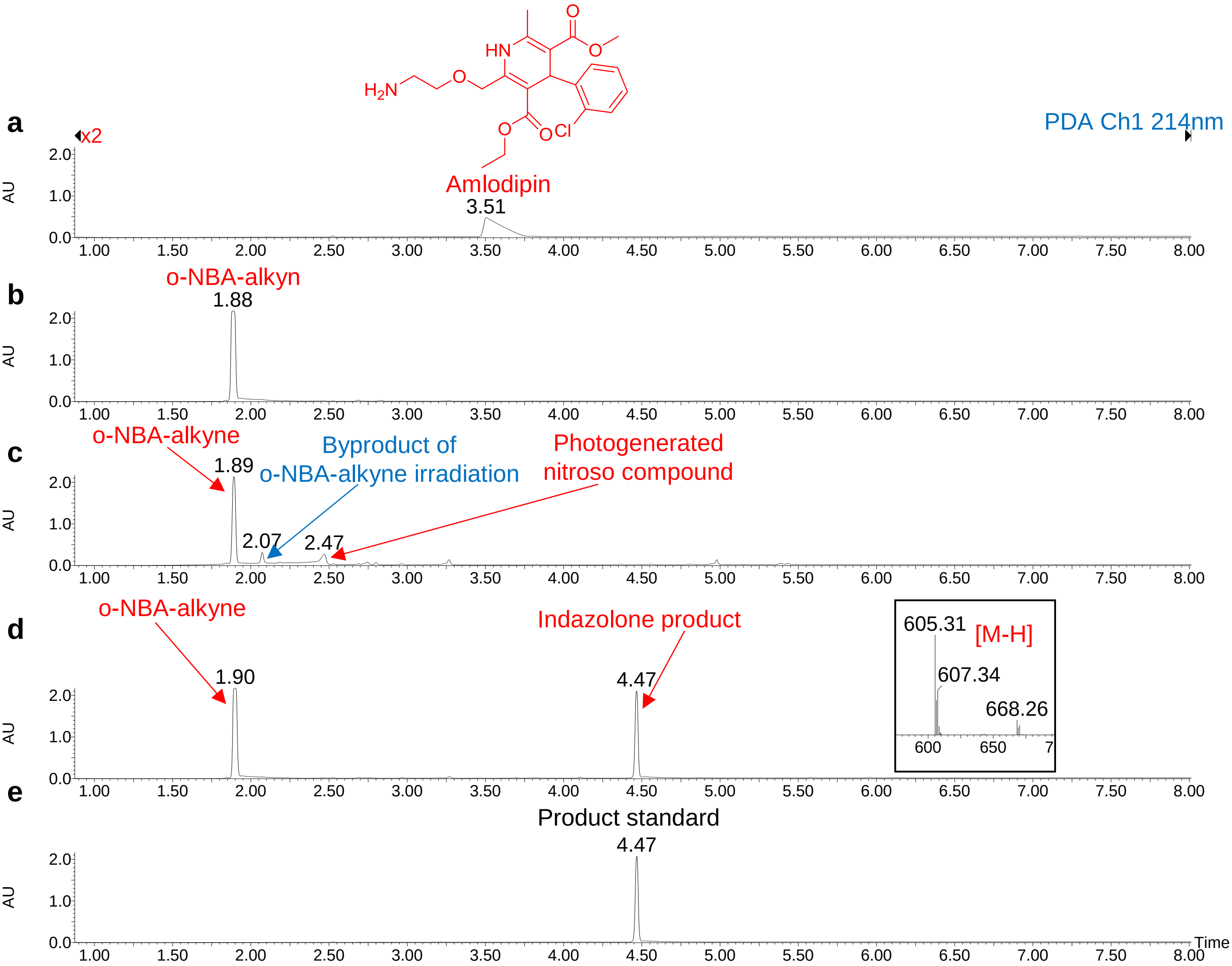

Supplementary Fig. 49 | a) Amine substrate (0.5 mM); b) o-NBA-alkyne (2 mM); c) o-NBA-alkyne (2 mM) was irradiated with 365 nm UV light; d) o-NBA-alkyne (2 mM) photo-reacted with amine substrate (0.5 mM); e) Product standard (0.5 mM) (NMR and HRMS data in Synthesis Part).

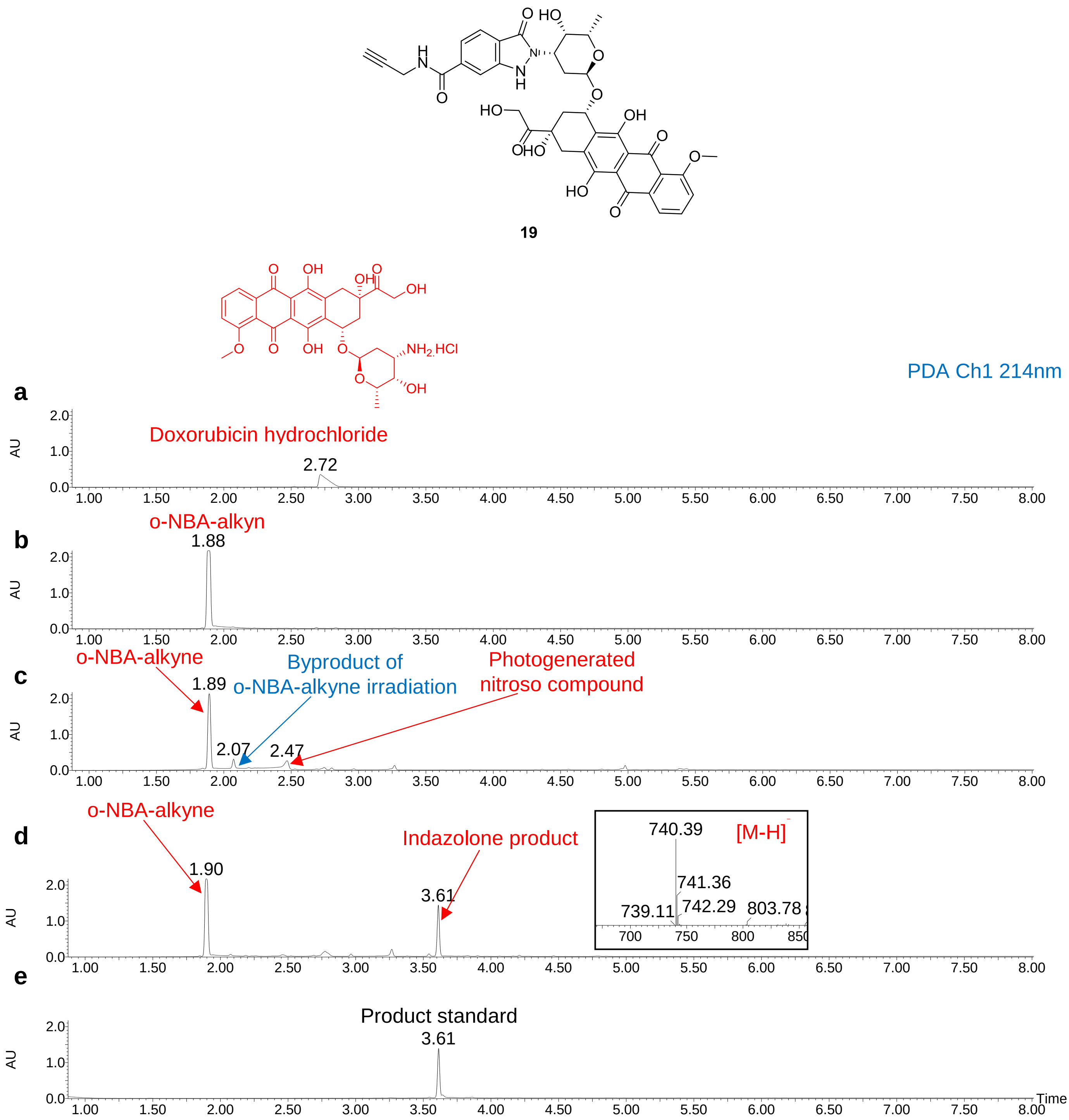

Supplementary Fig. 50 | a) Amine substrate (0.5 mM); b) o-NBA-alkyne (2 mM); c) o-NBA-alkyne (2 mM) was irradiated with 365 nm UV light; d) o-NBA-alkyne (2 mM) photo-reacted with amine substrate (0.5 mM); e) Product standard (0.5 mM) (NMR and HRMS data in Synthesis Part).

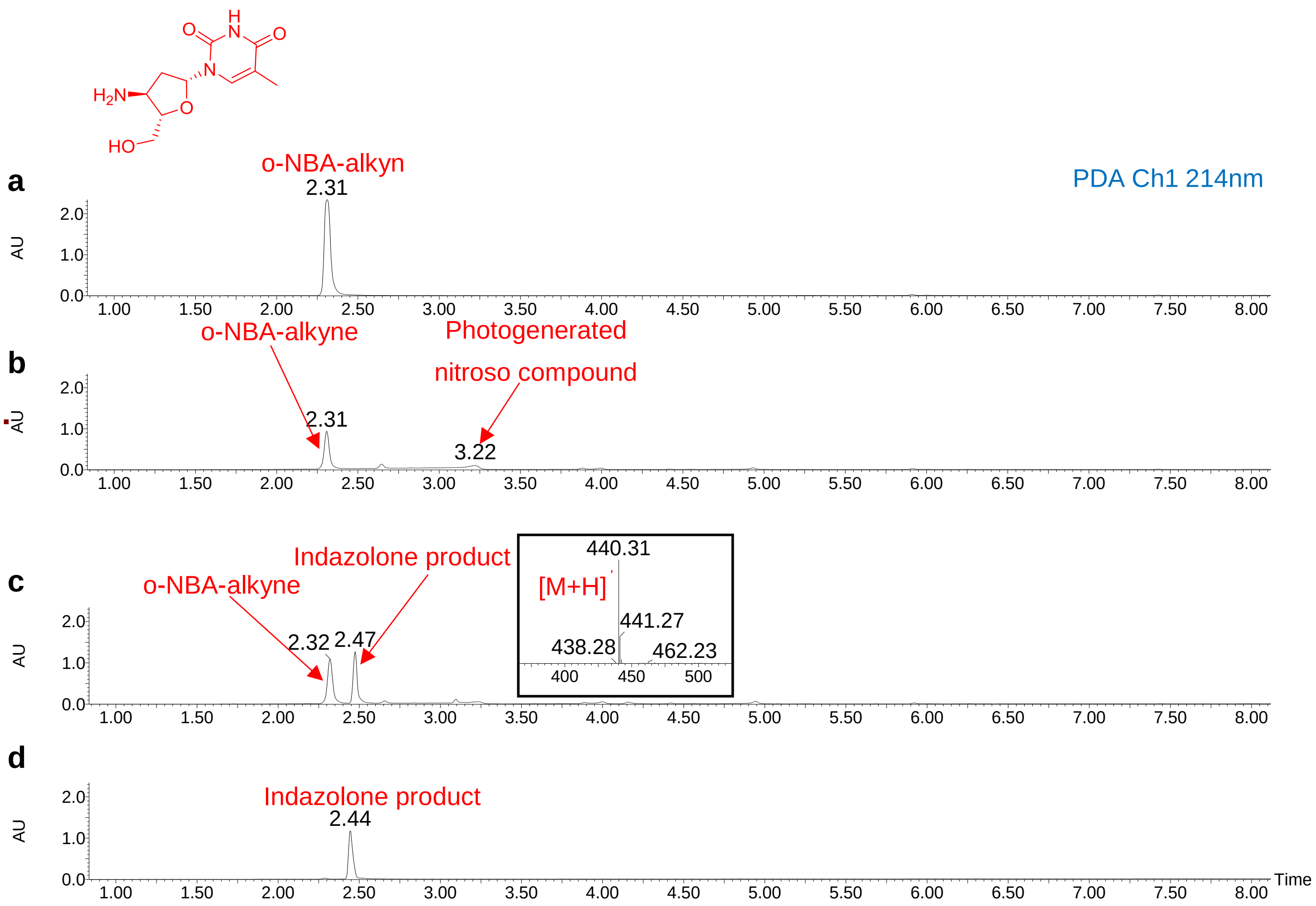

Supplementary Fig. 51| a) o-NBA-alkyne (2 mM); b) o-NBA-alkyne (2 mM) was irradiated with 365 nm UV light; c) o-NBA-alkyne (2 mM) photo-reacted with amine substrate (0.5 mM); d) Product standard (0.5 mM) (NMR and MS data in Synthesis Part).

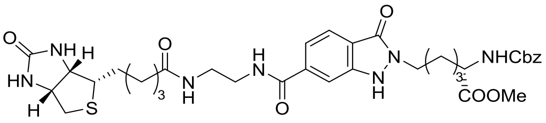

21

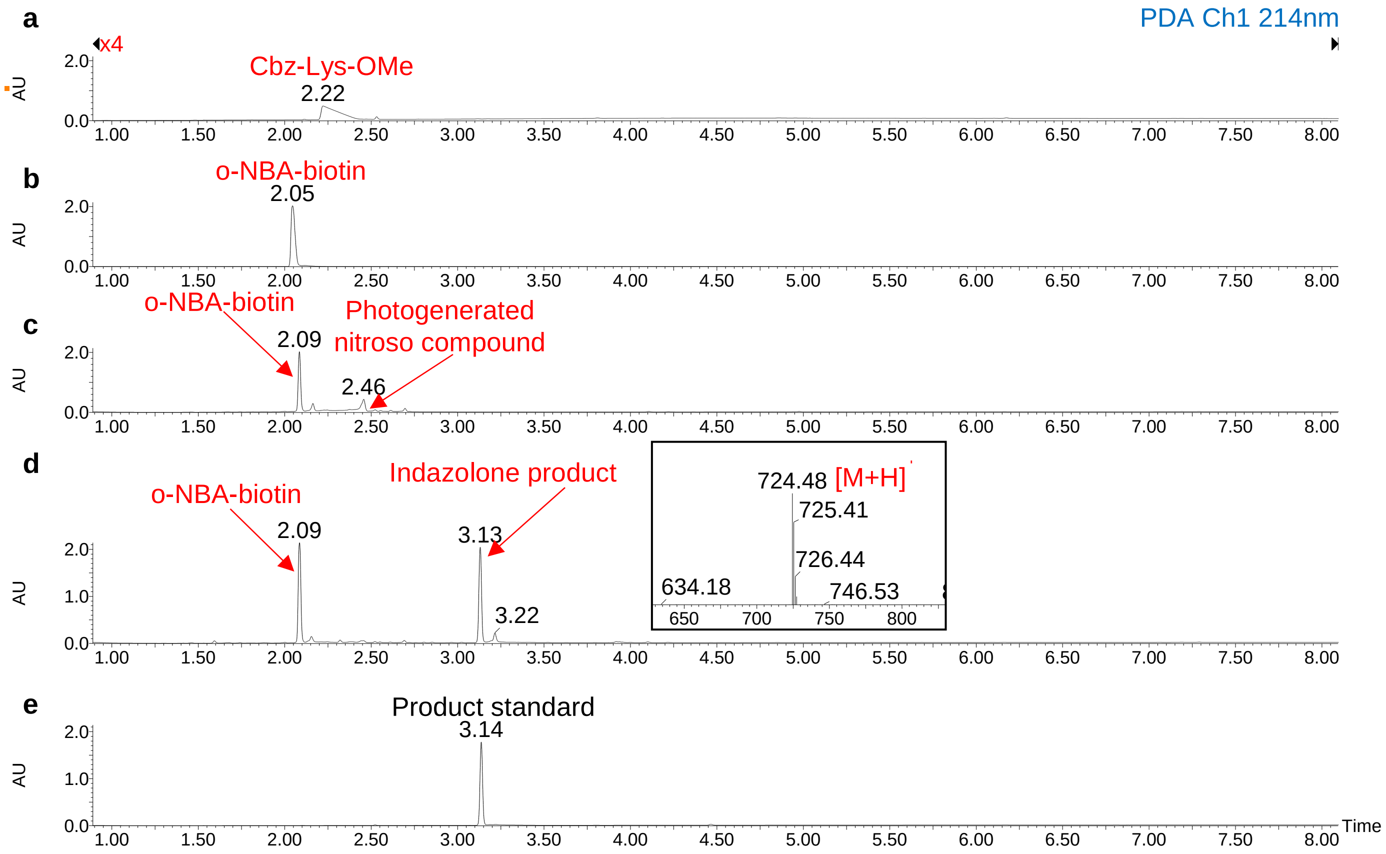

Supplementary Fig. 52 | a) Cbz-Lys-OMe (0.5 mM); b) o-NBA-biotin (2 mM); c) o-NBA-biotin (2 mM) was irradiated with 365 nm UV light; d) o-NBA-biotin (2 mM) photo-reacted with Cbz-Lys-OMe (0.5 mM); e) Product standard (0.5 mM) (NMR and HRMS data in Synthesis Part).

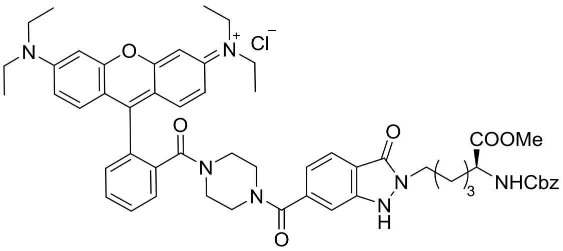

22

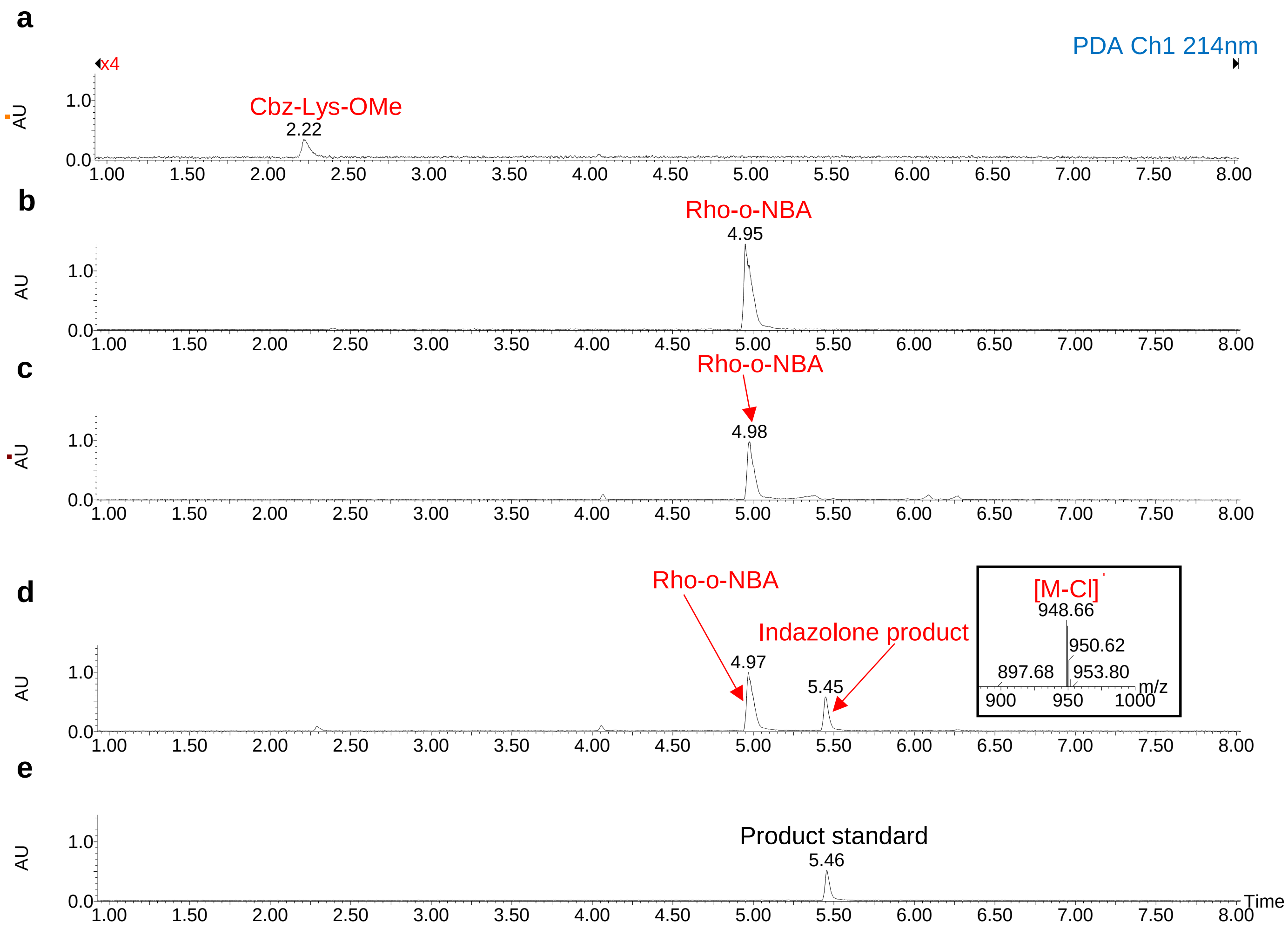

Supplementary Fig. 53 | a) Cbz-Lys-OMe (0.5 mM); b) Rho-o-NBA (2 mM); c) Rho-o-NBA (2 mM) was irradiated with 365 nm UV light; d) Rho-o-NBA (2 mM) photo-reacted with Cbz-Lys-OMe (0.5 mM); e) Product standard (0.5 mM) (NMR and HRMS data in Synthesis Part).

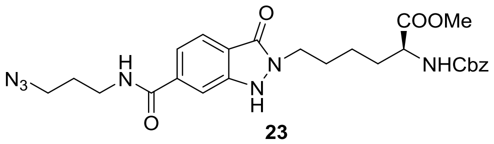

23

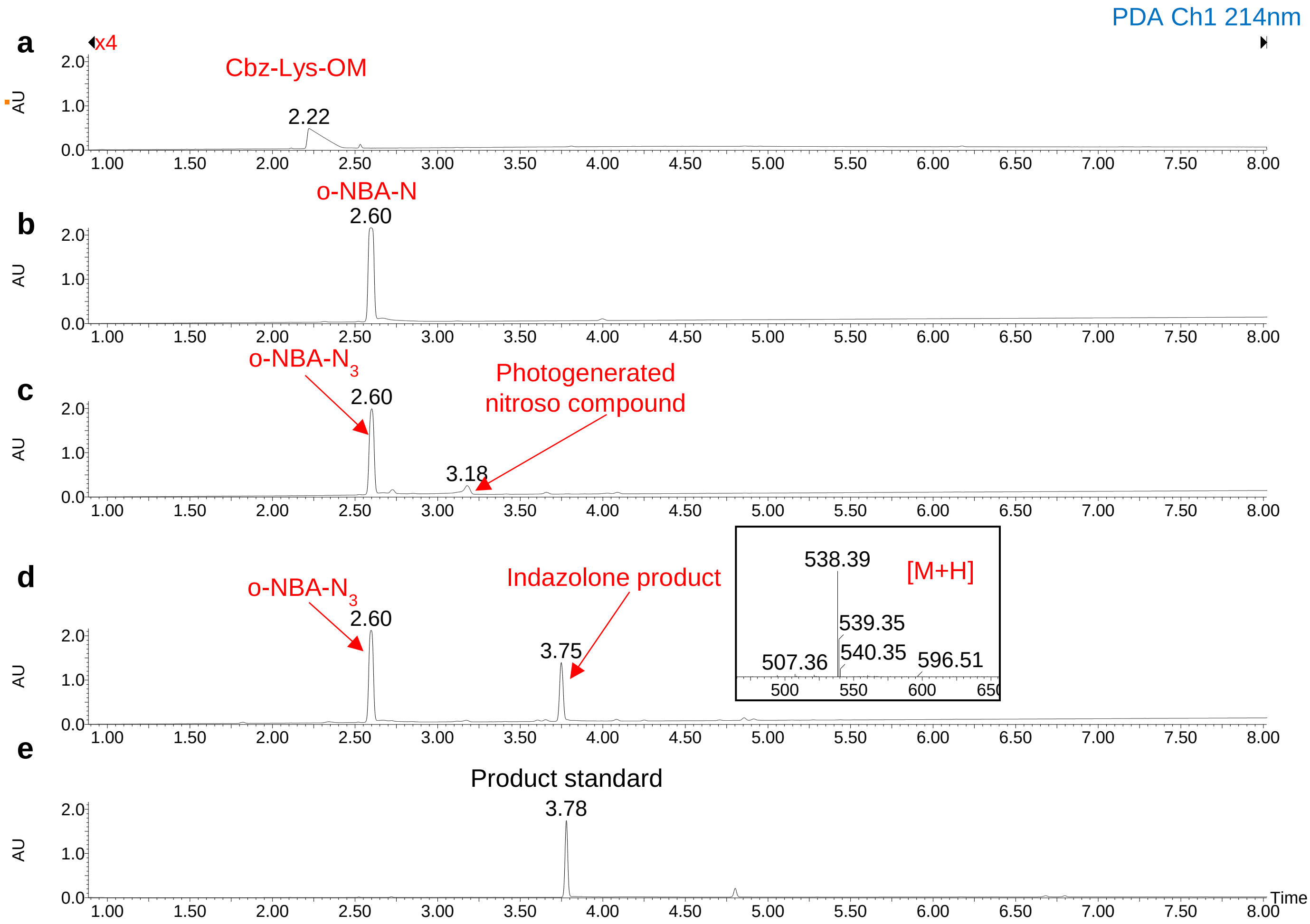

Supplementary Fig. 54 | a) Cbz-Lys-OMe (0.5 mM); b) o-NBA-N_3 (2 mM); c) o-NBA-N_3 (2 mM) was irradiated with 365 nm UV light; d) o-NBA-N_3 (2 mM) photo-reacted with Cbz-Lys-OMe (0.5 mM); e) Product standard (0.5 mM) (NMR and HRMS data in Synthesis Part)**.**

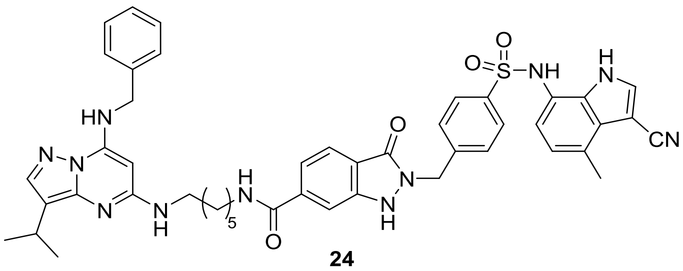

24

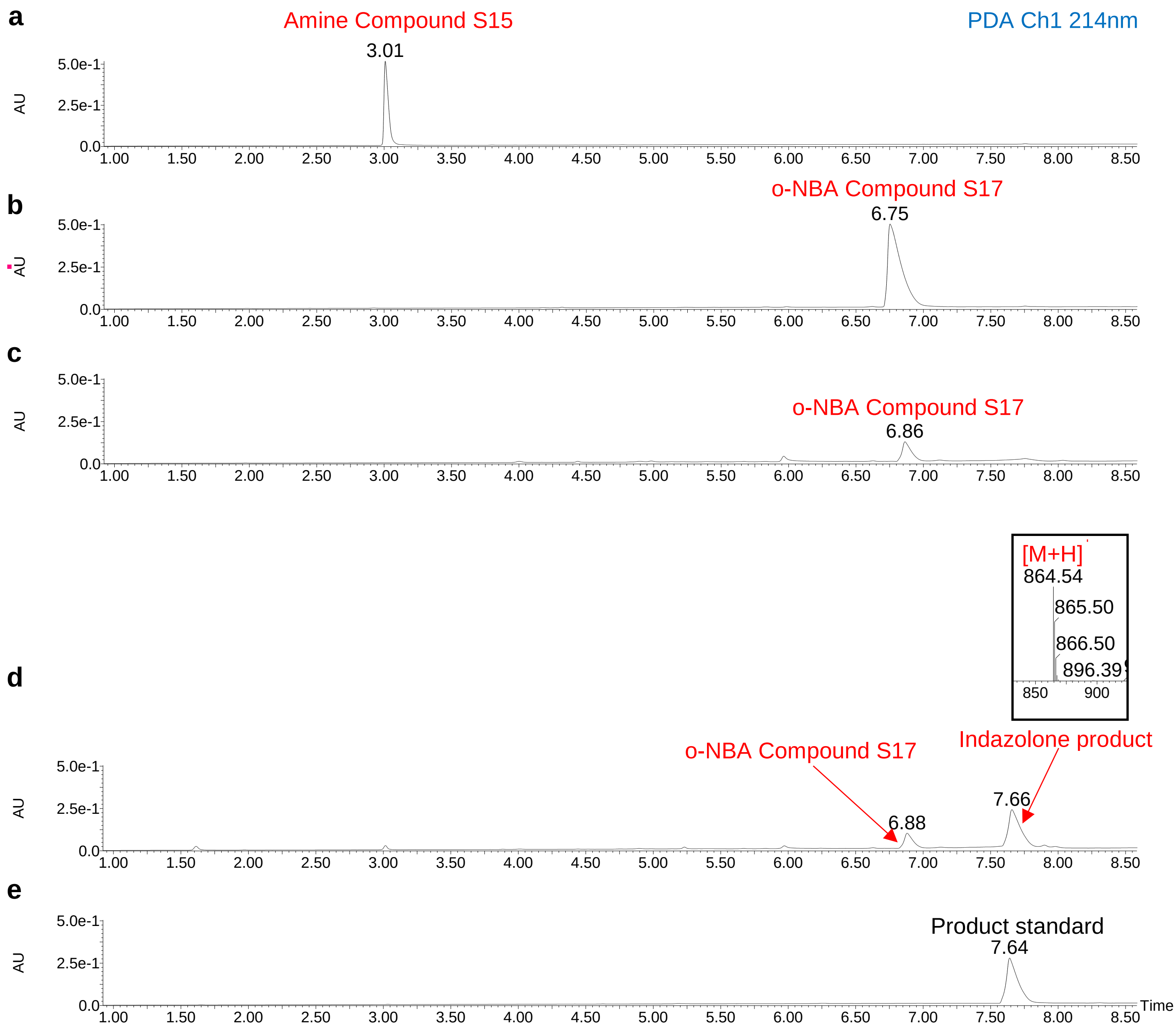

Supplementary Fig. 55 | a) Amine substrate (0.5 mM); b) compound S17(2 mM); c) compound S17(2 mM) was irradiated with 365 nm UV light; d) compound S17(2 mM) photo-reacted with amine substrate (0.5 mM); e) Product standard (0.5 mM) (NMR and MS data in Synthesis Part).

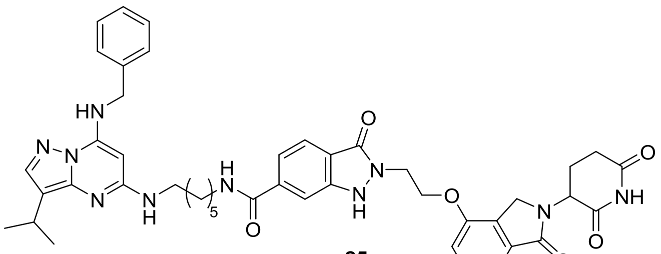

25

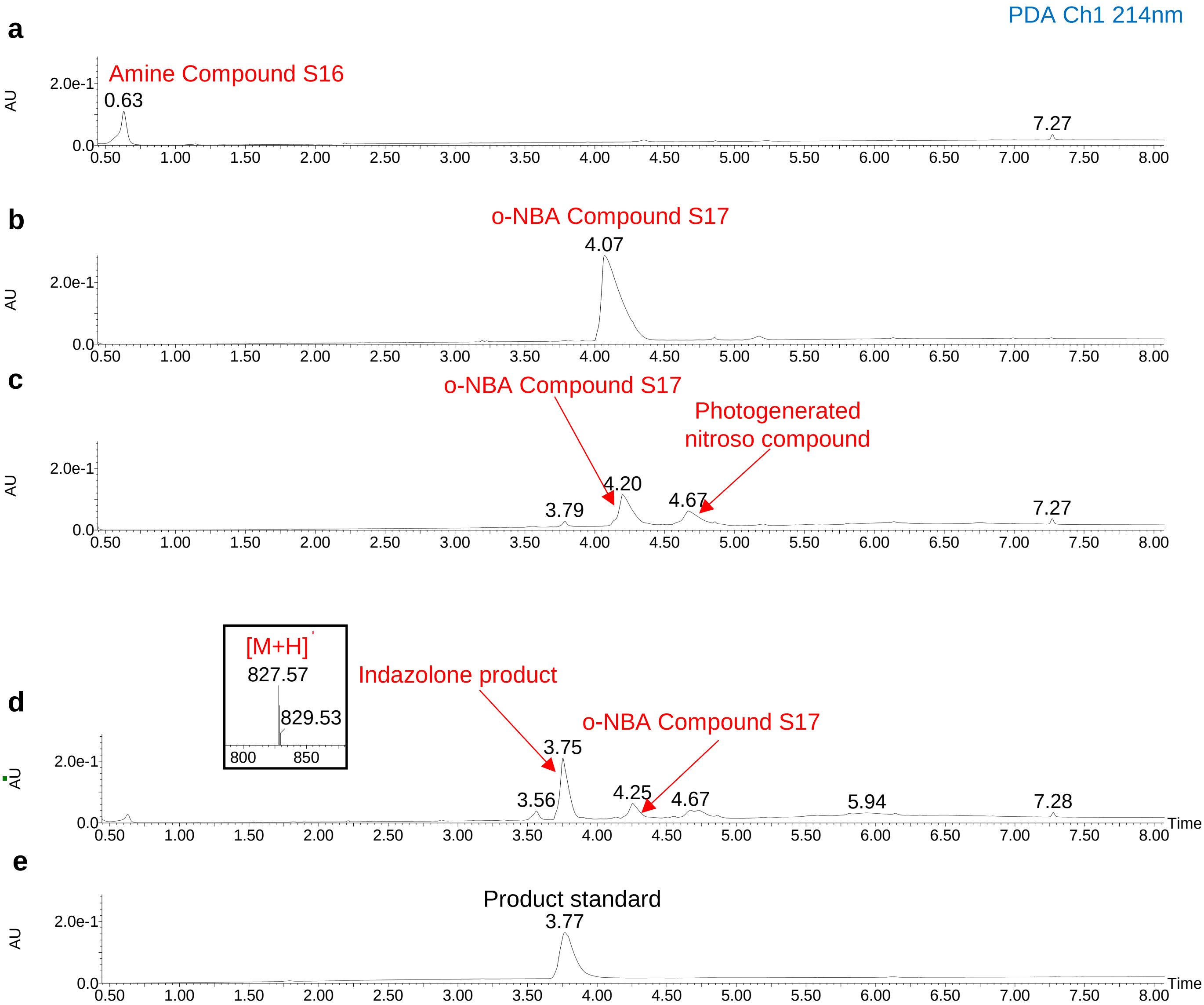

Supplementary Fig. 56 | a) Amine substrate (0.5 mM); b) Compound S17 (2 mM); c) Compound S17 (2 mM) was irradiated with 365 nm UV light; d) Compound S17 (2 mM) photo-reacted with amine substrate (0.5 mM); e) Product standard (0.5 mM) (NMR and MS data in Synthesis Part).

8. ^{1}H and ^{13}C NMR spectrum of Indazolone Product

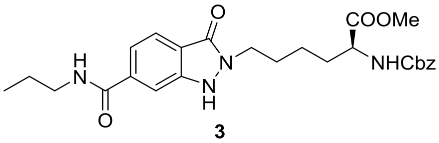

3

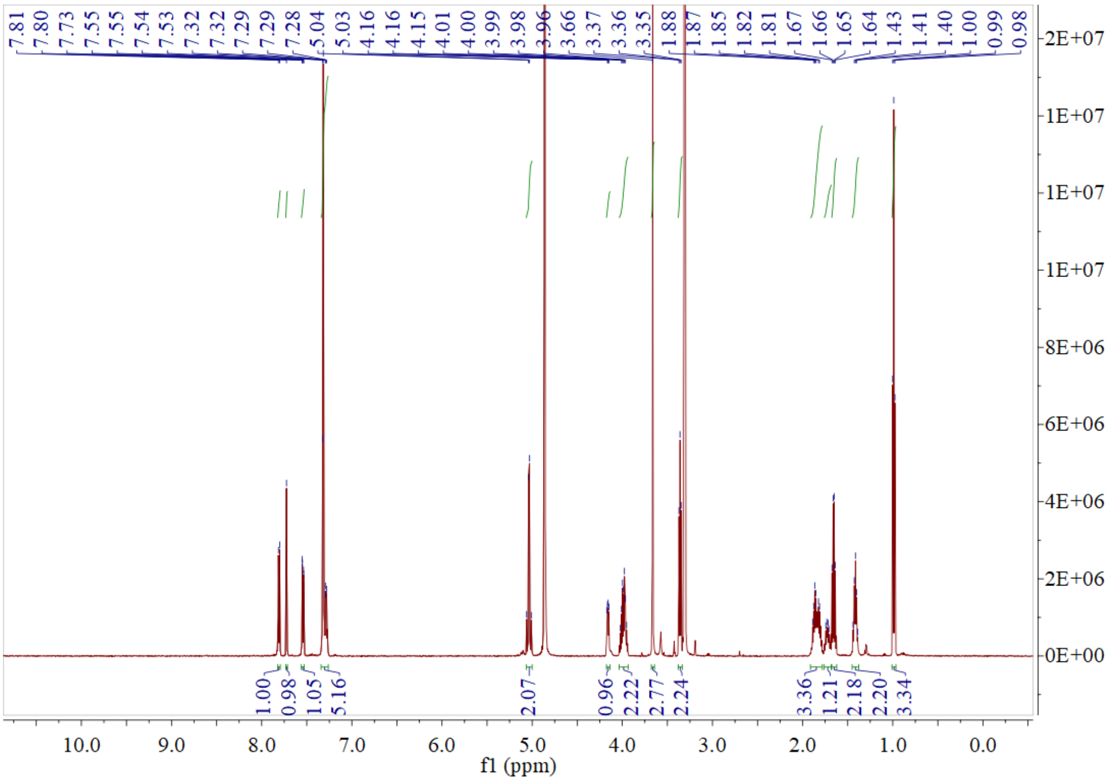

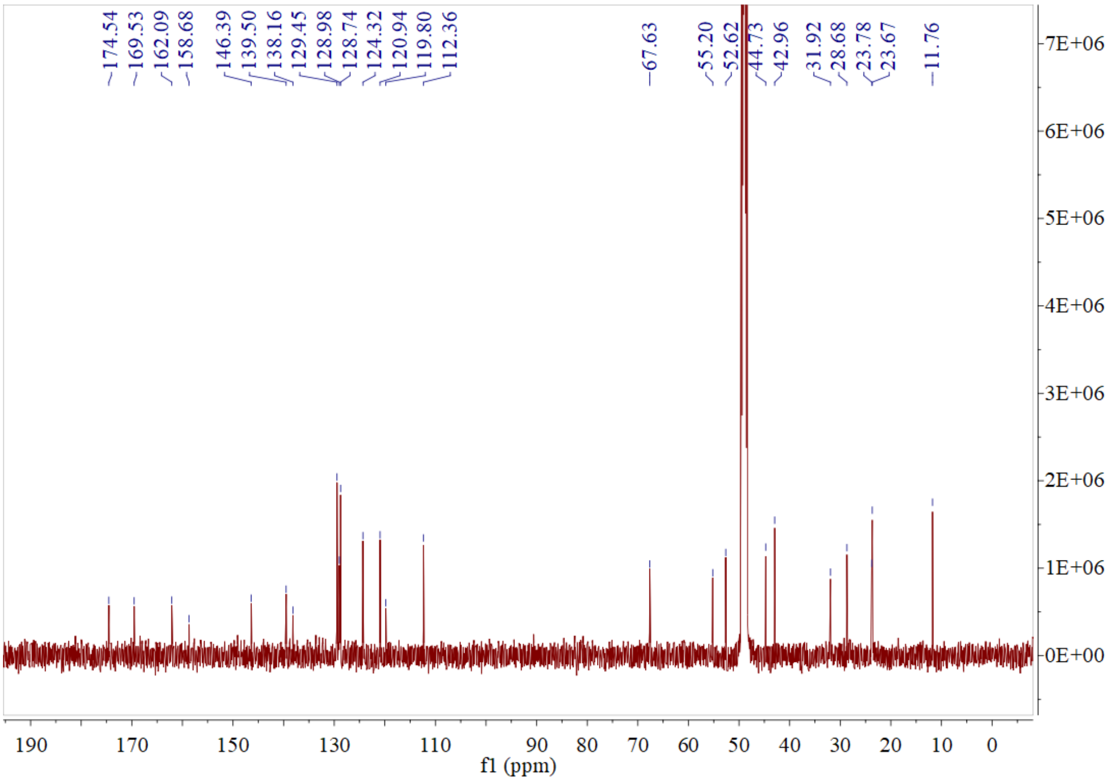

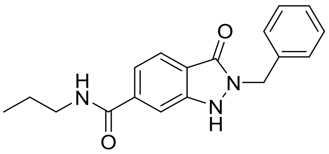
O
H
N
N
N
H
O

5

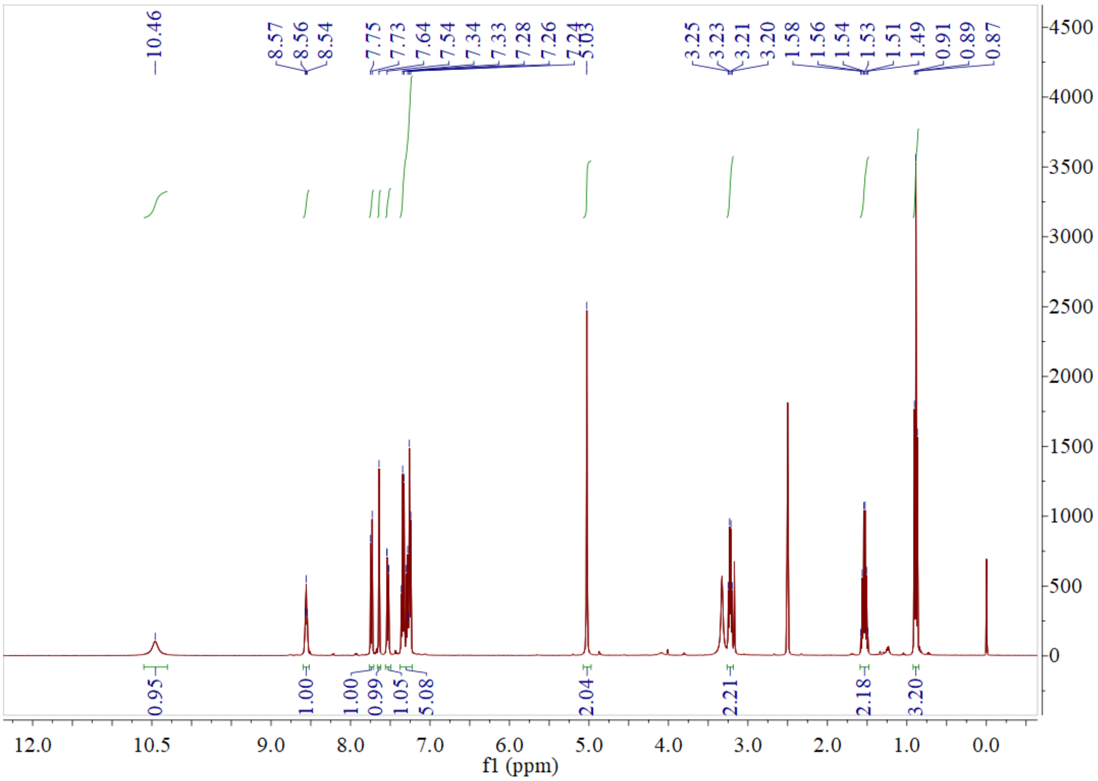
10.46
8.57
8.56
8.54
7.75
7.73
7.64
7.54
7.34
7.33
7.28
7.26
7.24
5.03
3.25
3.23
3.21
3.20
1.58
1.56
1.54
1.53
1.51
1.49
0.91
0.89
0.87
0.95
1.00
1.00
0.99
1.05
5.08
2.04
2.21
2.18
3.20
12.0
10.5
9.0
8.0
7.0
6.0
5.0
4.0
3.0
2.0
1.0
0.0
f1 (ppm)
4500
4000
3500
3000
2500
2000
1500
1000
500
0

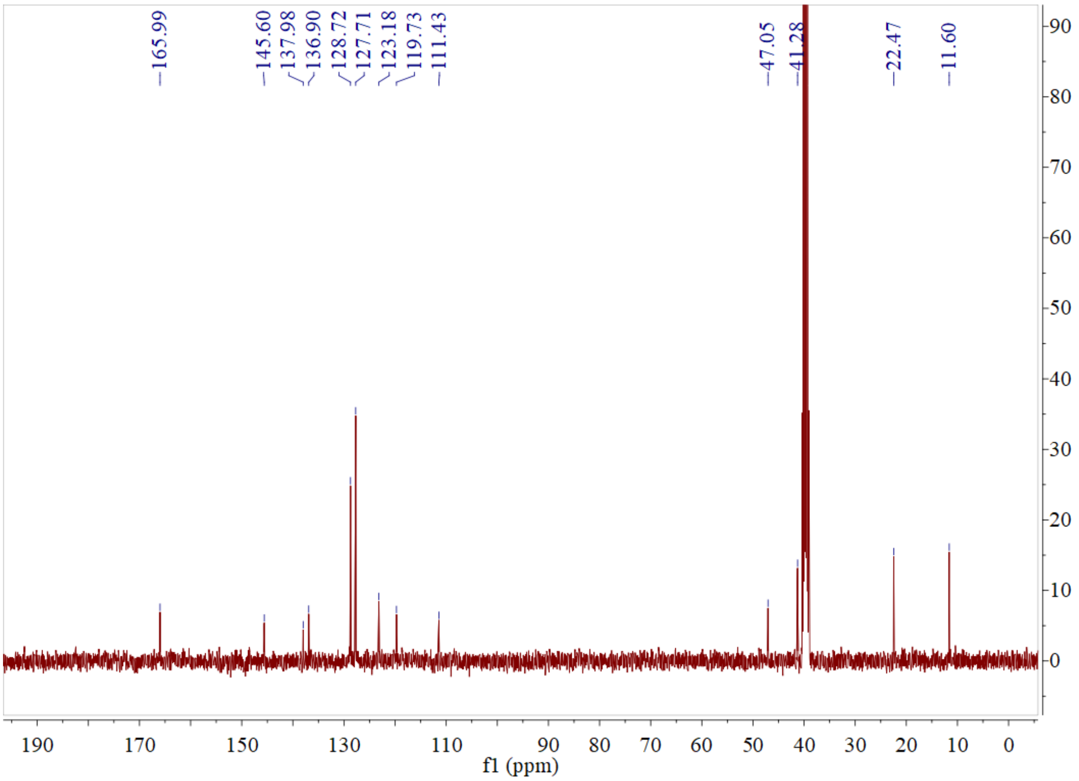
165.99
145.60
137.98
136.90
128.72
127.71
123.18
119.73
111.43
47.05
41.28
22.47
11.60
190
170
150
130
110
90
80
70
60
50
40
30
20
10
0
f1 (ppm)
90
80
70
60
50
40
30
20
10
0

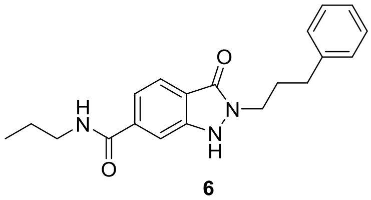
O
N
N
H
H
N
O

6

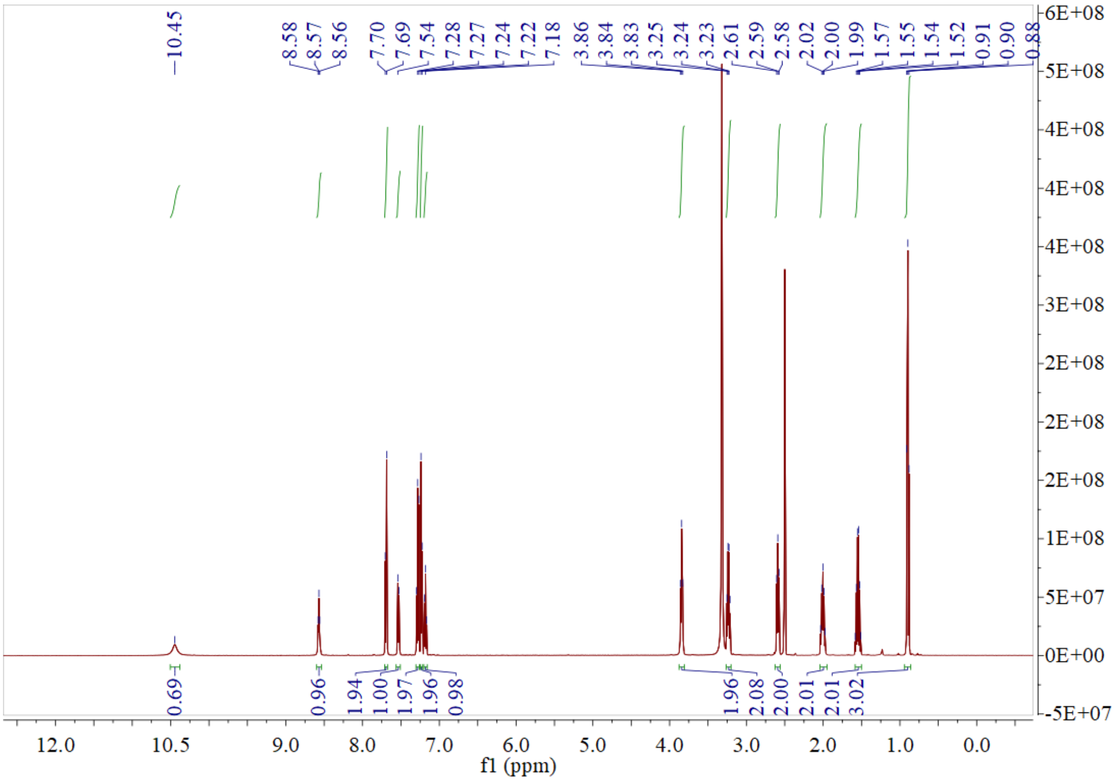
10.45
8.58
8.57
8.56
7.70
7.69
7.54
7.28
7.27
7.24
7.22
7.18
3.86
3.84
3.83
3.25
3.24
3.23
2.61
2.59
2.58
2.02
2.00
1.99
1.57
1.55
1.54
1.52
0.91
0.90
0.88
0.69
0.96
1.94
1.00
1.97
1.96
0.98
1.96
2.08
2.00
2.01
2.01
3.02
6E+08
5E+08
4E+08
4E+08
4E+08
3E+08
2E+08
2E+08
2E+08
1E+08
5E+07
0E+00
-5E+07
12.0
10.5
9.0
8.0
7.0
6.0
5.0
4.0
3.0
2.0
1.0
0.0
f1 (ppm)

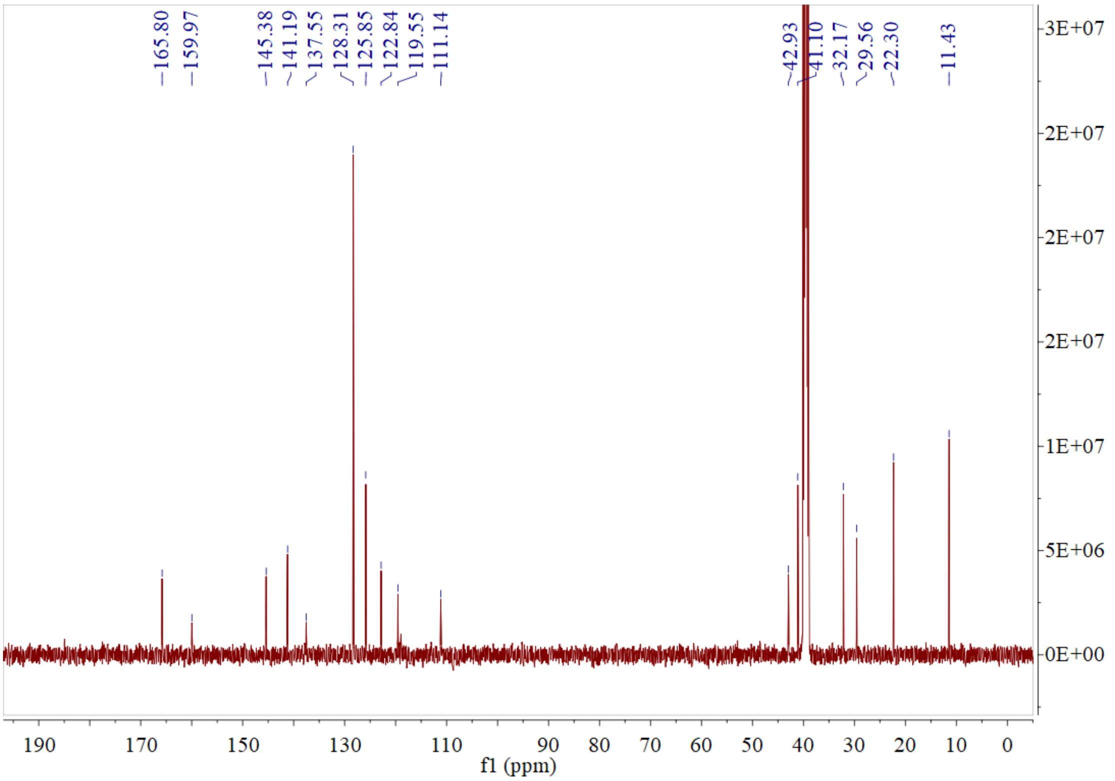
165.80
159.97
145.38
141.19
137.55
128.31
125.85
122.84
119.55
111.14
42.93
41.10
32.17
29.56
22.30
11.43
3E+07
2E+07
2E+07
2E+07
1E+07
5E+06
0E+00
190
170
150
130
110
90
80
70
60
50
40
30
20
10
0
f1 (ppm)

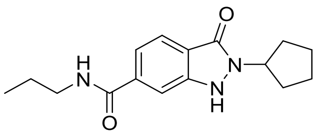
O
N
H
N
H
N
O

7

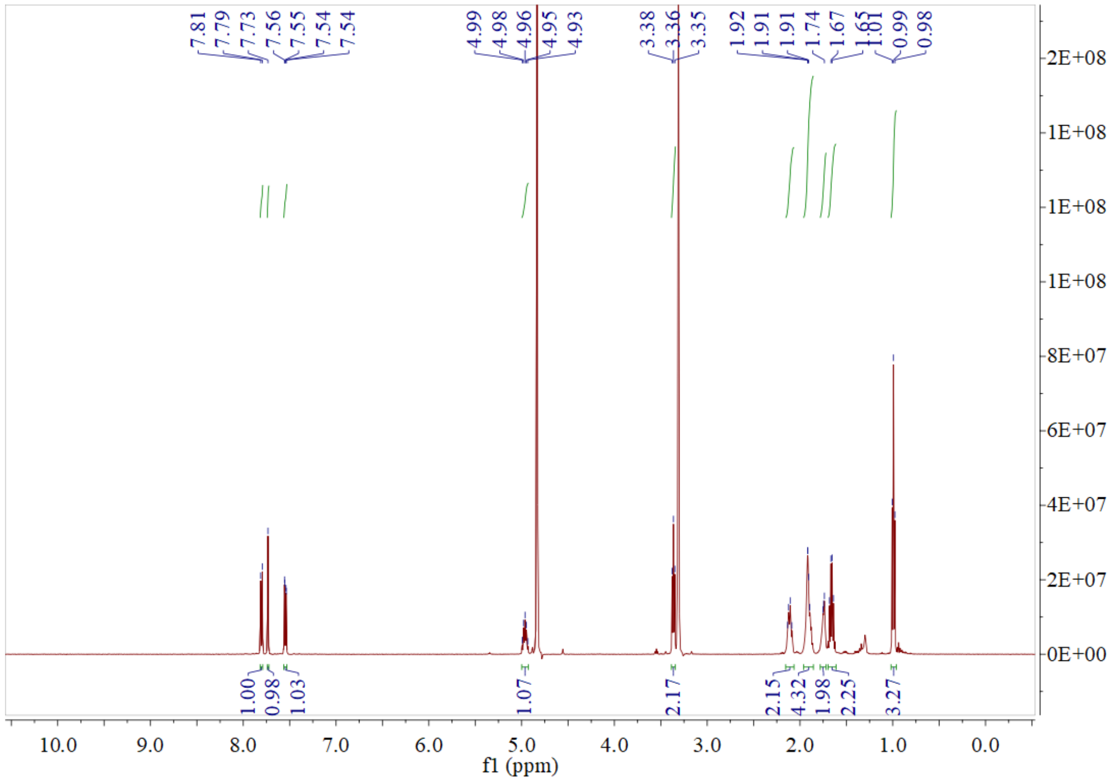
7.81
7.79
7.73
7.56
7.55
7.54
7.54
4.99
4.98
4.96
4.95
4.93
3.38
3.36
3.35
1.92
1.91
1.91
1.74
1.67
1.65
1.61
0.99
0.98
1.00
0.98
1.03
1.07
2.17
2.15
4.32
1.98
2.25
3.27
f1 (ppm)

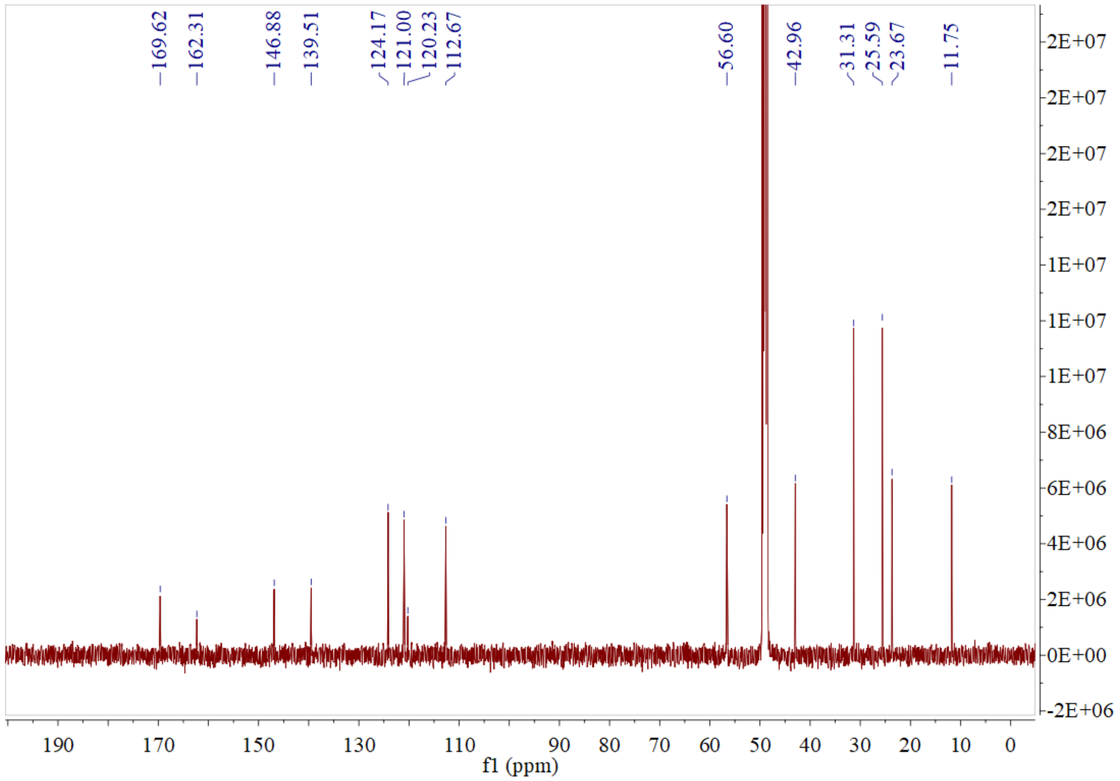
169.62
162.31
146.88
139.51
124.17
121.00
120.23
112.67
56.60
42.96
31.31
25.59
23.67
11.75
f1 (ppm)

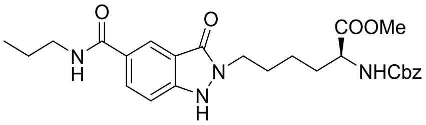

O
O
COOMe
N
H
N
N
H
NHCbz

8

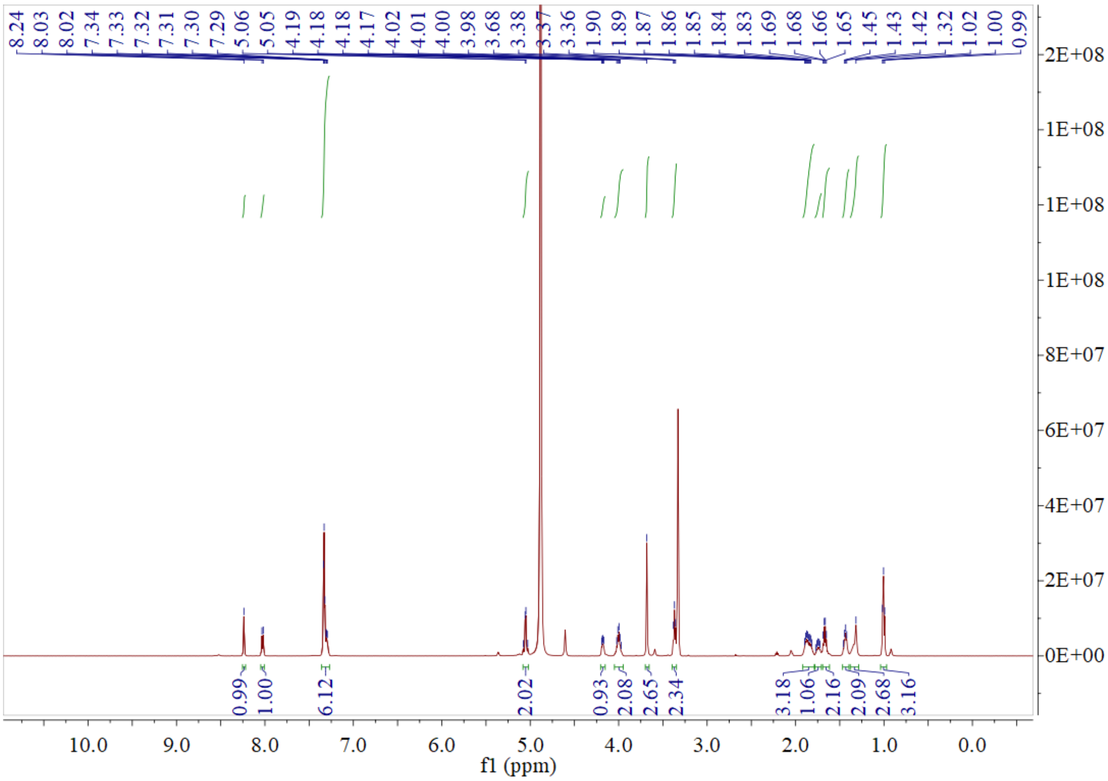

8.24 8.03 8.02 7.34 7.33 7.32 7.31 7.30 7.29 5.06 5.05 4.19 4.18 4.18 4.17 4.02 4.01 4.00 3.98 3.68 3.38 3.37 3.36 1.90 1.89 1.87 1.86 1.85 1.84 1.83 1.69 1.68 1.66 1.65 1.45 1.43 1.42 1.32 1.02 1.00 0.99
0.99 1.00 6.12 2.02 0.93 2.08 2.65 2.34 3.18 1.06 2.16 2.09 2.68 3.16
2E+08
1E+08
1E+08
1E+08
8E+07
6E+07
4E+07
2E+07
0E+00
10.0 9.0 8.0 7.0 6.0 5.0 4.0 3.0 2.0 1.0 0.0
f1 (ppm)

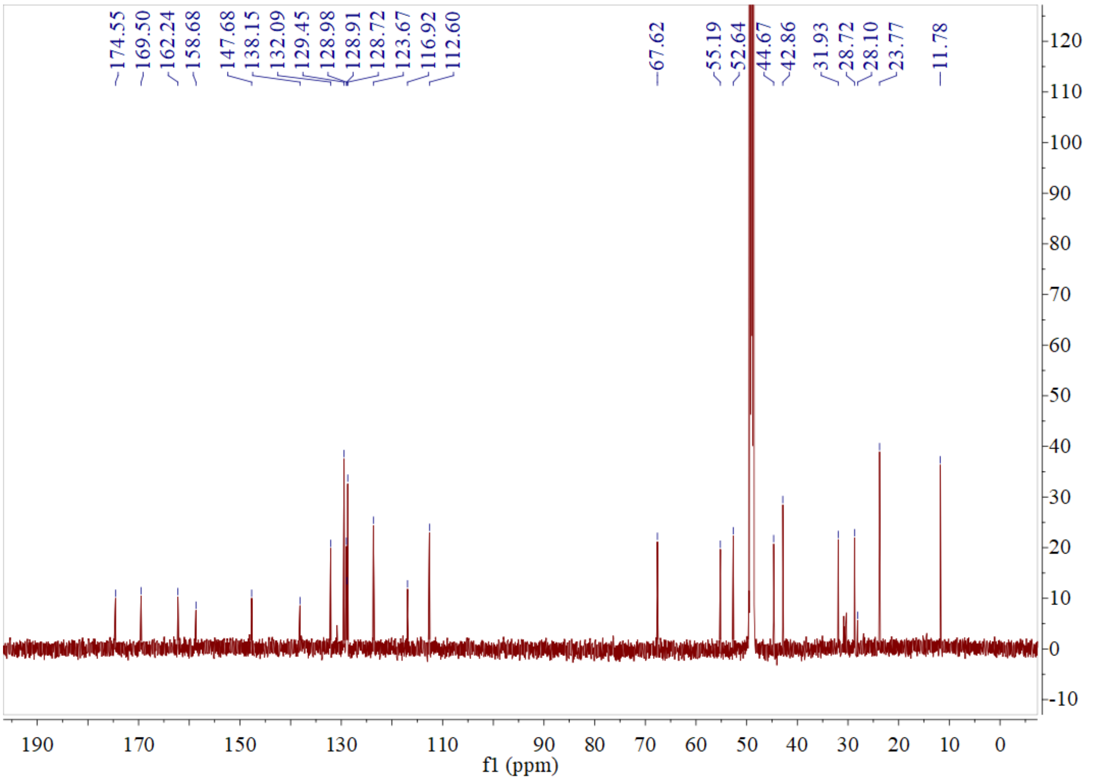

174.55 169.50 162.24 158.68 147.68 138.15 132.09 129.45 128.98 128.91 128.72 123.67 116.92 112.60 67.62 55.19 52.64 44.67 42.86 31.93 28.72 28.10 23.77 11.78
120
110
100
90
80
70
60
50
40
30
20
10
0
-10
190 170 150 130 110 90 80 70 60 50 40 30 20 10 0
f1 (ppm)

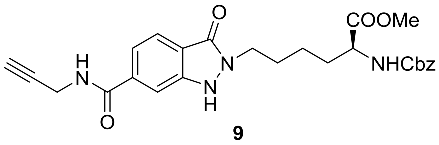

9

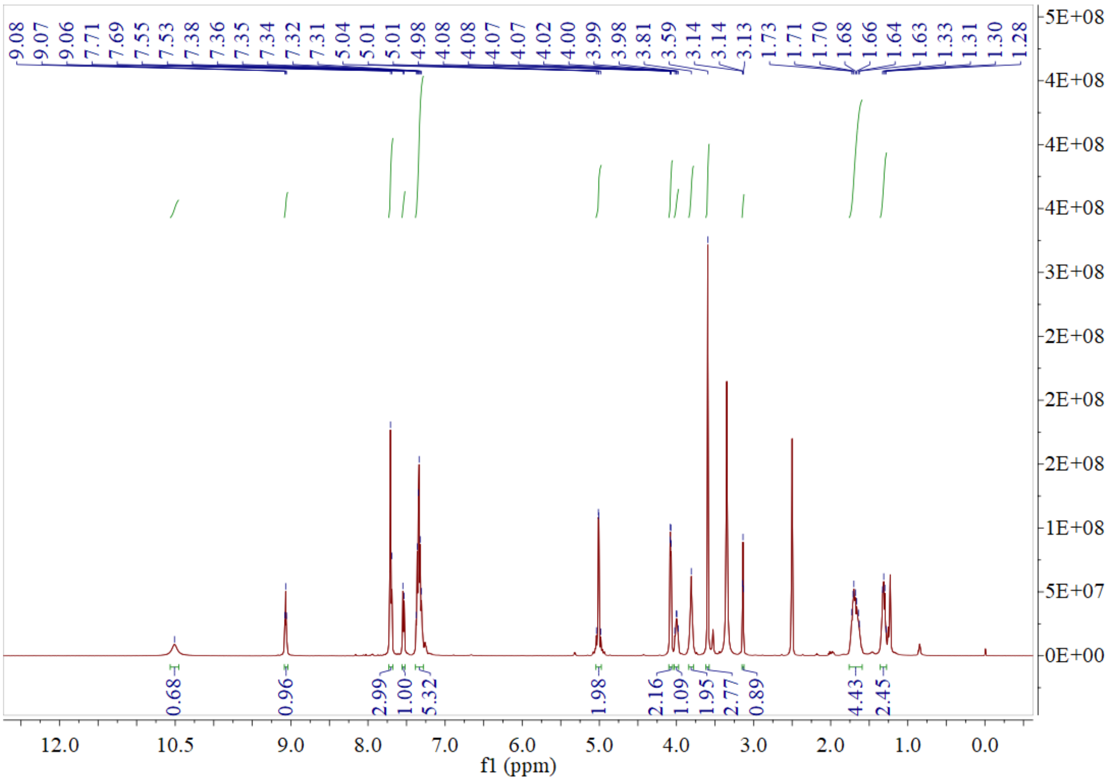

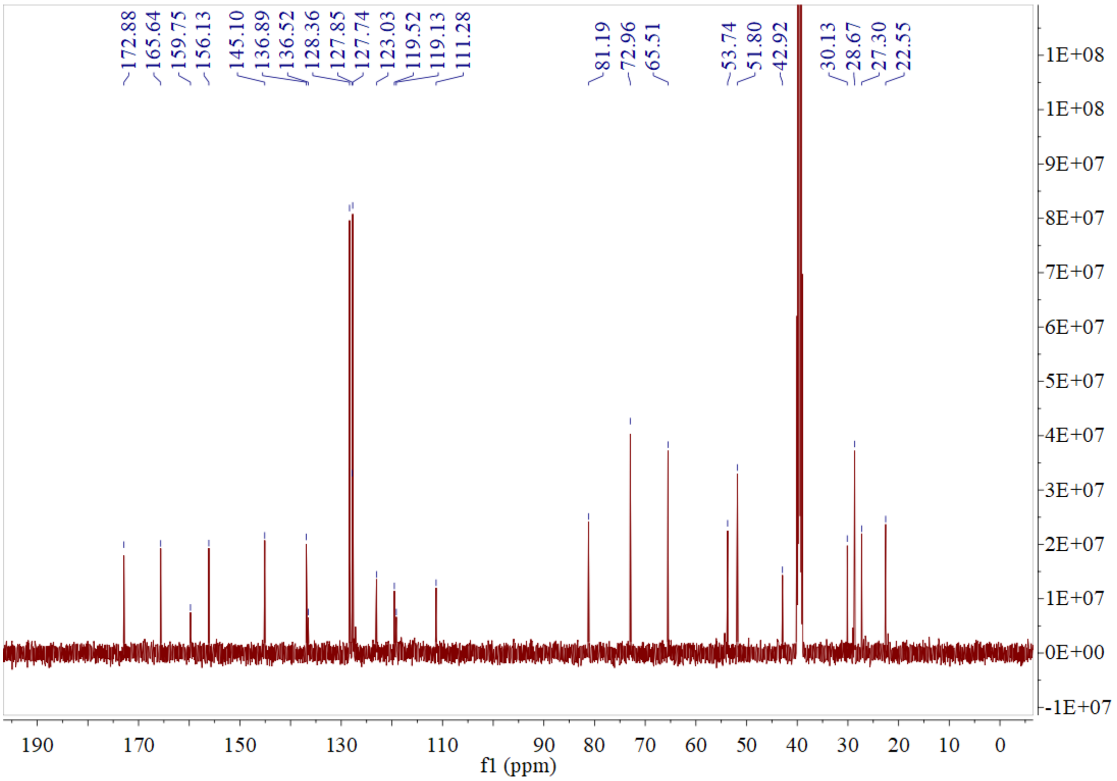

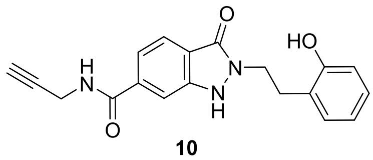

10

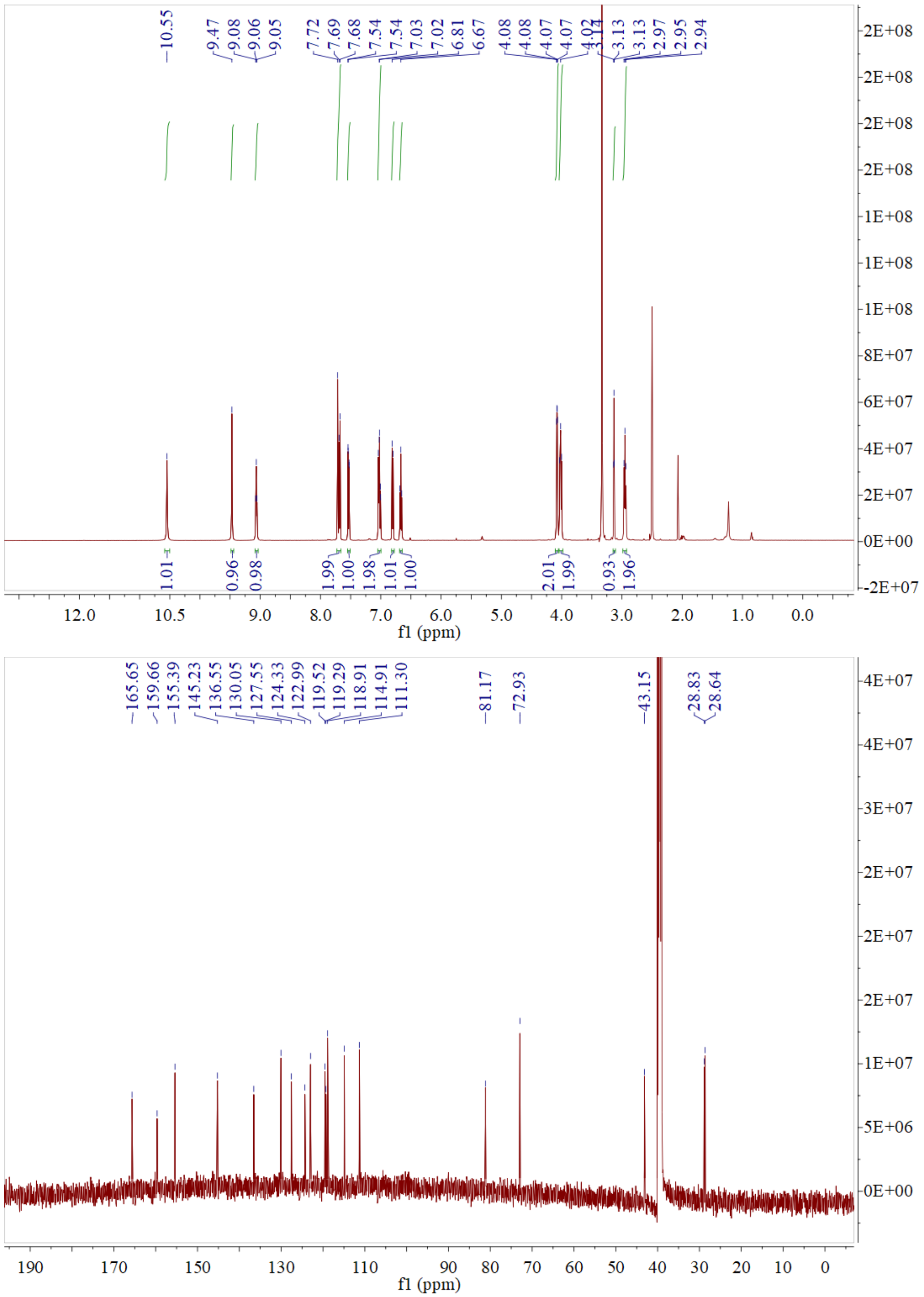

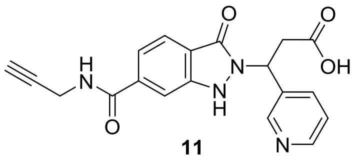

11

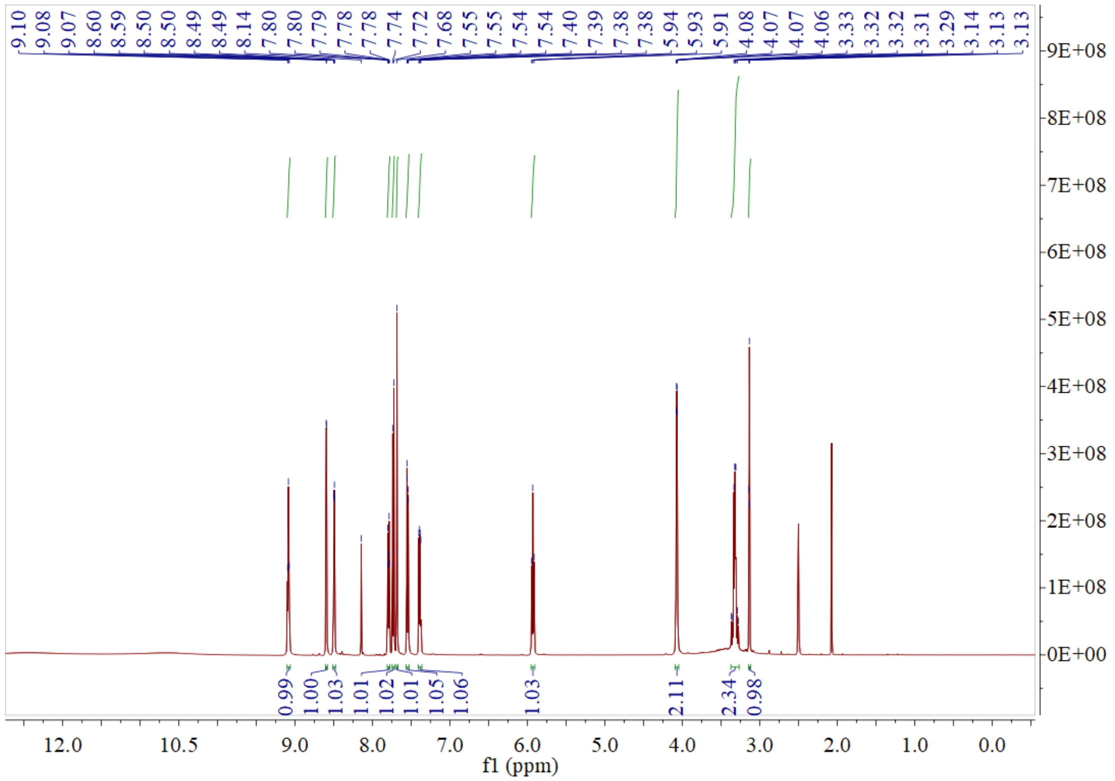

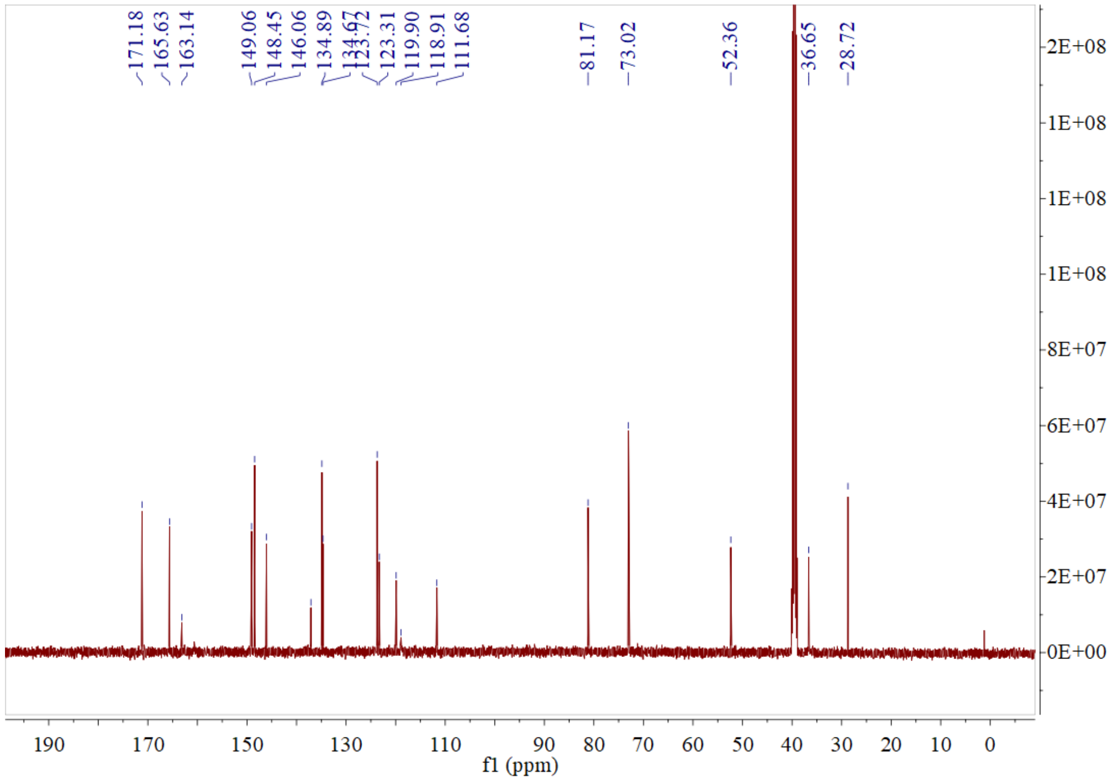

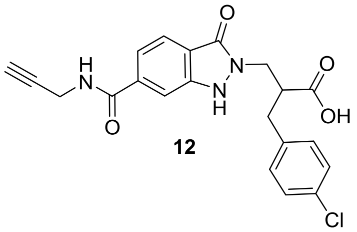
O
H
N
N
N
H
O
OH
O
Cl

12

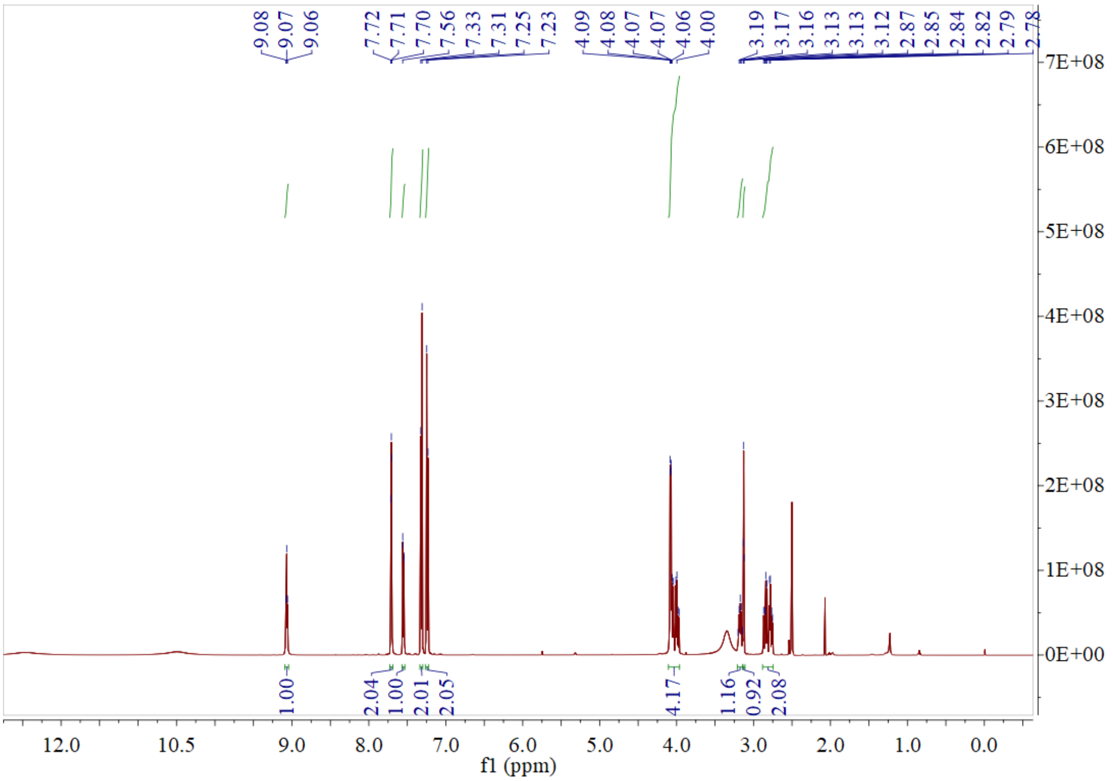
9.08
9.07
9.06
7.72
7.71
7.70
7.56
7.33
7.31
7.25
7.23
4.09
4.08
4.07
4.07
4.06
4.00
3.19
3.17
3.16
3.13
3.13
3.12
2.87
2.85
2.84
2.82
2.79
2.78
1.00
2.04
1.00
2.01
2.05
4.17
1.16
0.92
2.08
7E+08
6E+08
5E+08
4E+08
3E+08
2E+08
1E+08
0E+00
12.0
10.5
9.0
8.0
7.0
6.0
5.0
4.0
3.0
2.0
1.0
0.0
f1 (ppm)

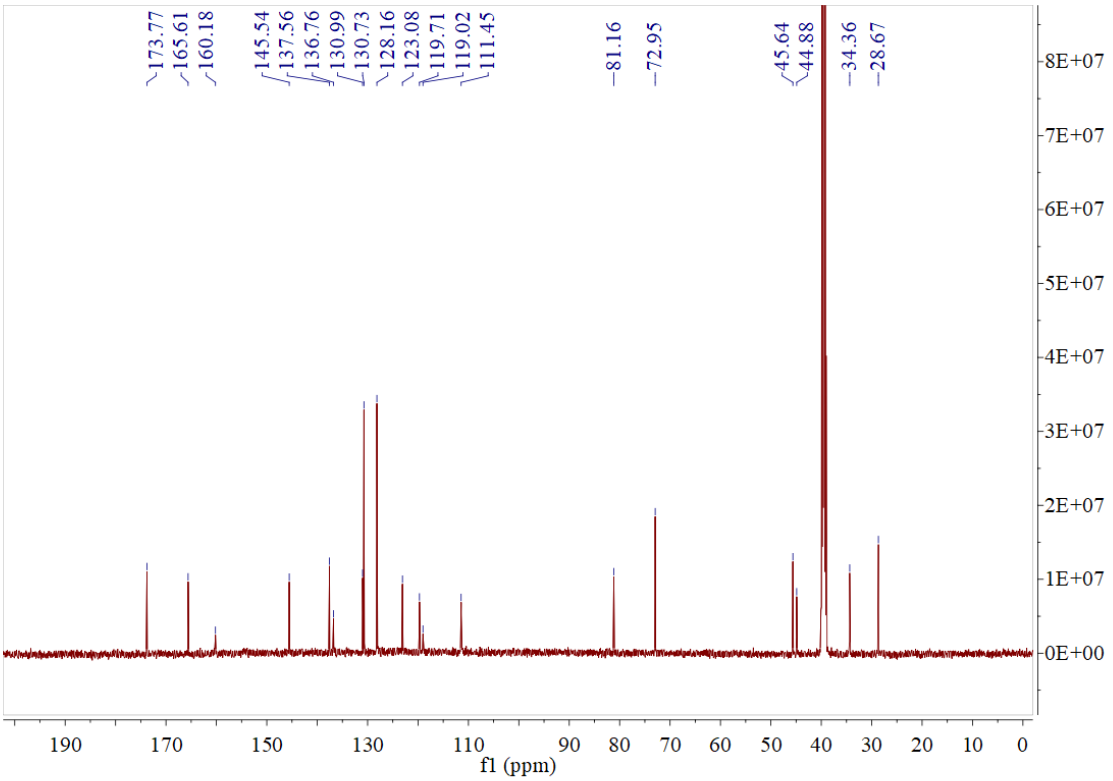
173.77
165.61
160.18
145.54
137.56
136.76
130.99
130.73
128.16
123.08
119.71
119.02
111.45
81.16
72.95
45.64
44.88
34.36
28.67
8E+07
7E+07
6E+07
5E+07
4E+07
3E+07
2E+07
1E+07
0E+00
190
170
150
130
110
90
80
70
60
50
40
30
20
10
0
f1 (ppm)

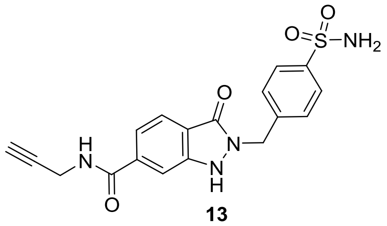

13

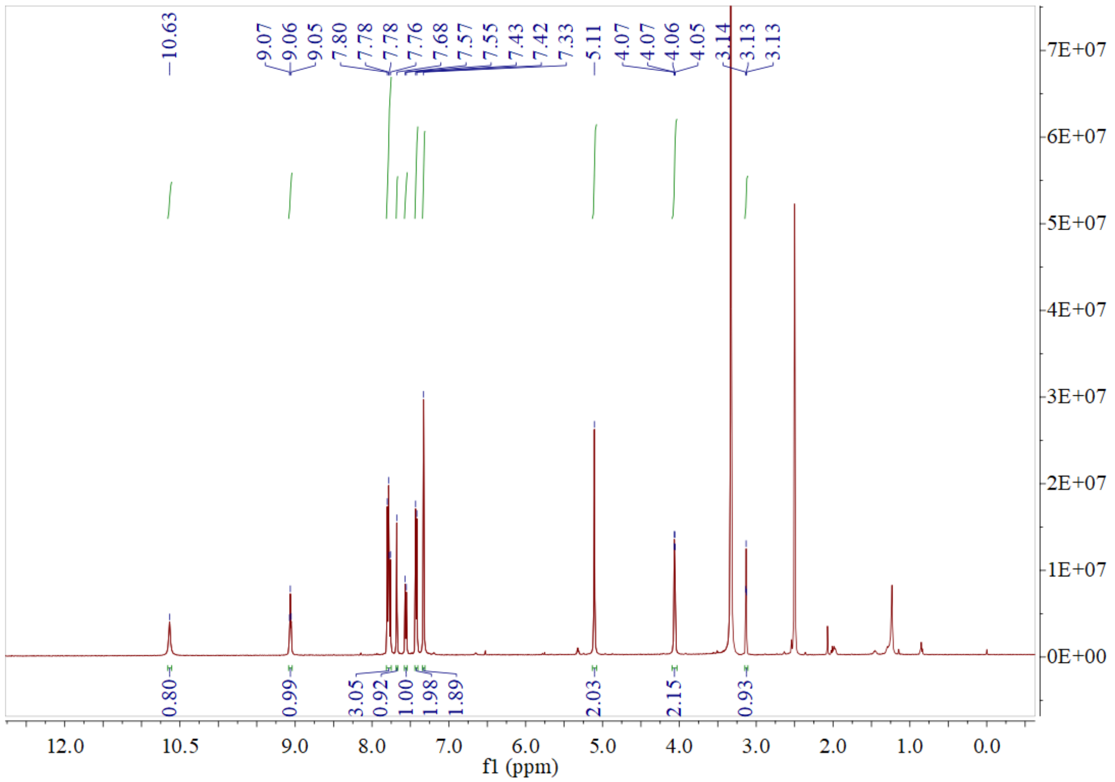

10.63
9.07
9.06
9.05
7.80
7.78
7.78
7.76
7.68
7.57
7.55
7.43
7.42
7.33
5.11
4.07
4.07
4.06
4.05
3.14
3.13
3.13
0.80
0.99
3.05
0.92
1.00
1.98
1.89
2.03
2.15
0.93
7E+07
6E+07
5E+07
4E+07
3E+07
2E+07
1E+07
0E+00
12.0
10.5
9.0
8.0
7.0
6.0
5.0
4.0
3.0
2.0
1.0
0.0
f1 (ppm)

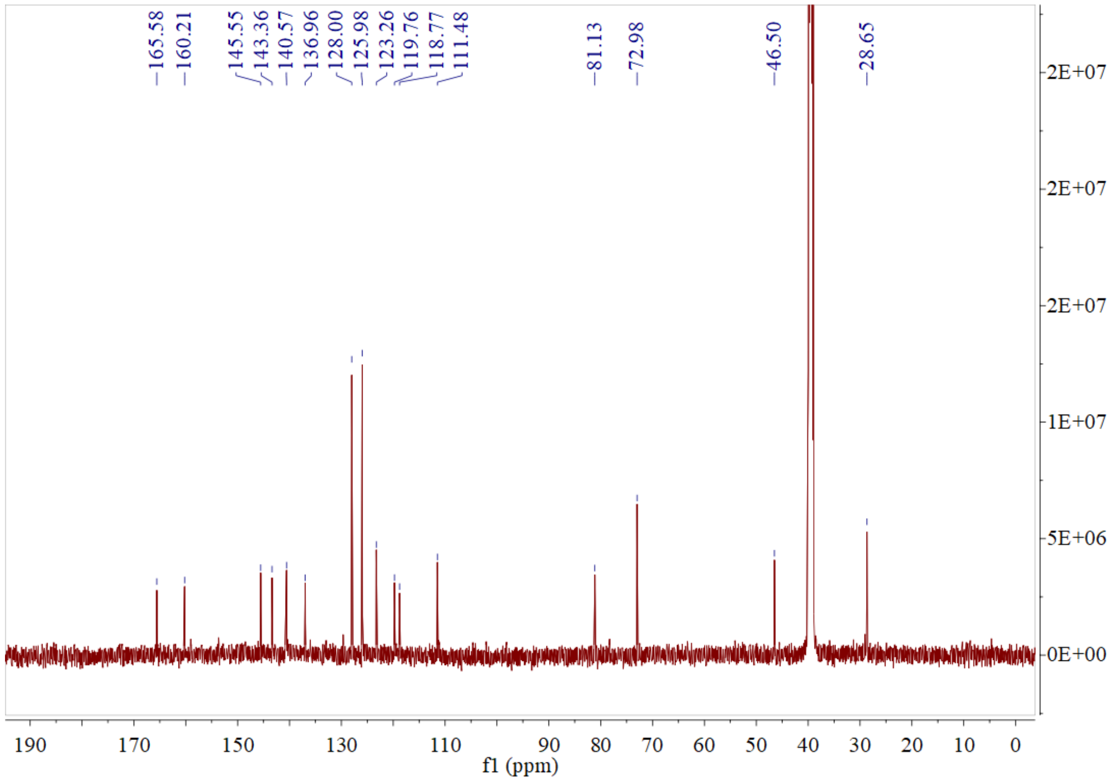

165.58
160.21
145.55
143.36
140.57
136.96
128.00
125.98
123.26
119.76
118.77
111.48
81.13
72.98
46.50
28.65
2E+07
2E+07
2E+07
1E+07
5E+06
0E+00
190
170
150
130
110
90
80
70
60
50
40
30
20
10
0
f1 (ppm)

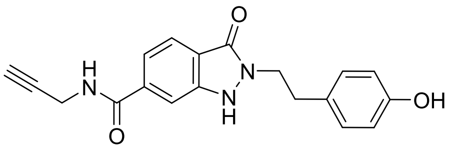

14

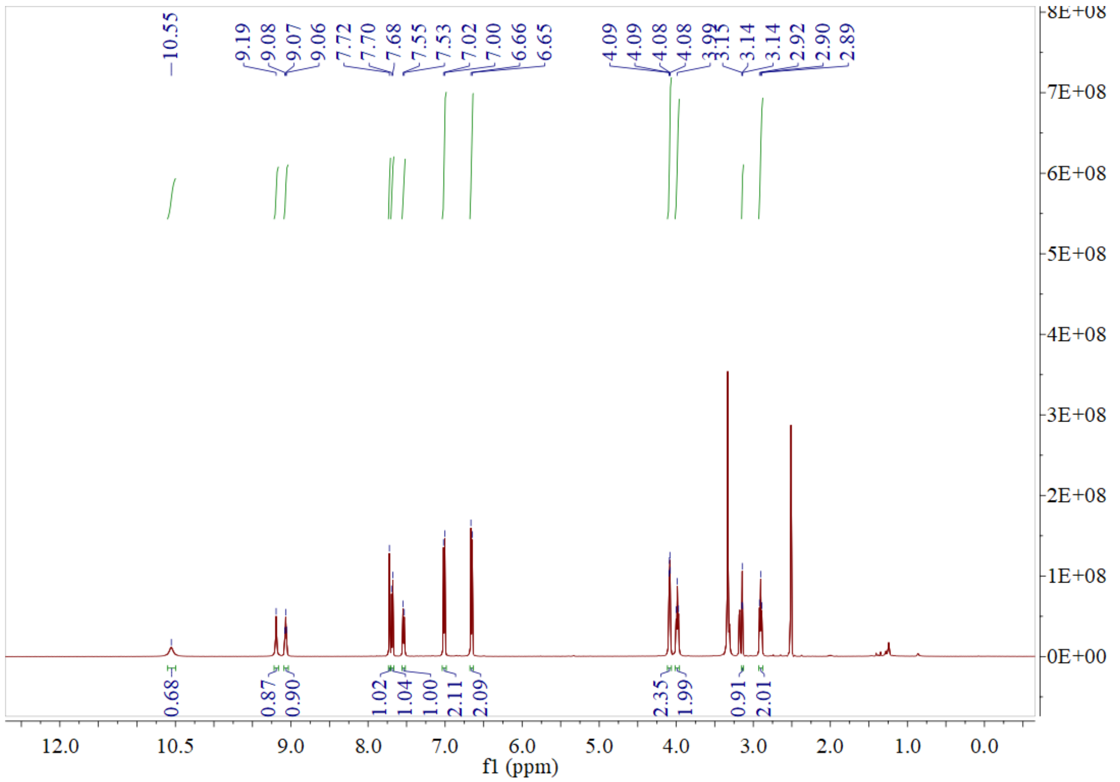

10.55
9.19
9.08
9.07
9.06
7.72
7.70
7.68
7.55
7.53
7.02
7.00
6.66
6.65
4.09
4.09
4.08
4.08
3.99
3.15
3.14
3.14
2.92
2.90
2.89
0.68
0.87
0.90
1.02
1.04
1.00
2.11
2.09
2.35
1.99
0.91
2.01
f1 (ppm)

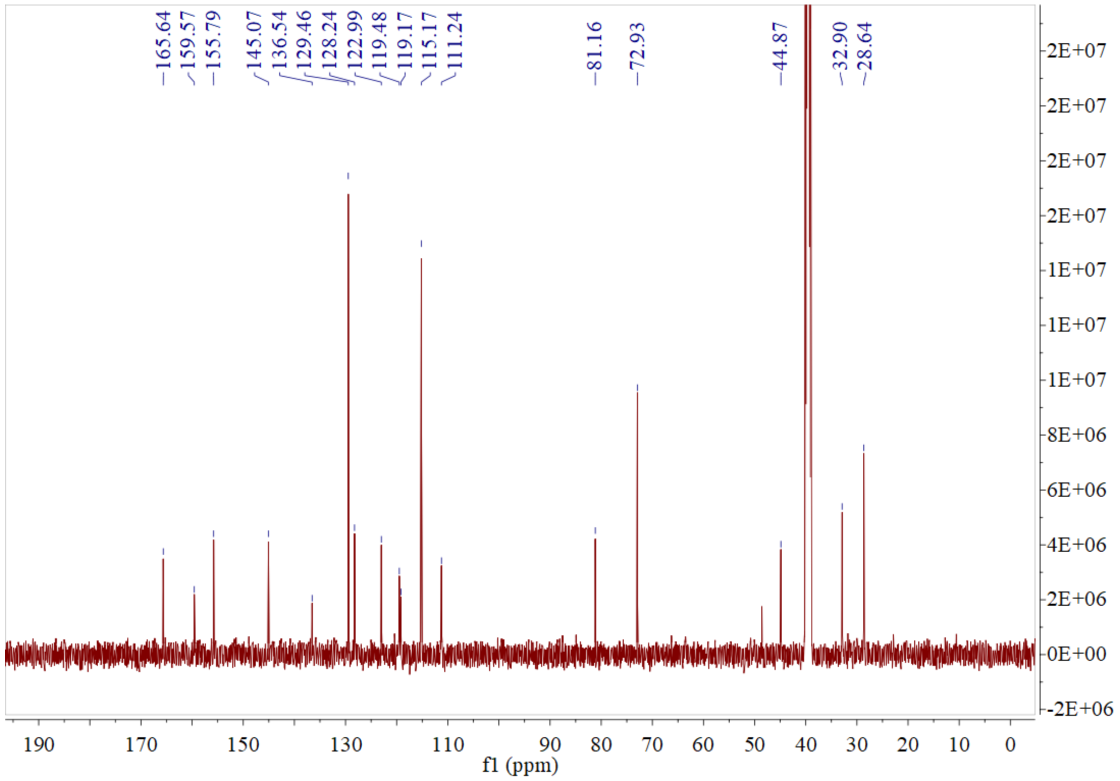

165.64
159.57
155.79
145.07
136.54
129.46
128.24
122.99
119.48
119.17
115.17
111.24
81.16
72.93
44.87
32.90
28.64
f1 (ppm)

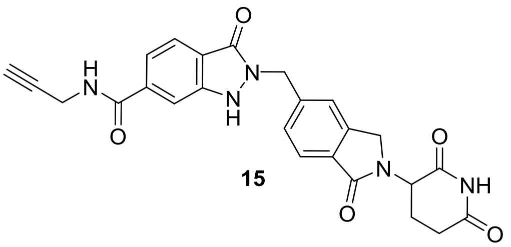
O
H
N
N
N
H
O
N
O
O
NH
O

15

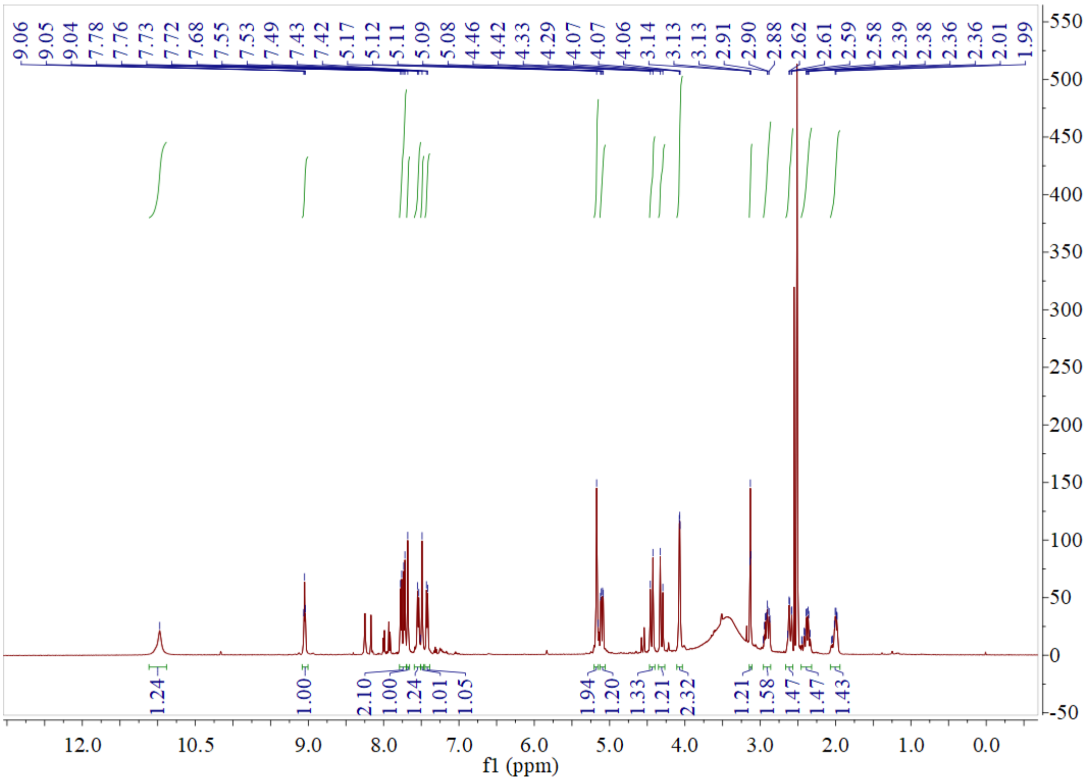
9.06 9.05 9.04 7.78 7.76 7.73 7.72 7.68 7.55 7.53 7.49 7.43 7.42 5.17 5.12 5.11 5.09 5.08 4.46 4.42 4.33 4.29 4.07 4.07 4.06 3.14 3.13 3.13 2.91 2.90 2.88 2.62 2.61 2.59 2.58 2.39 2.38 2.36 2.36 2.01 1.99
1.24 1.00 2.10 1.00 1.24 1.01 1.05 1.94 1.20 1.33 1.21 2.32 1.21 1.58 1.47 1.47 1.43
12.0 10.5 9.0 8.0 7.0 6.0 5.0 4.0 3.0 2.0 1.0 0.0
f1 (ppm)
550 500 450 400 350 300 250 200 150 100 50 0 -50

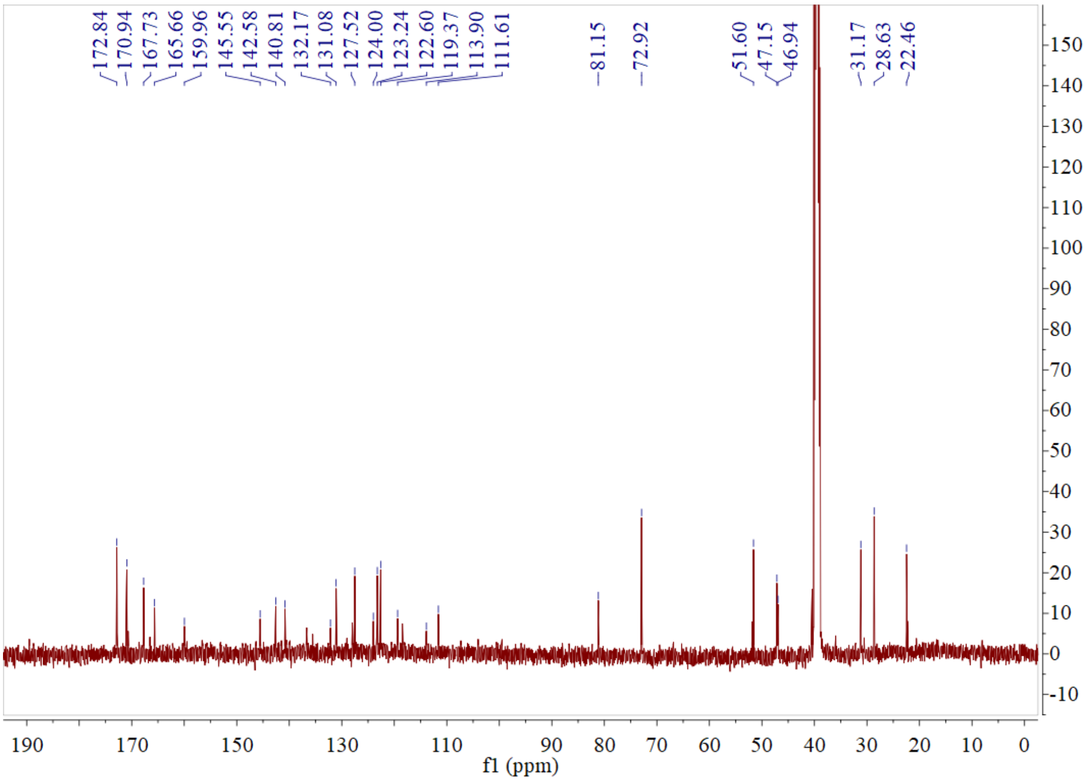
172.84 170.94 167.73 165.66 159.96 145.55 142.58 140.81 132.17 131.08 127.52 124.00 123.24 122.60 119.37 113.90 111.61
81.15 72.92
51.60 47.15 46.94
31.17 28.63 22.46
190 170 150 130 110 90 80 70 60 50 40 30 20 10 0
f1 (ppm)
150 140 130 120 110 100 90 80 70 60 50 40 30 20 10 0 -10

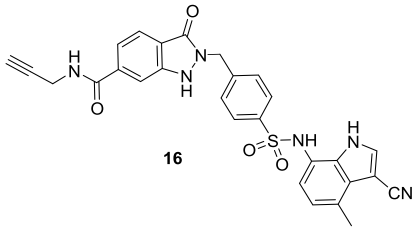
O
N
H
N
N
H
O
O
S
O
NH
H
N
CN

16

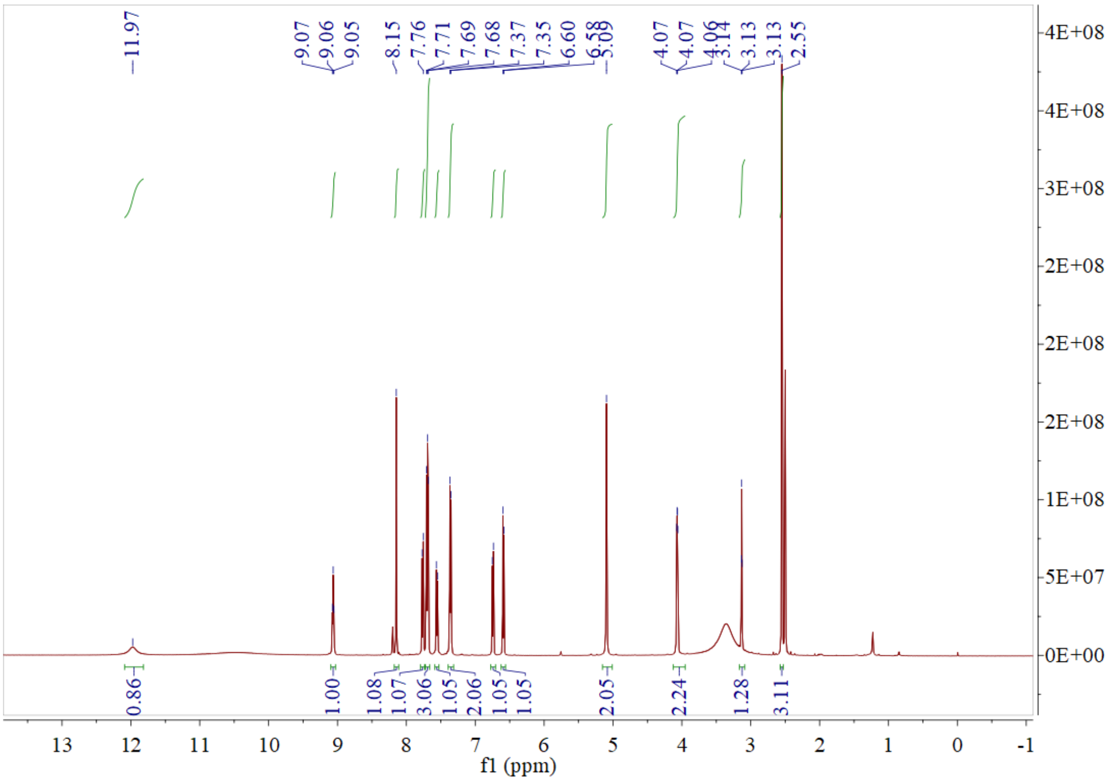
11.97
9.07
9.06
9.05
8.15
7.76
7.71
7.69
7.68
7.37
7.35
6.60
6.58
5.09
4.07
4.07
4.06
3.14
3.13
3.13
2.55
0.86
1.00
1.08
1.07
3.06
1.05
2.06
1.05
1.05
2.05
2.24
1.28
3.11
f1 (ppm)

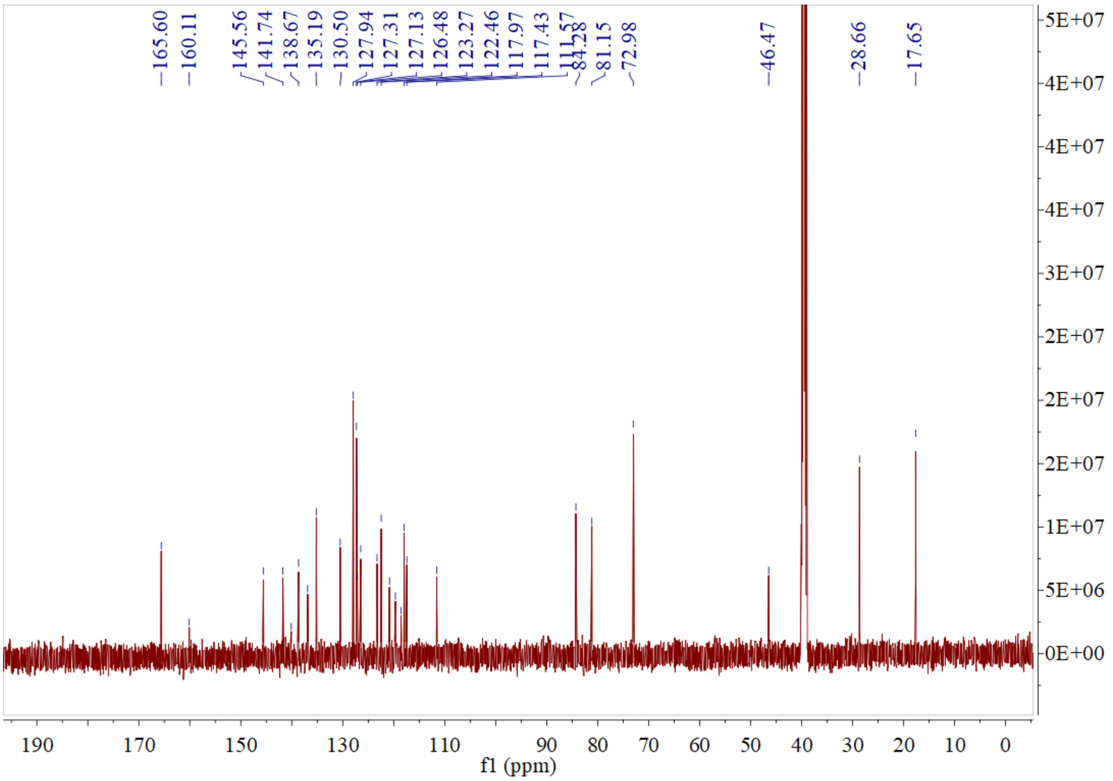
165.60
160.11
145.56
141.74
138.67
135.19
130.50
127.94
127.31
127.13
126.48
123.27
122.46
117.97
117.43
111.57
84.28
81.15
72.98
46.47
28.66
17.65
f1 (ppm)

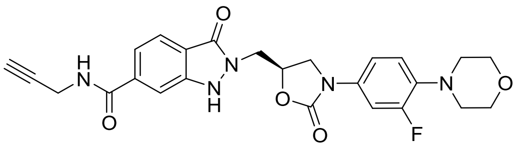

17

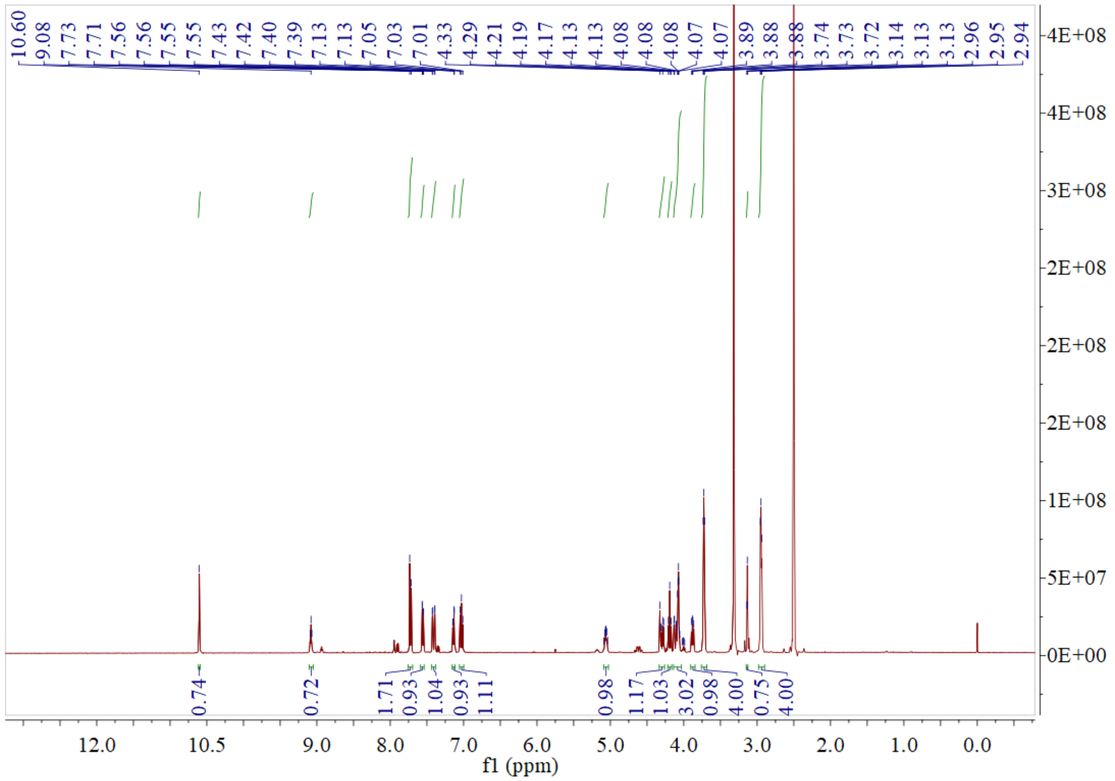

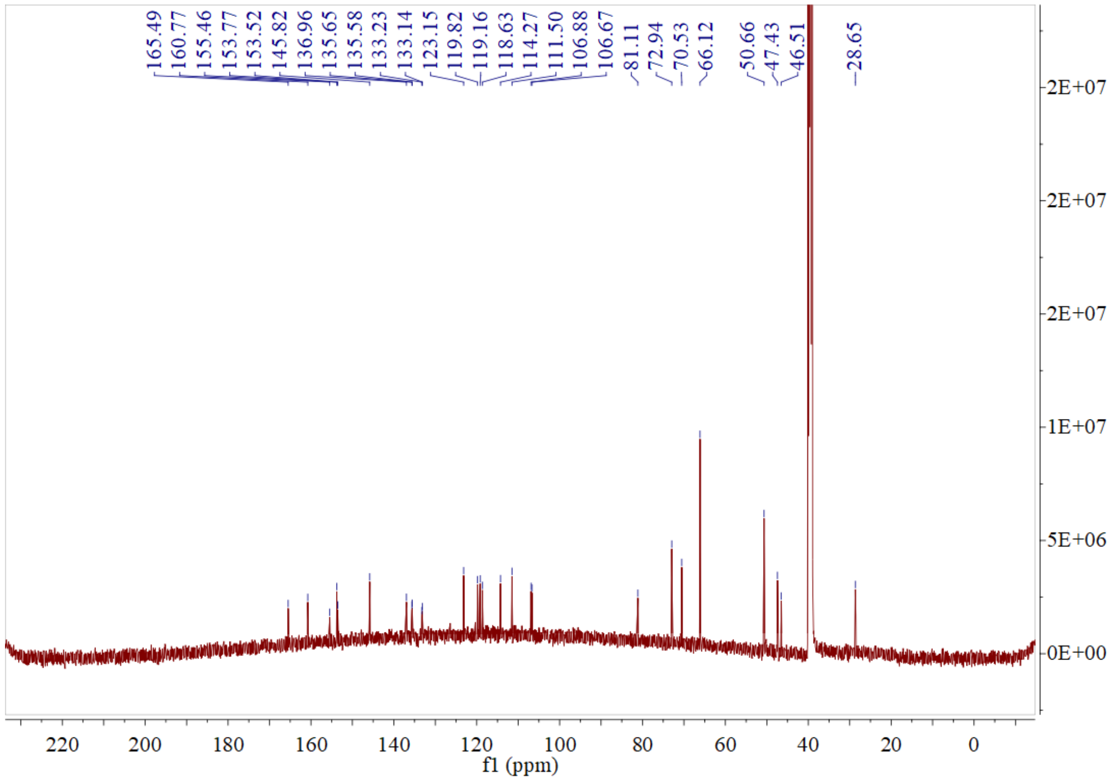

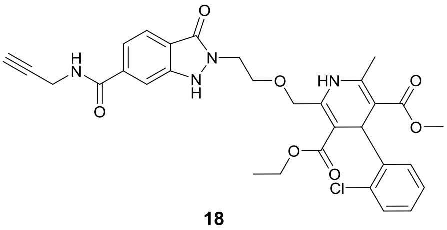

18

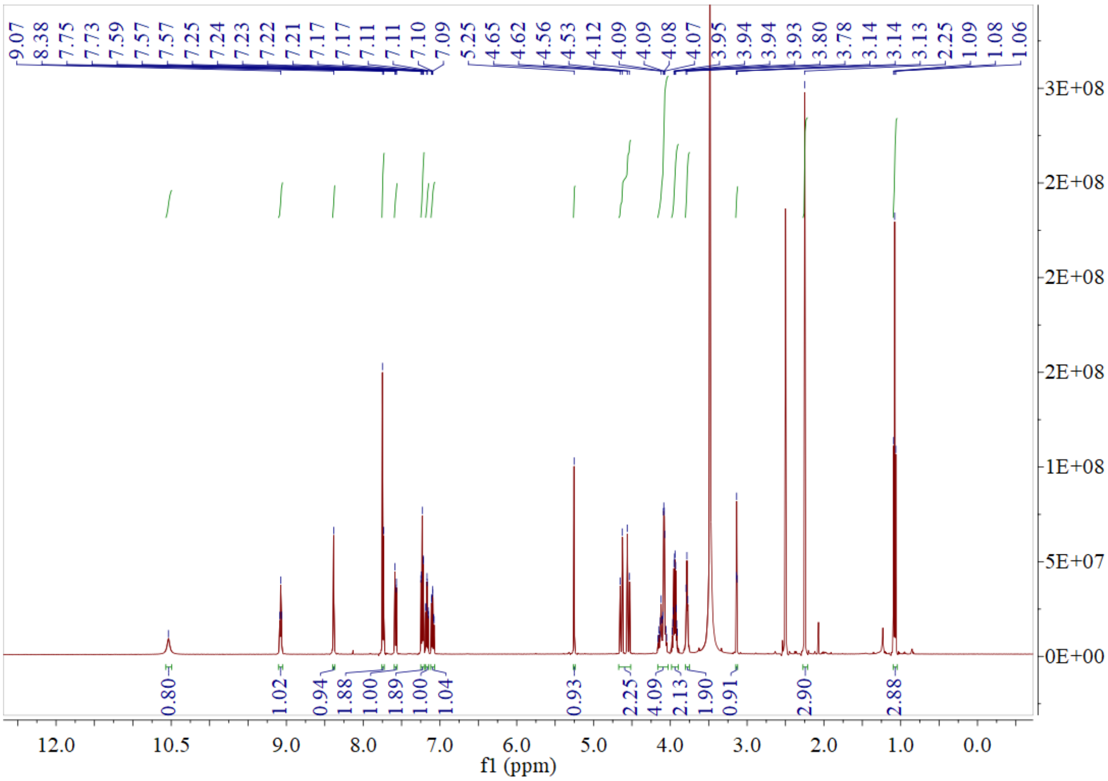

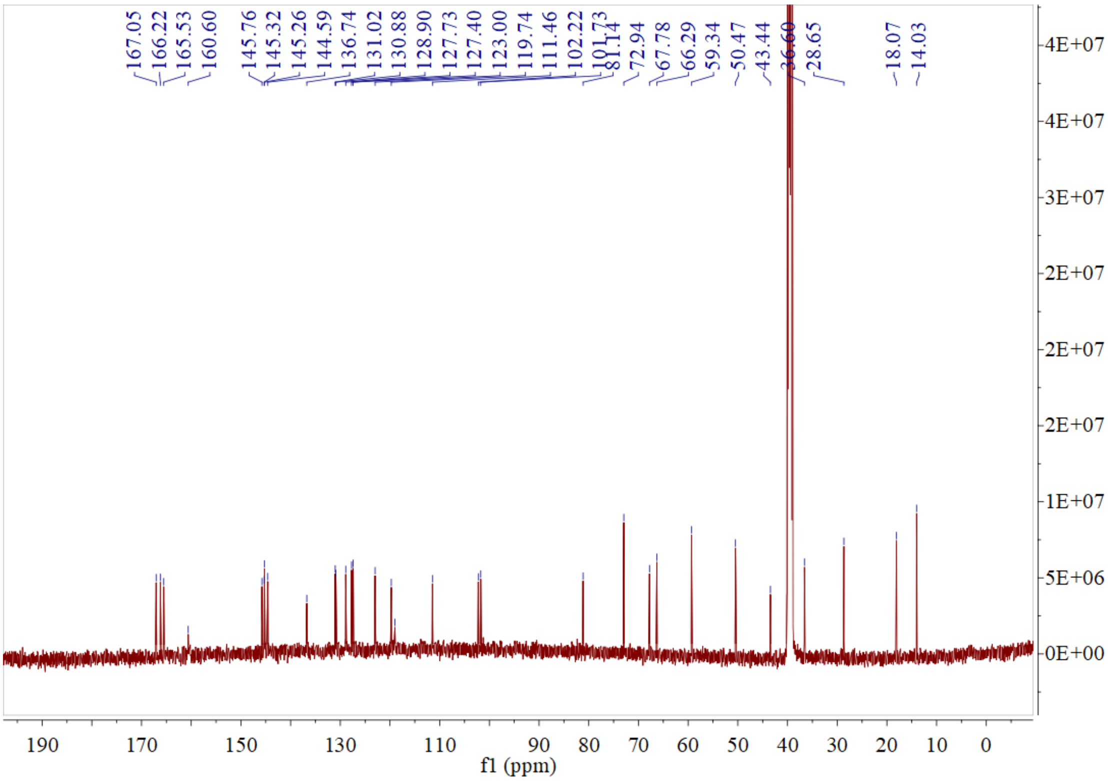

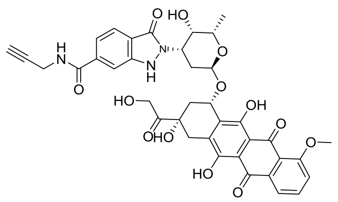
HO
O
N
H
N
H
O
HO
OH
O
O
HO
O
O

19

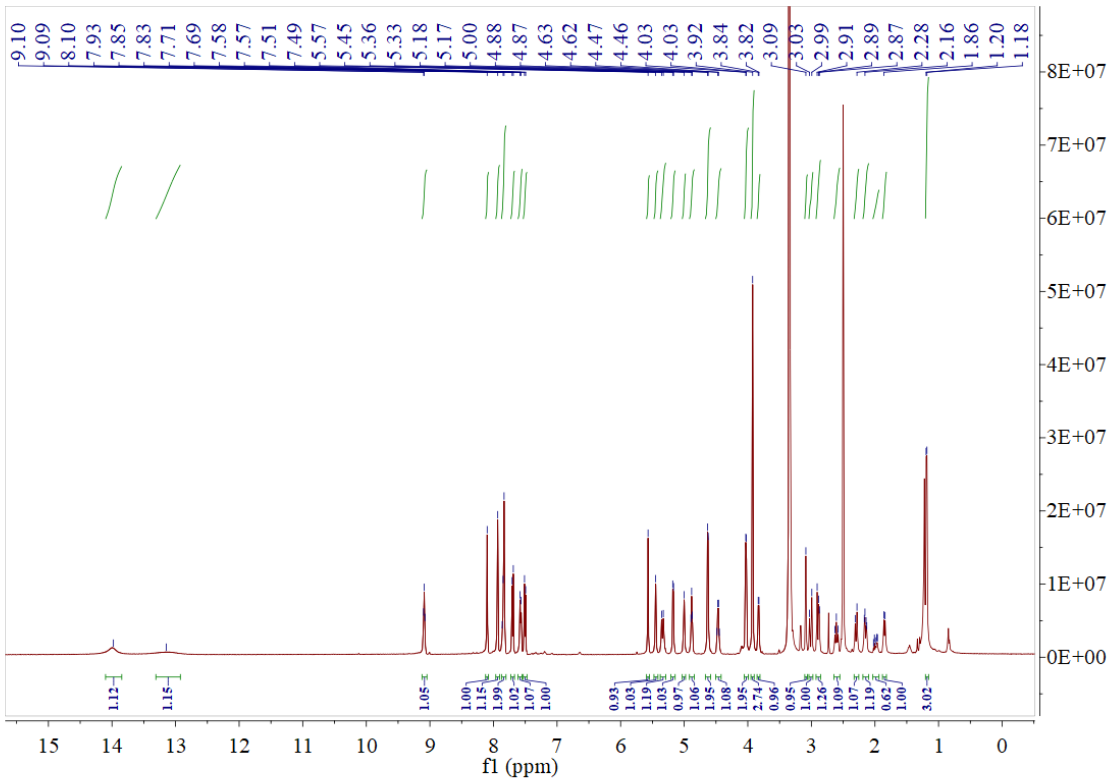
9.10
9.09
8.10
7.93
7.85
7.83
7.71
7.69
7.58
7.57
7.51
7.49
5.57
5.45
5.36
5.33
5.18
5.17
5.00
4.88
4.87
4.63
4.62
4.47
4.46
4.03
4.03
3.92
3.84
3.82
3.09
3.03
2.99
2.91
2.89
2.87
2.28
2.16
1.86
1.20
1.18
8E+07
7E+07
6E+07
5E+07
4E+07
3E+07
2E+07
1E+07
0E+00
1.12
1.15
1.05
1.00
1.15
1.99
1.02
1.07
1.00
0.93
1.03
1.19
1.03
0.97
1.06
1.95
1.08
1.95
2.74
0.96
0.95
1.00
1.26
1.09
1.07
1.19
0.62
1.00
3.02
15 14 13 12 11 10 9 8 7 6 5 4 3 2 1 0
f1 (ppm)

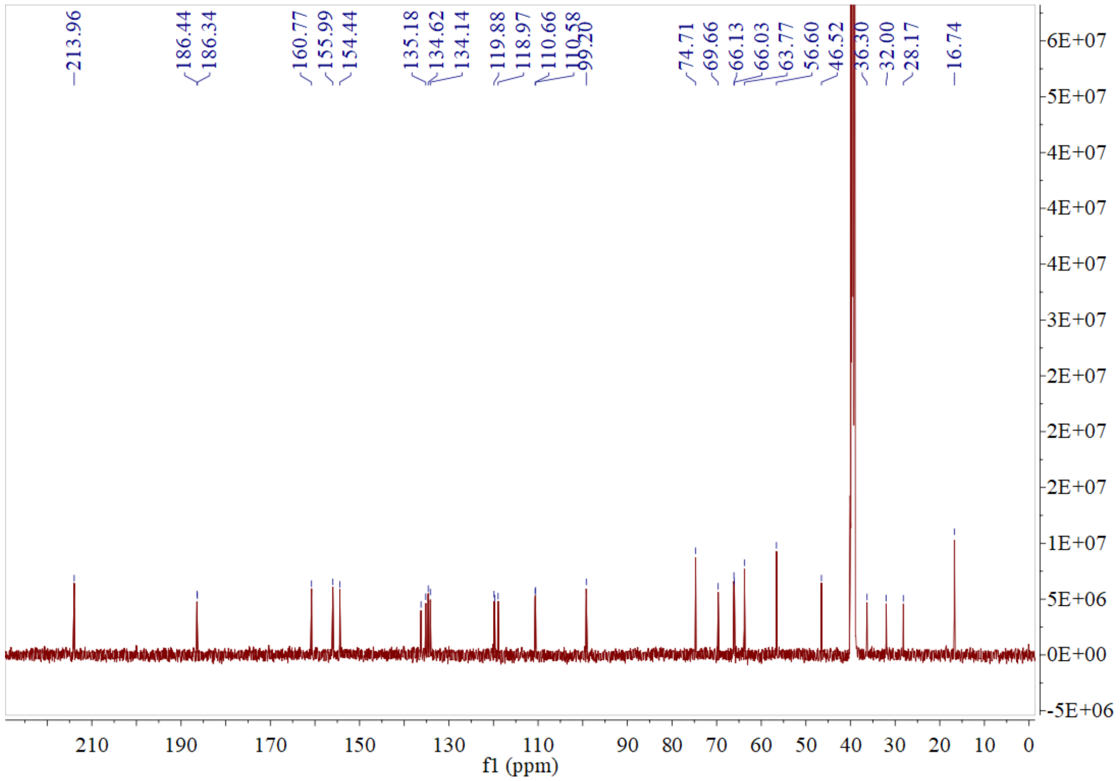
213.96
186.44
186.34
160.77
155.99
154.44
135.18
134.62
134.14
119.88
118.97
110.66
110.58
99.20
74.71
69.66
66.13
66.03
63.77
56.60
46.52
36.30
32.00
28.17
16.74
6E+07
5E+07
4E+07
4E+07
3E+07
2E+07
2E+07
1E+07
5E+06
0E+00
-5E+06
210 190 170 150 130 110 90 80 70 60 50 40 30 20 10 0
f1 (ppm)

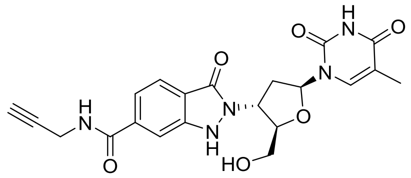

20

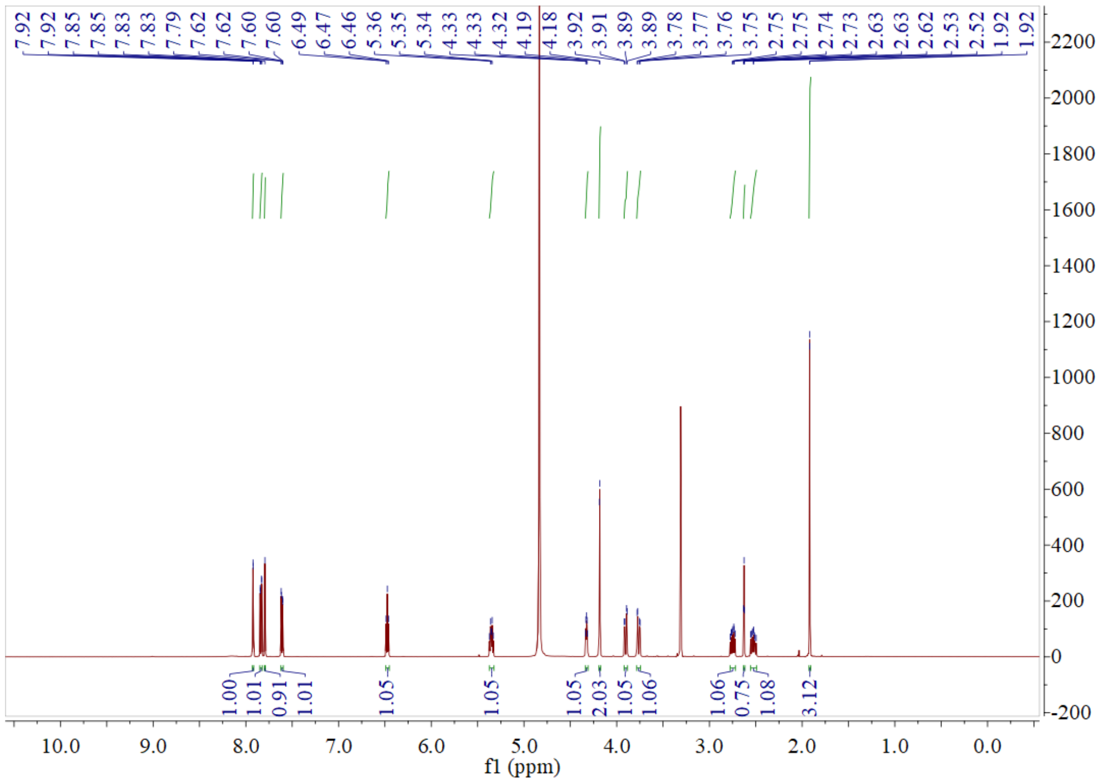

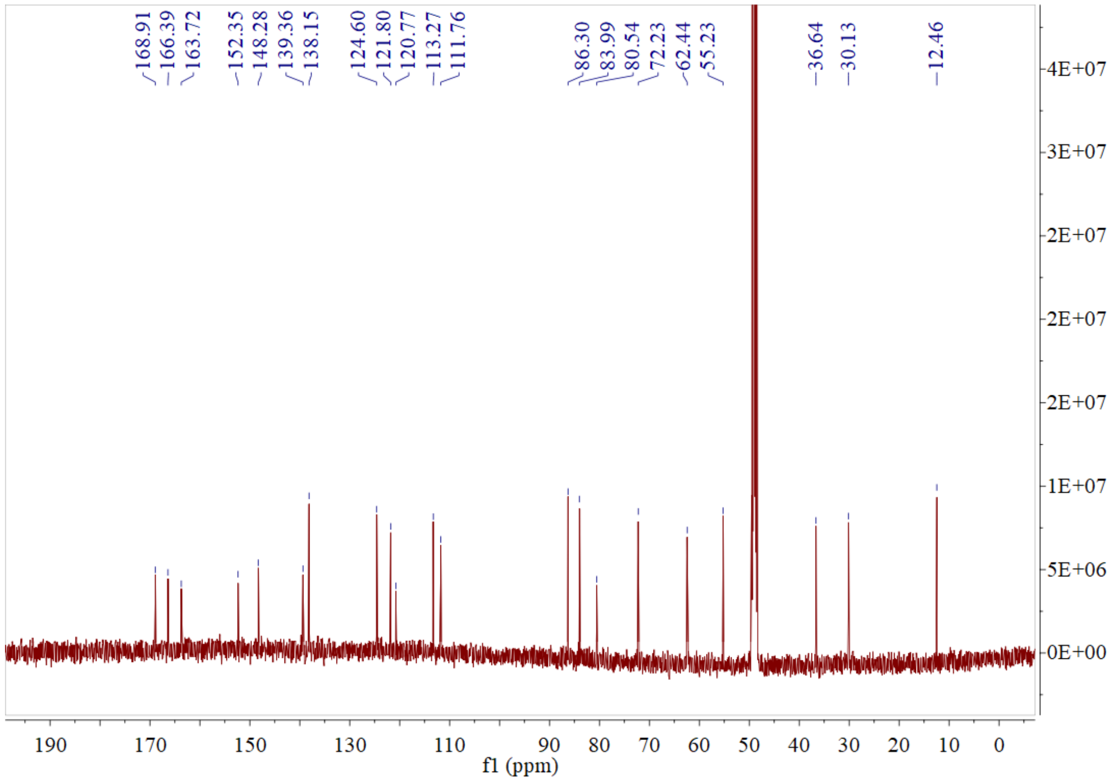

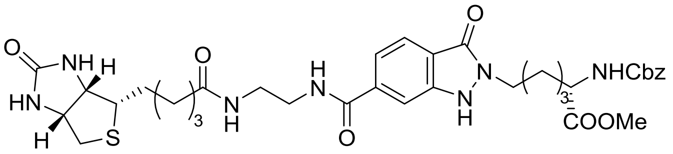
NHCbz
COOMe

21

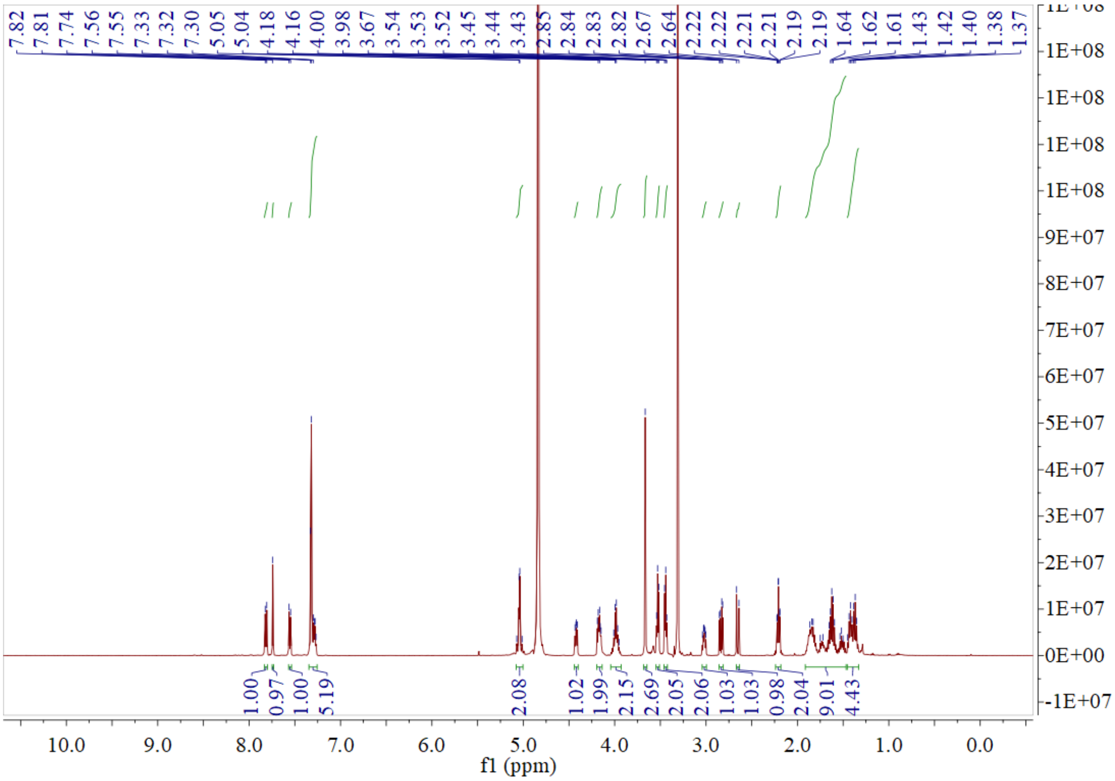
7.82
7.81
7.74
7.56
7.55
7.33
7.32
7.30
5.05
5.04
4.18
4.16
4.00
3.98
3.67
3.54
3.53
3.52
3.45
3.44
3.43
2.85
2.84
2.83
2.82
2.67
2.64
2.22
2.22
2.21
2.21
2.19
2.19
1.64
1.62
1.61
1.43
1.42
1.40
1.38
1.37
1.00
0.97
1.00
5.19
2.08
1.02
1.99
2.15
2.69
2.05
2.06
1.03
1.03
0.98
2.04
9.01
4.43
f1 (ppm)

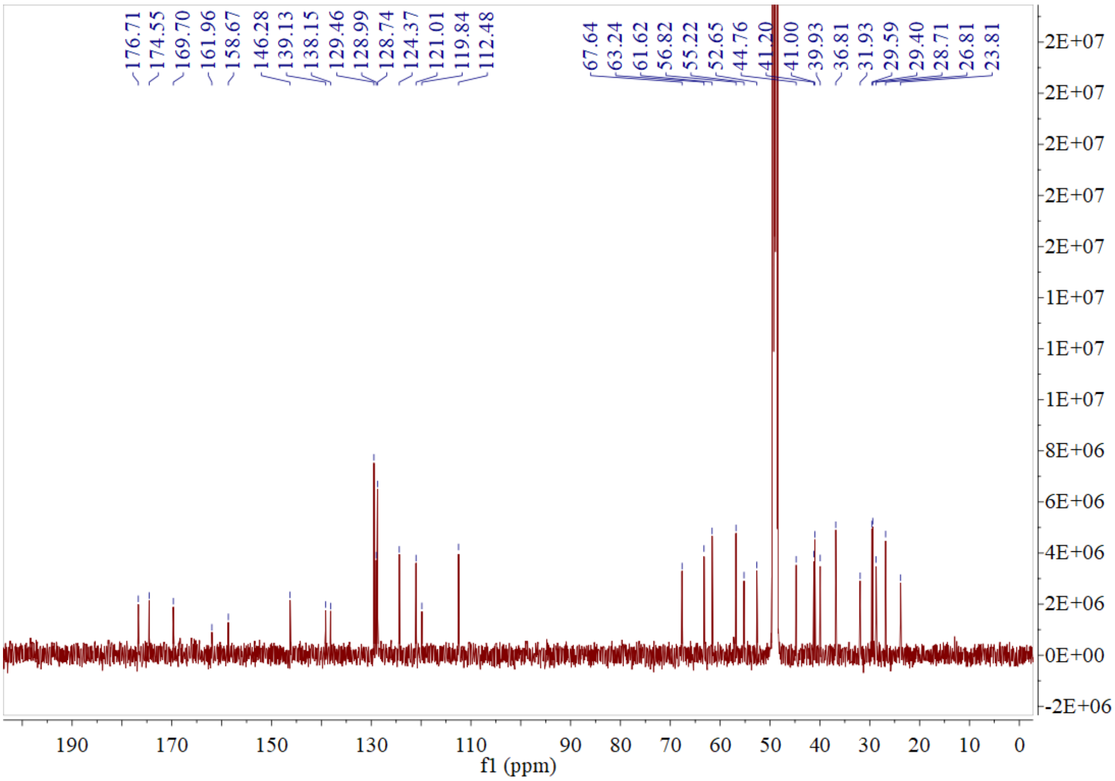
176.71
174.55
169.70
161.96
158.67
146.28
139.13
138.15
129.46
128.99
128.74
124.37
121.01
119.84
112.48
67.64
63.24
61.62
56.82
55.22
52.65
44.76
41.20
41.00
39.93
36.81
31.93
29.59
29.40
28.71
26.81
23.81
f1 (ppm)

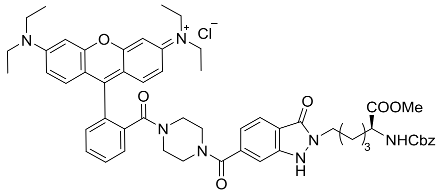
Cl⁻
COOMe
NHCbz

22

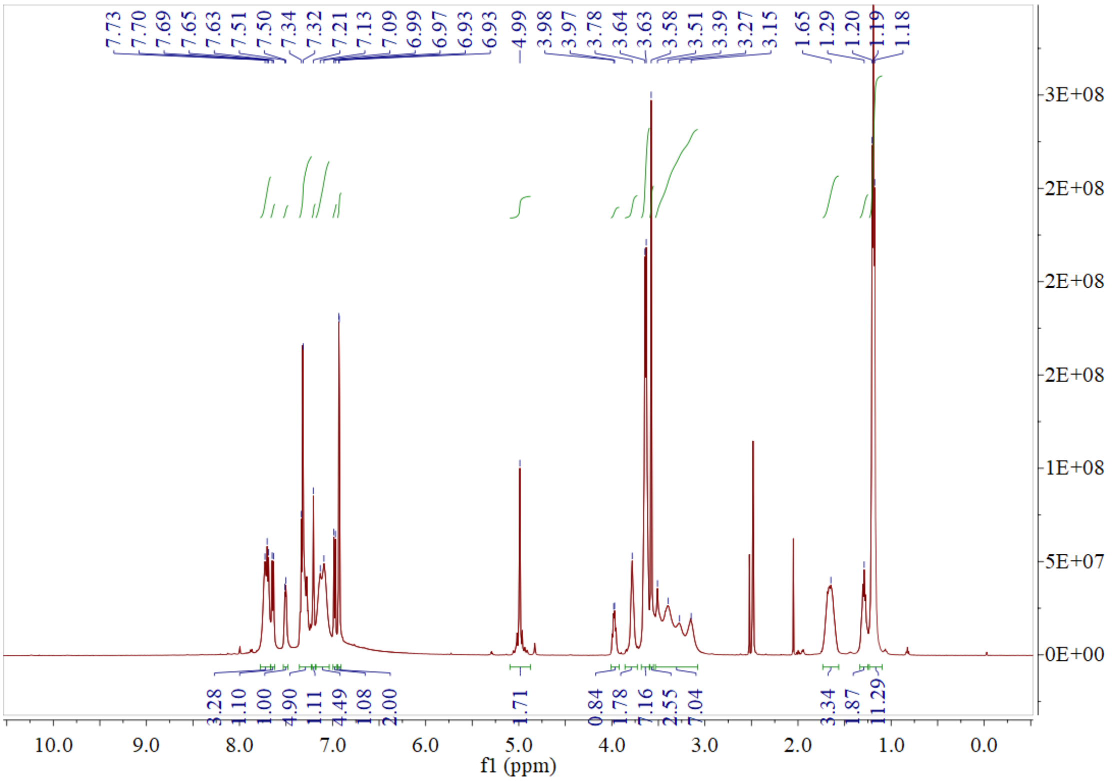
7.73 7.70 7.69 7.65 7.63 7.51 7.50 7.34 7.32 7.21 7.13 7.09 6.99 6.97 6.93 6.93 4.99 3.98 3.97 3.78 3.64 3.63 3.58 3.51 3.39 3.27 3.15 1.65 1.29 1.20 1.19 1.18
3.28 1.10 1.00 4.90 1.11 4.49 1.08 2.00 1.71 0.84 1.78 7.16 2.55 7.04 3.34 1.87 11.29
f1 (ppm)

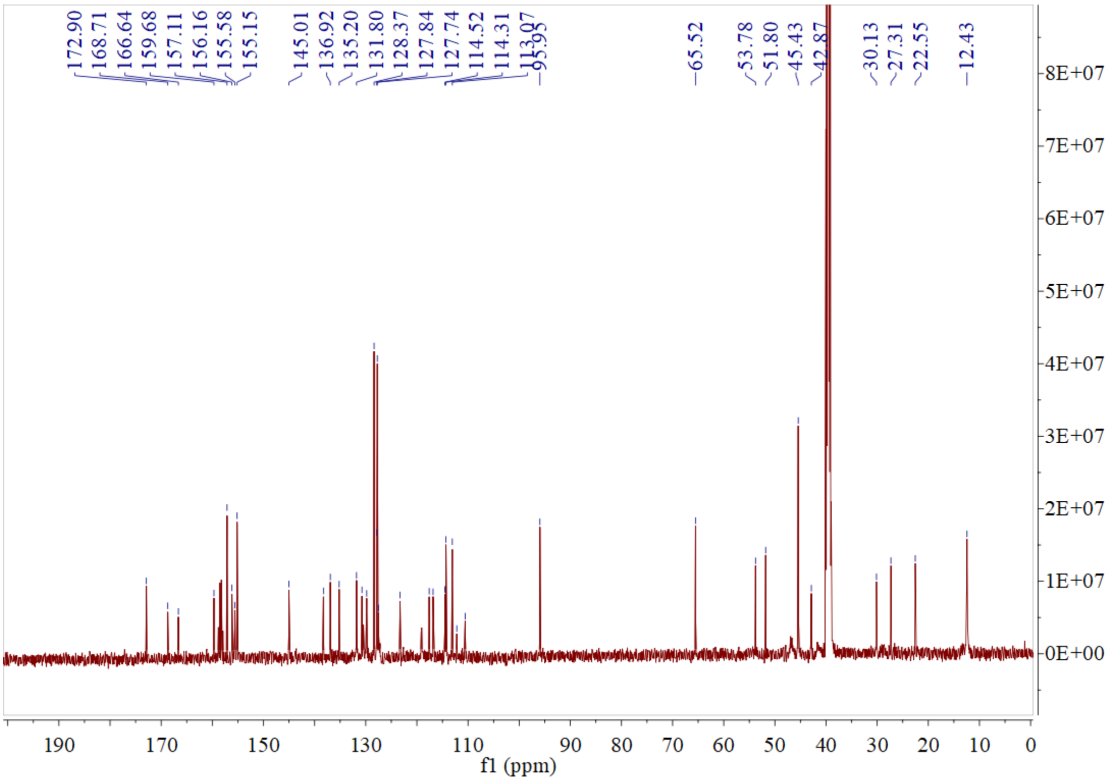
172.90 168.71 166.64 159.68 157.11 156.16 155.58 155.15 145.01 136.92 135.20 131.80 128.37 127.84 127.74 114.52 114.31 113.07 95.95 65.52 53.78 51.80 45.43 42.87 30.13 27.31 22.55 12.43
f1 (ppm)

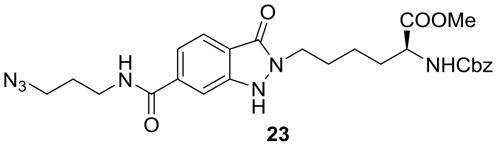

23

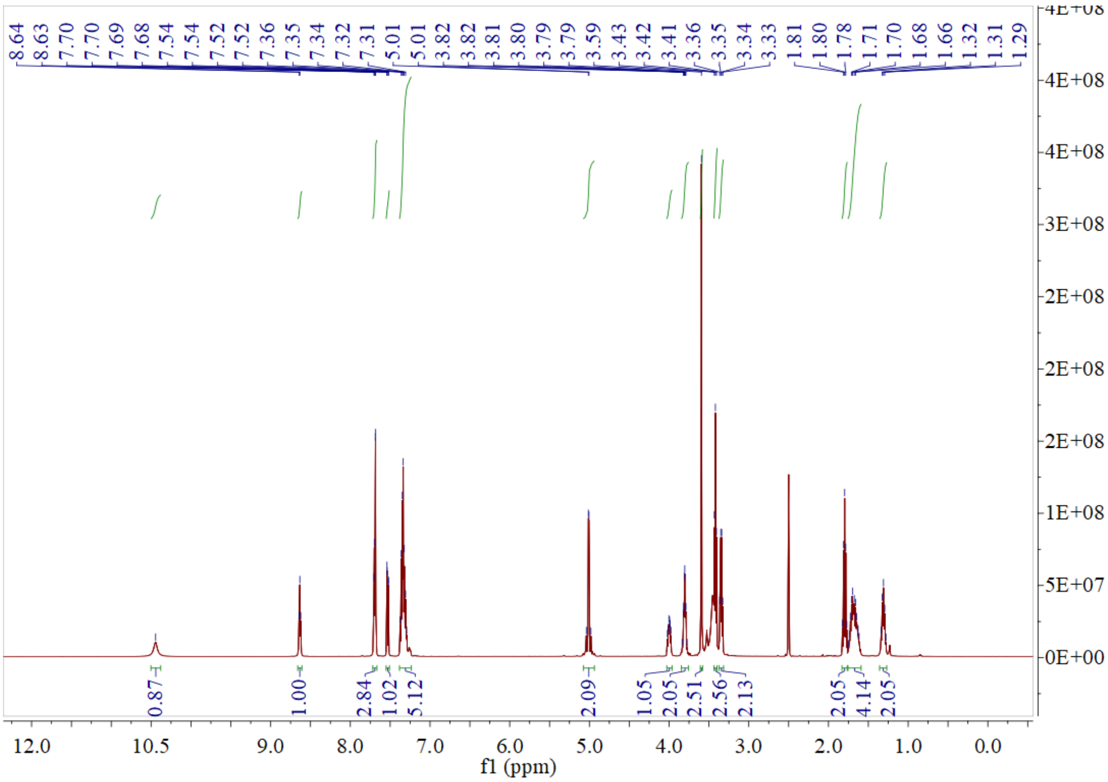

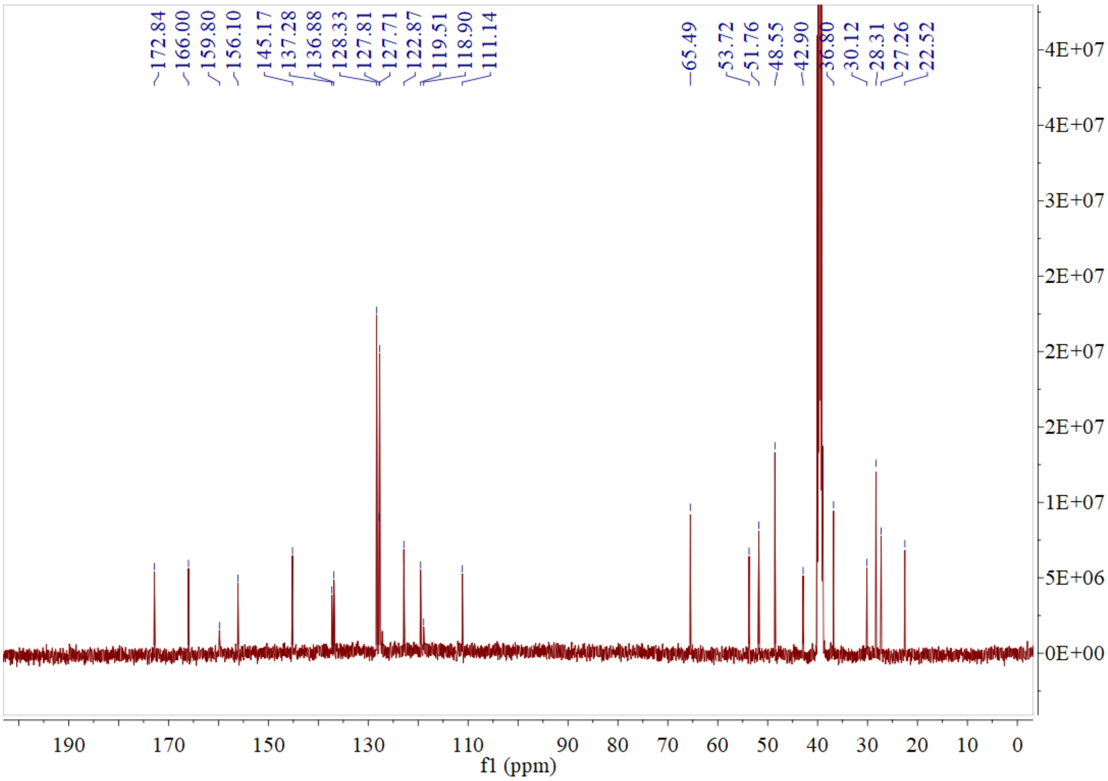

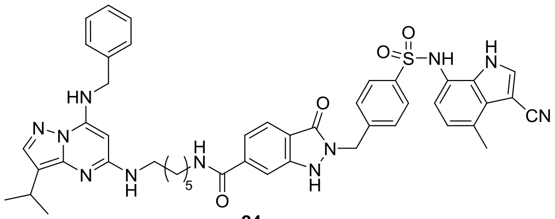

HN
N
N
N
H
5
H
N
O
O
N
N
H
O
S
O
NH
H
N
CN

24

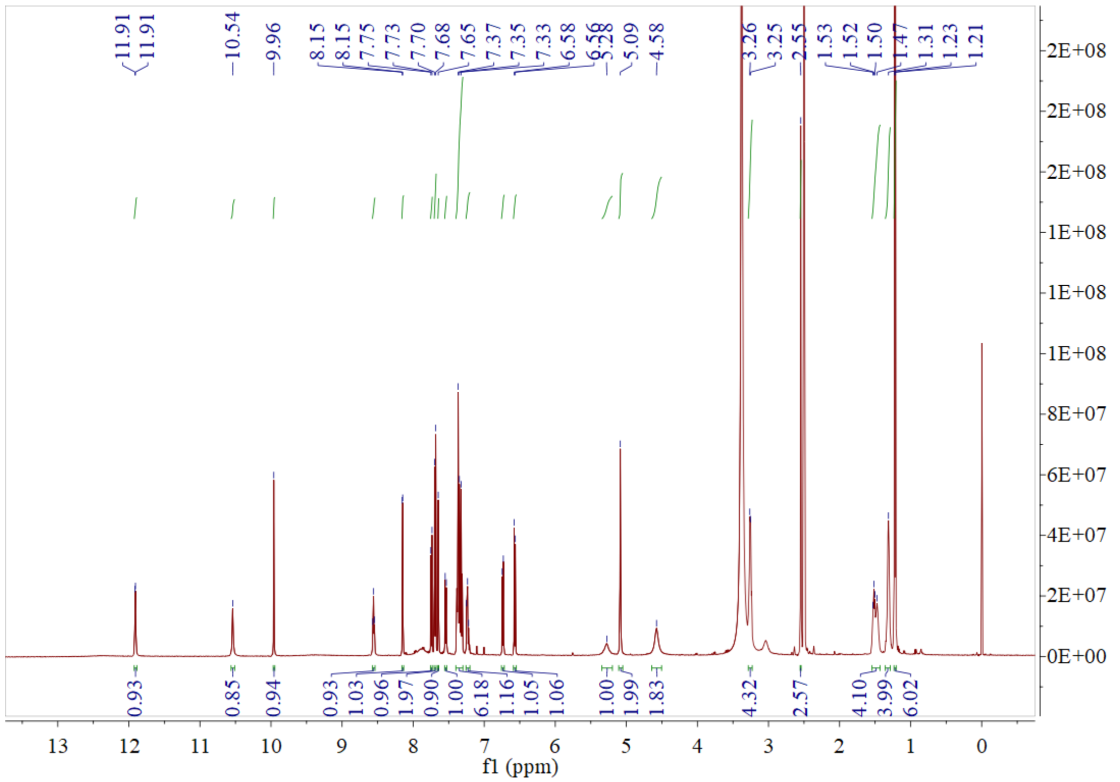

11.91
11.91
10.54
9.96
8.15
8.15
7.75
7.73
7.70
7.68
7.65
7.37
7.35
7.33
6.58
6.56
5.28
5.09
4.58
3.26
3.25
2.55
1.53
1.52
1.50
1.47
1.31
1.23
1.21
0.93
0.85
0.94
0.93
1.03
0.96
1.97
0.90
1.00
6.18
1.16
1.05
1.06
1.00
1.99
1.83
4.32
2.57
4.10
3.99
6.02
f1 (ppm)

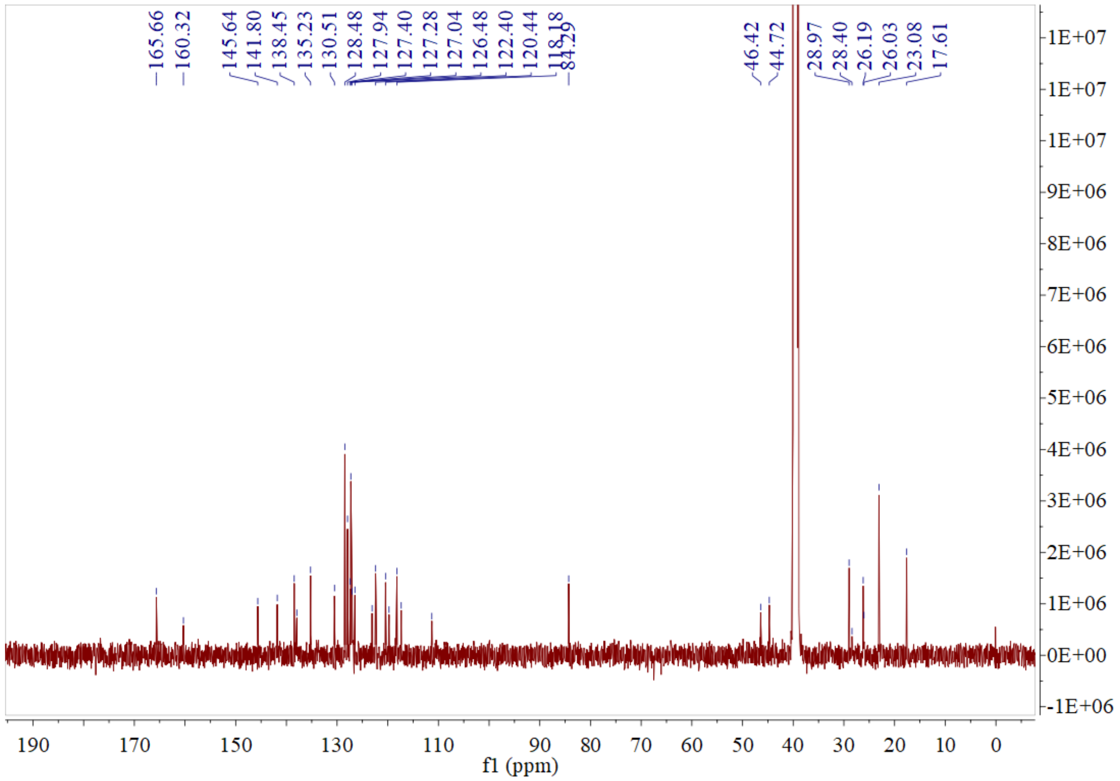

165.66
160.32
145.64
141.80
138.45
135.23
130.51
128.48
127.94
127.40
127.28
127.04
126.48
122.40
120.44
118.18
84.29
46.42
44.72
28.97
28.40
26.19
26.03
23.08
17.61
f1 (ppm)

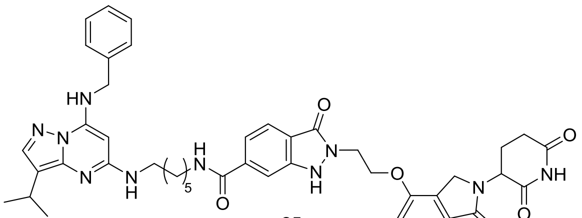

25

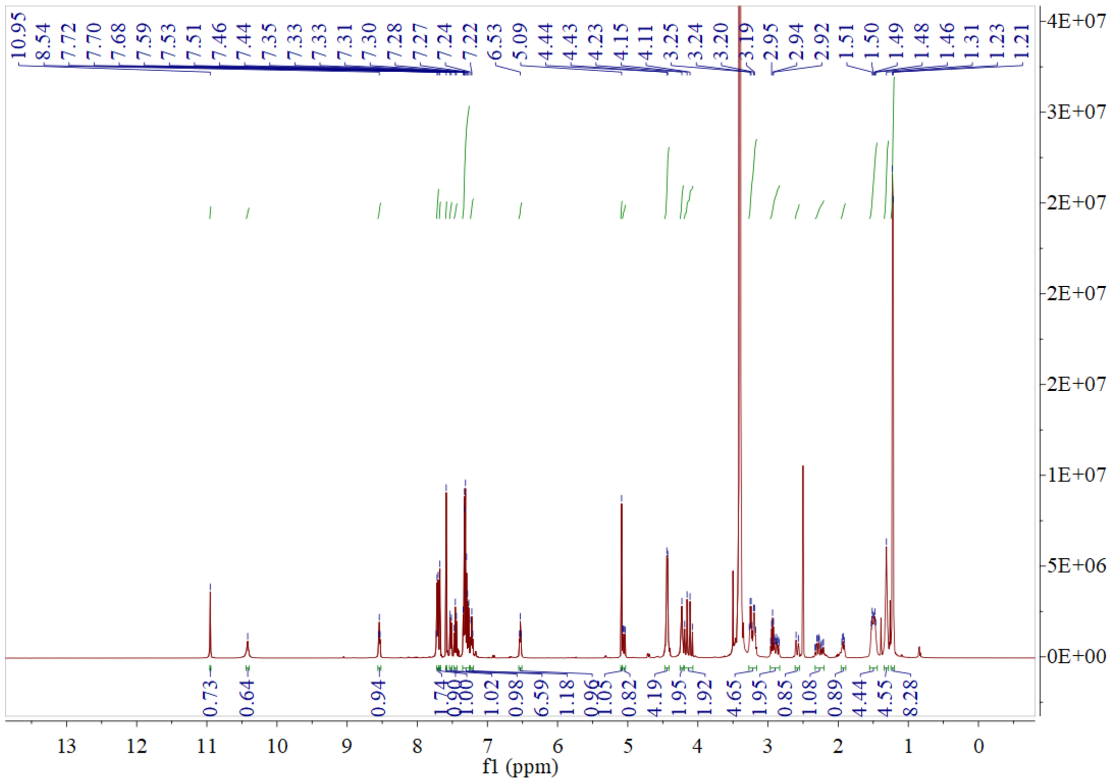

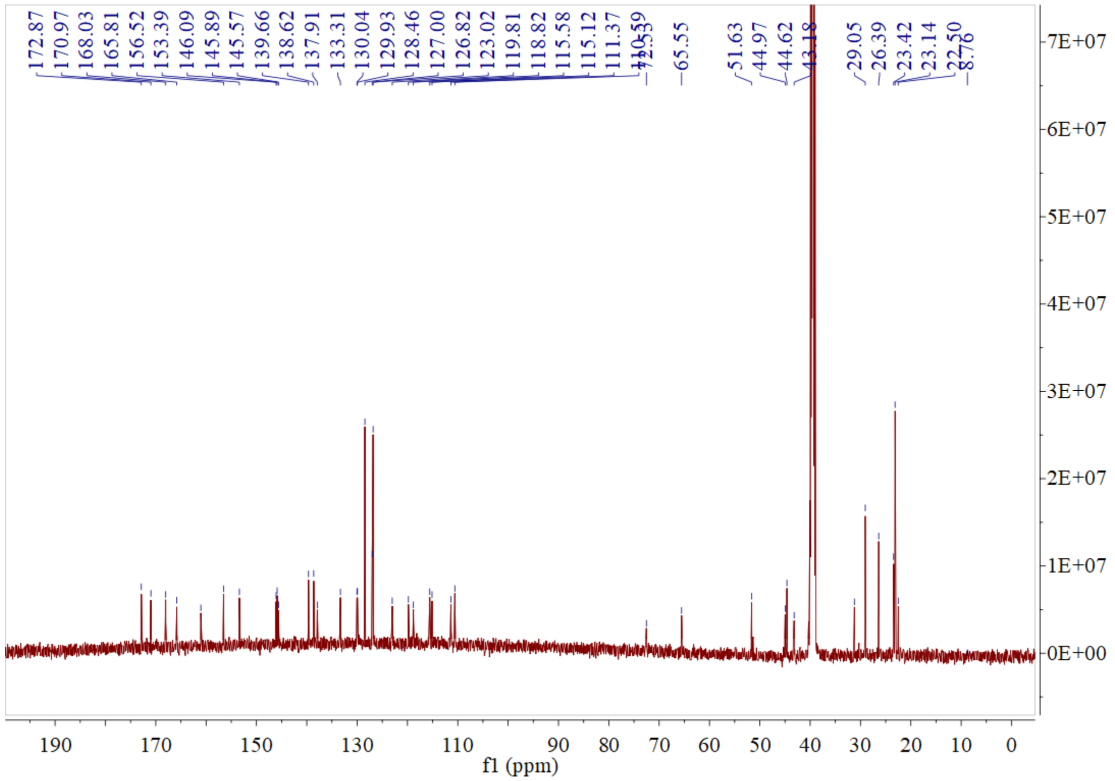

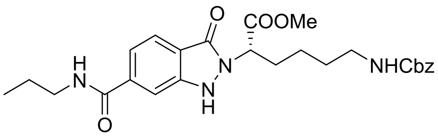
COOMe
NHCbz
O
N
H

S36

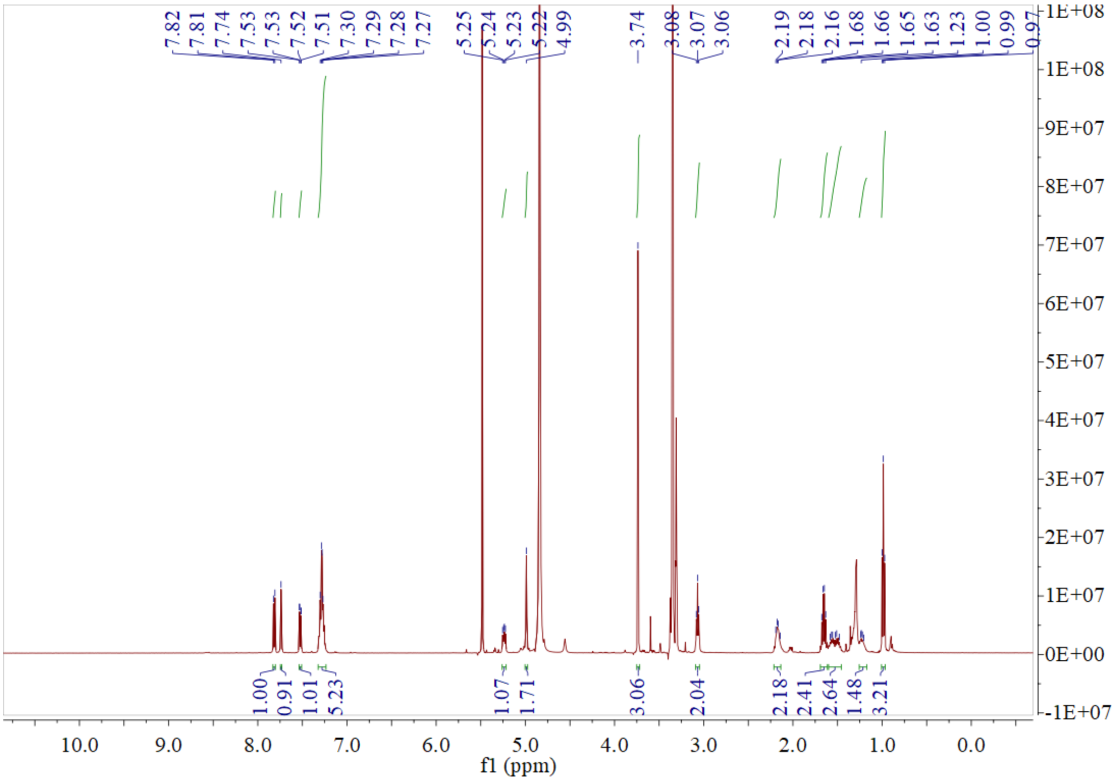
7.82 7.81 7.74 7.53 7.53 7.52 7.51 7.30 7.29 7.28 7.27 5.25 5.24 5.23 5.22 4.99 3.74 3.08 3.07 3.06 2.19 2.18 2.16 1.68 1.66 1.65 1.63 1.23 1.00 0.99 0.97
1.00 0.91 1.01 5.23 1.07 1.71 3.06 2.04 2.18 2.41 2.64 1.48 3.21
f1 (ppm)

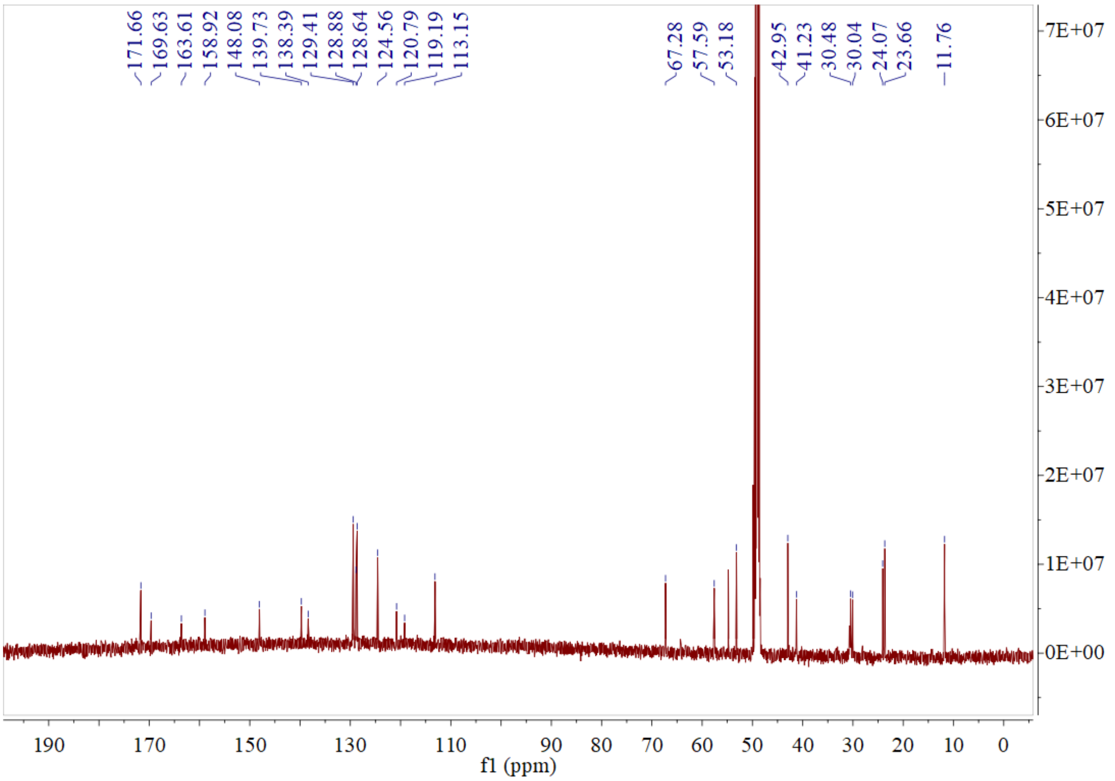
171.66 169.63 163.61 158.92 148.08 139.73 138.39 129.41 128.88 128.64 124.56 120.79 119.19 113.15 67.28 57.59 53.18 42.95 41.23 30.48 30.04 24.07 23.66 11.76
f1 (ppm)

9. Full Gel Scan

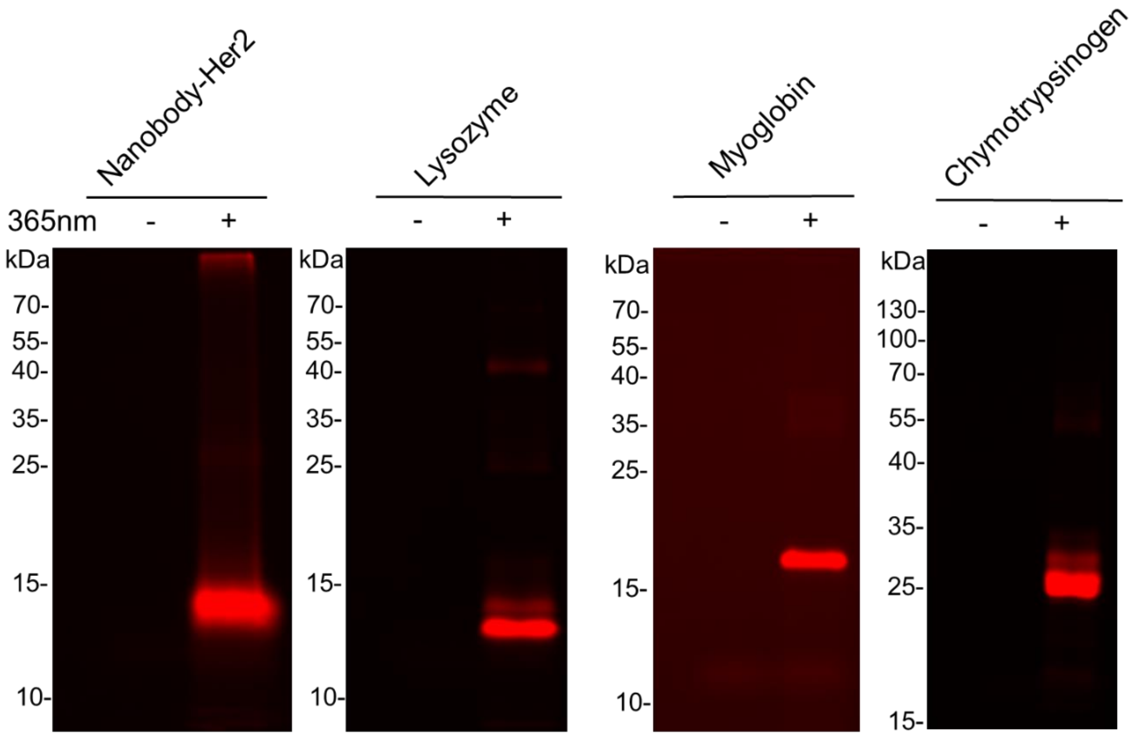

10. Supplementary References

1. Massa, S. *et al.* Site-specific labeling of cysteine-tagged camelid single-domain antibody-fragments for use in molecular imaging. *Bioconjug Chem* 2014, **25**(5)**:** 979-988.
2. Serfling, R. *et al.* NAR Breakthrough Article Designer tRNAs for efficient incorporation of non-canonical amino acids by the pyrrolysine system in mammalian cells. *Nucleic Acids Research* 2018, **46:** 1–10.
3. Lee J., Kim J. & Choi W. Ferrioxalate-Polyoxometalate System as a New Chemical Actinometer. *Environ. Sci. Technol.* 2007, **41:** 5433-5438.
4. Yu Z. & Ohulchanskyy T.Y., An P, Prasad PN & Lin Q. Fluorogenic, two-photon-triggered photoclick chemistry in live mammalian cells. *J Am Chem Soc* 2013, **135**(45)**:** 16766-16769.
5. Ban, H., Gavrilyuk, J. & Barbas, C.F. Tyrosine Bioconjugation through Aqueous Ene-Type Reactions: A Click-Like Reaction for Tyrosine. *J. Am. Chem. Soc.* **132**, 1523-1525 (2010).
6. Wan, P. & Yates, K. Photochemical Oxidation of Nitrobenzyl Alcohols in Aqueous-Solution. *J. Chem. Soc., Chem. Commun.* 1023-1024 (1981).
7. Ellis, M.K., Hill, S. & Foster, P.M.D. Reactions of Nitrosonitrobenzenes with Biological Thiols - Identification and Reactivity of Glutathione-S-Yl Conjugates. *Chem.-Biol. Interact.* **82**, 151-163 (1992).
8. Eyer, P. Reactions of Nitrosobenzene with Reduced Glutathione. *Chem.-Biol. Interact.* **24**, 227-239 (1979).
9. Alamudi, S.H. *et al.* Development of background-free tame fluorescent probes for intracellular live cell imaging. *Nat Commun* 2016, **7:** 11964.
10. Chen, X.H. Isoindoline compound, and preparation method, pharmaceutical composition and application thereof. WO 2020064002.
11. Hansen, J. D. *et al.* Protein Degradation via CRL4^{CRBN} Ubiquitin Ligase: Discovery and Structure−Activity Relationships of Novel Glutarimide Analogs That Promote Degradation of Aiolos and/or GSPT1. *J. Med. Chem.* **61,** 492−503 (2018).
12. Uehara, T. *et al.* Selective Degradation of Splicing Factor CAPER by Anticancer Sulfonamides. *Nat. Chem. Biol.* **13,** 675-680 (2017).
13. Ali, S. *et al*. The Development of a Selective Cyclin-Dependent Kinase Inhibitor That Shows Antitumor Activity. *Cancer Res.* **69**, 6208-6215 (2009).
14. Zhao, Q. et al. Broad-Spectrum Kinase Profiling in Live Cells with Lysine-Targeted Sulfonyl Fluoride Probes. **139,** 680-685 (2017).
15. Schembri, L.S. *et al.* Synthesis, Biological Evaluation, and Utility of Fluorescent Ligands Targeting the mu-Opioid Receptor. *J Med Chem* 2015, **58**(24)**:** 9754-9767.
16. Krall, N., Pretto, F., Decurtins, W., Bernardes, G.J., Supuran, C.T., Neri, D. A small-molecule drug conjugate for the treatment of carbonic anhydrase IX expressing tumors. *Angew Chem Int Ed Engl* 2014, **53**(16)**:** 4231-4235.
